# Confined Acids Catalyze a Broadly Applicable β‑Selective *O*‑Glycosylation

**DOI:** 10.1021/jacs.6c01167

**Published:** 2026-04-06

**Authors:** Jiaxiang Lu, Tianyu Zheng, Satoshi Matsutani, Nobuya Tsuji, Chendan Zhu, Markus Leutzsch, Benjamin List

**Affiliations:** † 28314 Max-Planck-Institut für Kohlenforschung, 45470 Mülheim an der Ruhr, Germany; ∥ Graduate School of Chemical Sciences and Engineering, 12810 Hokkaido University, Sapporo 060-8628, Japan; § Institute for Chemical Reaction Design and Discovery, Hokkaido University, Sapporo 001-0021, Japan

## Abstract

Glycans are fundamental components of biological systems, composed of monosaccharides linked through glycosidic bonds. The stereogenic nature of these glycosidic bonds plays a decisive role in the structure and biological function of the glycan. Achieving precise, stereocontrolled formation of glycosidic linkages has therefore been a long-standing objective and an ongoing challenge for chemists. To date, general catalytic β-selective glycosylations have been limited to S_N_2-like reactions with competing S_N_1-like pathways via oxocarbenium ions eroding the selectivity. Here we report an alternative approach: confined acids catalyze a broadly applicable β-selective *O*-glycosylation, including 2-deoxyglucosylation, glucosylation, and mannosylation, that we suggest proceeds via an S_N_1-like pathway. Mechanistic and theoretical studies support this view.

## Graphical abstract

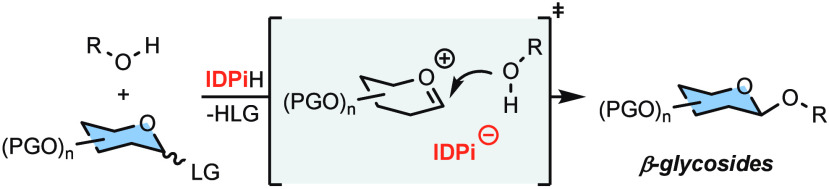

Glycans and their derivatives play key roles in life sciences, including energy storage, structural support, cellular recognition, and drug development.^1,2^ They are built up from monosaccharides usually linked through ethereal *O*-glycosidic bonds, which can be α- or β-configured, thereby determining form and function of the glycan. Formation of glycosidic bonds typically involves the displacement of a leaving group from a glycosyl donor by a glycosyl acceptor. Enzymes have been shown to catalyze glycosylations with high stereoselectivity, albeit with limited generality.^3^ Compared to α-selective glycosylation, which is favored by the anomeric effect, β-selective glycosylation is chemically more challenging.^4–6^ Readily available α-glycosyl donors can be employed in a stereospecific, catalytic S_N_2-like reaction, inverting the anomeric center and therefore yielding β-configured glycosides (Scheme 1A). Among several examples,^7–15^ Jacobsen and co-workers demonstrated general β-glycosylation reactions using glycosyl chlorides and phosphates, catalyzed by chiral bis-thiourea catalysts.^16–21^ However, in all S_N_2-like approaches, competing S_N_1-like pathways can erode the β-selectivity.^4^ To increase the β-selectivity, a catalyst must differentiate the diastereotopic faces of the oxocarbenium intermediate of an S_N_1-like pathway, thereby overriding the inherent stereopreferences of the chiral glycosyl donors and acceptors (Scheme 1B). Our group has reported that reactions of aliphatic oxocarbenium ions can be controlled by highly acidic, confined imidodiphosphorimidate (IDPi) catalysts.^22,23^ Here we show that IDPi catalysts are also able to control complex oxocarbenium ions^24^ generated by glycosyl donors and realize a broadly applicable approach to β-selective glycosylation.

**Scheme 1:**
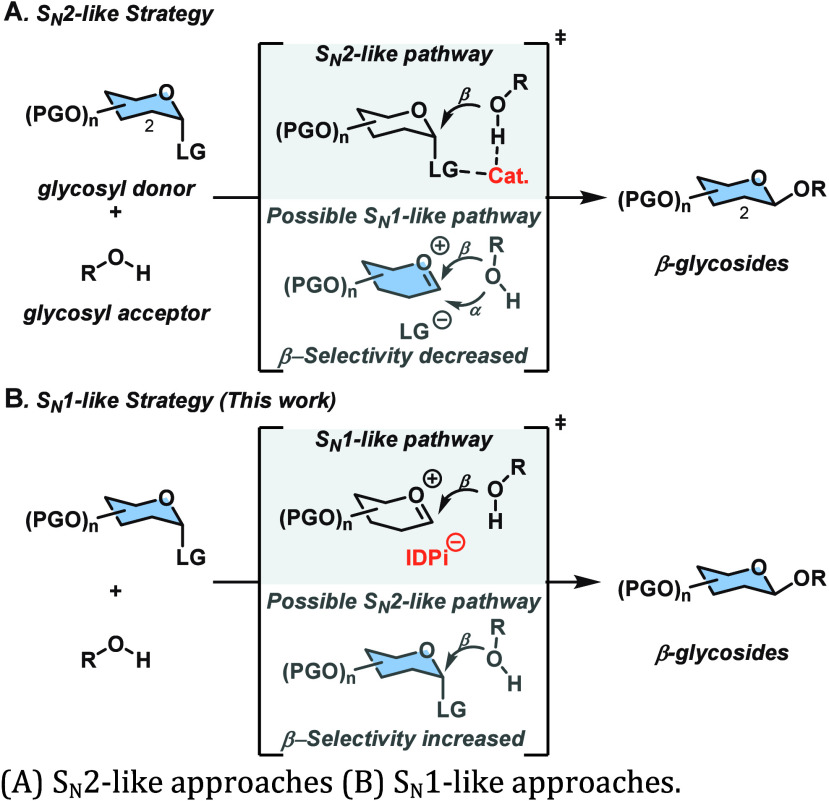
Catalytic Glycosylation

At the outset of this investigation, we chose glycosyl donors **1a**–**1c**, with different 2-substituents, as our model substrates with benzyl protecting groups to avoid anchimeric effects or conformational benefits.^25–28^ Trichloroacetimidate was employed as the leaving group to facilitate the generation of the oxocarbenium ion under acidic conditions.^29,30^ We identified (*R*,*R*)-IDPi **3a** as the optimum catalyst with glycosyl acceptor **2a** for a β-selective 2-deoxyglucosylation yielding β-1,6-2-deoxyglucoside **4a** with over 95:5 β-selectivity and full conversion (Table 1, entry 1). Notably, catalytic 2-deoxyglucosylation has been achieved only with moderate β-selectivity. Next, (*R*,*R*)-IDPi **3b** was found to be the optimal catalyst for β-selective glucosylations, where the glycosyl donor **1b** bears equatorial 2-substituents. (*R*,*R*)-IDPi **3b** was found to be the optimal catalyst delivering β-1,6-glucoside **5a**, which was obtained with 94:6 β-selectivity (Table 1, entry 5). One prominent challenge is the so-called “1,2-*cis* glycoside problem”, as β-mannosidic linkages are unfavored by the strong thermodynamic anomeric preference for the α-isomer, the absence of neighboring group participation, and steric hindrance from the axial C2 substituent. This challenge is further amplified when glycosyl donors bear conformationally flexible protecting groups, such as benzyl groups.^31^ To our delight, (*S*,*S*)-IDPi **3c** proved to be suitable for this transformation and delivered β-mannoside **6a** with 95:5 selectivity (Table 1, entry 9). The pronounced matched/mismatched effect (see Table S7) highlights the important role of catalyst stereochemistry, with the ion pair formed between IDPi and glycosyl donor governing the stereoselectivity.

**Table 1:**
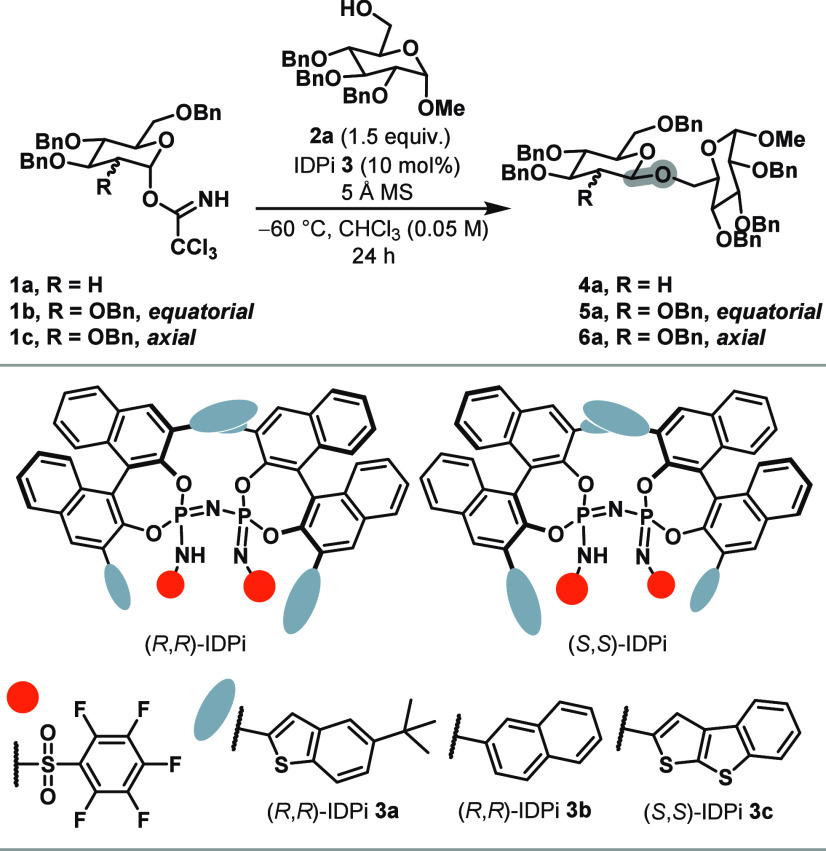
Catalyst Evaluation^a^

entry	IDPi **3**	product	β:α	conv. (%)
1	(*R*,*R*)-IDPi **3a**	**4a**	>95:5	99
2	(*R*,*R*)-IDPi **3a**	**5a**	83:17	99
3	(*R*,*R*)-IDPi **3a**	**6a**	29:71	99
4	(*R*,*R*)-IDPi **3b**	**4a**	71:29	99
5	(*R*,*R*)-IDPi **3b**	**5a**	94:6	99
6	(*R*,*R*)-IDPi **3b**	**6a**	67:33	99
7	(*S*,*S*)-IDPi **3c**	**4a**	50:50	99
8	(*S*,*S*)-IDPi **3c**	**5a**	80:20	99
9	(*S*,*S*)-IDPi **3c**	**6a**	95:5	99

a The reactions were carried out with glycosyl donor **1** (0.01 mmol), IDPi **3** (10 mol %), 5 Å molecular sieves (10 mg), and glycosyl acceptor **2a** (0.015 mmol) in chloroform (0.2 mL) at −60 °C for 24 h. β:α ratios and conversions were determined by crude ^1^H NMR spectroscopic analysis.

Next, we evaluated the formation of β-glycosides bearing (1,1)-, (1,2)-, (1,3)-, (1,4)-, and (1,6)-linkages under the optimized conditions (Figure 1). These reactions afforded β-glycosides **4a**–**4f**, **5a**–**5f**, **6a**–**6f**, and **7** in good to high yields (59–99%) with good to excellent β-selectivities (78:22 to >95:5), demonstrating the broad applicability of the catalytic system presented herein. In glycoproteins, glycans are most commonly attached through l-threonine, a linkage that plays a central role in regulating protein structure, recognition, and biological function. We therefore evaluated a protected l-threonine **2g** as a glycosyl acceptor, affording β-glycosides **4g** (94:6), **5g** (>95:5), and **6g** (88:12) with good to excellent selectivities. The achiral glycosyl acceptor *i*-PrOH also performed well under optimal conditions, producing β-glycosides **4h** (>95:5), **5h** (>95:5), and **6h** (86:14) with synthetically useful selectivities. These results indicate that the catalyst, rather than the chiral glycosyl acceptors, predominantly controls the selectivity. In addition to benzyl-protected donors, acetonide protected glycosyl donor **1d** also undergoes β-selective glycosylation with over 95:5 β-selectivity, demonstrating the capability of the established system (Figure 1B).

**Figure 1:**
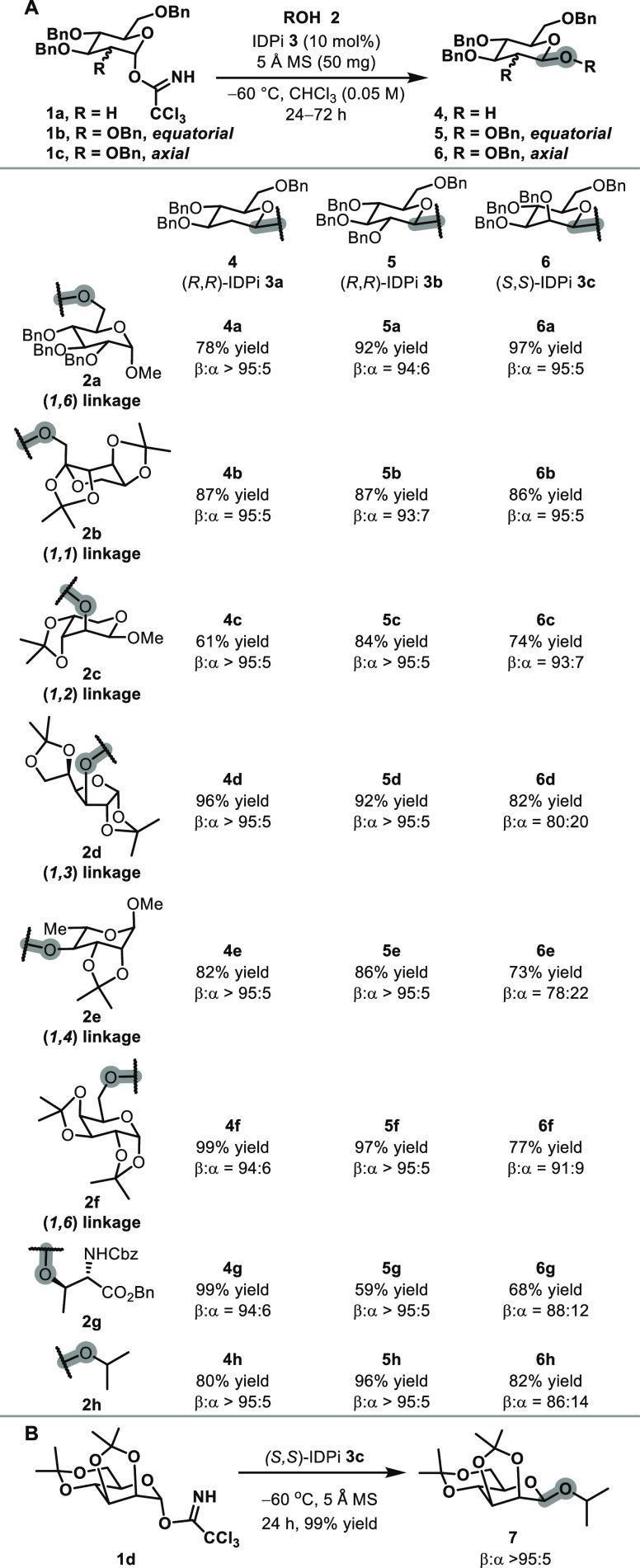
(A) Scope of β-glycosylation. Conditions: the reactions were carried out with glycosyl donor **1** (0.05 mmol), IDPi **3** (10 mol %), 5 Å molecular sieves (50 mg), and glycosyl acceptor **2a** (0.075 mmol) in chloroform (1.0 mL) at −60 °C for 24–72 h. β:α ratios were determined by crude ^1^H NMR analysis. (B) β-Glycosylation with acetonide protecting groups.

To investigate whether our IDPi-catalyzed β-glycosylation proceeds via an S_N_1-like pathway, we synthesized β-glycosyl donor **1e** and subjected it to our optimal conditions (Scheme 2A). This afforded product **5a** with 94:6 selectivity, comparable to the 94:6 selectivity observed when the α-isomer **1b** was used as the starting material, consistent with a convergent S_N_1-like pathway. In addition, a parallel secondary H/D KIE study gave *k*_H_/*k*_D_ = 1.20 ± 0.02, consistent with rehybridization and positive-charge development at C1 through an oxocarbenium ion intermediate along the reaction pathway (Scheme 2B). On the basis of this result, an S_N_1-like mechanism is proposed for the IDPi-catalyzed β-glycosylation (Scheme 2C). First, IDPi **3** activates the trichloroacetimidate on the glycosyl donor **1**, generating trichloroacetamide and the oxocarbenium ion, associated with the IDPi anion. Then, the glycosyl acceptor **2** attacks the opposing β face of the oxocarbenium ion in **TS1**, serving as the selectivity-determining step, producing the β-glycoside and regenerating IDPi **3**.

**Scheme 2:**
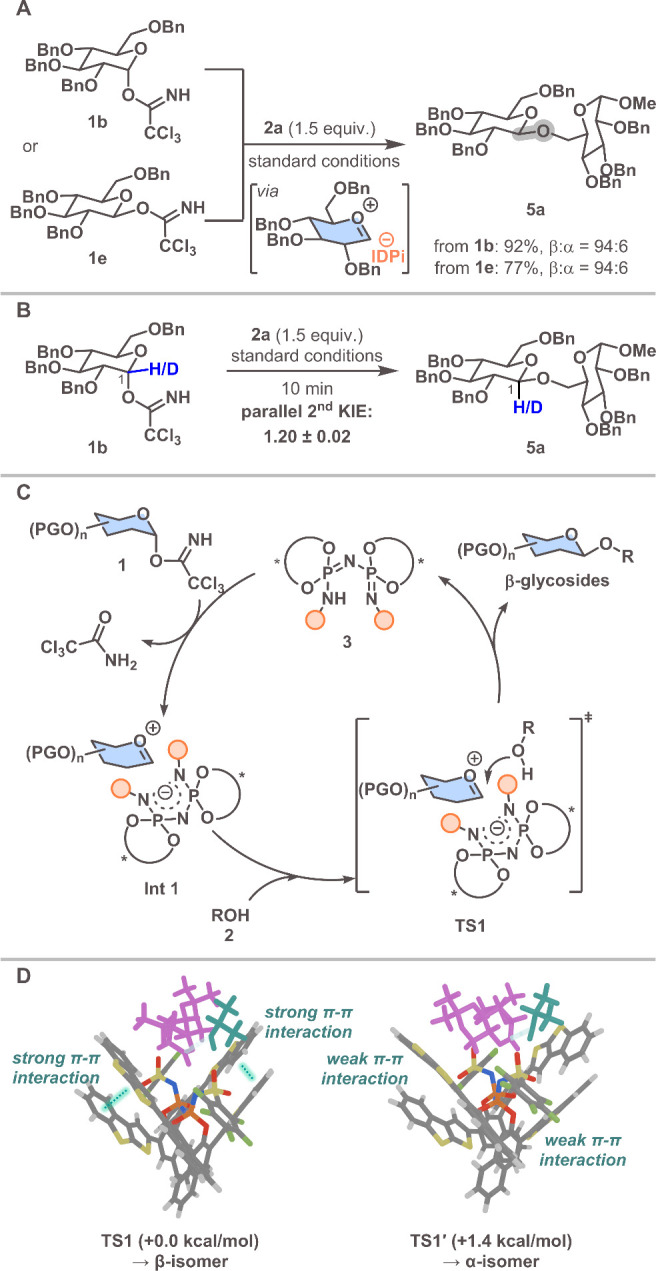
Mechanistic Studies: (A) Control Experiment; (B) Parallel Secondary H/D KIE Study; (C) Proposed Mechanism; (D) Visualization of Transition States Leading to the β-Isomer (**TS1**) and α-Isomer (**TS1′**)­^a^

a In (D), the free energies were obtained at the CPCM (chloroform)-ωB97M-V/(ma)-def2-TZVPP//r^2^SCAN-3c level of theory. The structure shown in pink corresponds to the oxocarbenium ion, while the structure shown in green corresponds to *i*-PrOH.

To elucidate the origin of stereoselectivity, DFT calculations were performed at the CPCM­(chloroform)-ωB97M-V/(ma)-def2-TZVPP//r^2^SCAN-3c level of theory on the reaction of glycosyl donor **1d**, IDPi **3c**, and *i*-PrOH (Scheme 2D).^32–35^ As hypothesized, formation of the *O*-glycosidic bond can proceed through two pathways: the glycosyl acceptor attacks the β face of the oxocarbenium ion **Int 1** via **TS1** to yield the β-isomer, and it attacks the α face via **TS1′** to yield the α-isomer. The energy difference between **TS1** and **TS1′** is 1.4 kcal/mol, corresponding to a 96.5:3.5 β-selectivity. This result is in good agreement with the experimentally determined result (>95:5 β-selectivity). Distortion-interaction analysis of **TS1** and **TS1′** suggests that the energy difference between the two transition states mainly arises from the destabilization of the IDPi anion in **TS1′** (see Supporting Information Table S8). In **TS1**, the oxocarbenium ion adopts a conformation within the catalytic pocket that enables *O*-glycosidic bond formation with *i*-PrOH, while maintaining π–π interactions between the heteroaromatic BINOL substituents, which ultimately leads to the observed energy difference (see Supporting Information Figure S5).

In conclusion, we have developed a general IDPi-catalyzed approach to β-selective glycosylation via an S_N_1-like pathway. Our highly acidic and confined IDPi catalysts may open a new avenue in real-world applications for stereoselective glycan assembly.

## Supplementary Material



## Notes

Experimental details and analytical data for all new compounds, NMR spectra, and computational studies (PDF)
