# Supplementary material for: Confined Acids Catalyze a Broadly Applicable β‑Selective O‑Glycosylation
Source: J Am Chem Soc. 2026 Apr 6;148(16):16594–8. doi: 10.1021/jacs.6c01167 (PMC13133915; doi:10.1021/jacs.6c01167)
Supplement: Supplementary file 1 [file ja6c01167_si_001.pdf]

Supporting Information

Confined Acids Catalyze a Broadly Applicable β-Selective Glycosylation

Jiaxiang Lu,[a‡] Tianyu Zheng,[a‡] Satoshi Matsutani,[b] Nobuya Tsuji,[c] Chendan Zhu,[a] Markus Leutzsch[a] and Benjamin List[a,c*]

[a] Max-Planck-Institut für Kohlenforschung, 45470 Mülheim an der Ruhr, Germany

[b] Graduate School of Chemical Sciences and Engineering, Hokkaido University, Sapporo, Hokkaido 060-8628, Japan

[c] Institute for Chemical Reaction Design and Discovery, Hokkaido University, Sapporo 001-0021, Japan

‡ These authors contributed equally: Jiaxiang Lu, Tianyu Zheng.

*Email: list@kofo.mpg.de

Table of Contents

1. General information........ S2

2. Reaction optimizations........ S3

3. IDPi Catalysts synthesis........ S6

4. Catalytic β-selective glycosylation........ S11

5. Mechanistic studies........ S103

6. References........ S161

1. General information

Nuclear Magnetic Resonance (NMR) Spectroscopy: ^{1}H, ^{13}C, ^{19}F, and ^{31}P NMR spectra were recorded on a Bruker Avance III 500 or Bruker Avance Neo 600 MHz spectrometer in the appropriate deuterated solvent unless otherwise specified. The solvent used and corresponding measurement frequency are indicated for each experiment. For ^{1}H NMR spectra, chemical shifts are reported in ppm downfield from TMS, using either TMS (0.00 ppm) or the residual solvent (e.g., $CDCl_3$ = 7.26 ppm, CD_2Cl_2 = 5.32 ppm) signal as an internal reference. Data are reported as follows: chemical shift (multiplicity: s = singlet, d = doublet, t = triplet, q = quartet, m = multiplet; coupling constants in Hz; integration). All ^{13}C NMR spectra were recorded with proton decoupling unless otherwise noted.

High resolution mass spectrometric (HRMS): HRMS were recorded on a Finnigan MAT 95 (ESI) or a Bruker APEX III FTMS (7 T magnet, ESI). The ionization method and detection mode are specified for each experiment. All masses are reported in m/z units, normalized to the most intense peak.

Specific Rotations ([α]): [α] were measured using a Rudolph RA Autopol IV automatic polarimeter at the specified temperature with a sodium D lamp (λ = 589 nm). Measurements were performed in an acid-resistant 1 mL cell (50 mm path length). Concentrations are reported in g per 100 mL in the indicated solvent.

Unless otherwise indicated, starting materials were determined by Sigma-Aldrich, ABCR-GmbH, TCI, Acros Co. Ltd., Fluorochem, Deutero GmbH, Adamas-beta, Alfa Aesar, J&K, BIOSYNTH or BLD et al. Commercially available substances were used without further purification. Solvents (Toluene, Et_2O, THF, 1,4-dioxane, CH_2Cl_2, $CHCl_3$, MeCN and MTBE) were dried by distillation from appropriate drying agents in the technical department of the Max-Planck-Institut für Kohlenforschung and collected in Schlenk flasks under an argon atmosphere.

2. Reaction optimizations

General Procedure A (0.01 mmol scale). In an oven-dried 2 mL vial equipped with a magnetic stir bar were placed the glycosyl donor (0.01 mmol, 1.0 equiv.), alcohol 2 (0.015 mmol, 1.5 equiv.), and activated 5 Å molecular sieves (10 mg). Anhydrous $CHCl_3$ (80 μL) was added, and the resulting suspension was stirred at −60 °C for 5 min (1000 rpm). In parallel, the IDPi catalyst (10 mol%) was dissolved in anhydrous $CHCl_3$ (20 μL), precooled to −60 °C, and transferred via syringe to the reaction vial. The mixture was stirred at −60 °C. Upon completion, Et_3N (2 μL) was added to quench the reaction, and the mixture was stirred for an additional 5 min at −60 °C. The reaction mixture was filtered through a short pad of Celite and rinsed with $CDCl_3$ (0.6 mL). The filtrate was analyzed directly by ^1H NMR to determine the β/α anomeric ratio. For condition optimization experiments, all reactions proceeded to full conversion.

Table S1. Catalyst class screening for glucose glycosylation

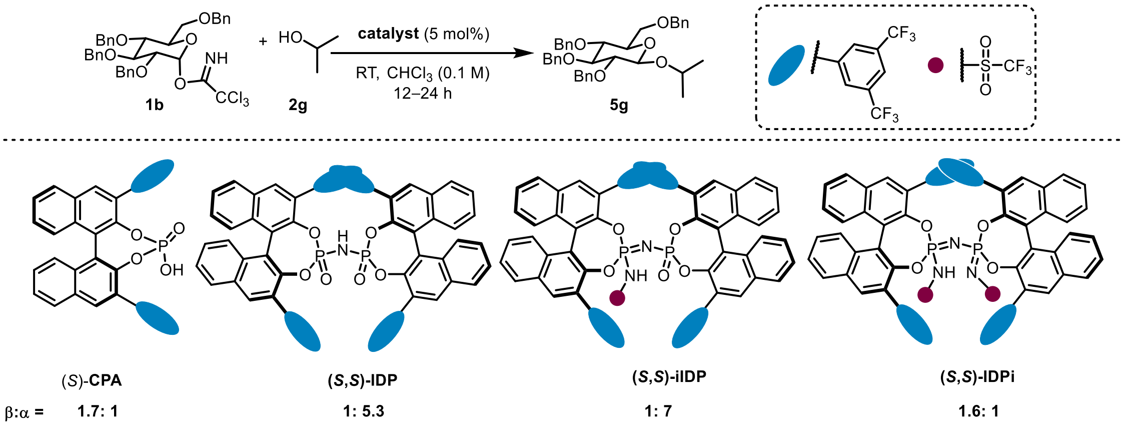

Table S2. Catalyst screening for glucose glycosylation

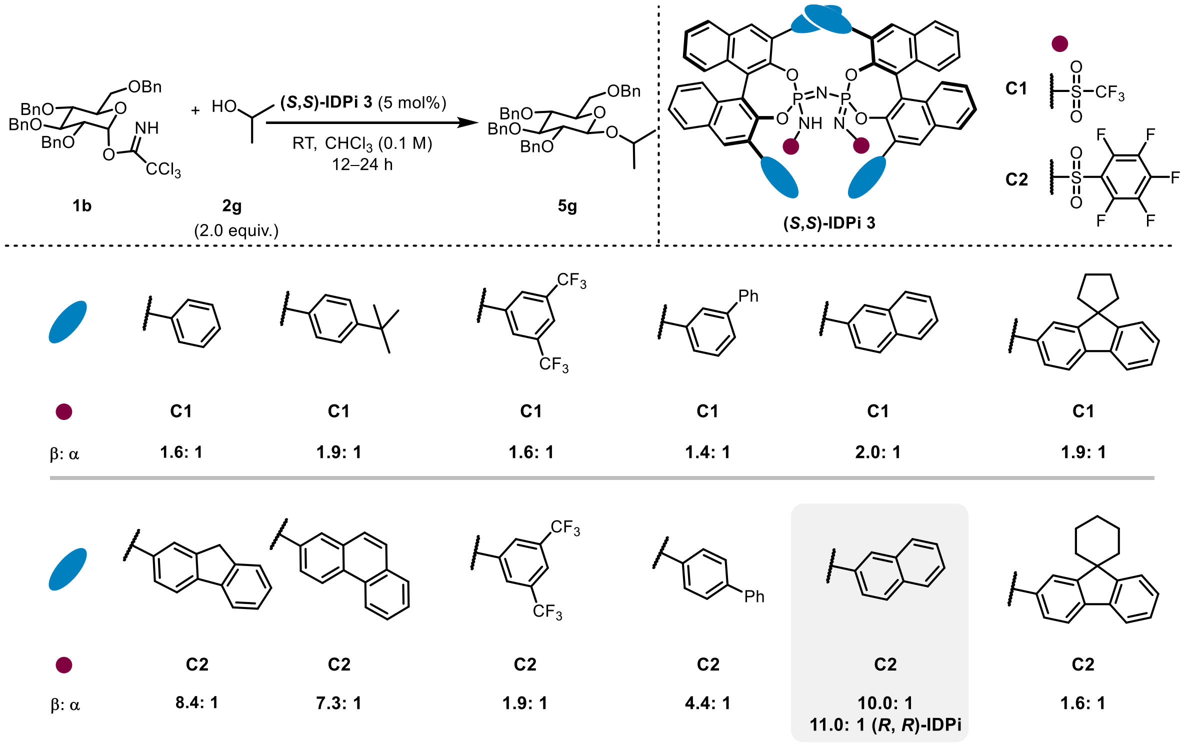

Table S3. Screening of temperature, solvent, and additives for glucose glycosylation

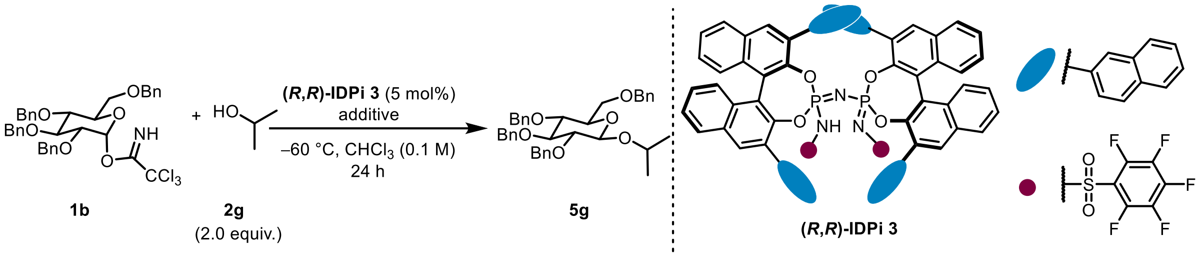

entry	T/°C	solvent	additive	β/α	note
1	-60	Toluene (0.1 M)	---	14:1	
2	-60	CH_2Cl_2 (0.1 M)	---	13:1	
3	-60	Et_2O (0.1 M)	---	12:1	
4	-60	MeCN (0.1 M)	---	---	messy
5	-60	MTBE (0.1 M)	---	6:1	
6	-60	$CHCl_3$ (0.1 M)	---	19:1	
7	-60	$CHCl_3$ (0.2 M)	---	19:1	
8	-60	$CHCl_3$ (0.05 M)	---	20:1	20% hydrolyzed
9	-60	$CHCl_3$ (0.05 M)	3Å MS	---	NR
10	-60	$CHCl_3$ (0.05 M)	4Å MS	---	NR
11	-60	$CHCl_3$ (0.05 M)	5Å MS	20:1	
12	-40	$CHCl_3$ (0.05 M)	5Å MS	14:1	
13	0	$CHCl_3$ (0.05 M)	5Å MS	11.5:1	
**14***	**-60**	**$CHCl_3$ (0.05 M)**	**5Å MS**	**32:1**	

* The reaction was equipped with 10 mol% IDPi

Table S4. Catalyst screening for 2-deoxyglucose glycosylation

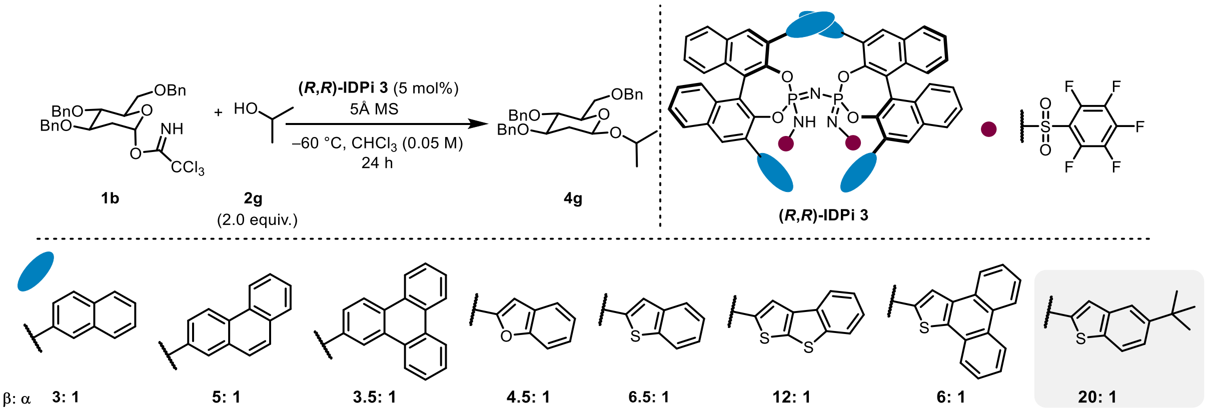

Table S5. Catalyst screening for mannose glycosylation

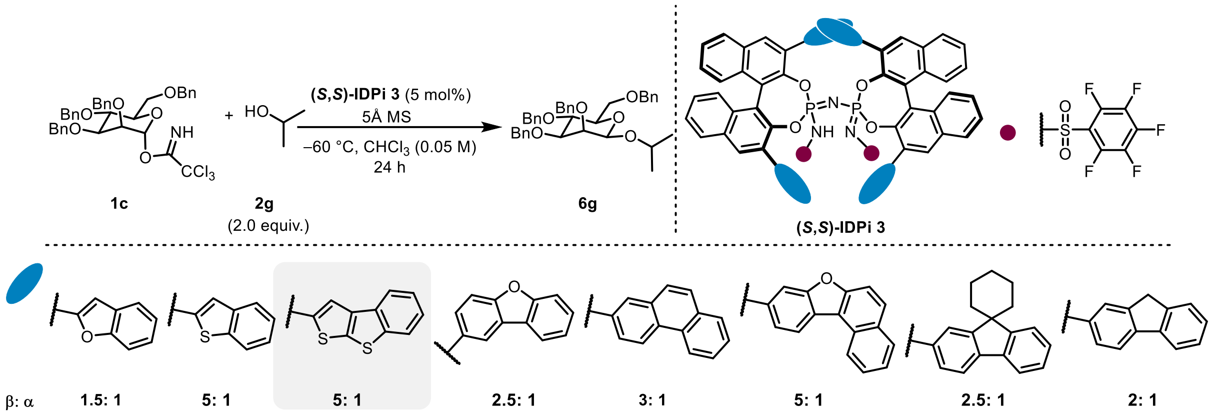

Table S6. Catalyst screening for glycosylation

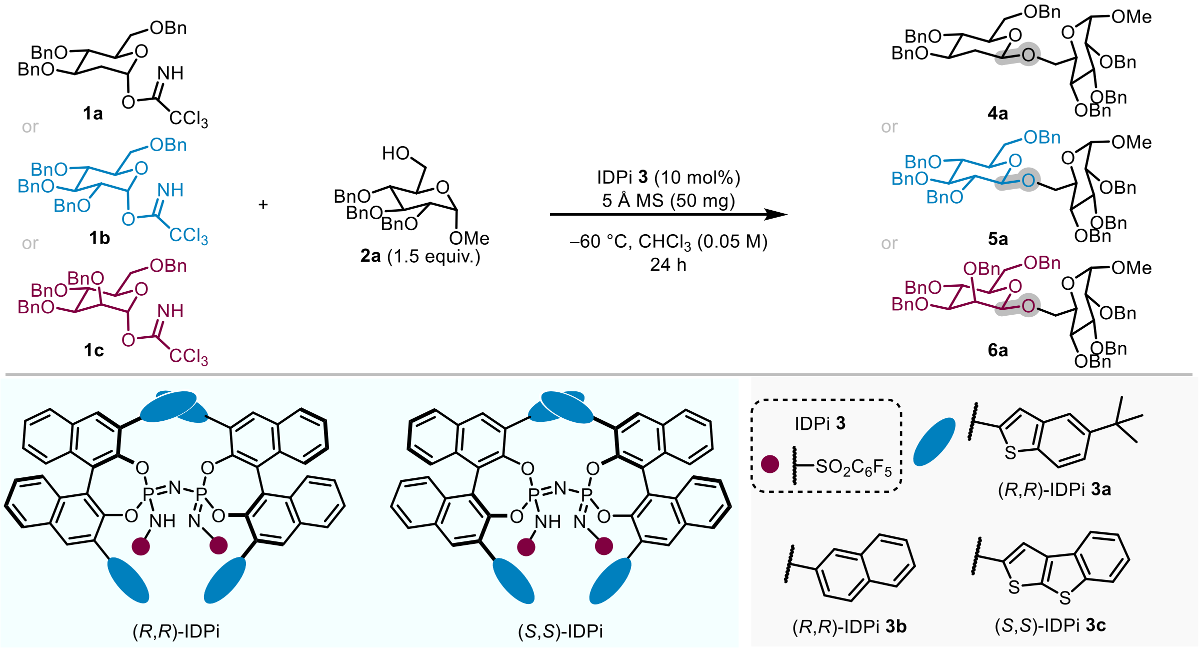

entry	IDPi 3	product	β:α
1		**4a**	>95:5
2	(*R*,*R*)-IDPi **3a**	**5a**	83:17
3		**6a**	29:71
4		**4a**	71:29
5	(*R*,*R*)-IDPi **3b**	**5a**	94:6
6		**6a**	67:33
7		**4a**	50:50
8	(*S*,*S*)-IDPi **3c**	**5a**	80:20
9		**6a**	>95:5

3. IDPi Catalysts synthesis

The catalysts used in the condition optimization studies are known catalysts. The NMR spectra of the (*R,R*)-IDPi catalysts reported herein are identical to those of the corresponding (*S,S*)-IDPi catalysts reported in the literature. (*R,R*)-IDPi **3b**[1] and (*S,S*)-IDPi **3c**[2] were prepared according to reported procedures, and their NMR spectra match those reported. The synthesis of (*R,R*)-IDPi **3a** is described below.

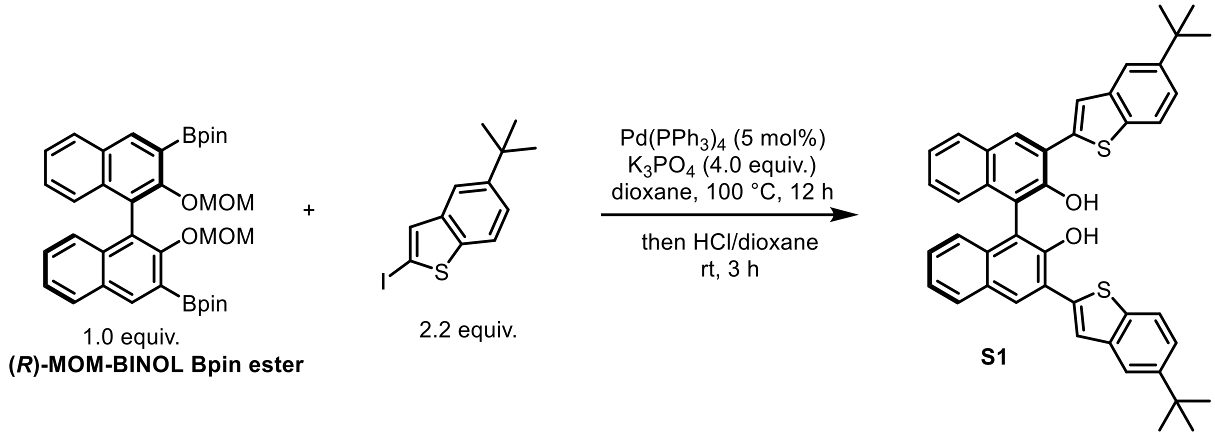

A Schlenk flask was charged with (*R*)-MOM-BINOL Bpin ester (1.0 equiv.), 2- 5-(tert-butyl)-2-iodobenzo[*b*]thiophene (2.2 equiv.), aqueous K_3PO_4 (4.0 equiv.), and 1,4-dioxane. The resulting suspension was sparged with argon for 30 min, $Pd(PPh_3)_4$ (5 mol%) was added under argon, and the mixture was heated at 100 °C overnight. After cooling, saturated aq NH_4Cl was added and the aqueous phase was extracted with DCM (3×). The combined organic layers were washed with water, dried over anhydrous Na_2SO_4, and concentrated under reduced pressure. The crude material was dissolved in HCl (4 M in 1,4-dioxane) and stirred at room temperature overnight. The reaction mixture was diluted with 10% aq HCl and DCM, and the aqueous phase was extracted with DCM (3×). The combined organic layers were washed with water, dried over anhydrous Na_2SO_4, and concentrated under reduced pressure. Purification by flash column chromatography on silica gel (Hexane/EtOAc = 15:1 to 8:1) furnished the title compound **S1** as a yellow solid.

^{1}H NMR (501 MHz, $CDCl_3$) δ 1.41 (s, 9H), 5.71 (s, 1H), 7.14 – 7.24 (m, 1H), 7.31 – 7.38 (m, 1H), 7.40 – 7.48 (m, 2H), 7.77 – 7.87 (m, 2H), 7.97 (d, *J* = 8.6 Hz, 2H), 8.37 (s, 1H).

^{13}C NMR (126 MHz, $CDCl_3$) *δ* 31.7, 34.9, 112.3, 120.2, 121.6, 123.3, 123.8, 124.3, 124.9, 128.1, 128.8, 129.5, 131.0, 132.9, 137.0, 139.2, 140.9, 147.9, 150.2.

HRMS m/z (ESI): calcd. for C44 H37 O2 S2 ($[M-H]^-$): 661.224050; found: 661.224970.

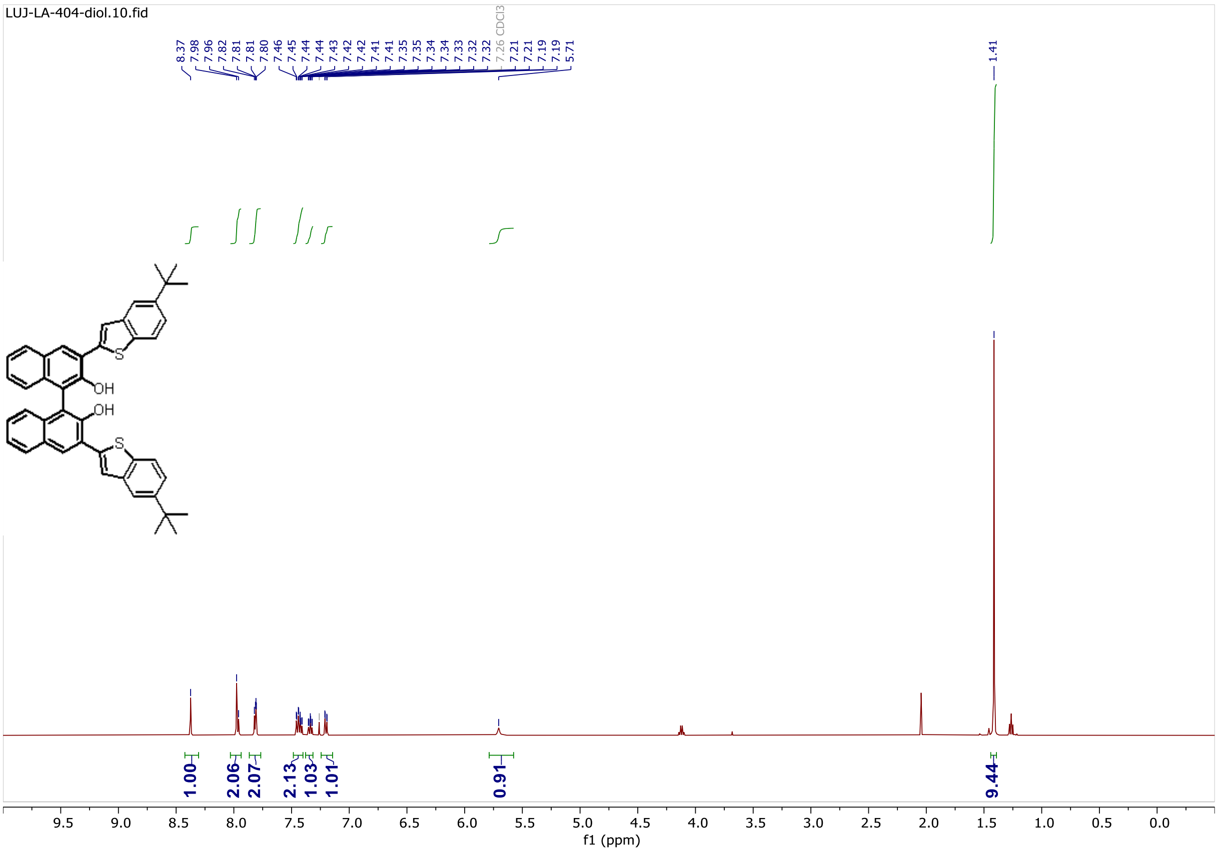
LUJ-LA-404-diol.10.fid
8.37
7.98
7.96
7.82
7.81
7.81
7.80
7.46
7.45
7.44
7.44
7.43
7.42
7.42
7.41
7.41
7.35
7.35
7.34
7.34
7.33
7.32
7.32
7.26 CDCl3
7.21
7.21
7.19
7.19
5.71
1.41
OH
OH
S
S
1.00
2.06
2.07
2.13
1.03
1.01
0.91
9.44
9.5
9.0
8.5
8.0
7.5
7.0
6.5
6.0
5.5
5.0
4.5
4.0
3.5
3.0
2.5
2.0
1.5
1.0
0.5
0.0
f1 (ppm)

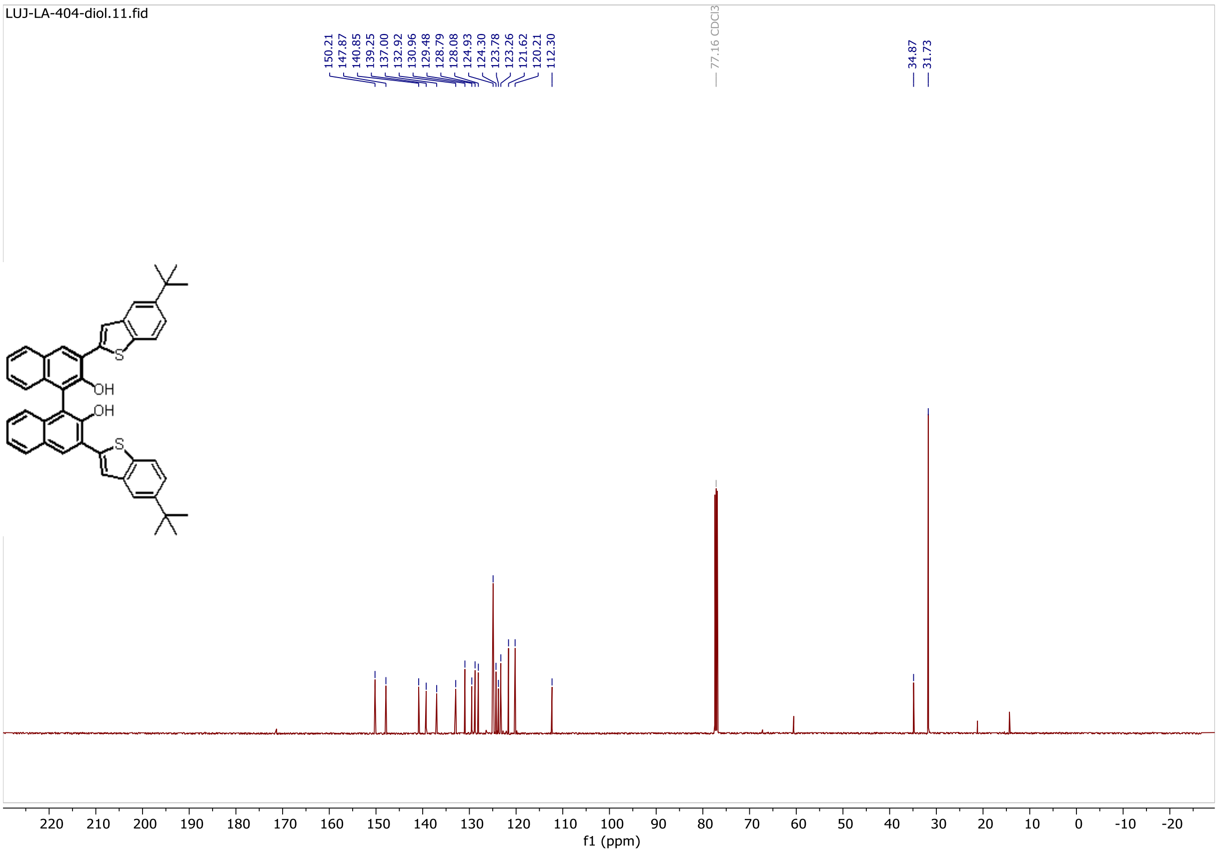
LUJ-LA-404-diol.11.fid
150.21
147.87
140.85
139.25
137.00
132.92
130.96
129.48
128.79
128.08
124.93
124.30
123.78
123.26
121.62
120.21
112.30
77.16 CDCl3
34.87
31.73
OH
OH
S
S
220
210
200
190
180
170
160
150
140
130
120
110
100
90
80
70
60
50
40
30
20
10
0
-10
-20
f1 (ppm)

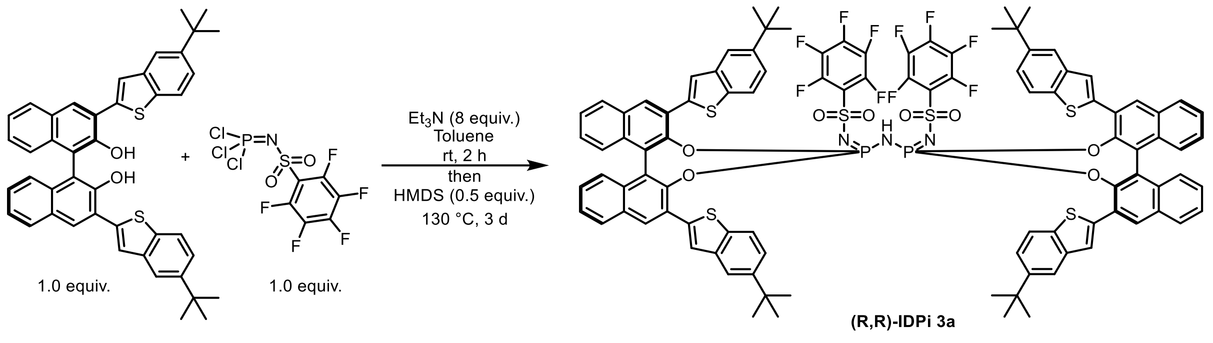

A flame-dried Schlenk flask was charged with (*R*)-BINOL (1.0 equiv.) under argon and dried at 50 °C under high vacuum overnight. After backfilling with argon, $P(NSO_2C_6F_5)Cl_3$ (1.0 equiv.), toluene, and Et_3N (8.0 equiv.) were added, and the reaction mixture was stirred at room temperature for 2 h. HMDS (0.5 equiv.) was then added and the mixture was stirred at room temperature for 15 min, followed by heating at 130 °C for 3 d. After cooling to room temperature, the reaction mixture was diluted with DCM and quenched with 1 M HCl. The aqueous phase was extracted with DCM (3×), and the combined organic layers were washed with 1 M HCl and water, dried over anhydrous Na_2SO_4, and concentrated under reduced pressure. Purification by flash column chromatography on silica gel (Hexane/DCM/EtOAc = 30:10:2 to 30:30:5) afforded the product as a white solid. The product was further acidified by dissolving it in DCM and vigorously stirring with 6 M HCl for 20 min. The organic layer was concentrated under reduced pressure and dried under high vacuum to furnish the title compound as an off-white solid (0.54 mmol scale, 365 mg, 71% yield).

^{1}H NMR (501 MHz, $CDCl_3$) δ 0.74 (s, 18H), 1.26 (s, 18H), 6.46 (s, 2H), 6.68 (s, 1H), 6.72 (dd, *J* = 8.5, 1.8 Hz, 2H), 6.97 (d, *J* = 8.5 Hz, 2H), 7.11 – 7.18 (m, 4H), 7.20 (d, *J* = 8.4 Hz, 2H), 7.22 – 7.26 (m, 2H), 7.29 (dd, *J* = 8.6, 1.9 Hz, 2H), 7.34 (d, *J* = 8.6 Hz, 2H), 7.44 – 7.54 (m, 6H), 7.56 (d, *J* = 8.5 Hz, 2H), 7.70 (t, *J* = 7.5 Hz, 2H), 7.83 – 7.89 (m, 4H), 8.01 (d, *J* = 8.2 Hz, 2H), 8.07 (s, 2H).

^{13}C NMR (126 MHz, $CDCl_3$) *δ* 31.0, 31.6, 34.3, 34.9, 117.1, 119.5, 120.7, 121.1, 121.7, 122.4, 123.2, 123.7, 123.8, 124.7, 125.9, 126.1, 126.8, 127.1, 127.1, 127.3, 127.4, 127.4, 128.5, 128.9, 129.2, 130.0, 131.2, 131.5, 132.27, 132.30, 135.7, 135.9, 136.7, 137.5, 138.0, 139.7, 140.9, 142.5, 143.0, 147.1, 148.0.

^{19}F NMR (471 MHz, $CDCl_3$) δ -159.1 (t, *J* = 20.7 Hz), -145.9, -135.1 (d, *J* = 21.6 Hz).

^{31}P NMR (203 MHz, $CDCl_3$) δ -15.6.

HRMS m/z (ESI): calcd. for C100 H72 F10 N3 O8 P2 S6 ($[M-H]^-$): 1886.29647; found: 1886.29759.

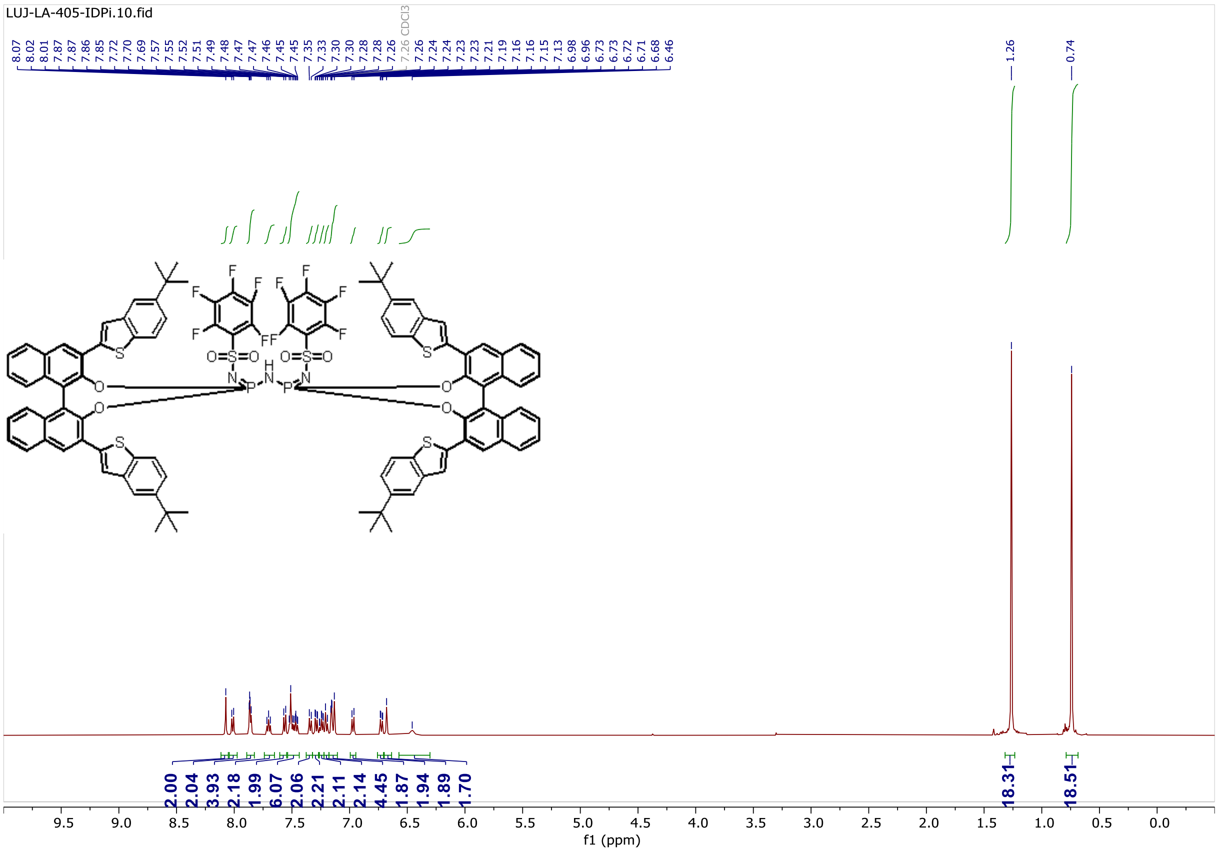

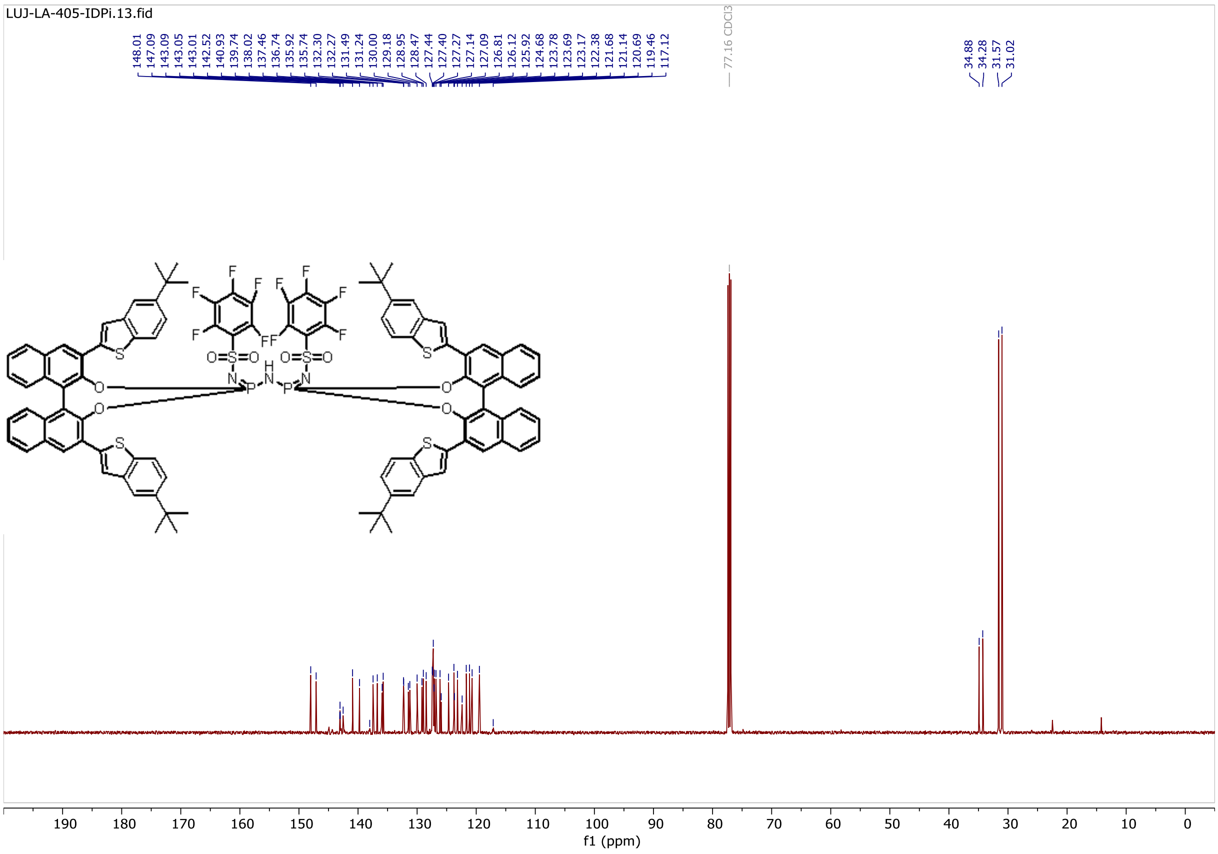

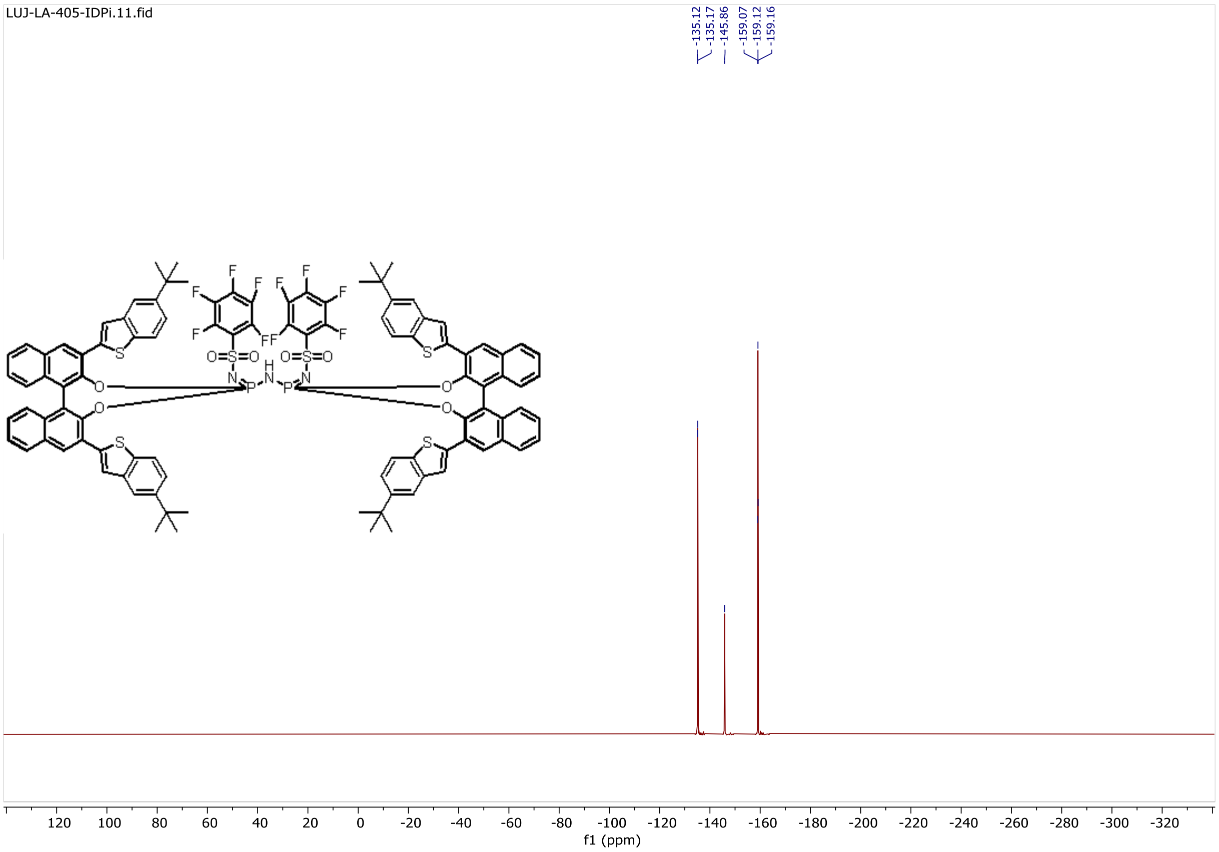
LUJ-LA-405-IDPi.11.fid
-135.12
-135.17
-145.86
-159.07
-159.12
-159.16
120 100 80 60 40 20 0 -20 -40 -60 -80 -100 -120 -140 -160 -180 -200 -220 -240 -260 -280 -300 -320
f1 (ppm)

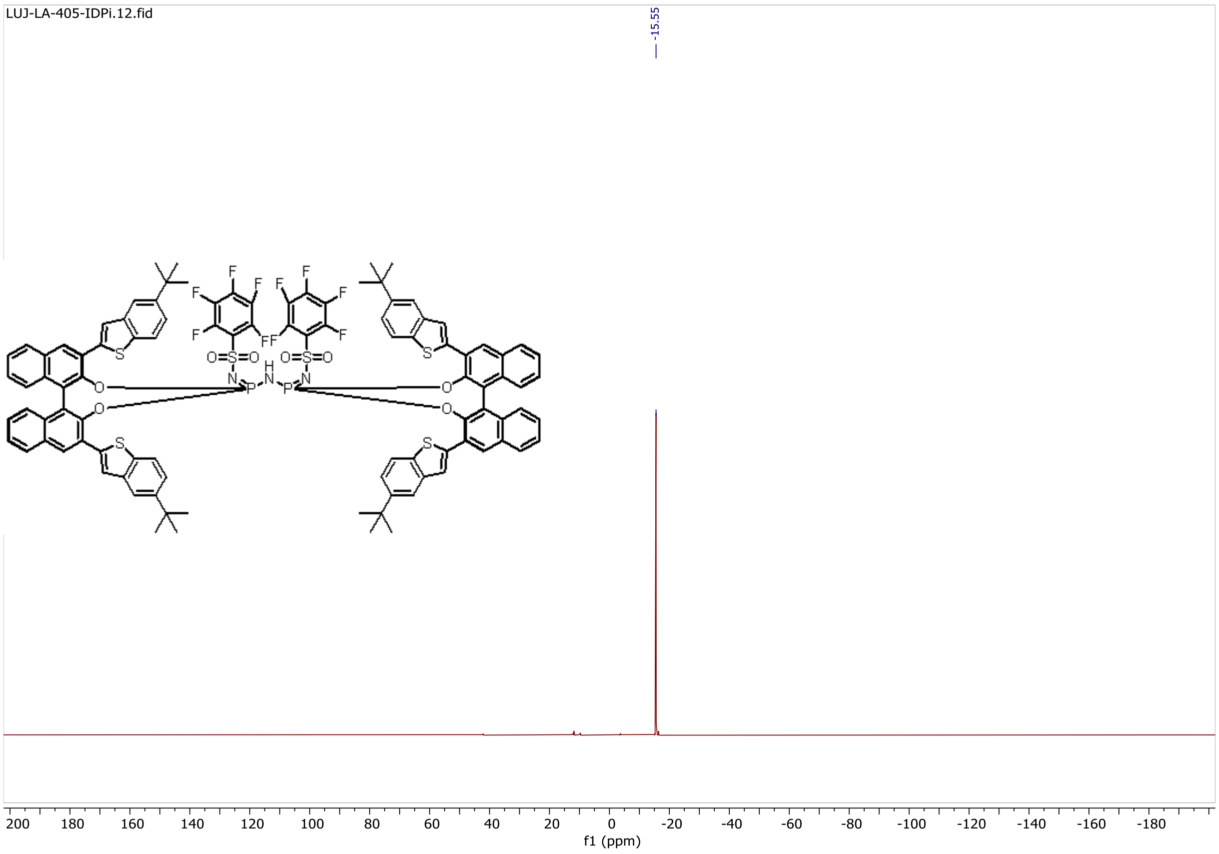
LUJ-LA-405-IDPi.12.fid
-15.55
200 180 160 140 120 100 80 60 40 20 0 -20 -40 -60 -80 -100 -120 -140 -160 -180
f1 (ppm)

4. Catalytic β-selective glycosylation

Table S7. Substrate scope for glycosylation

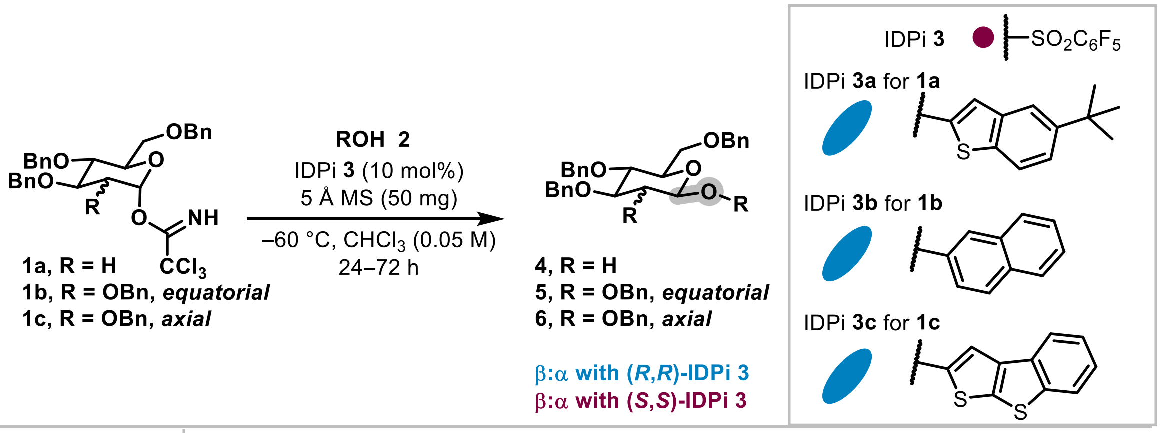

	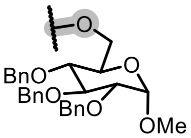 **2a**	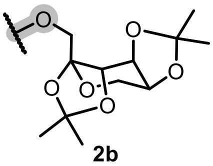 **2b**	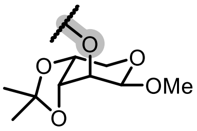 **2c**	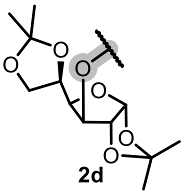 **2d**
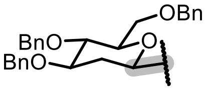 **4**	**4a**, 78%<br>β:α > 95:5<br>β:α = 69:31	**4b**, 87%<br>β:α = 95:5<br>β:α = 60:40	**4c**, 61%<br>β:α > 95:5<br>β:α = 80:20	**4d**, 96%<br>β:α > 95:5<br>β:α >95:5
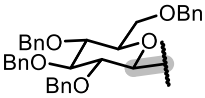 **5**	**5a**, 92%<br>β:α = 94:6<br>β:α > 95:5	**5b**, 87%<br>β:α = 93:7<br>β:α > 95:5	**5c**, 84%<br>β:α > 95:5<br>β:α > 95:5	**5d**, 92%<br>β:α > 95:5<br>β:α > 95:5
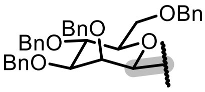 **6**	**6a**, 97%<br>β:α = 83:17<br>β:α = 95:5	**6b**, 86%<br>β:α = 76:24<br>β:α = 95:5	**6c**, 74%<br>β:α = 81:19<br>β:α = 93:7	**6d**, 82%<br>β:α = 80:20<br>β:α = 80:20
	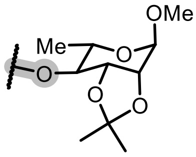 **2e**	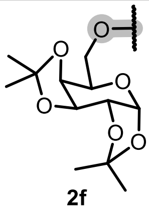 **2f**	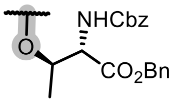 **2g**	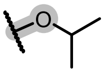 **2h**
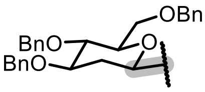 **4**	**4e**, 82%<br>β:α > 95:5<br>β:α = 85:15	**4f**, 99%<br>β:α = 94:6<br>β:α = 71:29	**4g**, 99%<br>β:α = 94:6<br>β:α = 92:8	**4h**, 80%<br>β:α > 95:5<br>β:α = 88:12
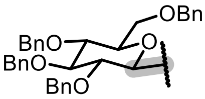 **5**	**5e**, 86%<br>β:α > 95:5<br>β:α > 95:5	**5f**, 97%<br>β:α > 95:5<br>β:α > 95:5	**5g**, 59%<br>β:α > 95:5<br>β:α = 82:18	**5h**, 96%<br>β:α > 95:5<br>β:α > 95:5
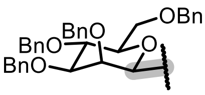 **6**	**6e**, 73%<br>β:α = 64:36<br>β:α = 78:22	**6f**, 77%<br>β:α = 78:22<br>β:α = 91:9	**6g**, 68%<br>β:α = 77:23<br>β:α = 88:12	**6h**, 82%<br>β:α = 69:31<br>β:α = 86:14

General Procedure B:

In an oven-dried 2 mL vial equipped with a magnetic stir bar were placed the glycosyl donor (0.05 mmol, 1.0 equiv.), alcohol 2 (0.075 mmol, 1.5 equiv.), and activated 5 Å molecular sieves (50 mg). Anhydrous $CHCl_3$ (700 μL) was added, and the resulting suspension was stirred at −60 °C for 5 min (1000 rpm). In parallel, the IDPi catalyst (10 mol%) was dissolved in anhydrous $CHCl_3$ (300 μL), precooled to −60 °C, and transferred via syringe to the reaction vial. The mixture was stirred at −60 °C until completion (typically 24 h for primary alcohols and 72 h for secondary alcohols. (**2a**: CAS 53008-65-4; **2b**: CAS 20880-92-6; **2c**: CAS 4594-60-9; **2d**: CAS 582-52-5; **2e**: CAS 14133-63-2; **2f**: CAS 4064-06-6). Upon completion, Et_3N (10 μL) was added to quench the reaction, and the mixture was stirred for an additional 5 min at −60 °C. The reaction mixture was filtered through a 2 cm pad of Celite and rinsed with $CDCl_3$ (3 mL). The filtrate was analyzed directly by ^1H NMR to determine the β/α anomeric ratio.

For isolation, the filtrate was diluted with CH_2Cl_2 (10 mL) and washed with 10 wt% aqueous NaOH (10 mL)[3]. The aqueous layer was extracted with CH_2Cl_2 (2 × 10 mL). The combined organic layers were washed with brine, dried over anhydrous Na_2SO_4, filtered, and concentrated under reduced pressure. The residue was preadsorbed onto 2-gram silica gel and purified by flash column chromatography (hexane/EtOAc as eluent) to afford the desired product.

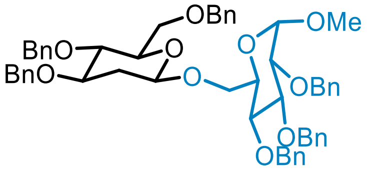

(2*R*,3*R*,4*S*,5*R*,6*S*)-3,4,5-tris(benzyloxy)-2-((((2*R*,4*R*,5*S*,6*R*)-4,5-bis(benzyloxy)-6-((benzyloxy)methyl)tetrahydro-2*H*-pyran-2-yl)oxy)methyl)-6-methoxytetrahydro-2*H*-pyran (4a)

Following **General Procedure B**, glycosyl donor **1a** (29 mg, 0.050 mmol) and alcohol **2a** (35 mg, 0.075 mmol) were coupled using (*R*,*R*)-IDPi **3a** (9.4 mg,10 mol%). Reaction time: 24 h. β/α >95:5 was determined by crude ^{1}H NMR. Flash chromatography (hexane/EtOAc = 6/1 to 4/1) gave **4a** as white solid (34 mg, 78%). The NMR data of this compound are consistent with those reported in the literature.[4]

Following **General Procedure A** on 0.01 mmol scale, the glycosylation was carried out using (*S*,*S*)-IDPi **3a**. β/α = 69:31 was determined by crude ^{1}H NMR.

TLC: R_f = 0.26 (Hexane/EtOAc = 3:1)

^{1}H NMR (501 MHz, $CDCl_3$) δ 1.56 (td, *J* = 12.1, 9.7 Hz, 1H), 2.09 (ddd, *J* = 12.4, 5.1, 1.9 Hz, 1H), 3.29 (s, 4H), 3.36 (t, *J* = 9.1 Hz, 1H), 3.43 – 3.55 (m, 4H), 3.60 (dd, *J* = 10.8, 5.2 Hz, 1H), 3.63 – 3.70 (m, 2H), 3.93 (t, *J* = 9.2 Hz, 1H), 4.01 (dd, *J* = 10.8, 2.1 Hz, 1H), 4.10 (dd, *J* = 9.8, 1.9 Hz, 1H), 4.43 – 4.55 (m, 6H), 4.59 (d, *J* = 11.8 Hz, 2H), 4.69 – 4.77 (m, 2H), 4.80 (dd, *J* = 11.1, 2.2 Hz, 2H), 4.92 (d, *J* = 10.9 Hz, 1H), 7.08 – 7.15 (m, 2H), 7.16 – 7.32 (m, 26H).

^{13}C NMR (126 MHz, $CDCl_3$) *δ* 36.6, 55.3, 67.7, 69.5, 69.7, 71.5, 73.5, 73.5, 74.9, 75.9, 77.5, 98.1, 100.1, 127.6, 127.7, 127.8, 128.0, 128.1, 128.1, 128.2, 128.3, 128.4, 128.5, 128.5, 128.6, 128.6, 138.2, 138.4, 138.4, 138.4, 138.6, 138.9.

$[\alpha]_D^{22}$ = +11.0 ($CHCl_3$, *c* = 1.0).

HRMS m/z (ESI): calcd. for $C_{55}H_{60}NaO_{10}$ ($[M+Na]^+$): 903.40787; found: 903.40756.

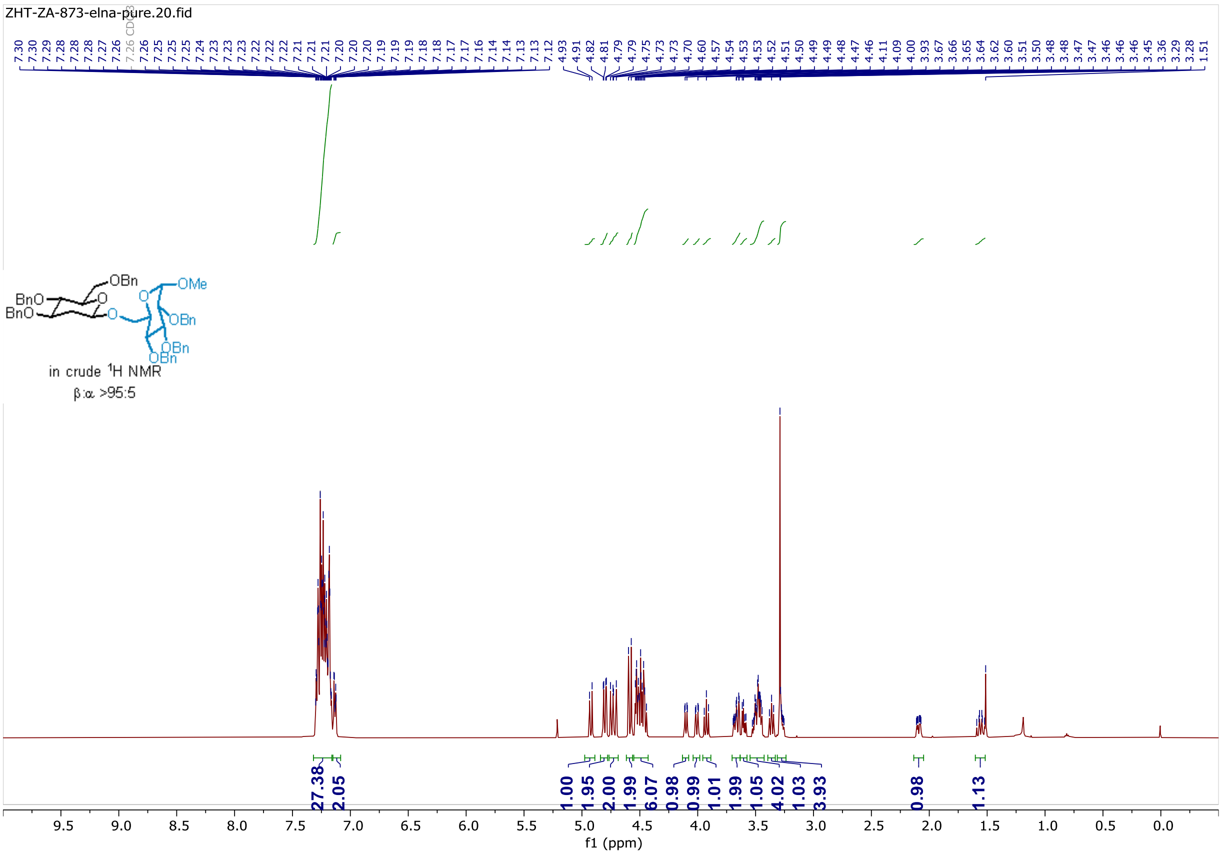

ZHT-ZA-873-elna-pure.20.fid
7.30 7.30 7.29 7.28 7.28 7.28 7.27 7.26 7.26 CDCl3 7.26 7.25 7.25 7.25 7.24 7.23 7.23 7.23 7.22 7.22 7.22 7.21 7.21 7.21 7.20 7.20 7.20 7.19 7.19 7.19 7.19 7.18 7.18 7.17 7.17 7.16 7.14 7.13 7.13 7.12
4.93 4.91 4.82 4.81 4.79 4.79 4.75 4.73 4.70 4.60 4.57 4.54 4.53 4.53 4.52 4.51 4.50 4.49 4.49 4.48 4.47 4.46 4.11 4.09 4.00 3.93 3.67 3.66 3.65 3.64 3.62 3.60 3.51 3.50 3.48 3.47 3.46 3.46 3.45 3.36 3.29 3.28 1.51
OBn
BnO
BnO
O
O
O
OMe
OBn
OBn
OBn
in crude ^{1}H NMR
β:α >95:5
27.38 2.05 1.00 1.95 2.00 1.99 6.07 0.98 0.99 1.01 1.99 1.05 4.02 1.03 3.93 0.98 1.13
9.5 9.0 8.5 8.0 7.5 7.0 6.5 6.0 5.5 5.0 4.5 4.0 3.5 3.0 2.5 2.0 1.5 1.0 0.5 0.0
f1 (ppm)

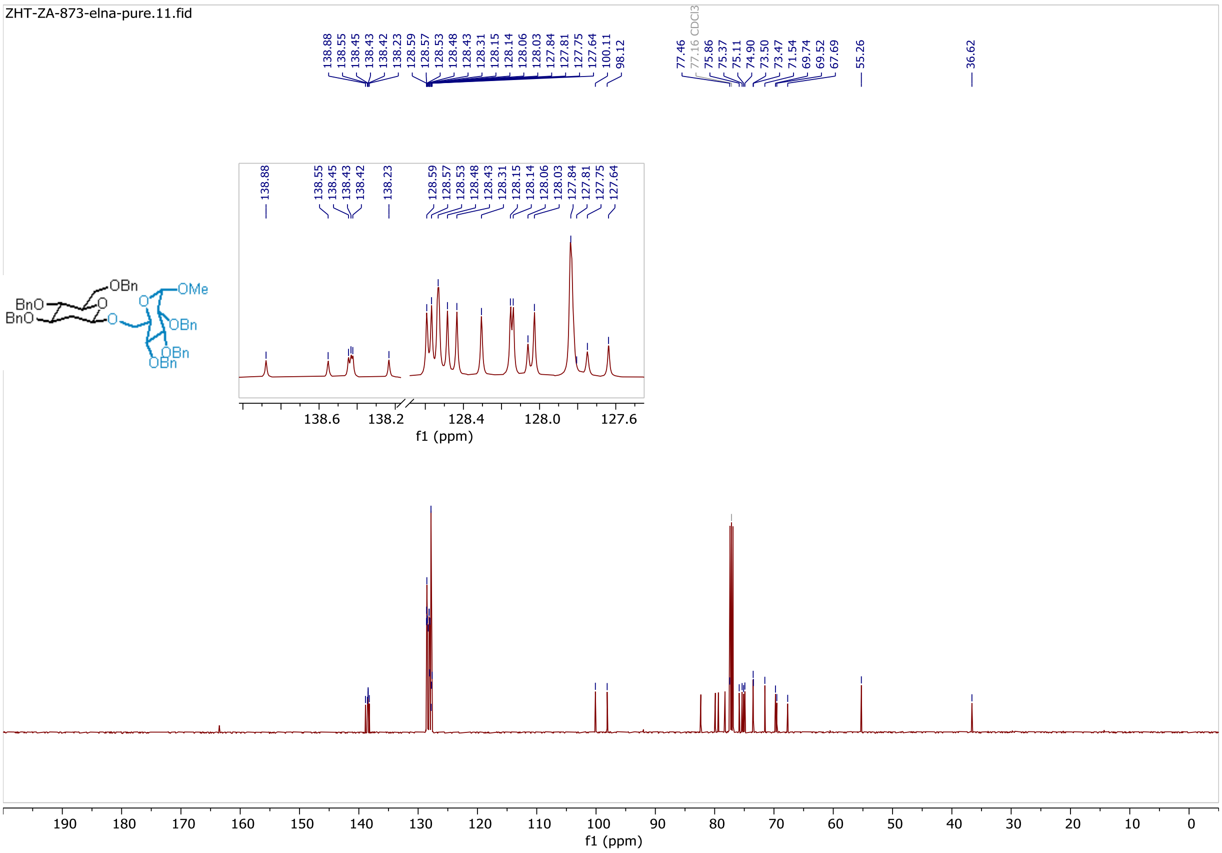

ZHT-ZA-873-elna-pure.11.fid
138.88 138.55 138.45 138.43 138.42 138.23 128.59 128.57 128.53 128.48 128.43 128.31 128.15 128.14 128.06 128.03 127.84 127.81 127.75 127.64 100.11 98.12
77.46 77.16 CDCl3 75.86 75.37 75.11 74.90 73.50 73.47 71.54 69.74 69.52 67.69 55.26 36.62
138.6 138.2 128.4 128.0 127.6
f1 (ppm)
OBn
BnO
BnO
O
O
O
OMe
OBn
OBn
OBn
190 180 170 160 150 140 130 120 110 100 90 80 70 60 50 40 30 20 10 0
f1 (ppm)

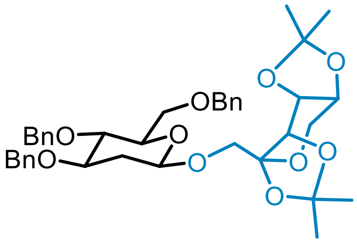

(3a*R*,5a*S*,8a*S*,8b*R*)-3a-((((2*R*,4*R*,5*S*,6*R*)-4,5-bis(benzyloxy)-6-((benzyloxy)methyl)tetrahydro-2*H*-pyran-2-yl)oxy)methyl)-2,2,7,7-tetramethyltetrahydro-5*H*-bis([1,3]dioxolo)[4,5-b:4',5'-d]pyran (4b)

Following **General Procedure B**, glycosyl donor **1a** (29 mg, 0.050 mmol) and alcohol **2b** (20 mg, 0.075 mmol) were coupled using (*R*,*R*)-IDPi **3a** (9.4 mg,10 mol%). Reaction time: 24 h. β/α = 95:5 was determined by crude ^{1}H NMR. Flash chromatography (hexane/EtOAc = 6/1 to 3/1) gave **4b** as white solid (30 mg, 87%). The NMR data of this compound are differed from the α-isomer with those reported in the literature.[5]

Following **General Procedure A** on 0.01 mmol scale, the glycosylation was carried out using (*S*,*S*)-IDPi **3a**. β/α = 60:40 was determined by crude ^{1}H NMR.

TLC: R_f = 0.31 (Hexane/EtOAc = 3:1)

^{1}H NMR (501 MHz, $CDCl_3$) δ 1.25 (s, 3H), 1.31 (s, 3H), 1.36 (s, 3H), 1.47 (s, 3H), 2.23 – 2.32 (m, 1H), 3.29 – 3.36 (m, 1H), 3.49 (t, *J* = 9.1 Hz, 1H), 3.54 – 3.60 (m, 1H), 3.61 – 3.72 (m, 4H), 3.81 – 3.89 (m, 2H), 4.15 (dd, *J* = 7.8, 1.6 Hz, 1H), 4.35 (d, *J* = 2.6 Hz, 1H), 4.45 – 4.62 (m, 7H), 4.82 (d, *J* = 10.9 Hz, 1H), 7.10 – 7.16 (m, 2H), 7.17 – 7.28 (m, 13H).

^{13}C NMR (126 MHz, $CDCl_3$) *δ* 24.19, 25.63, 26.02, 26.73, 36.65, 61.25, 69.21, 69.78, 70.12, 70.27, 71.11, 71.77, 73.69, 75.06, 75.36, 78.19, 79.45, 100.47, 102.60, 108.66, 109.10, 127.66, 127.79, 127.82, 127.85, 127.90, 128.11, 128.46, 128.48, 128.50, 128.55, 138.47, 138.54.

$[\alpha]_D^{22}$ = -9.8 ($CHCl_3$, *c* = 1.0).

HRMS m/z (ESI): calcd. for C39 H48 Na1 O10 ($[M+Na]^+$): 699.31397; found: 699.31409.

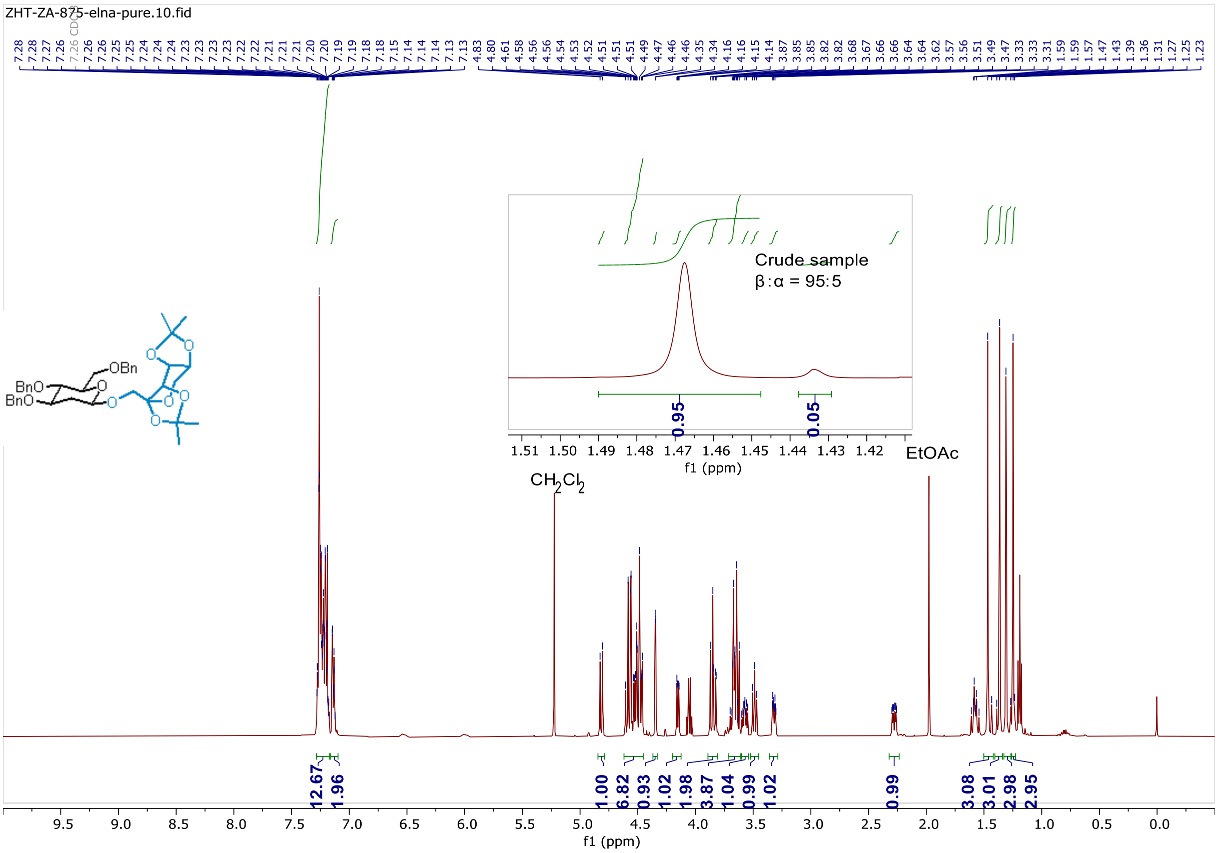
ZHT-ZA-875-elna-pure.10.fid
Crude sample
β:α = 95:5
CH2Cl2
EtOAc
f1 (ppm)

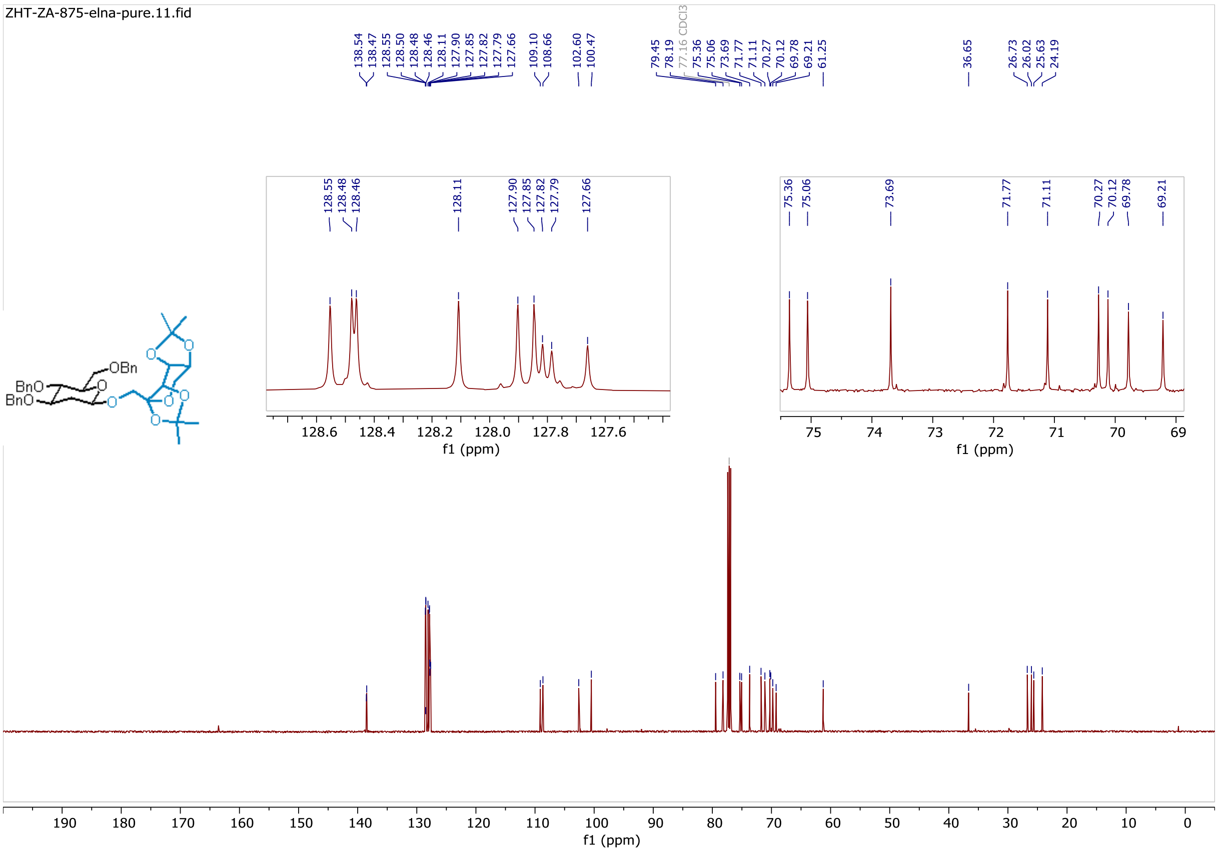
ZHT-ZA-875-elna-pure.11.fid
f1 (ppm)

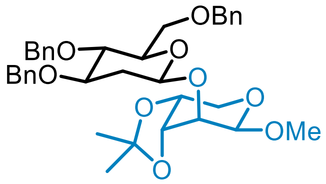

(3a*R*,6*R*,7*S*,7a*R*)-7-(((2*S*,4*R*,5*S*,6*R*)-4,5-bis(benzyloxy)-6-((benzyloxy)methyl)tetrahydro-2*H*-pyran-2-yl)oxy)-6-methoxy-2,2-dimethyltetrahydro-4*H*-[1,3]dioxolo[4,5-c]pyran (4c)

Following **General Procedure B**, glycosyl donor **1a** (29 mg, 0.050 mmol) and alcohol **2c** (15 mg, 0.075 mmol) were coupled using (*R*,*R*)-IDPi **3a** (9.4 mg,10 mol%). Reaction time: 72 h. β/α = 98:2 was determined by crude ^{1}H NMR. Flash chromatography (hexane/EtOAc = 6/1 to 2/1) gave **4c** as colorless oil (19 mg, 61%).

Following **General Procedure A** on 0.01 mmol scale, the glycosylation was carried out using (*S*,*S*)-IDPi **3a**. β/α = 80:20 was determined by crude ^{1}H NMR.

TLC: R_f = 0.15 (Hexane/EtOAc = 3:1)

^{1}H NMR (501 MHz, CDCl$_3$) δ 1.35 (s, 3H), 1.54 (s, 3H), 1.77 (td, *J* = 12.0, 9.8 Hz, 1H), 2.38 (ddd, *J* = 12.5, 5.1, 1.9 Hz, 1H), 3.39 (dt, *J* = 9.6, 3.4 Hz, 1H), 3.42 (s, 3H), 3.52 (dd, *J* = 9.6, 8.6 Hz, 1H), 3.66 (ddd, *J* = 11.5, 8.6, 5.1 Hz, 1H), 3.62 – 3.74 (m, 3H), 3.92 (d, *J* = 2.0 Hz, 2H), 4.03 (dd, *J* = 7.5, 3.1 Hz, 1H), 4.23 (dt, *J* = 5.7, 2.0 Hz, 1H), 4.30 (t, *J* = 7.5 Hz, 1H), 4.50 (d, *J* = 12.2 Hz, 1H), 4.56 (d, *J* = 12.1 Hz, 1H), 4.56 (d, *J* = 10.9 Hz, 1H), 4.47 – 4.65 (m, 5H), 4.69 (d, *J* = 11.8 Hz, 1H), 4.78 (d, *J* = 3.1 Hz, 1H), 4.89 (d, *J* = 10.9 Hz, 1H), 7.17 – 7.23 (m, 2H), 7.23 – 7.38 (m, 13H).

^{13}C NMR (126 MHz, CDCl$_3$) δ 138.51, 138.49, 138.40, 128.56, 128.49, 128.46, 128.21, 127.94, 127.81, 127.78, 127.77, 127.67, 109.18, 97.98, 97.71, 79.60, 78.03, 75.52, 75.10, 74.30, 74.06, 73.58, 73.46, 71.39, 69.61, 59.51, 55.85, 36.51, 28.08, 26.31.

$[\alpha]_D^{22}$ = -52.4 (CHCl$_3$, *c* = 1.0).

HRMS m/z (ESI): calcd. For C36 H44 Na1 O9 ([M+Na]$^+$): 643.28775; found: 643.28765.

User Report
LUJ-LA-398-01

The following compound was assigned in the sample

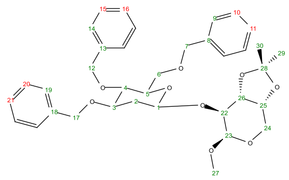

Remarks:
The relative stereochemistry at the anomeric center is supported by NOE correlations from H1 to the axial positioned protons H3 and H5. The couplings of H1 to its neighbours H2ax (9.8 Hz -> trans) & H2eq (1.9 Hz -> gauche) are also typical for a proton in an axial position of a 6-membered ring in a chair conformation.

1H{},NOESY, 500.81 MHz,CDCl3,298.0K, pulse sequence: noesygpphpp

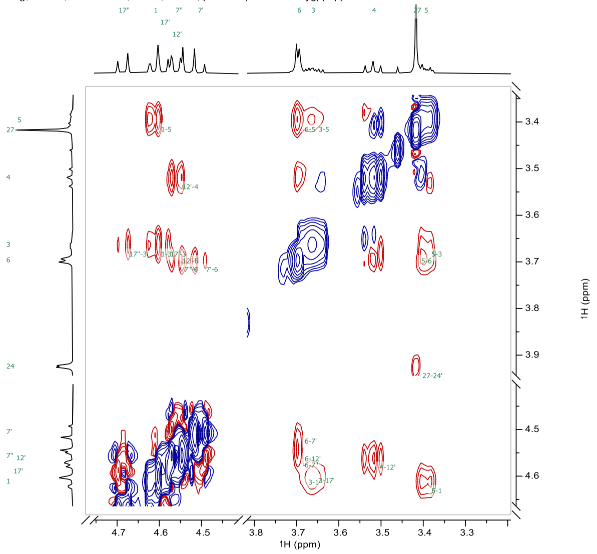

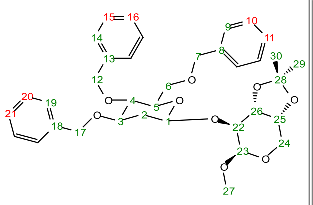

Atom	δ (ppm)	J	COSY	HSQC	HMBC	NOESY
1 C	97.979			1', 1''	2ax, 2eq, 5, 22	
H	4.611	9.80(2ax), 1.90(2eq)	2ax	1	2, 3, 22	2eq, 3, 5, 22
2 C	36.515			2ax, 2eq	1, 3, 4	
Hax	1.768	9.80(1), 11.50(3), 12.50(2eq)	1, 2eq, 3	2	1, 3, 4	2eq, 4
Heq	2.381	5.10(3), 12.50(2ax), 1.90(1)	2ax, 3	2	1, 3, 4	1, 2ax, 3, 17', 17''
3 C	79.603			3	1, 2ax, 2eq, 4, 5, 17', 17''	
H	3.663	8.80(4), 11.50(2ax), 5.10(2eq)	2ax, 2eq, 4	3	2, 4, 5, 17	1, 2eq, 5, 17', 17''
4 C	78.025			4	2ax, 2eq, 3, 5, 6, 12', 12''	
H	3.519	8.80(3), 9.50(5)	3, 5	4	2, 3, 5, 6, 12	2ax, 12', 12''
5 C	75.521			5	3, 4, 6	
H	3.396	9.50(4), 3.40(6)	4, 6	5	1, 3, 4, 6	1, 3, 6
6 C	69.609			6	4, 5, 7', 7''	
H2	3.696	3.40(5)	5	6	4, 5, 7	5, 7', 7'', 9, 12', 14
7 C	73.572			7', 7''	6, 9	
H'	4.505	12.20(7'')	7''	7	6, 8	6, 9
H''	4.556	12.20(7')	7'	7	6, 8	6, 9
8 C	138.396				7', 7''	
9 C						
H	7.320				7	6, 7', 7''
10 C						
H						

Atom	δ (ppm)	J	COSY	HSQC	HMBC	NOESY
11 C						
H						
12 C	75.103			12', 12''	4, 14	
H'	4.561	10.90(12'')	12''	12	4, 13	4, 6, 14
H''	4.890	10.90(12')	12'	12	4, 13	4, 14
13 C	138.513				12', 12''	
14 C	128.210					
H	7.202				12	6, 12', 12''
15 C						
H						
16 C						
H						
17 C	71.386			17', 17''	3, 19	
H'	4.589	11.80(17'')	17''	17	3, 18	2eq, 3, 19
H''	4.687	11.80(17')	17'	17	3, 18	2eq, 3, 19
18 C	138.493				17', 17''	
19 C						
H	7.330				17	17', 17''
20 C						
H						
21 C						
H						

Atom	δ (ppm)	J	COSY	HSQC	HMBC	NOESY
22 C	74.299			22	1, 23, 25, 26	
H	4.025	3.10(23), 7.50(26)	23, 26	22	1, 26	1, 23, 29
23 C	97.709			23	24', 24'', 27	
H	4.776	3.10(22)	22	23	22, 24, 26, 27	22, 27
24 C	59.505			24', 24''	23, 25	
H'	3.924	2.00(25)	25	24	23, 25	25, 27
H''	3.924	2.00(25)	25	24	23, 25, 26	25
25 C	73.464			25	24', 24'', 26	
H	4.231	2.00(24'), 2.00(24''), 5.70(26)	24', 24'', 26	25	22, 24, 26	24', 24'', 26, 30
26 C	74.058			26	22, 23, 24'', 25	
H	4.295	5.70(25), 7.50(22)	22, 25	26	22, 25, 28	25
27 C	55.850			27	23	
H3	3.417			27	23	23, 24'
28 C	109.180				26, 29, 30	
29 C	28.078			29	30	
H3	1.536		30	29	28, 30	22
30 C	26.314			30	29	
H3	1.347		29	30	28, 29	25

1H{},1D, 500.81 MHz,CDCl3,298.0K, pulse sequence: zg30

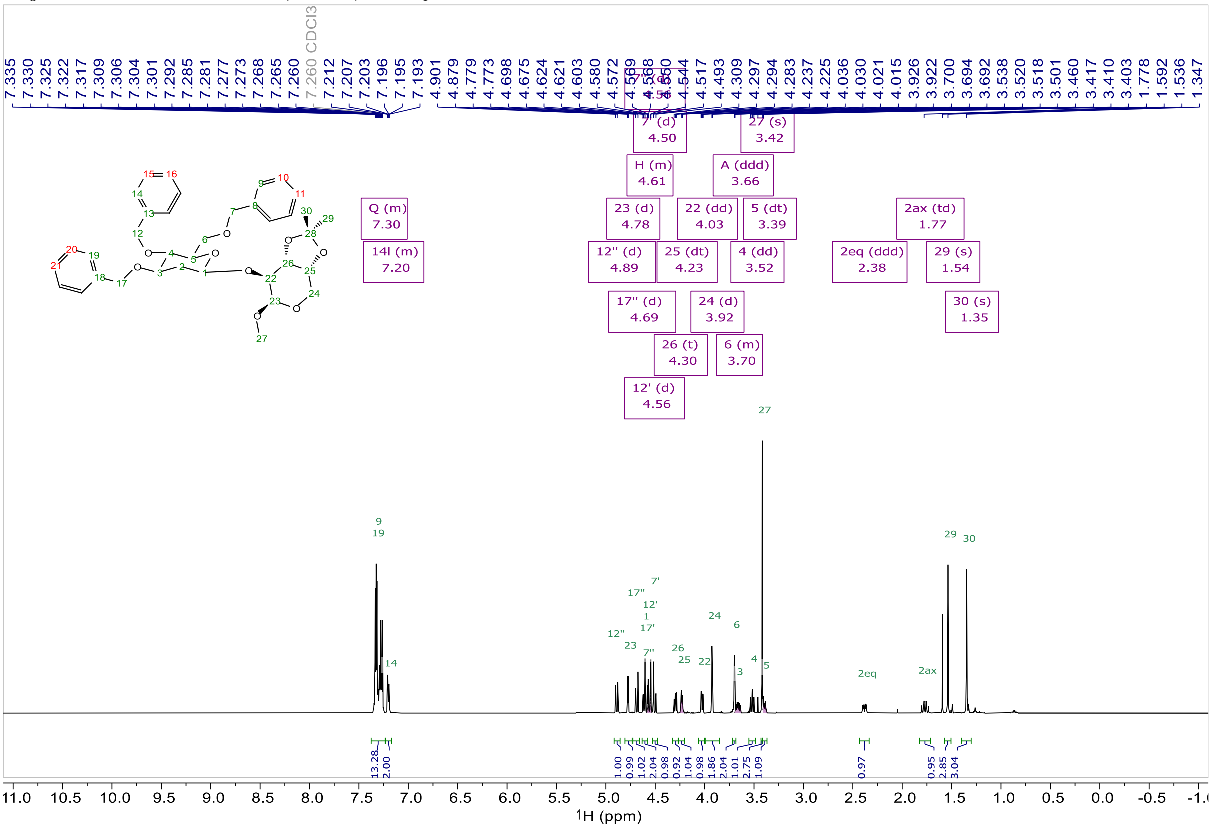

13C{},1D, 125.94 MHz,CDCl3,298.0K, pulse sequence: zgpg30

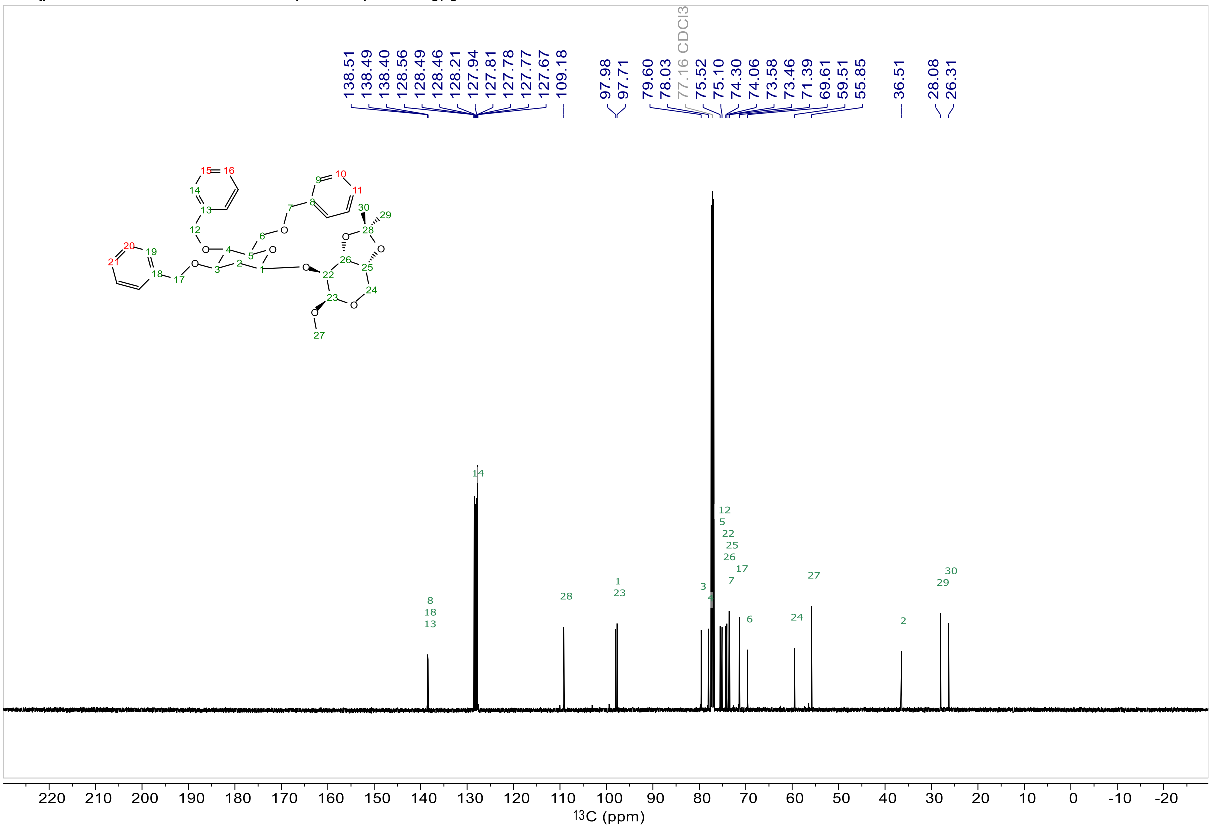

1H{},COSY, 500.81 MHz,CDCl3,298.0K, pulse sequence: cosygpppqf

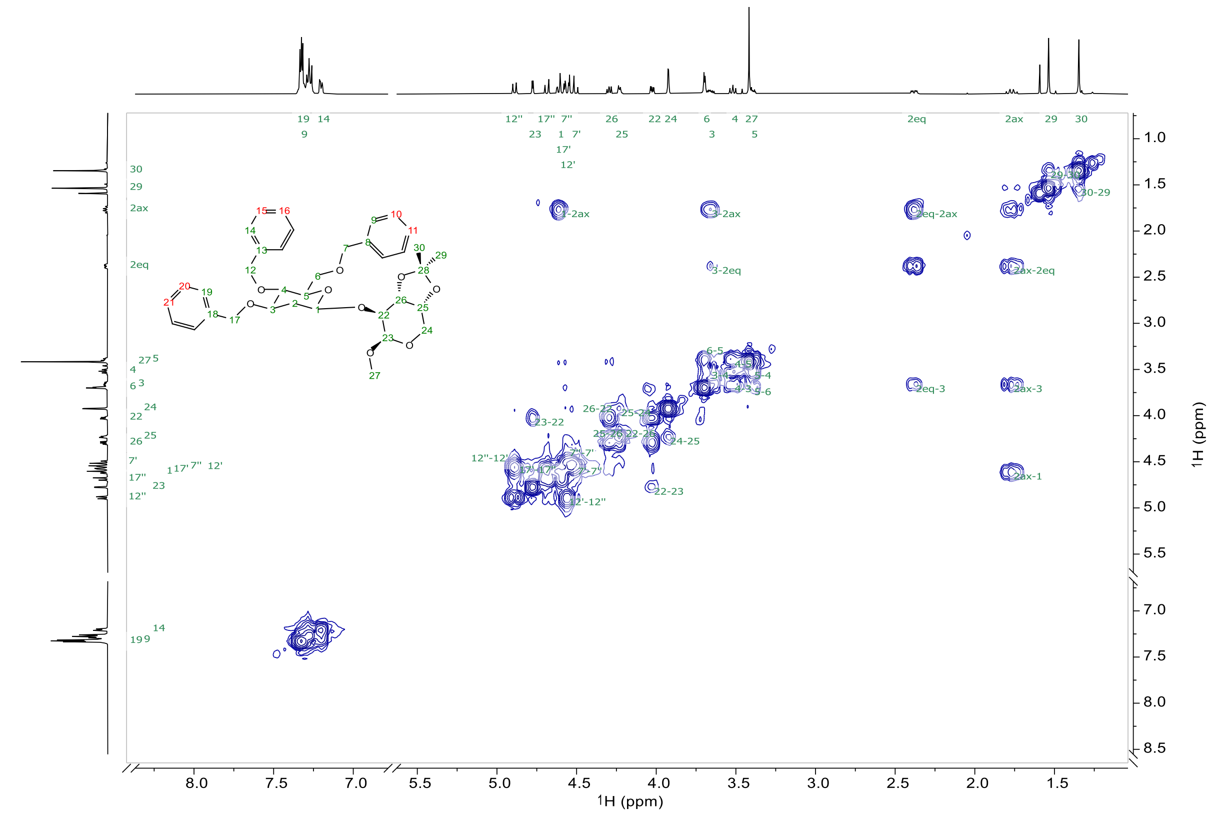

1H{},HSQC-EDITED, 500.81 MHz,CDCl3,298.0K, pulse sequence: hsqcedetgpsisp2.3

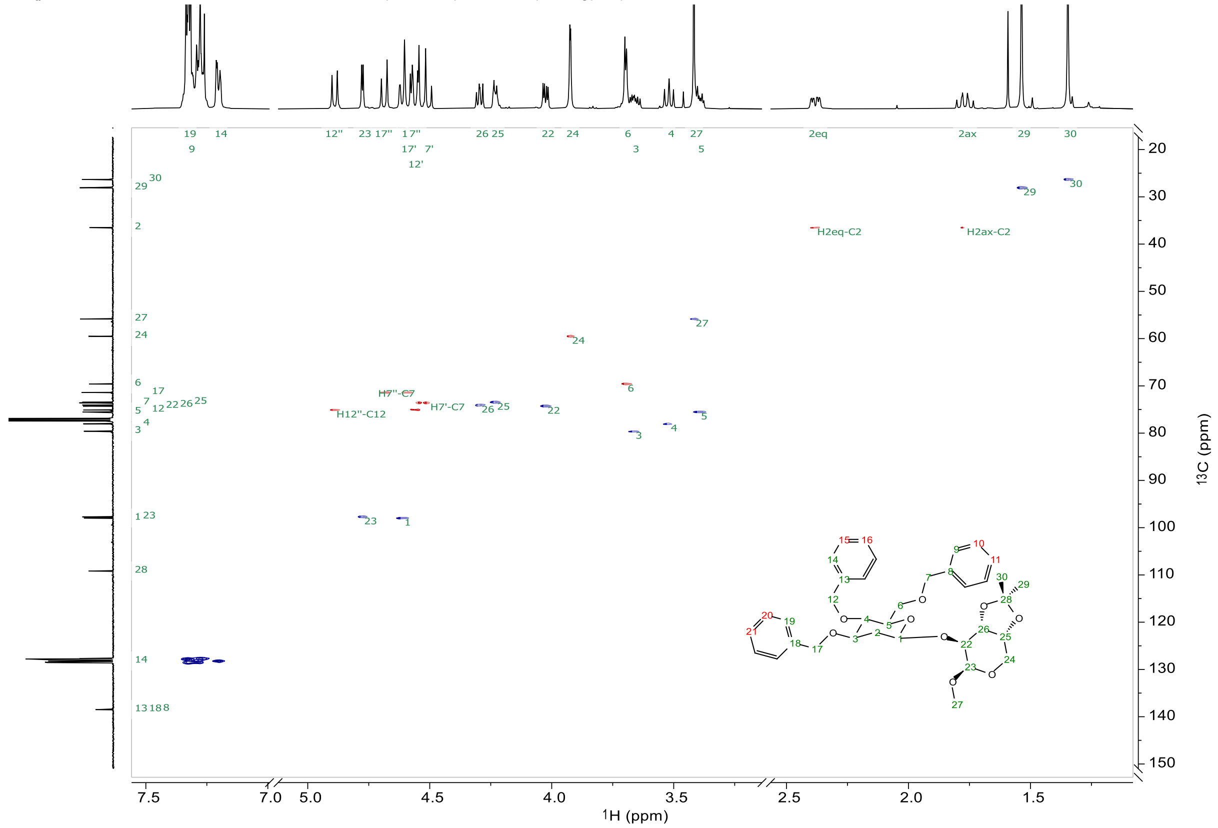

1H{},HMBC, 500.81 MHz,CDCl3,298.0K, pulse sequence: hmbcetgpl3nd

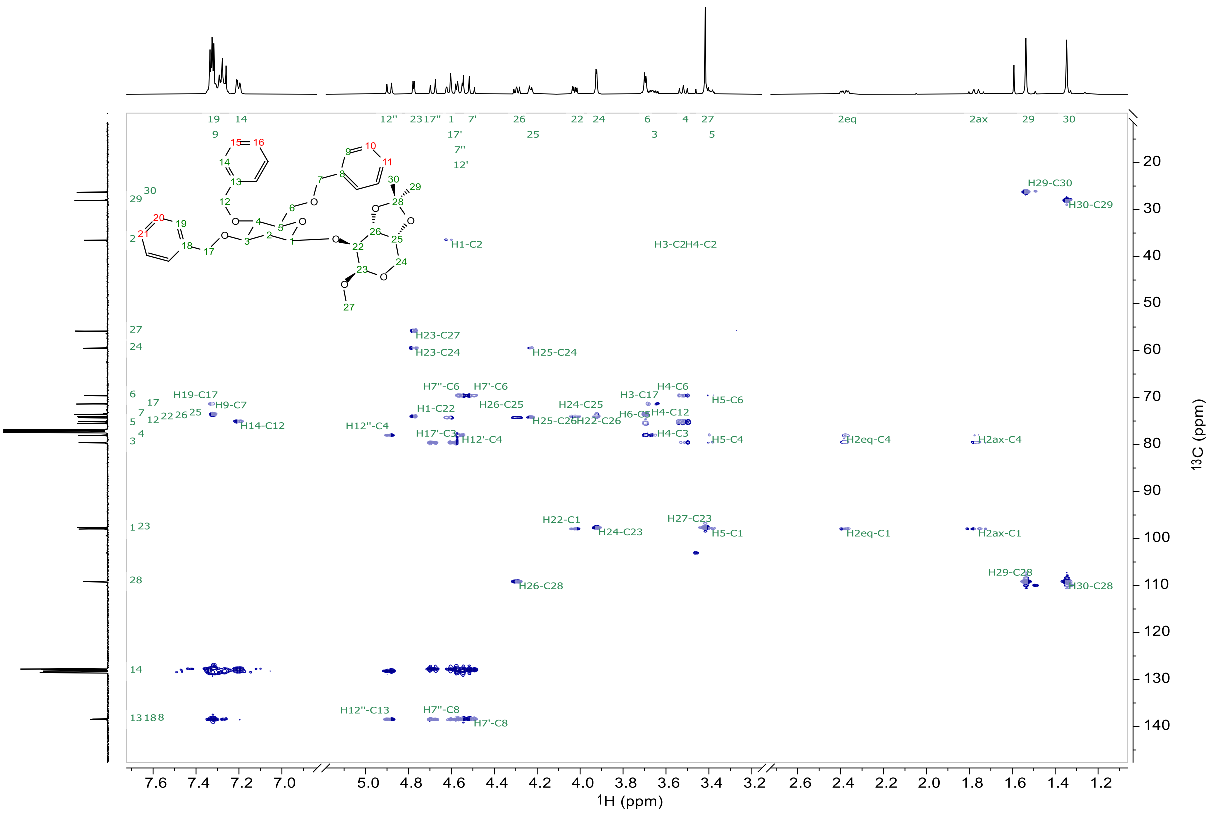

1H{},NOESY, 500.81 MHz,CDCl3,298.0K, pulse sequence: noesygpphpp

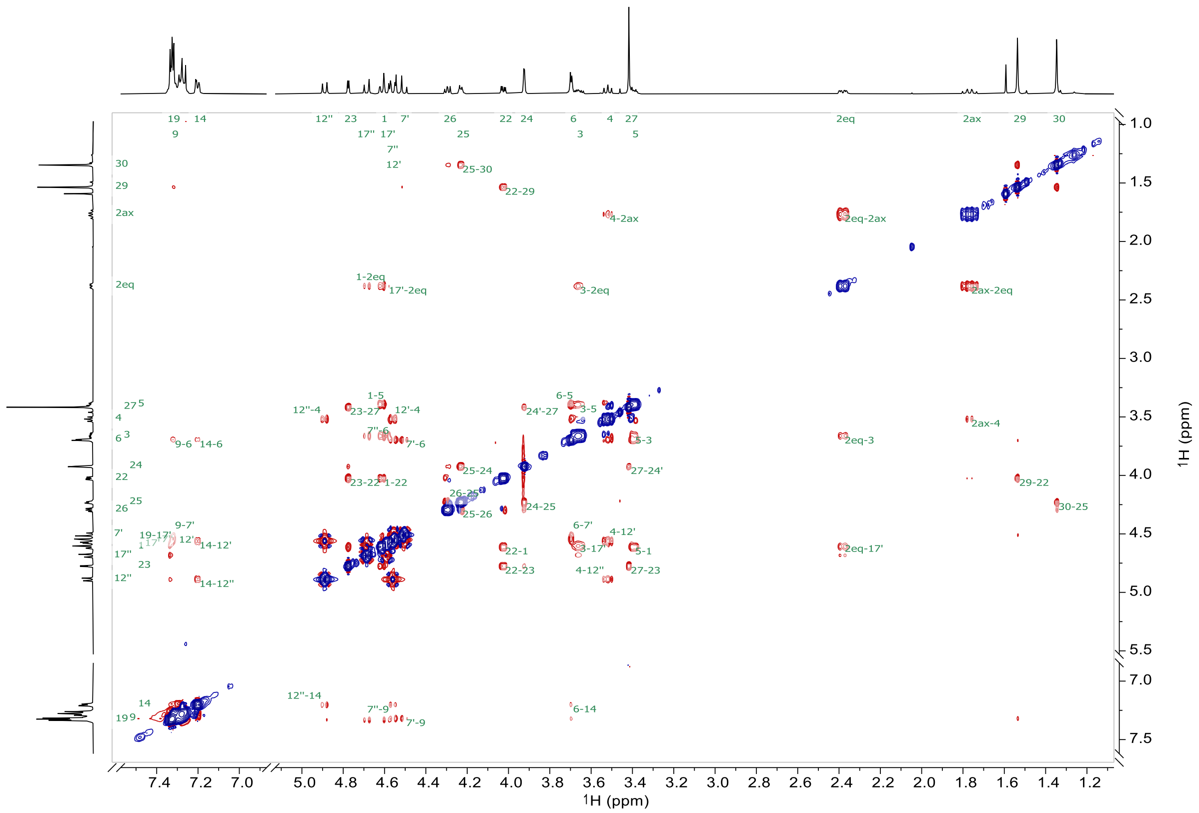

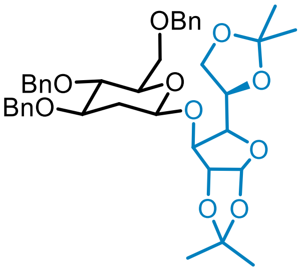

(3a*R*,5*R*,6*S*,6a*R*)-6-(((2*R*,4*S*,6*S*)-4,5-bis(benzyloxy)-6-((benzyloxy)methyl)tetrahydro-2*H*-pyran-2-yl)oxy)-5-((*S*)-2,2-dimethyl-1,3-dioxolan-4-yl)-2,2-dimethyltetrahydrofuro[2,3-d][1,3]dioxole (4d)

Following **General Procedure B**, glycosyl donor **1a** (29 mg, 0.050 mmol) and alcohol **2d** (20 mg, 0.075 mmol) were coupled using (*R*,*R*)-IDPi **3a** (9.4 mg,10 mol%). Reaction time: 72 h. β/α >95:5 was determined by crude ^{1}H NMR. Flash chromatography (hexane/EtOAc = 6/1 to 3/1) gave **4d** as off-white solid (32 mg, 96%). The NMR data of this compound are consistent with those reported in the literature.[6]

Following **General Procedure A** on 0.01 mmol scale, the glycosylation was carried out using (*S*,*S*)-IDPi **3a**. β/α >95:5 was determined by crude ^{1}H NMR.

TLC: R_f = 0.30 (Hexane/EtOAc = 3:1)

^{1}H NMR (501 MHz, $CDCl_3$) δ 1.16 – 1.29 (m, 9H), 1.35 (s, 3H), 1.43 (s, 3H), 1.50 – 1.59 (m, 1H), 2.20 (ddd, *J* = 12.6, 5.0, 2.0 Hz, 1H), 3.31 (ddd, *J* = 9.6, 4.2, 2.2 Hz, 1H), 3.48 (t, *J* = 9.2 Hz, 1H), 3.58 (ddd, *J* = 11.5, 8.7, 5.0 Hz, 1H), 3.63 – 3.71 (m, 2H), 3.91 – 4.02 (m, 2H), 4.26 – 4.32 (m, 2H), 4.33 – 4.38 (m, 1H), 4.43 (d, *J* = 3.8 Hz, 1H), 4.46 – 4.51 (m, 2H), 4.51 – 4.64 (m, 4H), 4.82 (d, *J* = 10.8 Hz, 1H), 5.85 (d, *J* = 3.8 Hz, 1H), 7.16 (dd, *J* = 7.8, 1.8 Hz, 2H), 7.19 – 7.30 (m, 13H).

^{13}C NMR (126 MHz, $CDCl_3$) *δ* 25.45, 26.41, 26.66, 26.90, 36.70, 65.91, 69.13, 71.62, 73.70, 75.18, 75.63, 77.98, 79.24, 79.27, 80.45, 83.02, 97.79, 105.24, 108.54, 111.97, 127.75, 127.84, 127.88, 128.21, 128.51, 128.52, 128.58, 138.38, 138.40, 138.41.

$[\alpha]_D^{22}$ = -5.4 ($CHCl_3$, *c* = 1.0).

HRMS m/z (ESI): calcd. for C39 H48 Na1 O10 ($[M+Na]^+$): 699.31397; found: 699.31429.

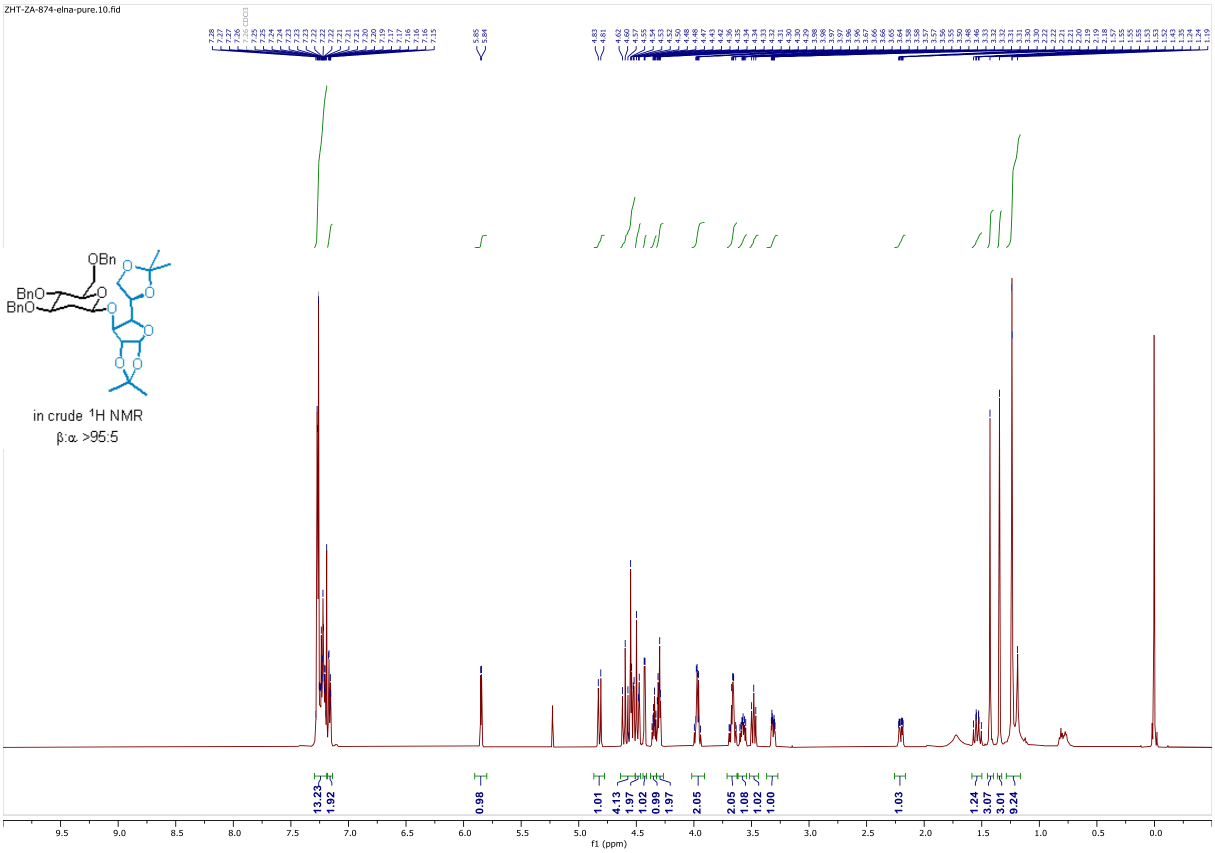
ZHT-ZA-874-elna-pure.10.fid
OBn
BnO
BnO
in crude ¹H NMR
β:α >95:5
f1 (ppm)

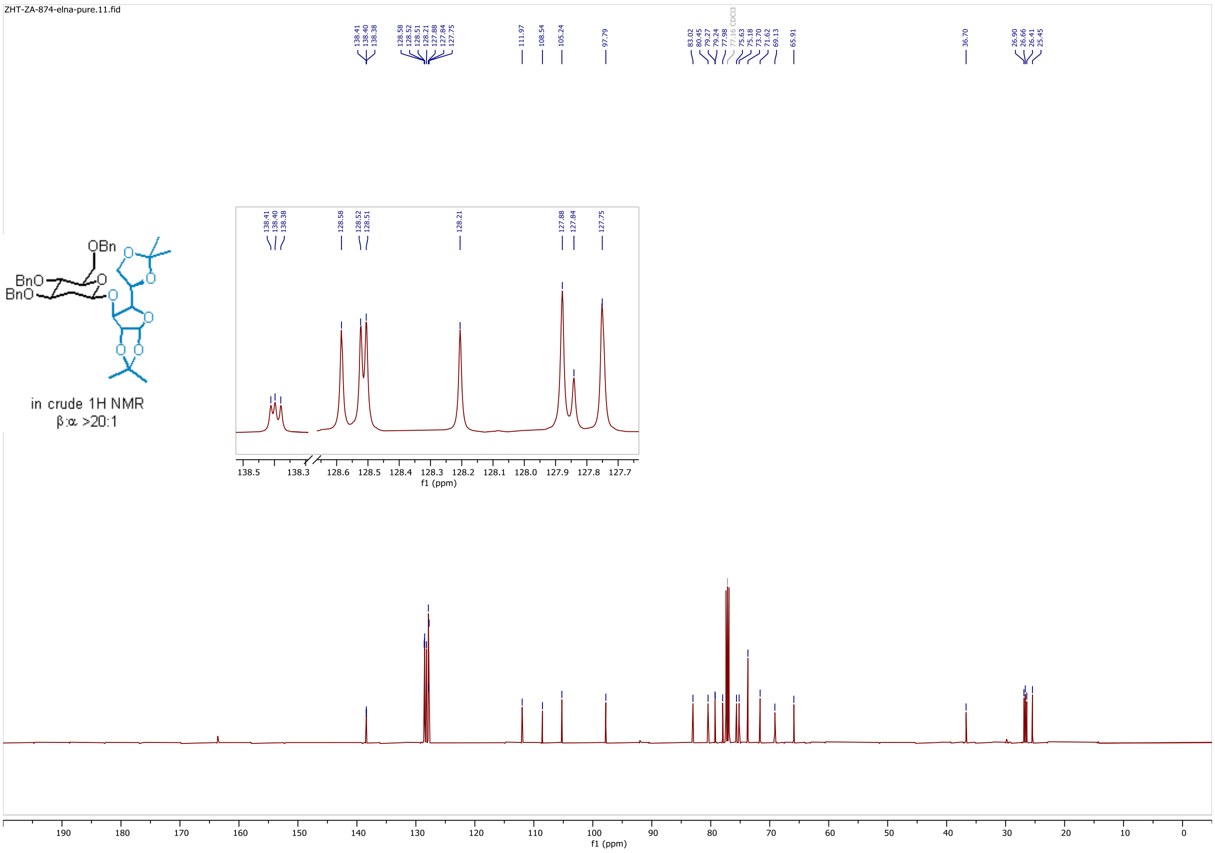
ZHT-ZA-874-elna-pure.11.fid
OBn
BnO
BnO
in crude 1H NMR
β:α >20:1
f1 (ppm)

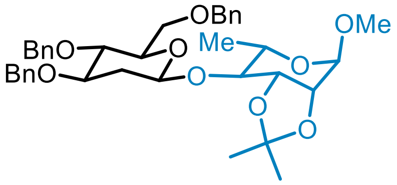

(3a*R*,4*R*,6*S*,7*S*,7a*R*)-7-(((2*S*,4*R*,5*S*,6*R*)-4,5-bis(benzyloxy)-6-((benzyloxy)methyl)tetrahydro-2*H*-pyran-2-yl)oxy)-4-methoxy-2,2,6-trimethyltetrahydro-4*H*-[1,3]dioxolo[4,5-c]pyran (4e)

Following **General Procedure B**, glycosyl donor **1a** (29 mg, 0.050 mmol) and alcohol **2e** (20 mg, 0.075 mmol) were coupled using (*R*,*R*)-IDPi **3a** (9.4 mg,10 mol%). Reaction time: 72 h. β/α >95:5 was determined by crude ^{1}H NMR. Flash chromatography (hexane/EtOAc = 8/1 to 5/1) gave **4e** as colorless oil (26 mg, 82%). The NMR data of this compound are consistent with those reported in the literature.[7]

Following **General Procedure A** on 0.01 mmol scale, the glycosylation was carried out using (*S*,*S*)-IDPi **3a**. β/α = 85:15 was determined by crude ^{1}H NMR.

TLC: R_f = 0.61 (Hexane/EtOAc = 3:1)

^{1}H NMR (501 MHz, $CDCl_3$) δ 1.26 (t, *J* = 3.1 Hz, 6H), 1.39 (s, 3H), 1.49 – 1.57 (m, 1H), 2.32 (ddd, *J* = 12.4, 5.1, 1.9 Hz, 1H), 3.30 (s, 4H), 3.47 – 3.58 (m, 2H), 3.59 – 3.66 (m, 3H), 3.69 (dd, *J* = 11.0, 4.3 Hz, 1H), 4.01 (d, *J* = 5.5 Hz, 1H), 4.09 (dd, *J* = 7.4, 5.5 Hz, 1H), 4.46 – 4.66 (m, 5H), 4.79 (s, 1H), 4.81 – 4.88 (m, 2H), 7.14 – 7.33 (m, 15H).

^{13}C NMR (126 MHz, $CDCl_3$) *δ* 17.77, 26.59, 27.99, 37.05, 54.93, 64.42, 69.31, 71.46, 73.62, 75.06, 75.48, 76.23, 77.87, 78.20, 78.76, 79.74, 98.01, 98.66, 109.41, 127.62, 127.72, 127.74, 127.75, 128.12, 128.48, 128.55, 138.63, 138.69.

$[\alpha]_D^{22}$ = -13.6 ($CHCl_3$, *c* = 1.0).

HRMS m/z (ESI): calcd. for C37 H46 O9 Na1 ($[M+Na]^+$): 657.303404; found: 657.303850.

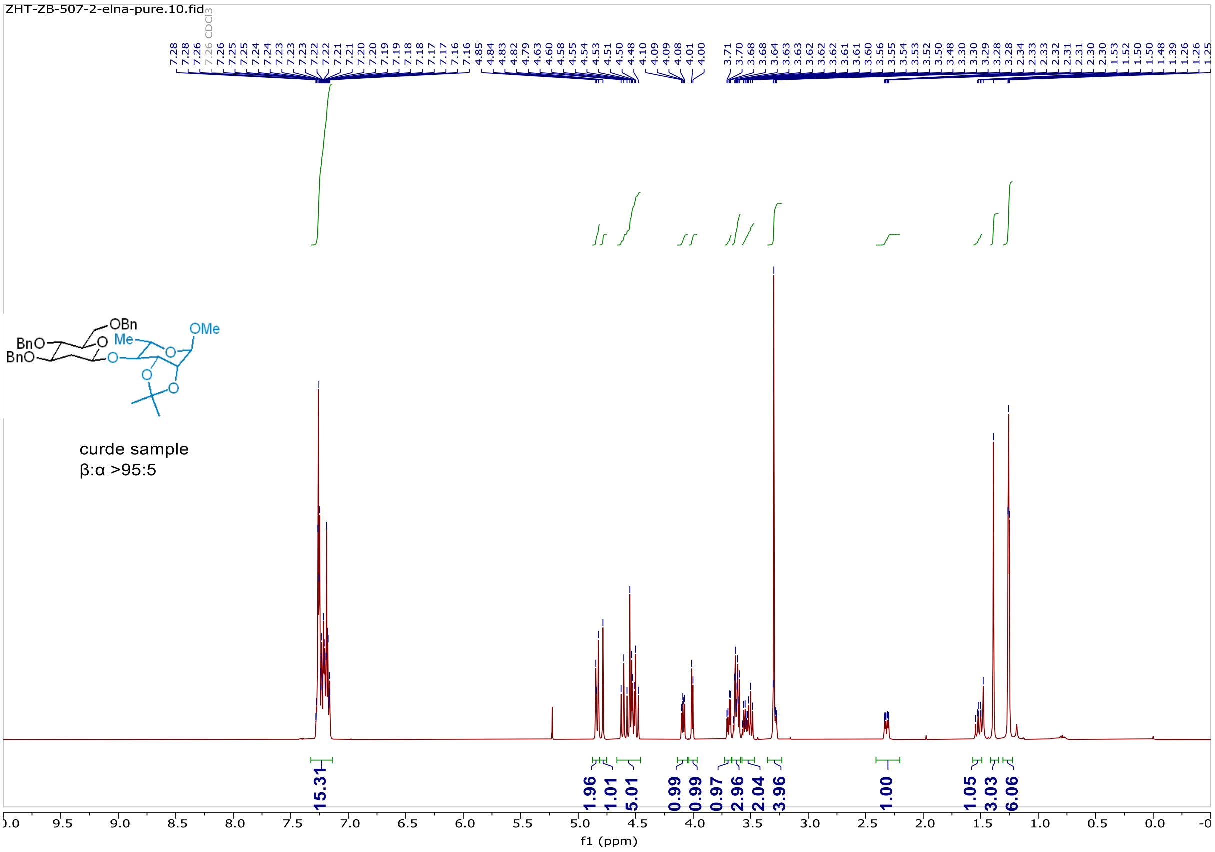
ZHT-ZB-507-2-elna-pure.10.fid
CDCl3
7.28 7.28 7.26 7.26 7.25 7.25 7.24 7.24 7.23 7.23 7.22 7.22 7.22 7.21 7.21 7.20 7.20 7.19 7.19 7.18 7.18 7.17 7.17 7.16 7.16 4.85 4.84 4.83 4.82 4.79 4.63 4.60 4.58 4.55 4.54 4.53 4.51 4.50 4.48 4.10 4.09 4.09 4.08 4.01 4.00 3.71 3.70 3.68 3.68 3.64 3.63 3.63 3.62 3.62 3.62 3.61 3.61 3.60 3.56 3.55 3.54 3.53 3.52 3.50 3.48 3.30 3.29 3.28 3.28 2.34 2.33 2.33 2.32 2.31 2.31 2.30 2.30 1.53 1.52 1.50 1.50 1.48 1.39 1.26 1.25
OBn
BnO
BnO
O
O
Me
O
OMe
O
O
curde sample
β:α >95:5
15.31
1.96
1.01
5.01
0.99
0.99
0.97
2.96
2.04
3.96
1.00
1.05
3.03
6.06
f1 (ppm)

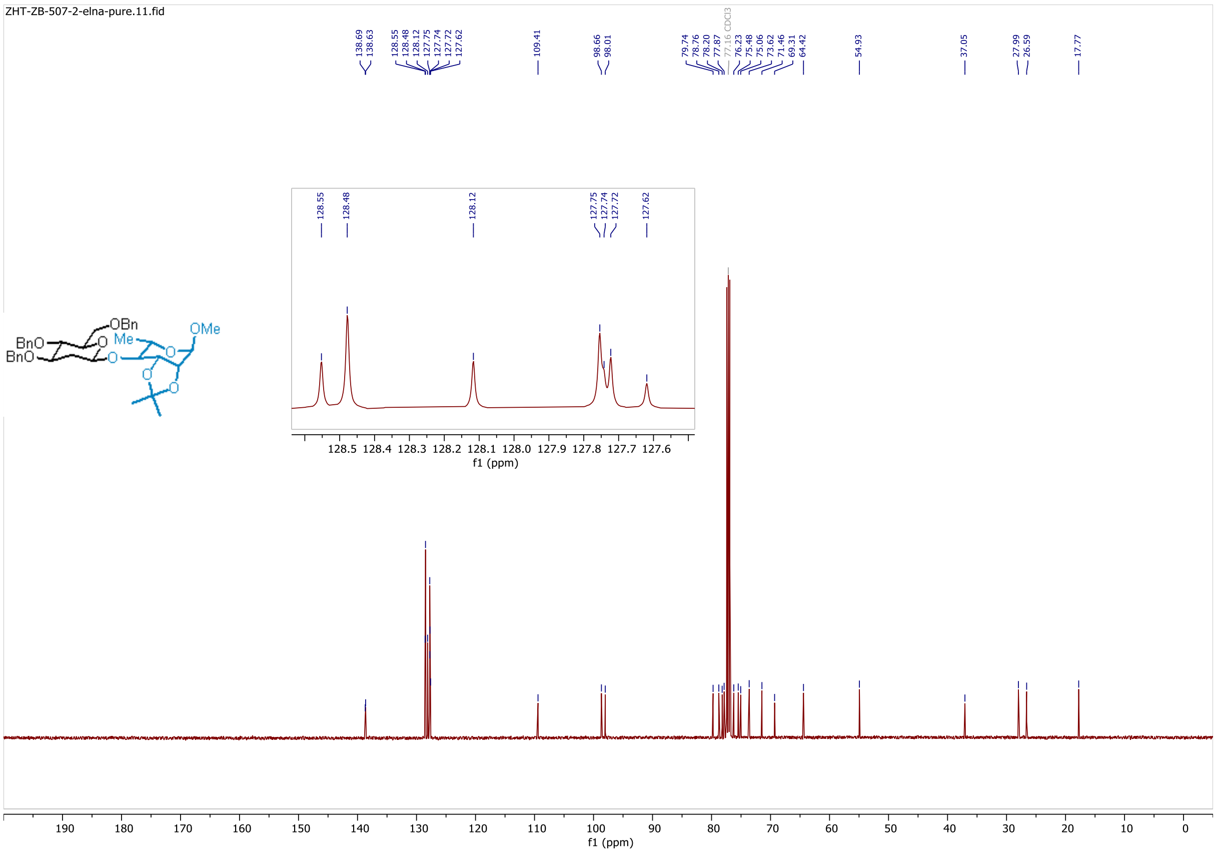
ZHT-ZB-507-2-elna-pure.11.fid
138.69 138.63 128.55 128.48 128.12 127.75 127.74 127.72 127.62 109.41 98.66 98.01 79.74 78.76 78.20 77.87 77.16 CDCl3 76.23 75.48 75.06 73.62 71.46 69.31 64.42 54.93 37.05 27.99 26.59 17.77
128.55 128.48 128.12 127.75 127.74 127.72 127.62
f1 (ppm)
OBn
BnO
BnO
O
O
Me
O
OMe
O
O
f1 (ppm)

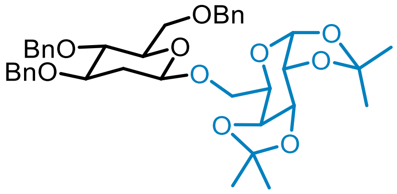

(3a*R*,5*R*,5a*S*,8a*S*,8b*R*)-5-((((2*R*,4*R*,5*S*,6*R*)-4,5-bis(benzyloxy)-6-((benzyloxy)methyl)tetrahydro-2*H*-pyran-2-yl)oxy)methyl)-2,2,7,7-tetramethyltetrahydro-5*H*-bis([1,3]dioxolo)[4,5-b:4',5'-d]pyran (4f)

Following **General Procedure B**, glycosyl donor **1a** (29 mg, 0.050 mmol) and alcohol **2f** (20 mg, 0.075 mmol) were coupled using (*R*,*R*)-IDPi **3a** (9.4 mg,10 mol%). Reaction time: 24 h. β/α = 94:6 was determined by crude ^{1}H NMR. Flash chromatography (hexane/EtOAc = 6/1 to 3/1) gave **4f** as colorless oil (34 mg, 99%). The NMR data of this compound are consistent with those reported in the literature.[6]

Following **General Procedure A** on 0.01 mmol scale, the glycosylation was carried out using (*S*,*S*)-IDPi **3a**. β/α = 71:29 was determined by crude ^{1}H NMR.

TLC: R_f = 0.33 (Hexane/EtOAc = 3:1)

^{1}H NMR (501 MHz, CDCl$_3$) δ 1.33 (d, *J* = 7.5 Hz, 6H), 1.44 (s, 3H), 1.54 (s, 3H), 1.66 (td, *J* = 12.1, 9.7 Hz, 1H), 2.46 (ddd, *J* = 12.6, 5.1, 1.9 Hz, 1H), 3.40 (ddd, *J* = 9.7, 4.3, 2.4 Hz, 1H), 3.54 (t, *J* = 9.1 Hz, 1H), 3.65 (ddd, *J* = 11.6, 7.7, 5.5 Hz, 2H), 3.70 – 3.79 (m, 2H), 3.97 – 4.03 (m, 1H), 4.06 – 4.10 (m, 1H), 4.22 (dd, *J* = 7.9, 2.0 Hz, 1H), 4.31 (dd, *J* = 5.0, 2.4 Hz, 1H), 4.49 – 4.71 (m, 7H), 4.90 (d, *J* = 10.8 Hz, 1H), 5.55 (d, *J* = 5.0 Hz, 1H), 7.15 – 7.22 (m, 2H), 7.23 – 7.38 (m, 13H).

^{13}C NMR (126 MHz, CDCl$_3$) *δ* 24.54, 25.13, 26.13, 26.21, 36.70, 67.88, 68.99, 69.33, 70.55, 70.87, 71.39, 71.65, 73.61, 75.05, 75.25, 79.52, 96.50, 100.57, 108.77, 109.47, 127.67, 127.75, 127.79, 127.89, 127.99, 128.10, 128.45, 128.46, 128.56, 138.45, 138.60.

$[\alpha]_D^{22}$ = -49.2 (CHCl$_3$, *c* = 1.0).

HRMS m/z (ESI): calcd. for C39 H48 Na1 O10 ([M+Na]$^+$): 699.31397; found: 699.31358.

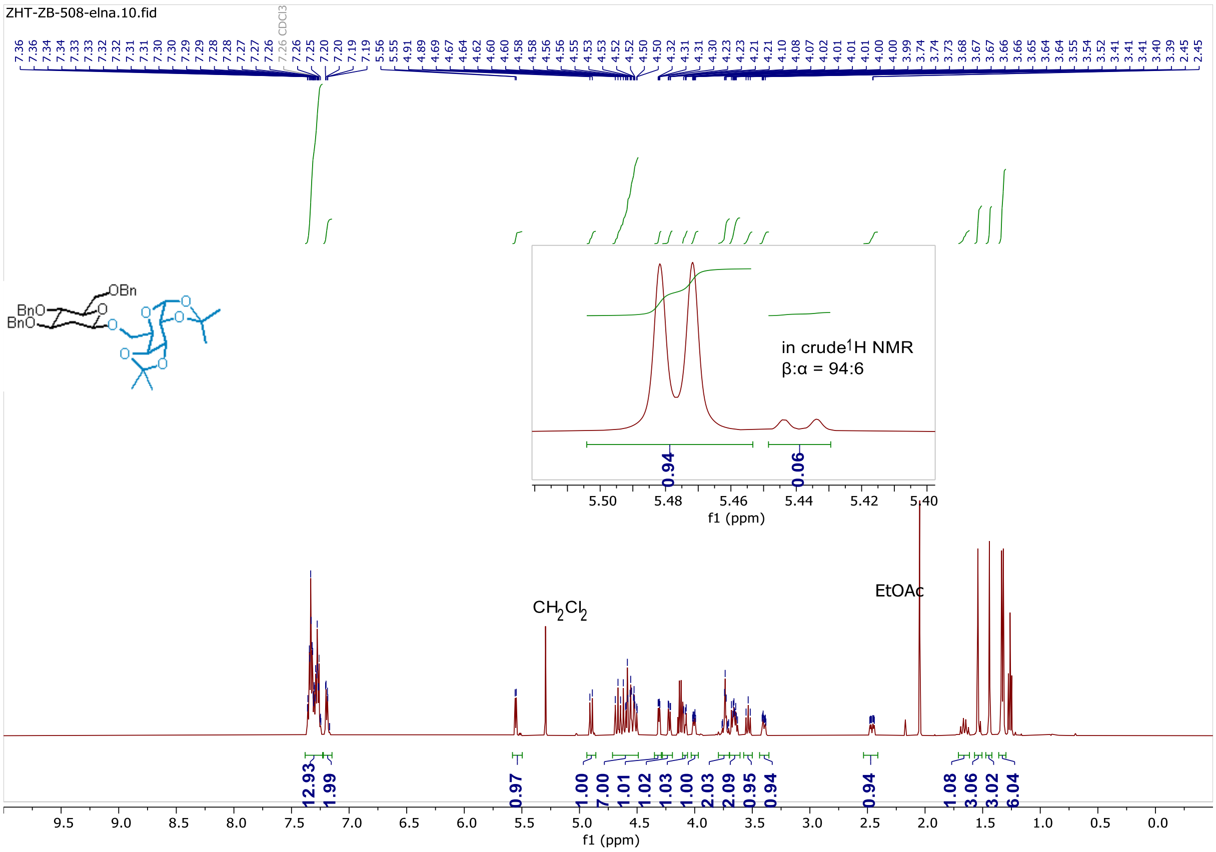
ZHT-ZB-508-elna.10.fid
7.36 7.36 7.34 7.34 7.33 7.33 7.32 7.32 7.31 7.31 7.30 7.30 7.29 7.29 7.28 7.28 7.27 7.27 7.26 7.26 CDCl3 7.26 7.25 7.20 7.20 7.19 7.19 5.56 5.55 4.91 4.89 4.69 4.67 4.64 4.62 4.60 4.60 4.58 4.58 4.56 4.56 4.55 4.53 4.53 4.52 4.52 4.50 4.50 4.32 4.31 4.31 4.30 4.23 4.23 4.21 4.21 4.10 4.08 4.07 4.02 4.01 4.01 4.01 4.00 3.99 3.74 3.74 3.73 3.68 3.67 3.67 3.66 3.66 3.65 3.64 3.64 3.55 3.54 3.52 3.41 3.41 3.40 3.39 2.45 2.45
BnO
BnO
OBn
O
O
O
O
O
O
O
in crude ^{1}H NMR
β:α = 94:6
0.94
0.06
5.50 5.48 5.46 5.44 5.42 5.40
f1 (ppm)
CH_2Cl_2
EtOAc
12.93 1.99 0.97 1.00 7.00 1.01 1.02 1.03 1.00 2.03 2.09 0.95 0.94 0.94 1.08 3.06 3.02 6.04
9.5 9.0 8.5 8.0 7.5 7.0 6.5 6.0 5.5 5.0 4.5 4.0 3.5 3.0 2.5 2.0 1.5 1.0 0.5 0.0
f1 (ppm)

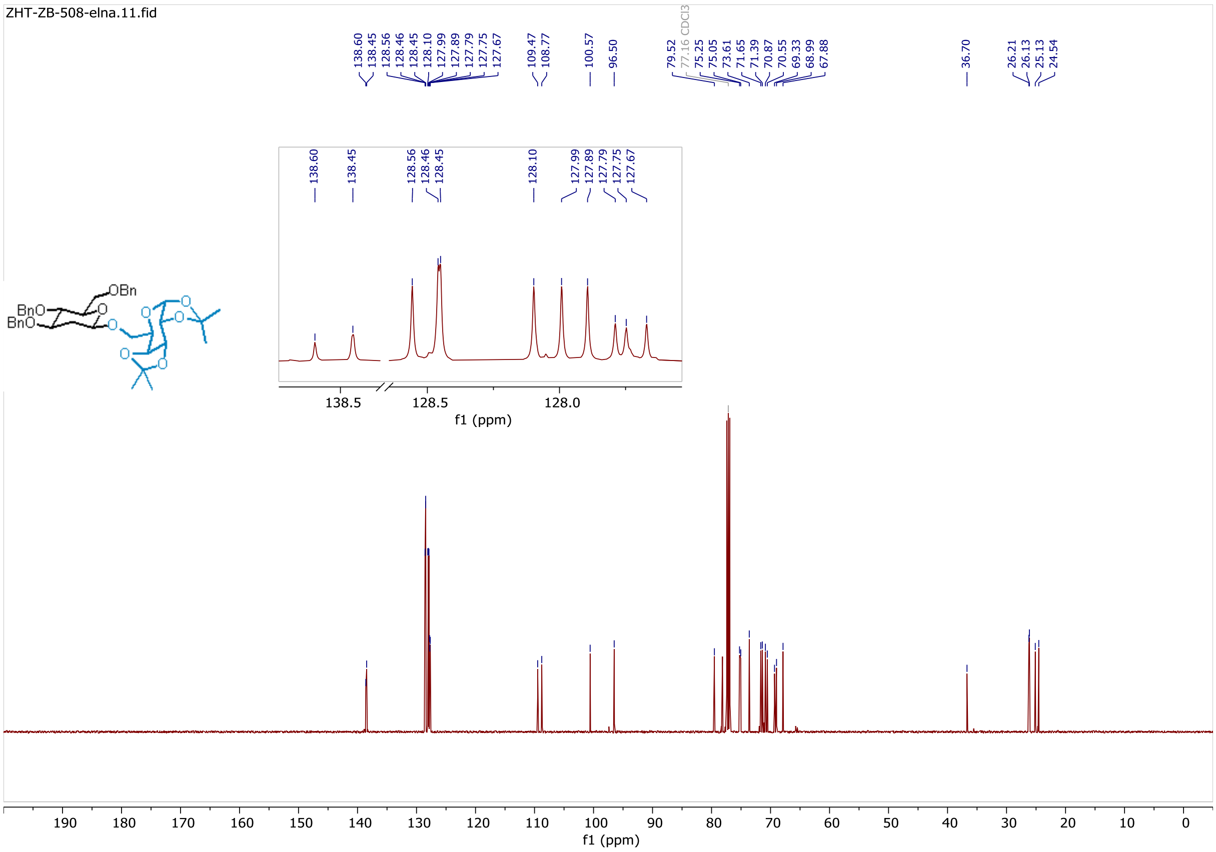
ZHT-ZB-508-elna.11.fid
138.60 138.45 128.56 128.46 128.45 128.10 127.99 127.89 127.79 127.75 127.67 109.47 108.77 100.57 96.50 79.52 77.16 CDCl3 75.25 75.05 73.61 71.65 71.39 70.87 70.55 69.33 68.99 67.88 36.70 26.21 26.13 25.13 24.54
BnO
BnO
OBn
O
O
O
O
O
O
O
138.60 138.45 128.56 128.46 128.45 128.10 127.99 127.89 127.79 127.75 127.67
138.5 128.5 128.0
f1 (ppm)
190 180 170 160 150 140 130 120 110 100 90 80 70 60 50 40 30 20 10 0
f1 (ppm)

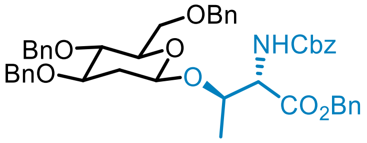

benzyl *N*-((*benzyloxy*)carbonyl)-*O*-((2*R*,4*R*,5*S*,6*R*)-4,5-bis(*benzyloxy*)-6-((*benzyloxy*)methyl)tetrahydro-2*H*-pyran-2-yl)-L-threoninate (4g)

Following **General Procedure B**, glycosyl donor **1a** (29 mg, 0.050 mmol) and alcohol **2g** (20 mg, 0.075 mmol) were coupled using (*R*,*R*)-IDPi **3a** (9.4 mg,10 mol%). Reaction time: 24 h. β/α = 94:6 was determined by crude ^{1}H NMR. Flash chromatography (hexane/EtOAc = 5/1 to 3/1) gave **4g** as light-yellow oil (38 mg, 99%).

Following **General Procedure A** on 0.01 mmol scale, the glycosylation was carried out using (*S*,*S*)-IDPi **3a**. β/α = 92:8 was determined by crude ^{1}H NMR.

TLC: R_f = 0.36 (Hexane/EtOAc = 3:1)

^{1}H NMR (600 MHz, CDCl$_3$) δ 1.23 (d, *J* = 6.4 Hz, 4H), 1.57 (ddd, *J* = 12.6, 11.5, 9.8 Hz, 1H), 2.23 (ddd, *J* = 12.6, 4.9, 1.9 Hz, 1H), 3.26 (dt, *J* = 9.5, 3.9 Hz, 1H), 3.54 (t, *J* = 9.1 Hz, 1H), 3.60 (ddd, *J* = 11.5, 8.8, 4.9 Hz, 1H), 3.62 – 3.68 (m, 2H), 4.36 (dd, *J* = 8.6, 3.2 Hz, 1H), 4.38 (d, *J* = 12.0 Hz, 1H), 4.44 (dd, *J* = 9.8, 1.9 Hz, 1H), 4.47 (d, *J* = 12.0 Hz, 1H), 4.47 (qd, *J* = 6.4, 3.2 Hz, 1H), 4.55 (d, *J* = 10.8 Hz, 1H), 4.59 (d, *J* = 11.7 Hz, 1H), 4.67 (d, *J* = 11.7 Hz, 1H), 4.88 (d, *J* = 10.8 Hz, 1H), 5.13 (s, 2H), 5.13 (d, *J* = 12.4 Hz, 1H), 5.18 (d, *J* = 12.4 Hz, 1H), 5.87 (d, *J* = 8.6 Hz, 1H), 7.18 – 7.26 (m, 2H), 7.23 – 7.37 (m, 22H).

^{13}C NMR (151 MHz, CDCl$_3$) δ 17.67, 36.81, 59.07, 67.18 (d, *J* = 5.1 Hz), 69.11, 71.45, 73.32, 73.49, 75.07, 75.14, 77.75, 79.23, 97.46, 127.61, 127.72 – 127.85 (m), 128.12, 128.23, 128.36, 128.42, 128.47, 128.55, 128.62 (d, *J* = 2.3 Hz), 135.66, 136.45, 138.34, 138.43, 138.47, 156.91, 170.46.

$[\alpha]_D^{22}$ = -1.8(CHCl$_3$, *c* = 1.0).

HRMS m/z (ESI): calcd. for C46 H49 N1 Na1 O9 ([M+Na]$^+$): 782.32995; found: 782.32963.

User Report
ZHT-ZB-509-01

NMR data supports the formation of the beta-anomer of the following structure:

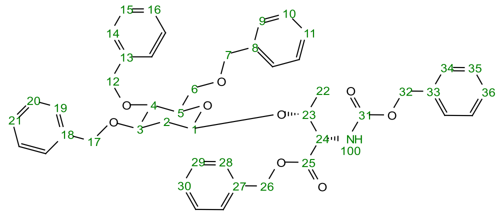

Remarks:
The relative stereochemistry at C1,C3,C4 &C 5 is supported by the observed ^3J values (^3J > 8.0 Hz → dominantly *trans* in the presence of multiple oxygen atoms). Additional support comes from NOE correlations of H1 to H3 and H5, consistent with all three being in axial orientation as indicated in the structure above. H2ax shows an NOE to H4 and has a large ^3J to H1 (9.8Hz, *trans*). While the relative stereochemistry within the ring is well supported by the data, the relative configuration between the ring and the threonine unit could not be unambiguously confirmed and was therefore assumed as submitted.
The ^1H NMR integrals are partially off due to the precence of a minor rotamer (~10%mol). The chemical exchange to the minor rotamer is visible as EXSY type cross peaks in the 2D NOESY. This species was not assigned in detail. Additionally, another amide is present in the sample (6.58 & 5.93 ppm; R-C=O-NH2), which could not be assigned in detail due to missing correlations, please check carefully the data from the MS department for more insights on this.

An overview of all assignments is shown on the next page. The aromatic regions exhibit significant signal overlap. High resolution, band-selective HSQC and HMBC experiments were used to support the assignments; however, some of the correlations remain ambiguous, and certain signals may be interchangeable.

P-ID: ML00412
Measured on: 09/01/2026
CHIFFRE: ZHT-ZB-509-01
ELNA#: 15812
Client: Tianyu Zheng
Group: List
Spectroscopist: Leutzsch
Analysed on: 12/01/2026
Analysed by: Leutzsch
Amount: 30.0 mg
Solvent: CDCl3
Reference: 1H+13C on solvent,other nuclei w/ xiref
Temperature: 298 K
Spectrometer: av600a
Probe: cryoBBO
Experiments: 1H-zg30, 13C-zgpg30, 1H-13C-hsqcedetgpsisp2.3, 1H-13C-hmbcetgpl3nd, 1H-1H-cosygpppqf, 1H-1H-noesygpph, 1H-15N-hmbcgpndqf, 1H-13C-shsqcetgpsisp2.2, 1H-13C-shmbcctetgpl2nd, 1H-seldigpzs

Atom	δ (ppm)	J	COSY	HSQC	HMBC	NOESY
1 C	97.462			1	2ax, 2eq, 3, 5, 6', 23	
H	4.440	9.80(2ax), 1.90(2eq)	2ax, 2eq	1	2, 3, 5, 23	2eq, 3, 5
2 C	36.815			2ax, 2eq	1, 3, 4	
Hax	1.570	9.80(1), 11.50(3), 12.60(2eq)	1, 2eq, 3	2	1, 3, 4	2eq, 4
Heq	2.233	4.90(3), 12.60(2ax), 1.90(1)	1, 2ax, 3	2	1, 3, 4	1, 2ax, 3, 17', 17''
3 C	79.230			3	1, 2ax, 2eq, 4, 5, 17', 17''	
H	3.596	8.80(4), 11.50(2ax), 4.90(2eq)	2ax, 2eq, 4	3	1, 2, 4, 5, 17	1, 2eq, 5, 17', 17''
4 C	77.752			4	2ax, 2eq, 3, 5, 6', 6'', 12', 12''	
H	3.537	8.80(3), 9.50(5)	3, 5	4	2, 3, 5, 6, 12	2ax, 9, 12', 12'', 14
5 C	75.141			5	1, 3, 4, 6', 6''	
H	3.260	9.50(4), 3.90(6'), 2.10(6'')	4, 6', 6''	5	1, 3, 4, 6	1, 3, 6', 6''
6 C	69.106			6', 6''	4, 5, 7', 7''	
H'	3.665	3.90(5), 10.80(6'')	5	6	1, 4, 5, 7	5, 7', 7'', 9, 12', 14
H''	3.636	2.10(5), 10.80(6')	5	6	4, 5, 7	5, 7', 7'', 9, 12', 14
7 C	73.491			7', 7''	6', 6'', 9	
H'	4.375	12.00(7'')	7'', 9	7	6, 8, 9	6', 6'', 9
H''	4.470	12.00(7')	7', 9	7	6, 8, 9	6', 6'', 9
8 C	138.344				7', 7'', 10	
9 C	127.772			9	7', 7'', 9	
H	7.252		7', 7''	9	7, 9, 11	4, 6', 6'', 7', 7''
10 C	128.420			10	10	
H	7.276			10	8, 10	
11 C	127.608			11	9	
H	7.242			11		
12 C	75.074			12', 12''	4, 14	
H'	4.548	10.80(12'')	12'', 14	12	4, 13, 14	4, 6', 6'', 14
H''	4.879	10.80(12')	12', 14	12	4, 13, 14	4, 14

Atom	δ (ppm)	J	COSY	HSQC	HMBC	NOESY
13 C	138.467				12', 12'', 15	
14 C	128.118			14	12', 12'', 14	
H	7.200		12', 12'', 15	14	12, 14, 16	4, 6', 6'', 12', 12''
15 C	128.470			15	15	
H	7.301		14	15	13, 15	
16 C	127.794			16	14	
H	7.290			16		
17 C	71.446			17', 17''	3, 19	
H'	4.589	11.70(17'')	17''	17	3, 18, 19	2eq, 3, 19
H''	4.670	11.70(17')	17'	17	3, 18, 19	2eq, 3, 19
18 C	138.427				17', 17'', 20	
19 C	127.747			19	17', 17'', 19	
H	7.339			19	17, 19, 21	17', 17''
20 C	128.554			20	20	
H	7.339			20	18, 20	
21 C	127.794			21	19	
H	127.797			21		
22 C	17.669			22	23, 24	
H3	1.228	6.40(23)	23	22	23, 24	23, 24, 100
23 C	73.316			23	1, 22, 24, 100	
H	4.471	6.40(22), 3.20(24)	22, 24	23	1, 22, 100	22, 100
24 C	59.075			24	22, 100	
H	4.363	8.60(100), 3.20(23)	23, 100	24	22, 23, 25, 31, 100	22, 100
25 C	170.458				24, 26', 26'', 100	
26 C	67.194			26', 26''	28	
H'	5.133	12.40(26'')	26''	26	25, 27, 28	28
H''	5.184	12.40(26')	26'	26	25, 27, 28	28

Atom	δ (ppm)	J	COSY	HSQC	HMBC	NOESY
27 C	135.661				26', 26'', 29	
28 C	128.226			28	26', 26'', 28	
H	7.318			28	26, 28, 30	26', 26''
29 C	128.631			29	29	
H	7.318			29	27, 29	
30 C	128.355			30	28	
H	7.297			30		
31 C	156.914				24, 32, 100	
32 C	67.160			32	34	
H2	5.128		34	32	31, 33, 34	34
33 C	136.452				32, 35	
34 C	128.226			34	32, 36	
H	7.346		32	34	32	32
35 C	128.616			35	35	
H	7.345			35	33, 35	
36 C	128.226			36		
H	7.310			36	34	
100 N	-301.320				23, 24, 100	
H	5.866	8.60(24)	24		23, 24, 25, 31, 100	22, 23, 24

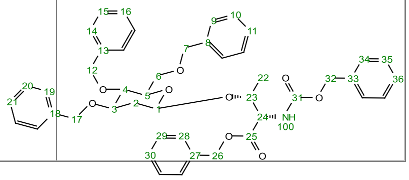

1H{off},1D, 600.23 MHz,CDCl3,298.0K, pulse sequence: zg30

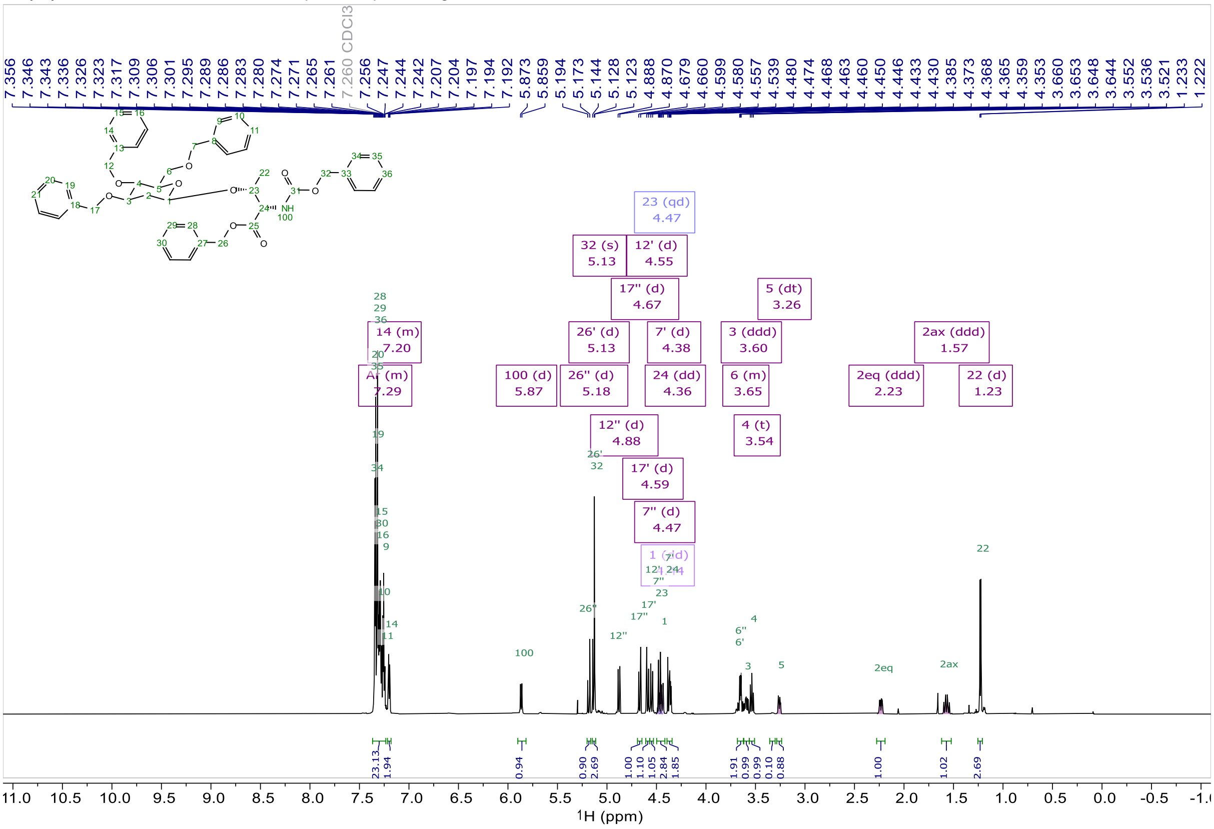

13C{1H},1D, 150.94 MHz,CDCl3,298.0K, pulse sequence: zgpg30

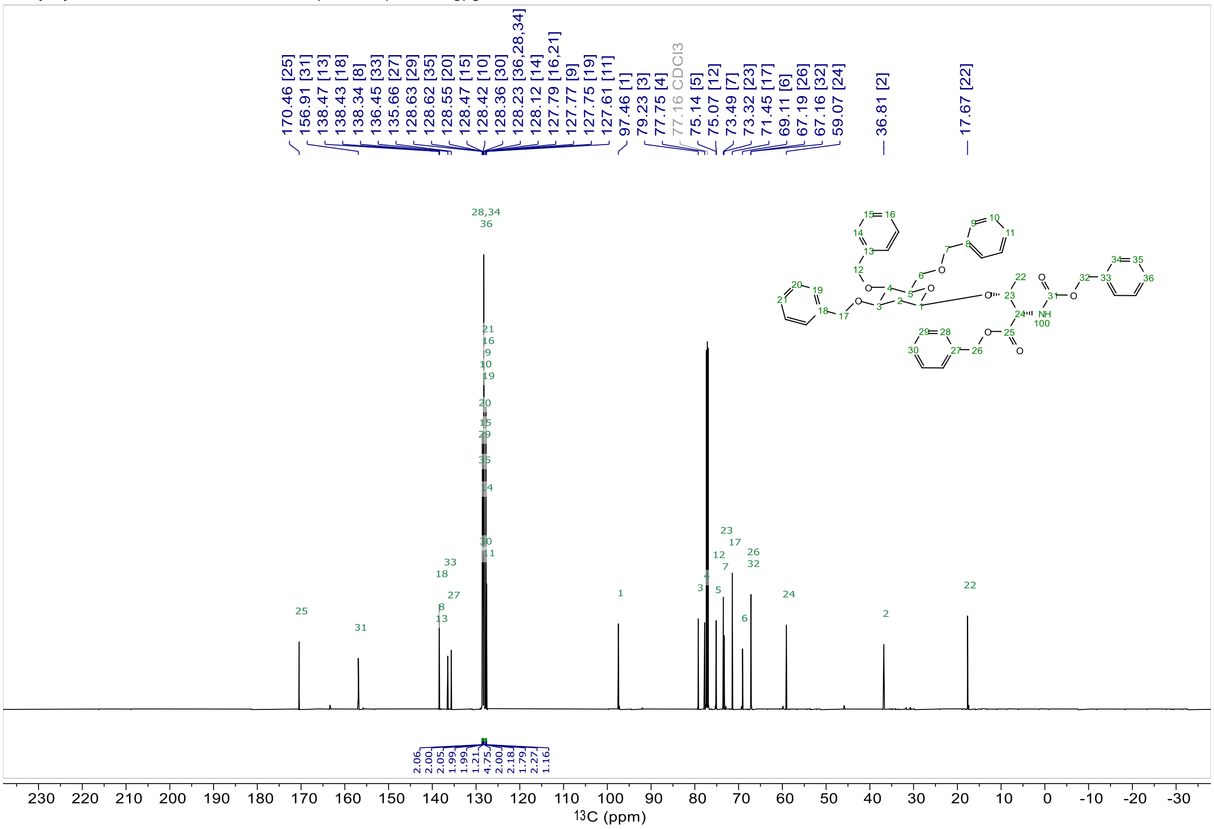

1H{off},COSY, 600.22 MHz,CDCl3,298.0K, pulse sequence: cosygpppqf

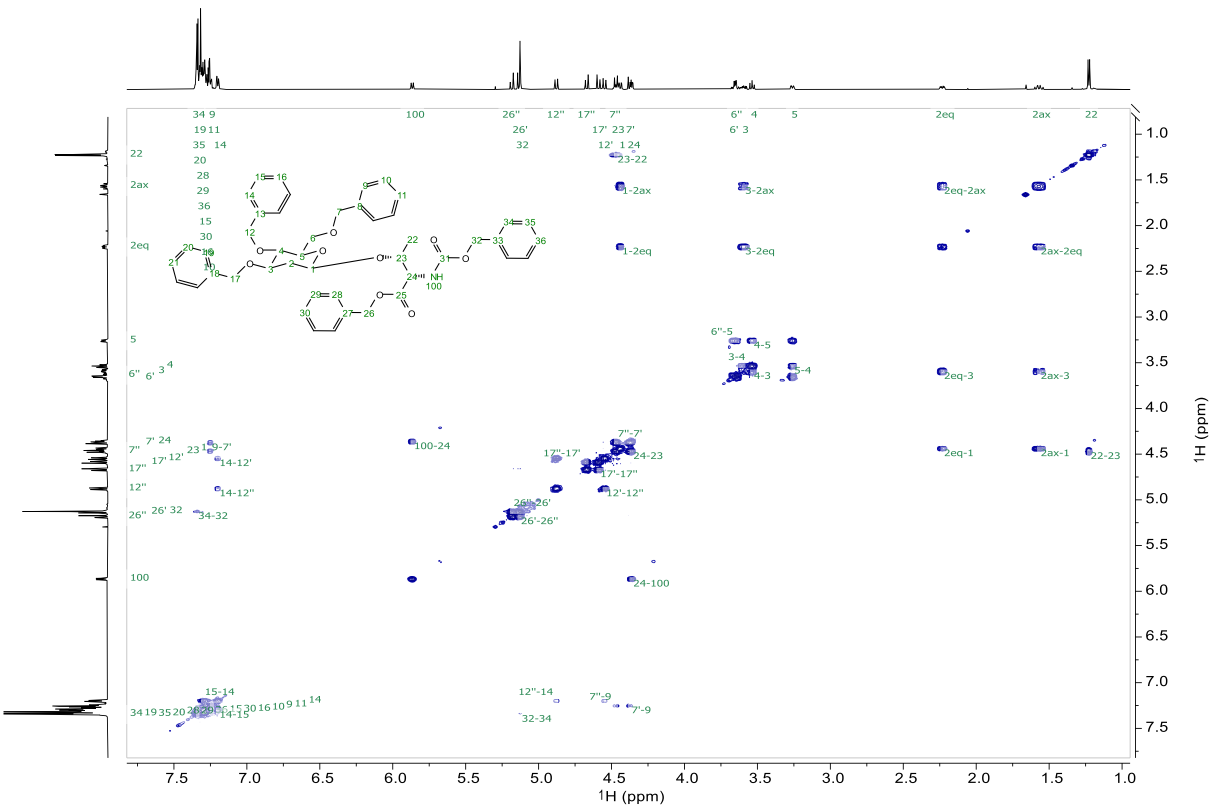

1H{off},NOESY, 600.22 MHz,CDCl3,298.0K, pulse sequence: noesygpph

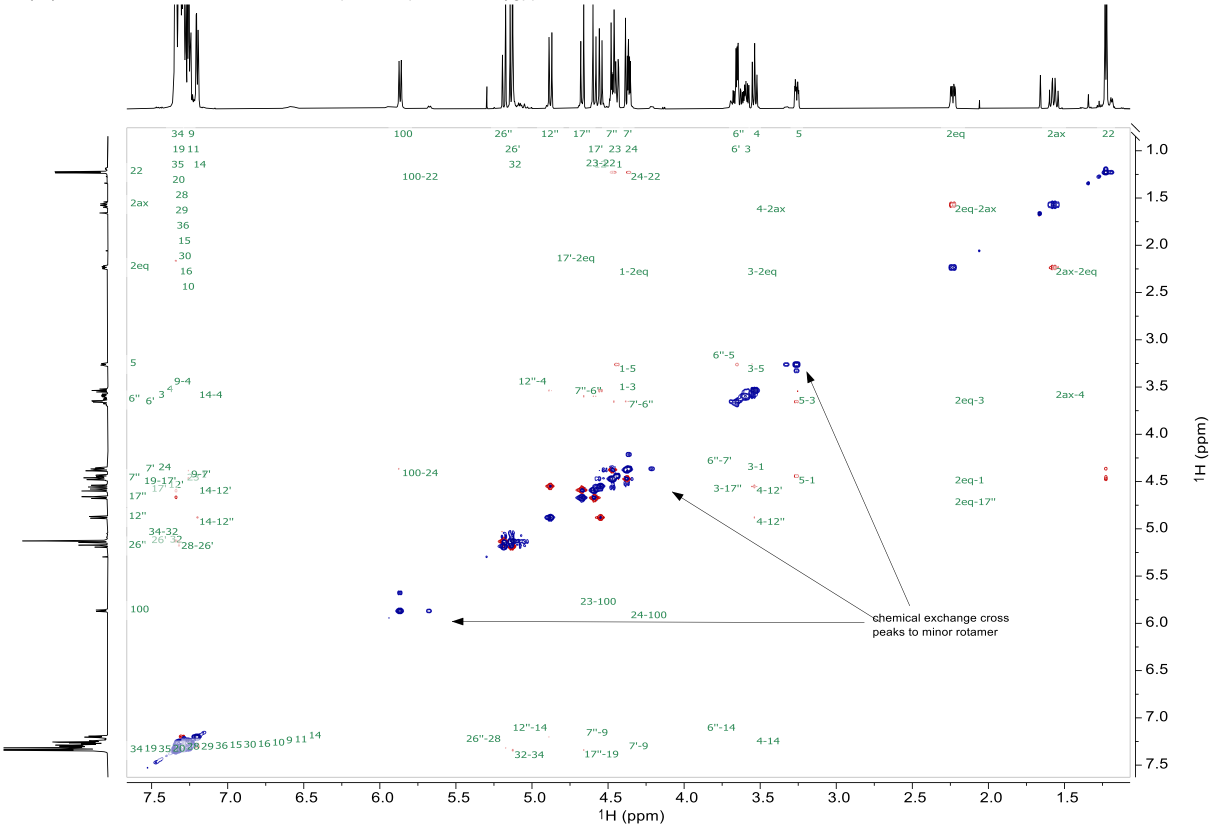

1H{15N},HMBC, 600.22 MHz,CDCl3,298.0K, pulse sequence: hmbcgpndqf

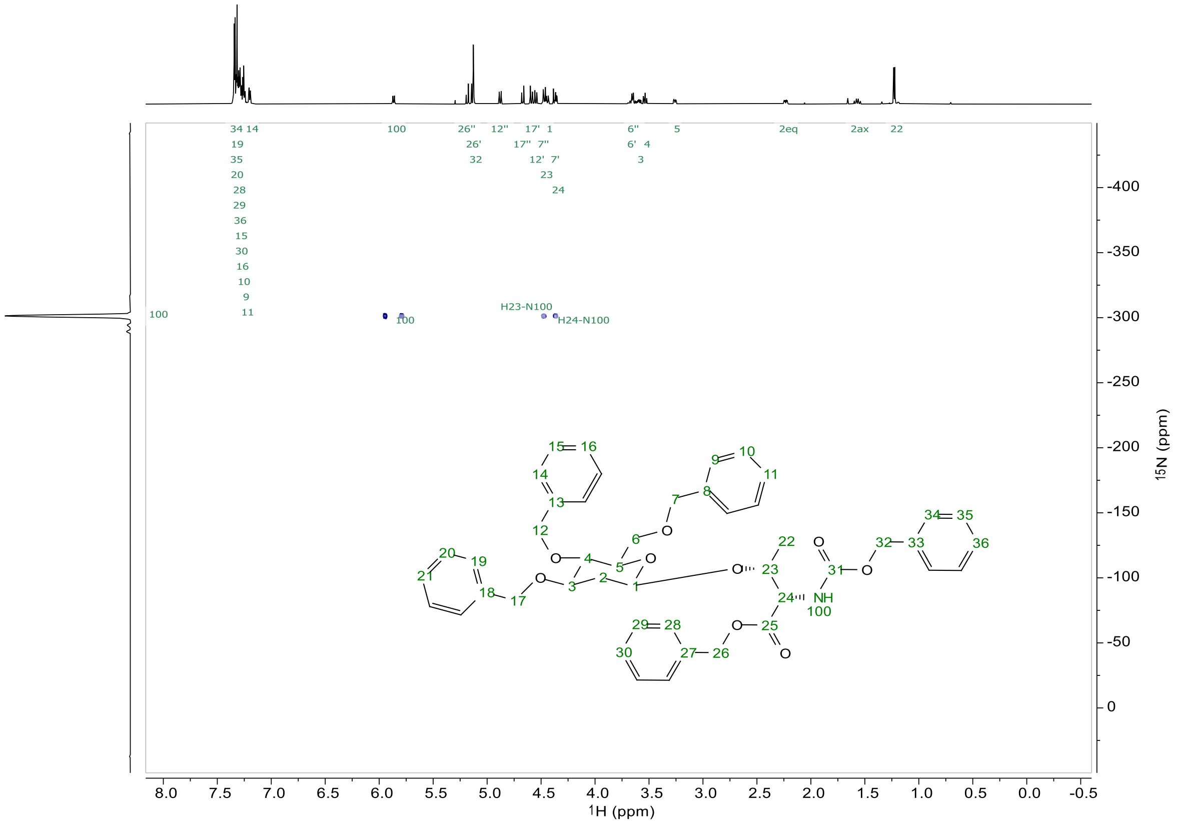

1H{13C},HSQC, 600.22 MHz,CDCl3,298.0K, pulse sequence: shsqcetgpsisp2.2

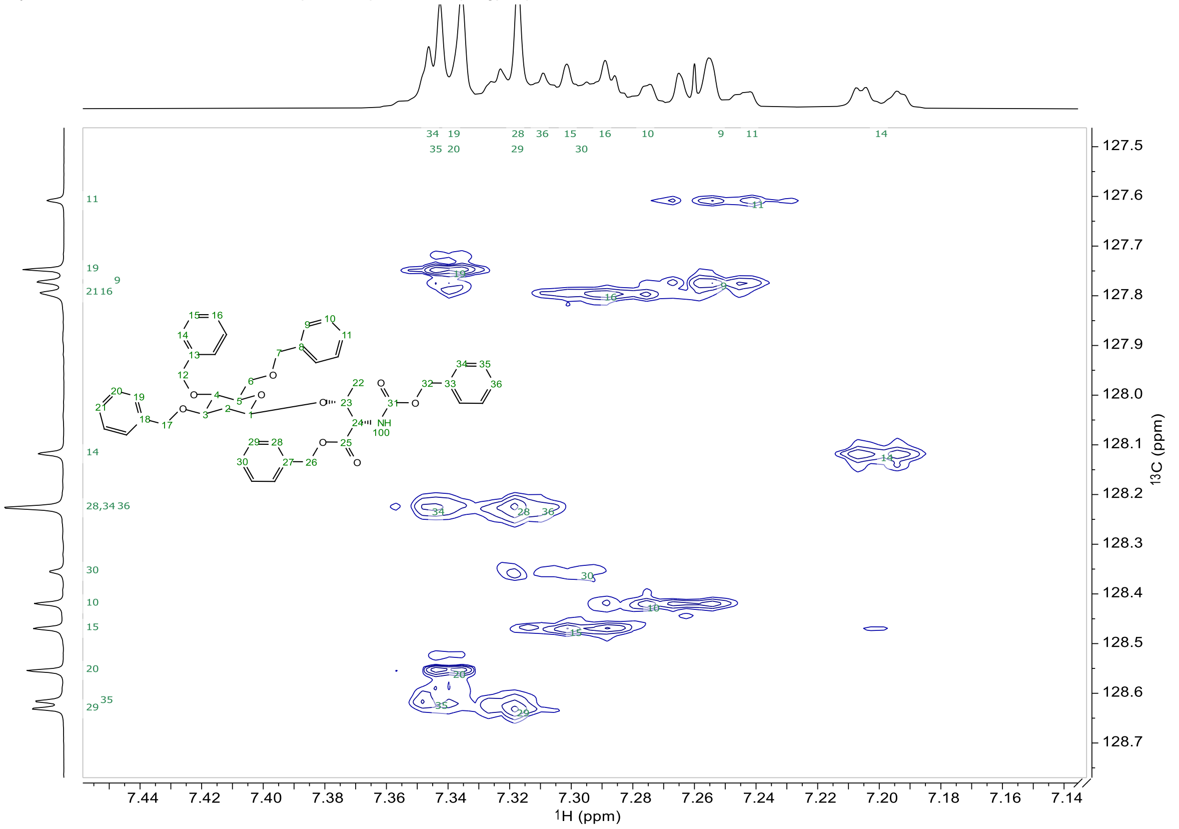

1H{13C},HMBC, 600.22 MHz,CDCl3,298.0K, pulse sequence: shmbcctetgpl2nd

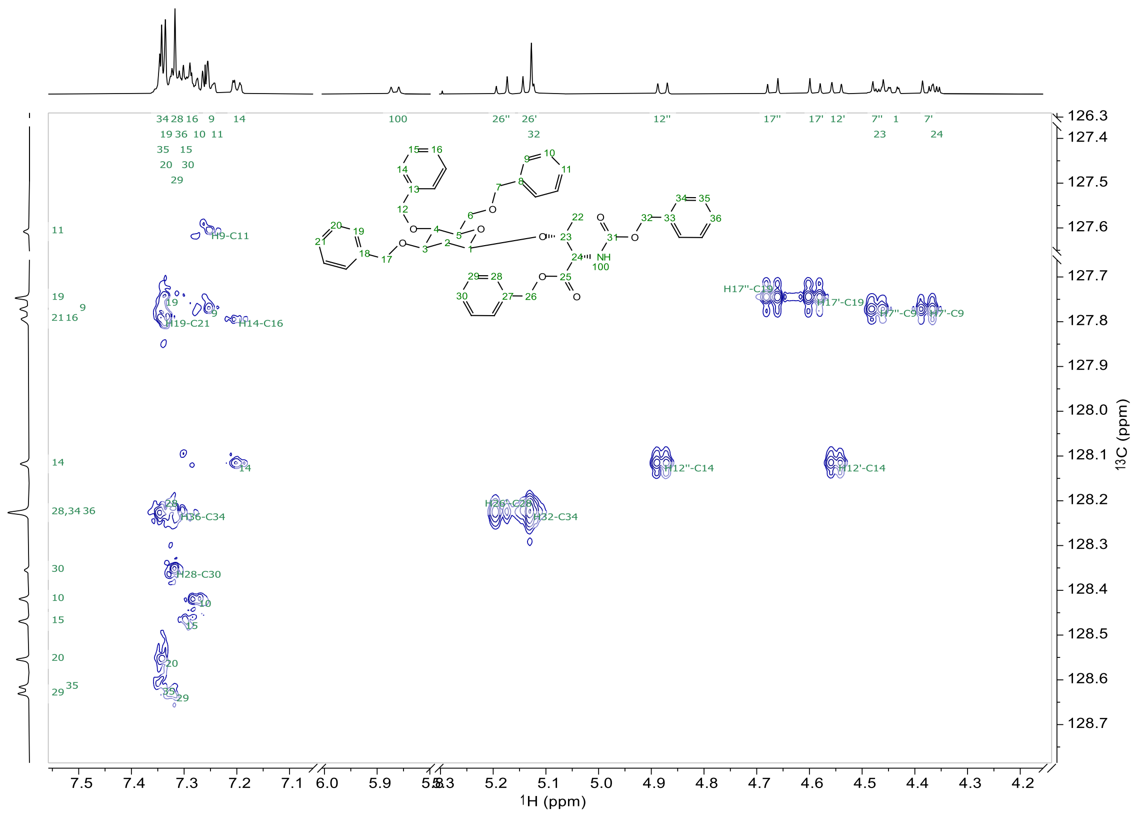

1H{off},1D, 600.23 MHz,CDCl3,298.0K, pulse sequence: seldigpzs

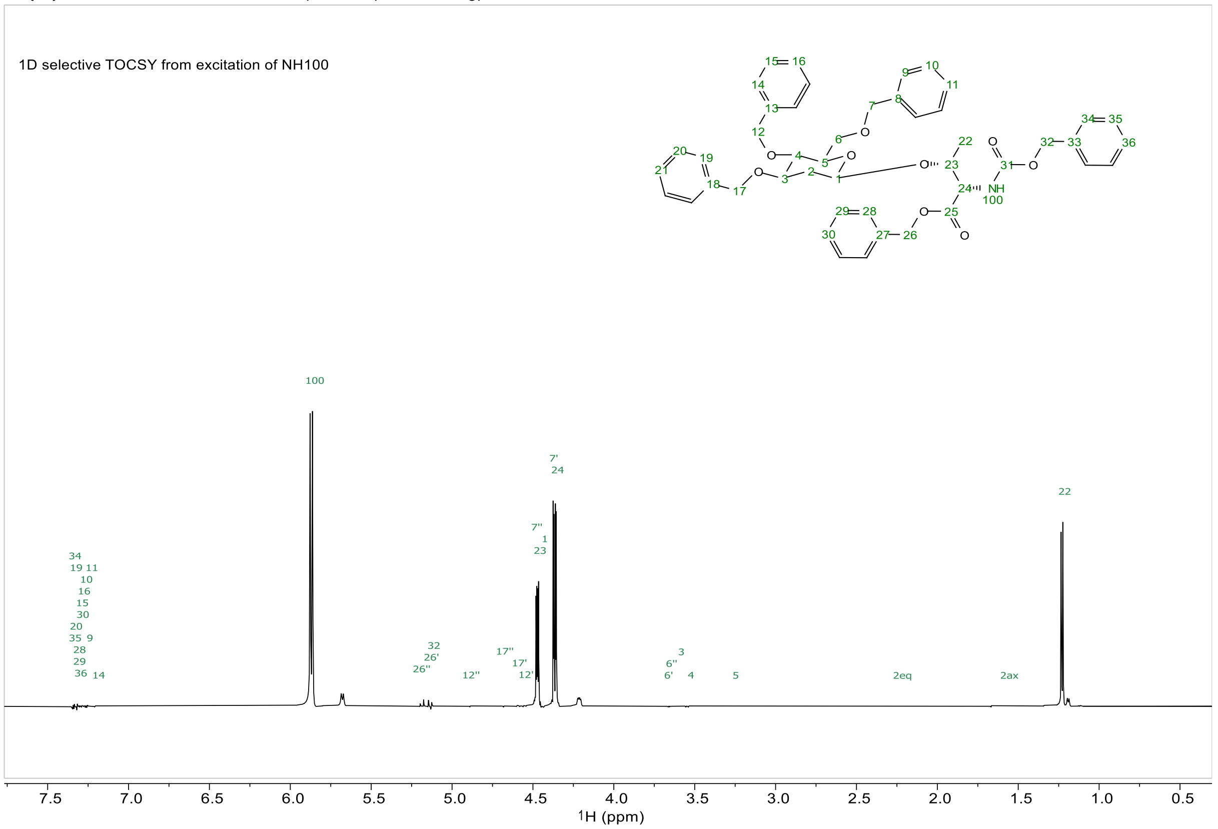

1H{off},1D, 600.23 MHz,CDCl3,298.0K, pulse sequence: seldigpzs

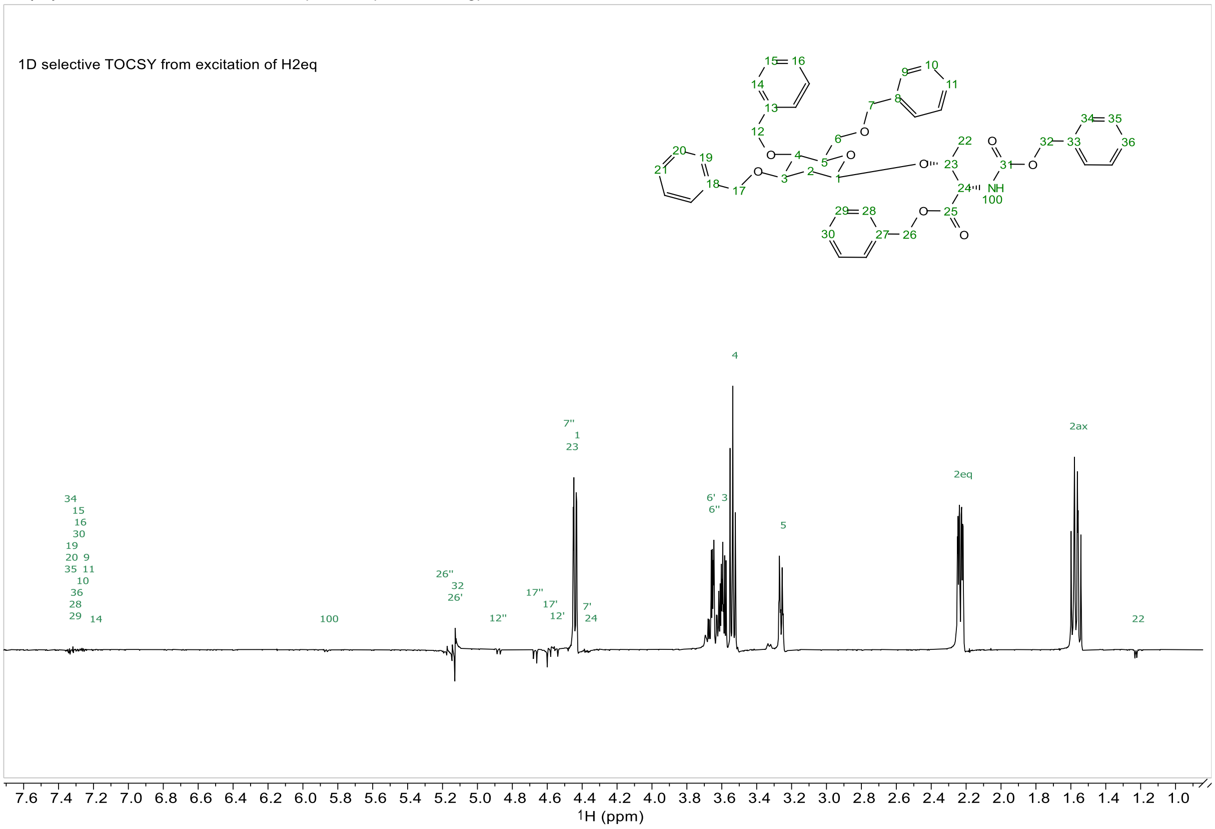

1H{off},1D, 600.23 MHz,CDCl3,298.0K, pulse sequence: seldigpzs

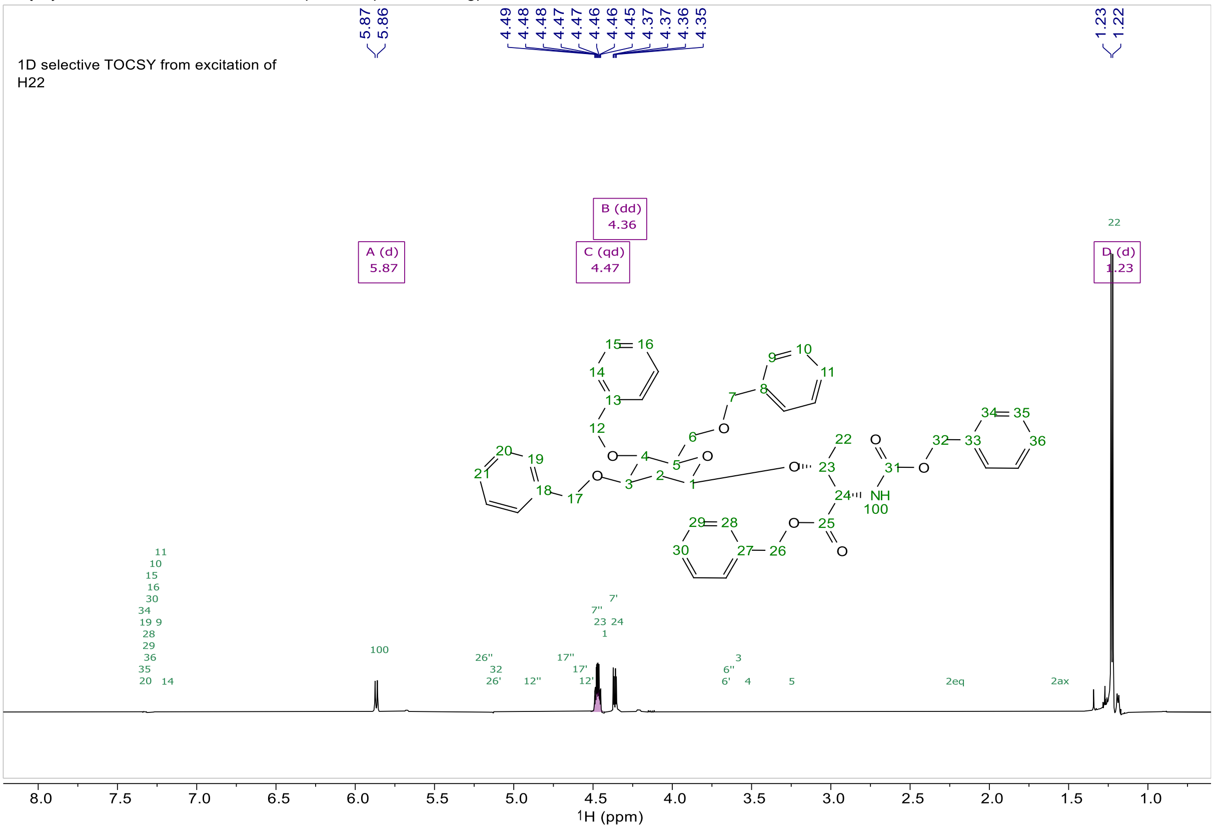

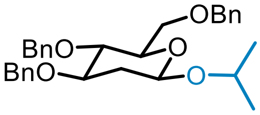

(2*R*,3*S*,4*R*,6*R*)-3,4-bis(benzyloxy)-2-((benzyloxy)methyl)-6-isopropoxytetrahydro-2*H*-pyran (4h)

Following **General Procedure B**, glycosyl donor **1a** (29 mg, 0.050 mmol) and alcohol **2h** (20 mg, 0.075 mmol) were coupled using (*R*,*R*)-IDPi **3a** (9.4 mg,10 mol%). Reaction time:48 h. β/α = 96:4 was determined by crude ^{1}H NMR. Flash chromatography (hexane/EtOAc = 10/1 to 8/1) gave **4h** as white solid (19 mg, 80%). The NMR data of this compound are consistent with those reported in the literature.[7]

Following **General Procedure A** on 0.01 mmol scale, the glycosylation was carried out using (*S*,*S*)-IDPi **3a**. β/α = 88:12 was determined by crude ^{1}H NMR.

TLC: R_f = 0.76 (Hexane/EtOAc = 3:1)

^{1}H NMR (501 MHz, $CDCl_3$) δ 1.08 (d, *J* = 6.1 Hz, 3H), 1.19 (d, *J* = 6.2 Hz, 3H), 1.53 – 1.63 (m, 1H), 2.23 (ddd, *J* = 12.6, 5.2, 2.0 Hz, 1H), 3.24 – 3.37 (m, 1H), 3.40 (dd, *J* = 9.7, 8.4 Hz, 1H), 3.52 – 3.64 (m, 2H), 3.68 (dd, *J* = 10.7, 2.0 Hz, 1H), 3.89 – 4.00 (m, 1H), 4.39 – 4.58 (m, 5H), 4.61 (d, *J* = 11.7 Hz, 1H), 4.82 (d, *J* = 10.9 Hz, 1H), 7.07 – 7.16 (m, 2H), 7.17 – 7.29 (m, 13H).

^{13}C NMR (126 MHz, $CDCl_3$) δ 21.99, 23.73, 37.38, 69.68, 70.87, 71.45, 73.56, 75.10, 75.33, 78.37, 79.79, 98.02, 127.64, 127.78, 127.80, 127.83, 127.87, 128.18, 128.46, 128.49, 128.57, 138.54, 138.56, 138.58.

$[\alpha]_D^{22}$ = -8.6 ($CHCl_3$, *c* = 1.0).

HRMS m/z (ESI): calcd. for C30 H36 Na1 O5 ([M+Na]$^+$): 499.24549; found: 499.24545.

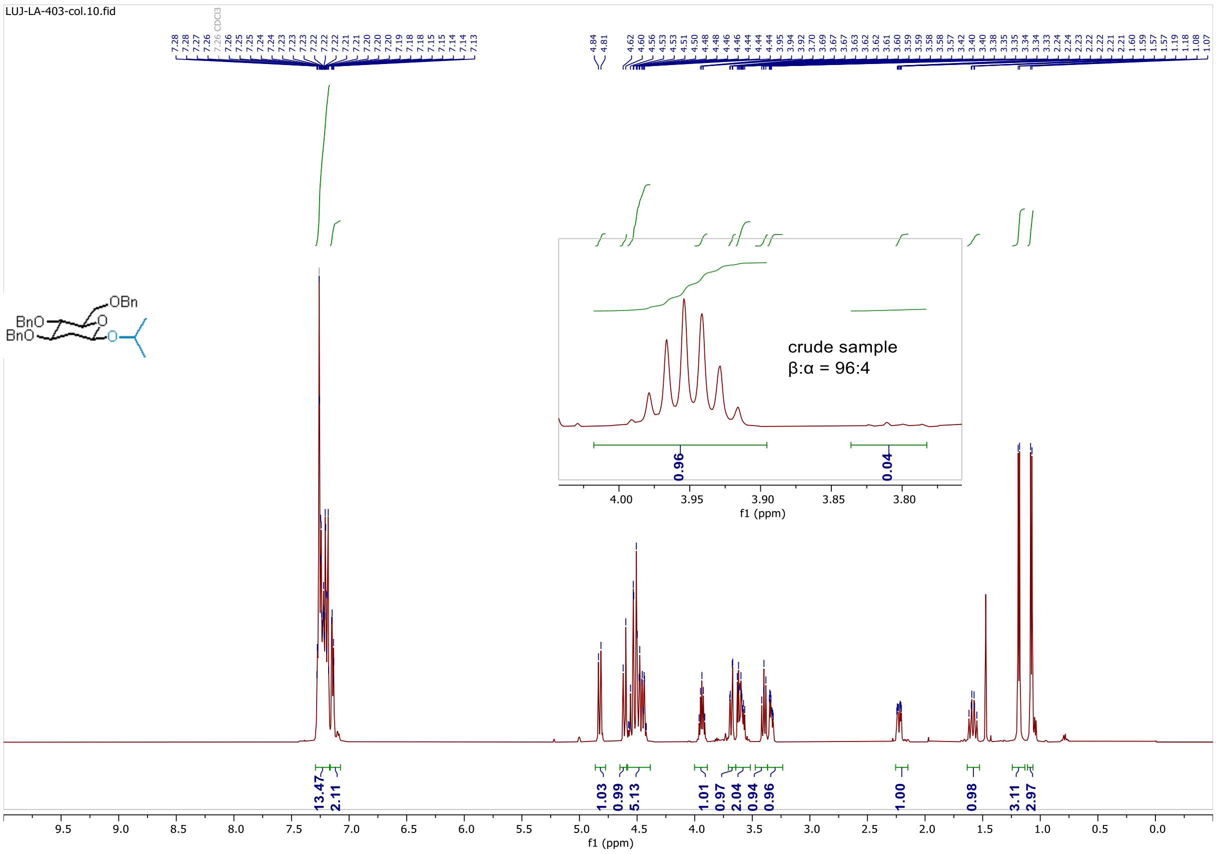

LUJ-LA-403-col.10.fid
7.28 7.28 7.27 7.26 7.26 CDCl3 7.26 7.25 7.24 7.24 7.23 7.23 7.22 7.22 7.22 7.21 7.21 7.20 7.20 7.20 7.19 7.18 7.18 7.15 7.14 7.14 7.13
4.84 4.81 4.62 4.60 4.56 4.53 4.53 4.51 4.50 4.48 4.48 4.46 4.46 4.44 4.44 4.44 3.95 3.94 3.92 3.70 3.69 3.67 3.67 3.63 3.62 3.62 3.61 3.60 3.59 3.59 3.58 3.58 3.57 3.42 3.40 3.38 3.35 3.35 3.34 3.34 3.33 2.24 2.24 2.23 2.22 2.22 2.22 2.21 1.60 1.59 1.57 1.57 1.19 1.18 1.08 1.07
OBn
BnO
BnO
O
O
crude sample
β:α = 96:4
0.96
0.04
4.00 3.95 3.90 3.85 3.80
f1 (ppm)
13.47 2.11 1.03 0.99 5.13 1.01 0.97 2.04 0.94 0.96 1.00 0.98 3.11 2.97
9.5 9.0 8.5 8.0 7.5 7.0 6.5 6.0 5.5 5.0 4.5 4.0 3.5 3.0 2.5 2.0 1.5 1.0 0.5 0.0
f1 (ppm)

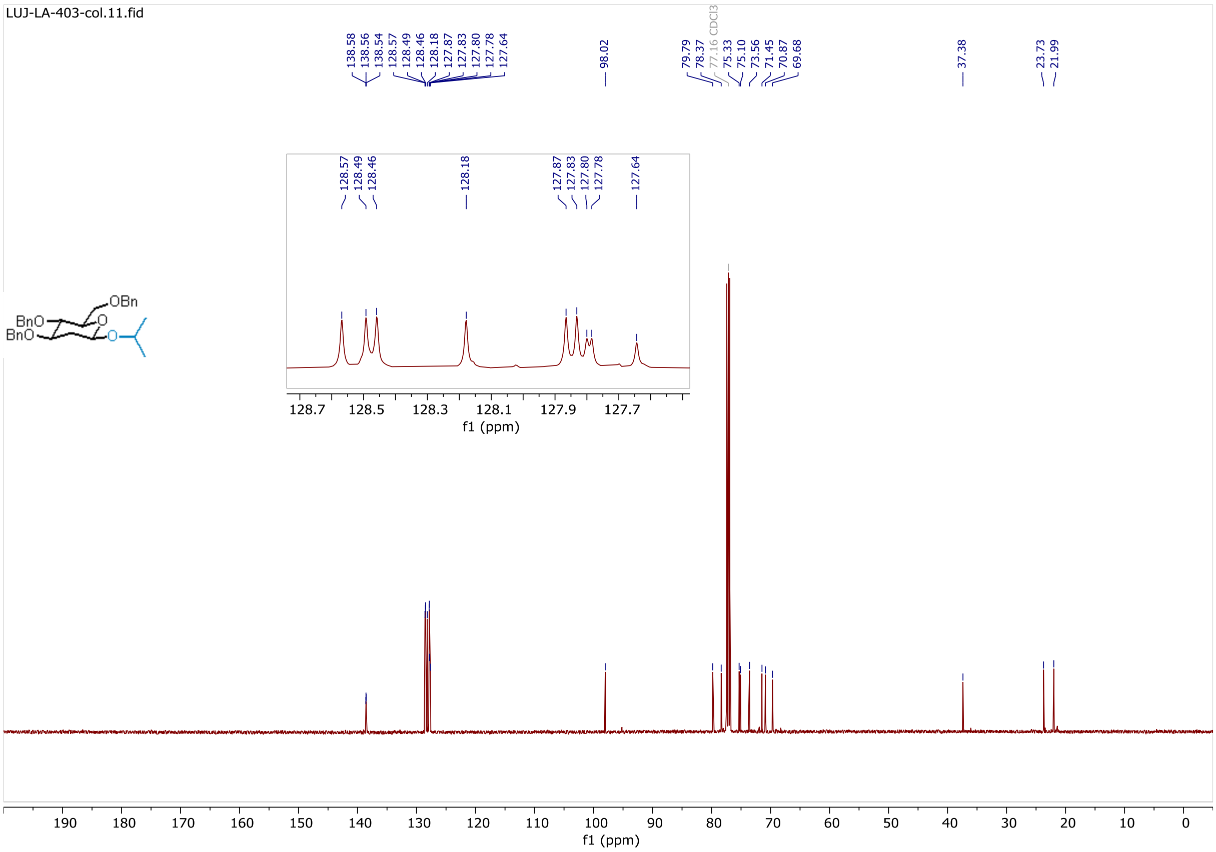

LUJ-LA-403-col.11.fid
138.58 138.56 138.54 128.57 128.49 128.46 128.18 127.87 127.83 127.80 127.78 127.64
98.02
79.79 78.37 77.16 CDCl3 75.33 75.10 73.56 71.45 70.87 69.68
37.38
23.73 21.99
128.57 128.49 128.46 128.18 127.87 127.83 127.80 127.78 127.64
128.7 128.5 128.3 128.1 127.9 127.7
f1 (ppm)
OBn
BnO
BnO
O
O
190 180 170 160 150 140 130 120 110 100 90 80 70 60 50 40 30 20 10 0
f1 (ppm)

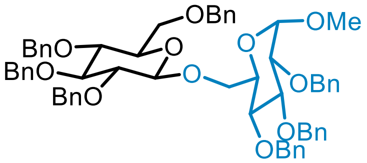

(2*R*,3*R*,4*S*,5*R*,6*R*)-3,4,5-tris(benzyloxy)-2-((benzyloxy)methyl)-6-(((2*R*,3*R*,4*S*,5*R*,6*S*)-3,4,5-tris(benzyloxy)-6-methoxytetrahydro-2*H*-pyran-2-yl)methoxy)tetrahydro-2*H*-pyran (5a)

Following **General Procedure B**, glycosyl donor **1b** (34 mg, 0.050 mmol) and alcohol **2a** (35 mg, 0.075 mmol) were coupled using (*R*,*R*)-IDPi **3b** (8.2 mg,10 mol%). Reaction time: 24 h. β/α = 94:6 was determined by crude ^{1}H NMR. Flash chromatography (hexane/EtOAc = 6/1 to 3/1) gave **5a** as white solid (46 mg, 92%). The NMR data of this compound are consistent with those reported in the literature.[4]

Following **General Procedure A** on 0.01 mmol scale, the glycosylation was carried out using (*S*,*S*)-IDPi **3b**. β/α > 95:5 was determined by crude ^{1}H NMR.

TLC: R_f = 0.33 (Hexane/EtOAc = 3:1)

^{1}H NMR (501 MHz, $CDCl_3$) δ 3.27 (s, 3H), 3.35 – 3.41 (m, 1H), 3.41 – 3.49 (m, 3H), 3.51 (t, *J* = 9.2 Hz, 1H), 3.54 – 3.64 (m, 3H), 3.67 (dd, *J* = 10.9, 2.0 Hz, 1H), 3.77 (ddd, *J* = 10.2, 4.8, 2.1 Hz, 1H), 3.93 (t, *J* = 9.3 Hz, 1H), 4.12 (dd, *J* = 10.8, 2.0 Hz, 1H), 4.29 (d, *J* = 7.8 Hz, 1H), 4.43 – 4.50 (m, 3H), 4.52 – 4.57 (m, 2H), 4.60 (d, *J* = 12.2 Hz, 1H), 4.63 – 4.77 (m, 6H), 4.85 (d, *J* = 10.8 Hz, 1H), 4.91 (dd, *J* = 11.0, 4.6 Hz, 2H), 7.05 – 7.16 (m, 7H), 7.17 – 7.31 (m, 29H).

^{13}C NMR (126 MHz, $CDCl_3$) *δ* 55.33, 68.67, 69.11, 69.97, 73.49, 73.55, 75.00, 75.02, 75.12, 75.15, 75.80, 75.84, 78.02, 78.12, 79.91, 82.09, 82.19, 84.91, 98.17, 103.91, 127.65, 127.67, 127.69, 127.72, 127.74, 127.79, 127.81, 127.89, 127.99, 128.01, 128.05, 128.08, 128.10, 128.28, 128.46, 128.47, 128.50, 128.52, 128.58, 138.22, 138.26, 138.34, 138.46, 138.49, 138.64, 138.96.

$[\alpha]_D^{22}$ = +9.4 ($CHCl_3$, *c* = 1.0).

HRMS m/z (ESI): calcd. for C62 H66 O11 Na1 ($[M+Na]^+$): 1009.449734; found: 1009.450320.

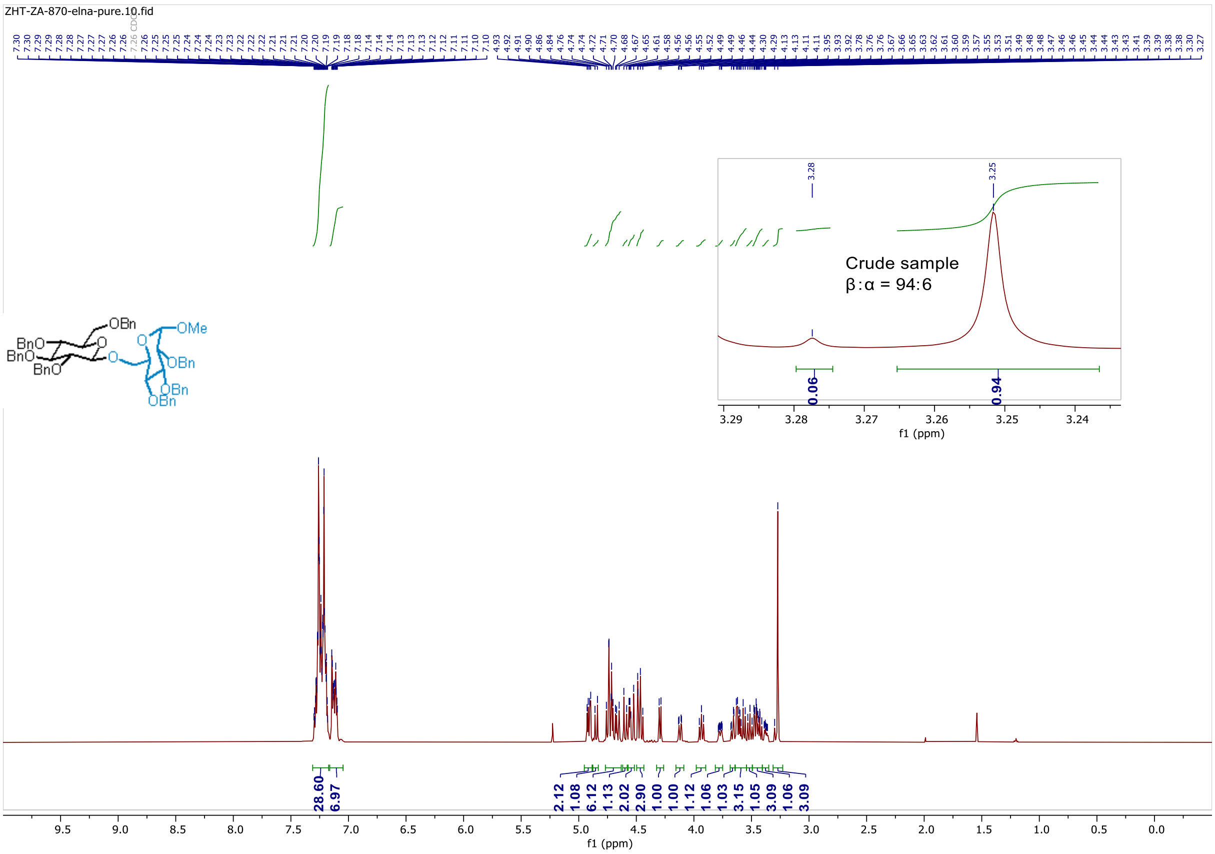
ZHT-ZA-870-elna-pure.10.fid
BnO
BnO
BnO
OBn
O
O
O
OMe
OBn
OBn
OBn
Crude sample
β:α = 94:6
f1 (ppm)

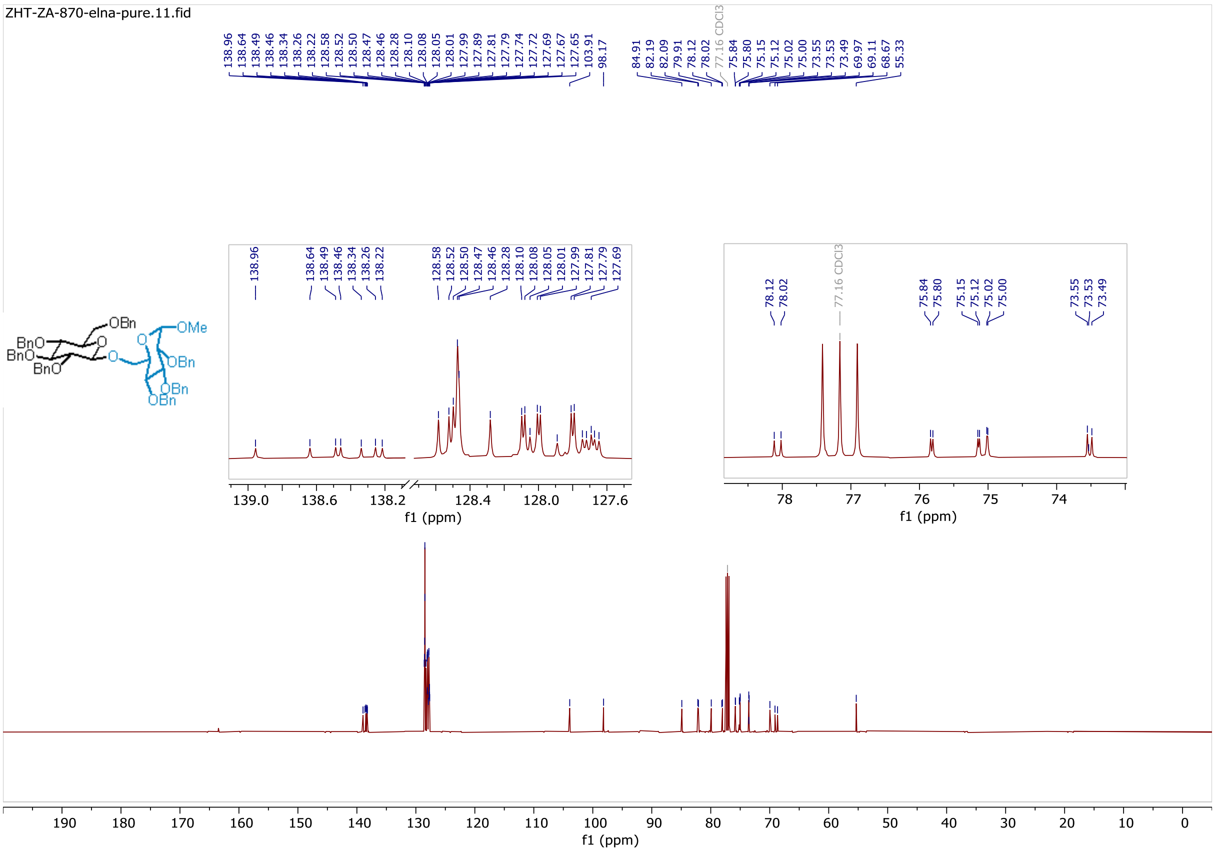
ZHT-ZA-870-elna-pure.11.fid
BnO
BnO
BnO
OBn
O
O
O
OMe
OBn
OBn
OBn
f1 (ppm)

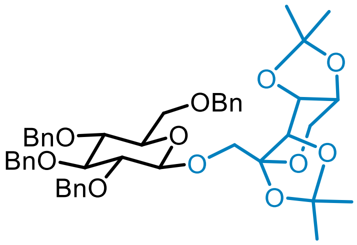

(3a*R*,5a*S*,8a*S*,8b*R*)-2,2,7,7-tetramethyl-3a-((((2*R*,3*R*,4*S*,5*R*,6*R*)-3,4,5-tris(benzyloxy)-6-((benzyloxy)methyl)tetrahydro-2*H*-pyran-2-yl)oxy)methyl)tetrahydro-5*H*-bis([1,3]dioxolo)[4,5-b:4',5'-d]pyran (5b)

Following **General Procedure B**, glycosyl donor **1b** (34 mg, 0.050 mmol) and alcohol **2b** (20 mg, 0.075 mmol) were coupled using (*R*,*R*)-IDPi **3b** (8.2 mg,10 mol%). Reaction time: 24 h. β/α = 93:7 was determined by crude ^{1}H NMR. Flash chromatography (hexane/EtOAc = 6/1 to 3/1) gave **5b** as colorless oil (34 mg, 87%). The NMR data of this compound are consistent with those reported in the literature.[8]

Following **General Procedure A** on 0.01 mmol scale, the glycosylation was carried out using (*S*,*S*)-IDPi **3b**. β/α > 95:5 was determined by crude ^{1}H NMR.

TLC: R_f = 0.35 (Hexane/EtOAc = 3:1)

^{1}H NMR (501 MHz, CDCl$_3$) δ 1.23 (s, 3H), 1.34 (s, 3H), 1.37 (s, 3H), 1.45 (s, 3H), 3.36 (ddd, *J* = 9.5, 4.2, 1.7 Hz, 1H), 3.41 (ddd, *J* = 9.0, 6.1, 3.0 Hz, 1H), 3.54 – 3.62 (m, 2H), 3.62 – 3.72 (m, 3H), 3.81 – 3.92 (m, 3H), 4.15 (dd, *J* = 7.8, 1.6 Hz, 1H), 4.41 – 4.45 (m, 2H), 4.45 – 4.54 (m, 3H), 4.56 – 4.63 (m, 2H), 4.73 (dd, *J* = 13.0, 10.9 Hz, 2H), 4.84 (d, *J* = 10.9 Hz, 1H), 4.93 (d, *J* = 10.7 Hz, 1H), 7.08 – 7.14 (m, 2H), 7.17 – 7.31 (m, 18H).

^{13}C NMR (126 MHz, CDCl$_3$) *δ* 24.23, 25.86, 26.11, 26.79, 61.26, 69.01, 70.21, 70.34, 70.38, 71.16, 73.81, 75.04, 75.09, 75.23, 75.82, 77.93, 82.66, 84.91, 102.32, 103.36, 108.81, 109.04, 127.63, 127.71, 127.77, 127.86, 127.97, 128.10, 128.39, 128.48, 128.50, 128.52, 138.33, 138.59, 138.62, 138.78.

$[\alpha]_D^{22}$ = -7.8 (CHCl$_3$, *c* = 1.0).

HRMS m/z (ESI): calcd. for C46 H54 Na1 O11 ([M+Na]$^+$): 805.35583; found: 805.35539.

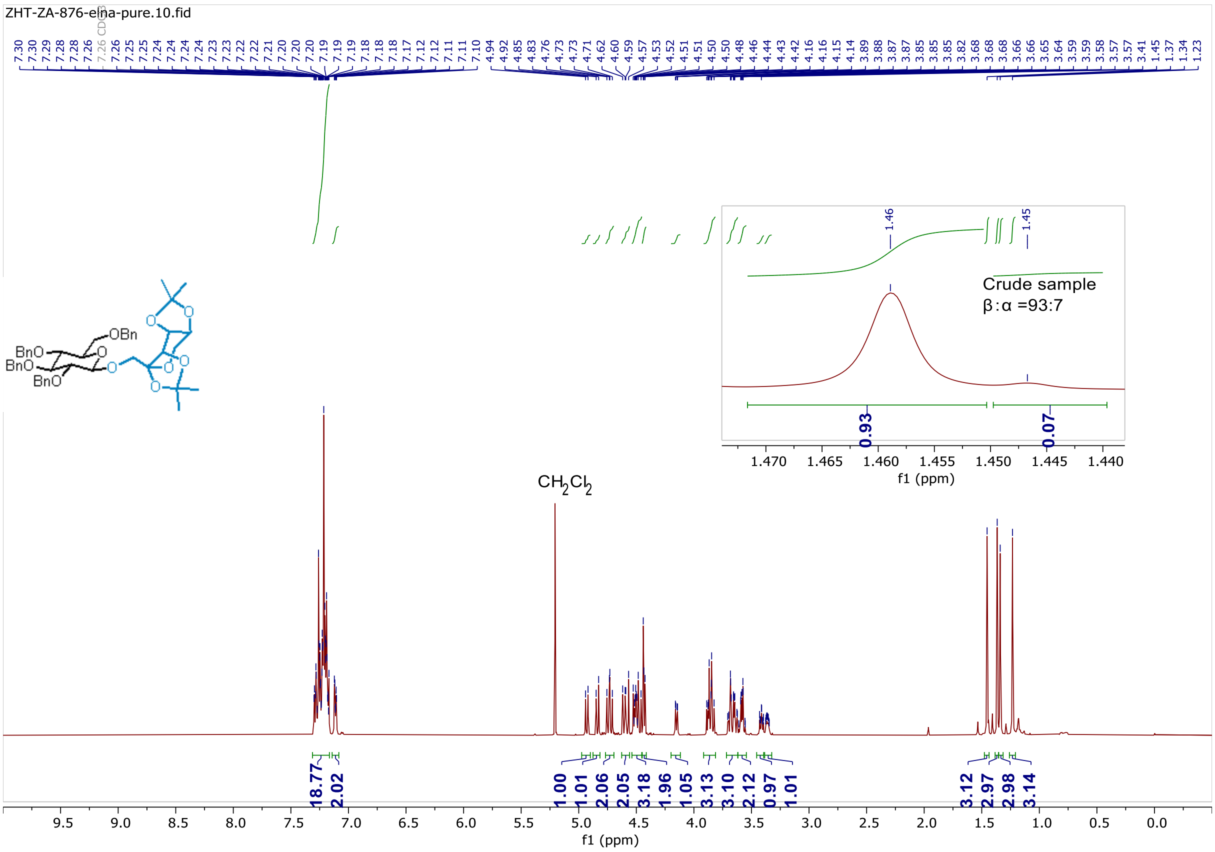

ZHT-ZA-876-elna-pure.10.fid
Crude sample
β:α =93:7
CH2Cl2
f1 (ppm)

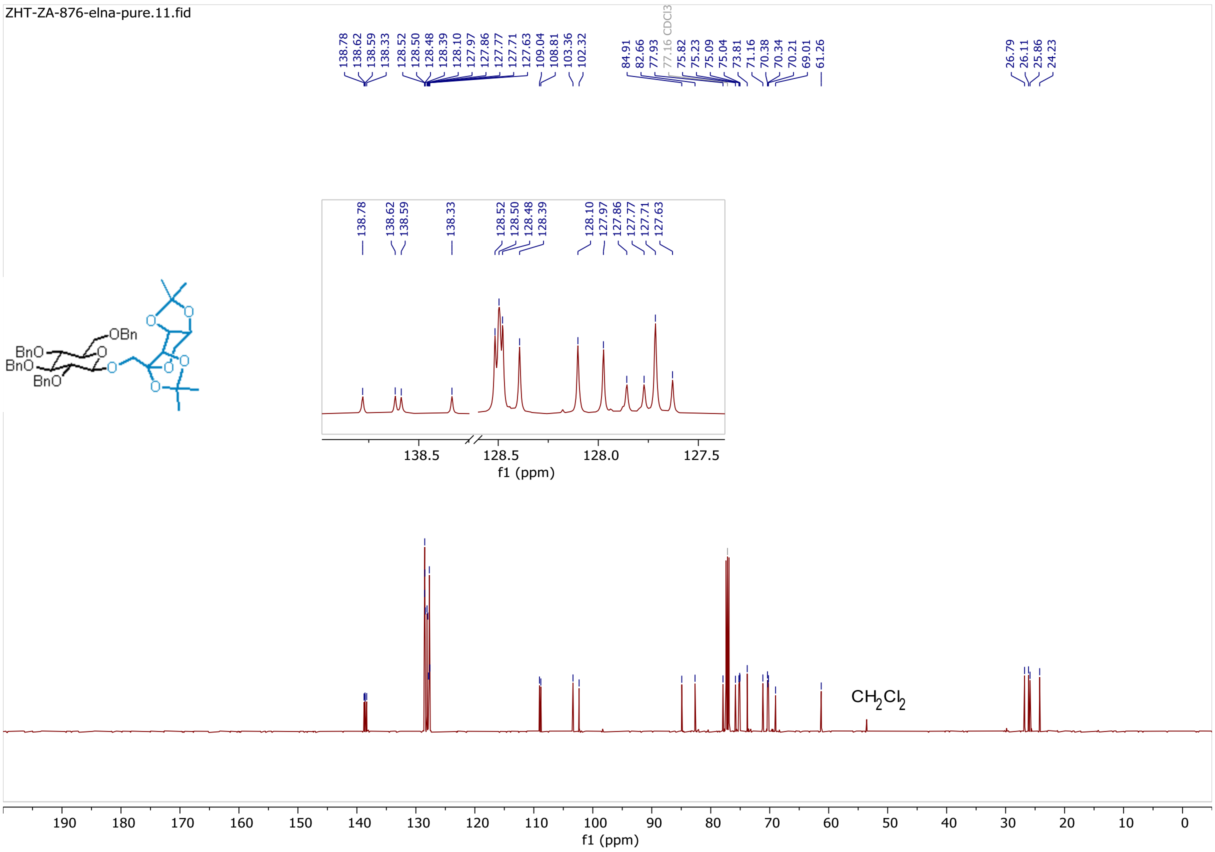

ZHT-ZA-876-elna-pure.11.fid
CH2Cl2
f1 (ppm)

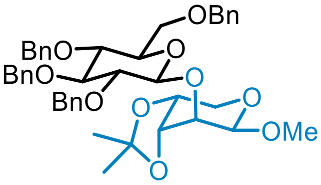

(3a*R*,6*R*,7*S*,7a*R*)-6-methoxy-2,2-dimethyl-7-(((2*S*,3*R*,4*S*,5*R*,6*R*)-3,4,5-tris(benzyloxy)-6-((benzyloxy)methyl)tetrahydro-2*H*-pyran-2-yl)oxy)tetrahydro-4*H*-[1,3]dioxolo[4,5-c]pyran (5c)

Following **General Procedure B**, glycosyl donor **1b** (34 mg, 0.050 mmol) and alcohol **2c** (15 mg, 0.075 mmol) were coupled using (*R*,*R*)-IDPi **3b** (8.2 mg,10 mol%). Reaction time: 72 h. β/α >95:5 was determined by crude ^{1}H NMR. Flash chromatography (hexane/EtOAc = 6/1 to 3/1) gave **5c** as light yellow foam (31 mg, 84%).

Following **General Procedure A** on 0.01 mmol scale, the glycosylation was carried out using (*S*,*S*)-IDPi **3b**. β/α > 95:5 was determined by crude ^{1}H NMR.

TLC: R_f = 0.25 (Hexane/EtOAc = 3:1)

^{1}H NMR (600 MHz, $CDCl_3$) δ 1.36 (s, 3H), 1.54 (s, 3H), 3.34 (s, 3H), 3.45 (dt, *J* = 9.6, 3.5 Hz, 1H), 3.55 (t, *J* = 8.9 Hz, 1H), 3.59 (t, *J* = 9.2 Hz, 1H), 3.64 (t, *J* = 8.9 Hz, 1H), 3.64 – 3.71 (m, 2H), 3.93 (dd, *J* = 13.3, 2.8 Hz, 1H), 3.97 (dd, *J* = 13.3, 1.0 Hz, 1H), 4.00 (dd, *J* = 8.0, 3.1 Hz, 1H), 4.25 (ddd, *J* = 5.5, 2.8, 1.0 Hz, 1H), 4.37 (dd, *J* = 8.0, 5.5 Hz, 1H), 4.51 (d, *J* = 12.2 Hz, 1H), 4.52 (d, *J* = 7.8 Hz, 0H), 4.53 (d, *J* = 10.8 Hz, 1H), 4.55 (d, *J* = 12.2 Hz, 1H), 4.72 (d, *J* = 11.0 Hz, 1H), 4.78 (d, *J* = 10.9 Hz, 1H), 4.81 (d, *J* = 10.8 Hz, 1H), 4.83 (d, *J* = 3.1 Hz, 1H), 4.92 (d, *J* = 10.9 Hz, 1H), 5.07 (d, *J* = 11.0 Hz, 1H), 7.13 – 7.18 (m, 2H), 7.26 – 7.34 (m, 14H), 7.36 – 7.40 (m, 2H).

^{13}C NMR (151 MHz, $CDCl_3$) δ 26.45, 28.18, 55.72, 59.14, 69.25, 73.60, 73.67, 74.04, 74.68, 75.09, 75.14, 75.83, 75.88, 77.84, 81.90, 84.88, 97.53, 101.74, 109.11, 127.68, 127.70, 127.89, 127.95, 128.00, 128.19, 128.24, 128.42, 128.46, 128.47, 128.51, 138.17, 138.23, 138.75, 138.78.

$[\alpha]_D^{22}$ = -38.2 ($CHCl_3$, *c* = 1.0).

HRMS m/z (ESI): calcd. for C43 H50 Na1 O10 ($[M+Na]^+$): 749.32962; found: 749.32924.

User Report
ZHT-ZA-844-01

NMR data supports the following structure

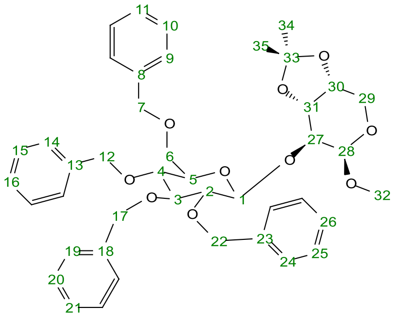

Remarks:
The relative stereochemistry at C1–C5 is supported by the observed ³J-values (³J > 7.8 Hz → dominantly *trans* in the presence of multiple oxygen atoms). Additional support comes from NOE correlations of H1 to H3 and H5, consistent with all three being in axial orientation as indicated in the structure above. While the relative stereochemistry within each ring is well supported by the data, the relative configuration between the rings could not be unambiguously confirmed and was therefore assumed as submitted.

An overview of all assignments is shown on the next page. The aromatic regions exhibit significant signal overlap. HIgh resolution, band-selective HSQC and HMBC experiments were used to support the assignments; however, some of the correlations remain ambiguous, and certain signals may be interchangeable.

P-ID: ML00001
Measured on: 31/10/2025
CHIFFRE:ZHT-ZA-884-01
ELNA#:15457
Client: Tianyu Zheng
Group: List
Spectroscopist: Leutzsch
Analysed on: 03/11/2025
Analysed by: Leutzsch
Amount: 30.0 mg
Solvent: CDCl3
Reference: 1H+13C on solvent,other nuclei w/ xiref
Temperature: 298 K
Spectrometer: av600a
Probe: cryoBBO
Experiments: 1H-zg30, 13C-zgpg30, 1H-13C-hsqcedetgpsisp2.3, 1H-13C-hmbcetgpl3nd, 1H-1H-cosygpppqf, 1H-1H-noesygpph, 1H-13C-shsqcetgpsisp2.2, 1H-13C-shmbcctetgpl2nd

Atom	δ (ppm)	J	COSY	HSQC	HMBC	NOESY
1 C	101.740			1	2, 5, 27	
H	4.515	7.80(2)	2	1	3, 5, 27	3, 5, 27
2 C	81.895			2	3, 22', 22"	
H	3.547	8.90(3), 7.80(1)	1, 3	2	1, 3, 22	4, 22', 22"
3 C	84.879			3	1, 2, 4, 5, 17', 17"	
H	3.640	8.80(4), 8.90(2)	2, 4	3	2, 4, 17	1, 17', 17"
4 C	77.836			4	3, 5, 6, 12', 12"	
H	3.594	8.80(3), 9.60(5)	3, 5	4	3, 5, 6	2, 12', 12"
5 C	75.085			5	1, 4, 6	
H	3.450	3.50(6), 9.60(4)	4, 6	5	1, 3, 4, 6	1, 6
6 C	69.250			6	4, 5, 7', 7"	
H2	3.676	3.50(5)	5	6	4, 5, 7	5, 9, 14, 34
7 C	73.596			7', 7"	6, 9	
H'	4.507	12.20(7")	7"	7	6, 8, 9	9
H"	4.553	12.20(7')	7'	7	6, 8, 9	9
8 C	138.228				7', 7"	
9 C	127.949			9	7', 7", 9	
H	7.325			9	7, 9, 11	6, 7', 7", 34
10 C	128.458			10		
H	7.324			10		
11 C	127.697			11	9	
H	7.276			11		
12 C	75.137			12', 12"	14	
H'	4.531	10.80(12")	12", 14	12	4, 13, 14	4, 14, 34
H"	4.805	10.80(12')	12', 14	12	4, 13, 14	4, 14

Atom	δ (ppm)	J	COSY	HSQC	HMBC	NOESY
13 C	138.164				12', 12"	
14 C	128.189			14	12', 12", 14	
H	7.154		12', 12", 15	14	12, 14, 16	6, 12', 12"
15 C	128.513			15		
H	7.271		14	15		
16 C	127.890			16	14	
H	7.270			16		
17 C	75.821			17', 17"	3, 19	
H'	4.783	10.90(17")	17"	17	3, 18, 19	3, 19
H"	4.925	10.90(17')	17'	17	3, 18, 19	3, 19, 24
18 C	138.753				17', 17"	
19 C	127.998			19	17', 17", 19	
H	7.297			19	17, 19, 21	17', 17"
20 C	128.472			20		
H	7.302			20		
21 C	127.697			21	19	
H	7.276			21		
22 C	74.676			22', 22"	2, 24	
H'	4.717	11.00(22")	22", 24	22	2, 23, 24	2, 24
H"	5.070	11.00(22')	22', 24	22	2, 23, 24	2, 24
23 C	138.778				22', 22"	
24 C	128.234			24	22', 22", 24	
H	7.380		22', 22", 25	24	22, 24, 26	17", 22', 22", 32
25 C	128.415			25		
H	7.299		24	25		

Atom	δ (ppm)	J	COSY	HSQC	HMBC	NOESY
26 C	127.677			26	24	
H	7.271			26		
27 C	75.887			27	1, 28, 30, 31	
H	4.000	3.10(28), 8.00(31)	28, 31	27	1, 31	1, 28, 34
28 C	97.530			28	29', 29", 32	
H	4.829	3.10(27)	27	28	27, 29, 31, 32	27, 32
29 C	59.142			29', 29"	28, 30	
H'	3.927	13.30(29"), 2.70(30)	29", 30	29	28, 30	30, 32
H"	3.974	13.30(29'), 1.00(30)	29', 31	29	28, 30, 31	30
30 C	73.670			30	29', 29", 31	
H	4.246	2.70(29'), 1.00(29"), 5.50(31)	29', 31	30	27, 29, 31	29', 29", 31, 35
31 C	74.037			31	27, 28, 29", 30	
H	4.373	8.00(27), 5.50(30)	27, 29", 30, 34	31	27, 30, 33	30
32 C	55.720			32	28	
H3	3.339			32	28	24, 28, 29'
33 C	109.104				31, 34, 35	
34 C	28.182			34	35	
H3	1.539		31, 35	34	33, 35	6, 9, 12', 27, 35
35 C	26.447			35	34	
H3	1.356		34	35	33, 34	30, 34

1H{off},1D, 600.23 MHz,CDCl3,298.0K, pulse sequence: zg30

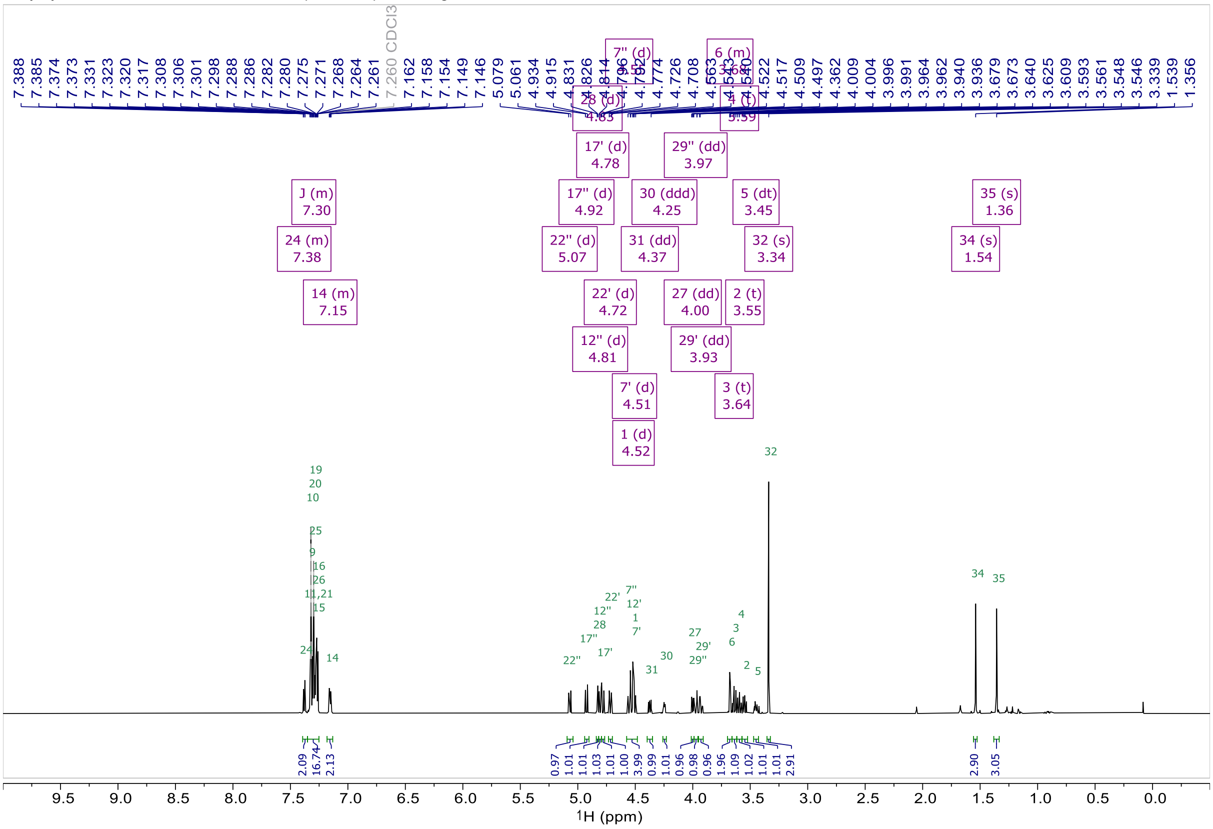

13C{1H},1D, 150.94 MHz,CDCl3,298.0K, pulse sequence: zgpg30

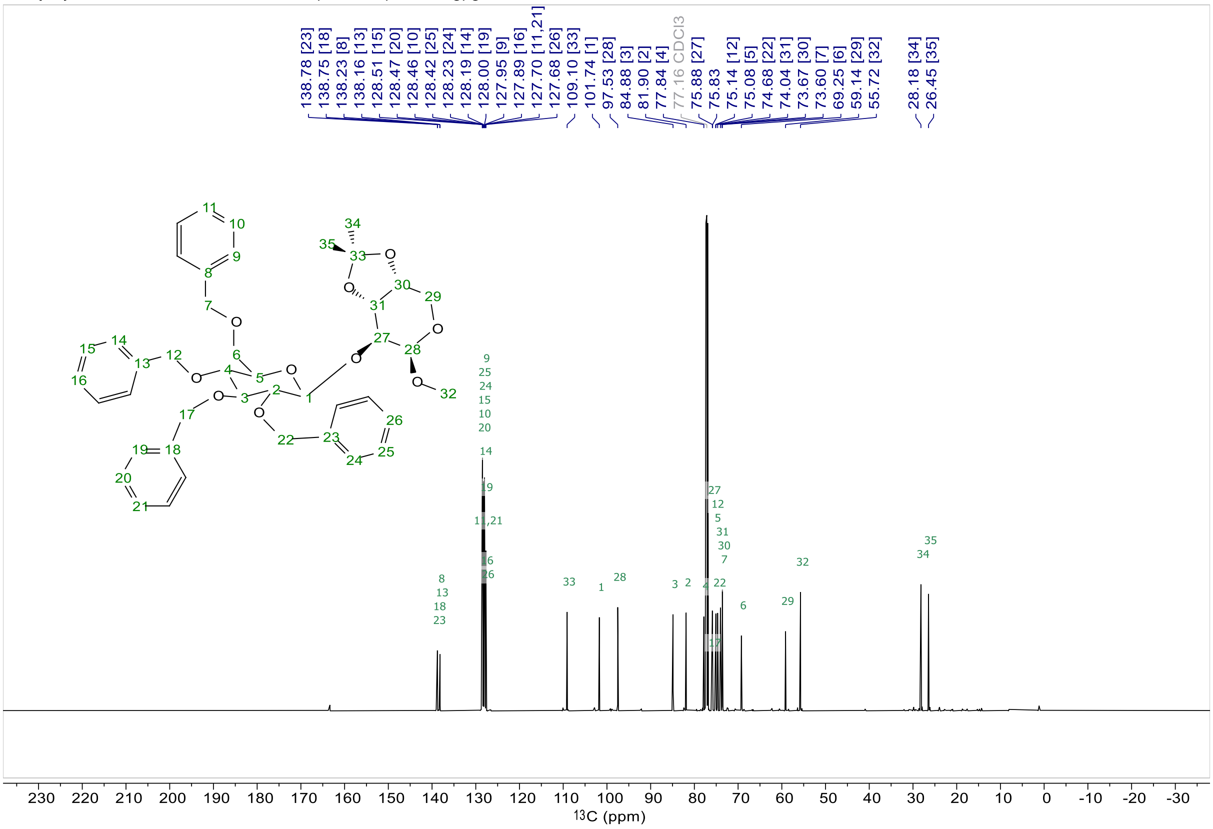

1H{13C},HSQC-EDITED, 600.22 MHz,CDCl3,298.0K, pulse sequence: hsqcedetgpsisp2.3

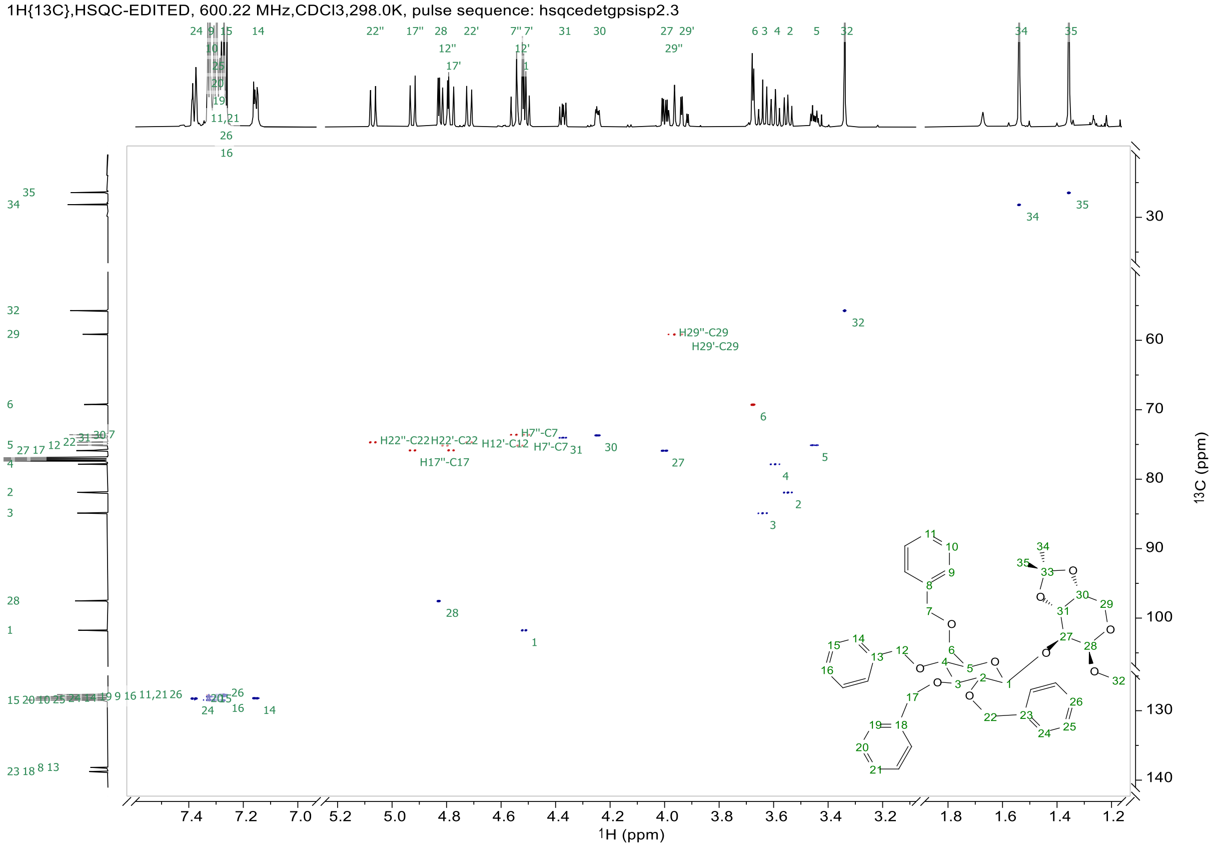

1H{13C},HMBC, 600.22 MHz,CDCl3,298.0K, pulse sequence: hmbcetgpl3nd

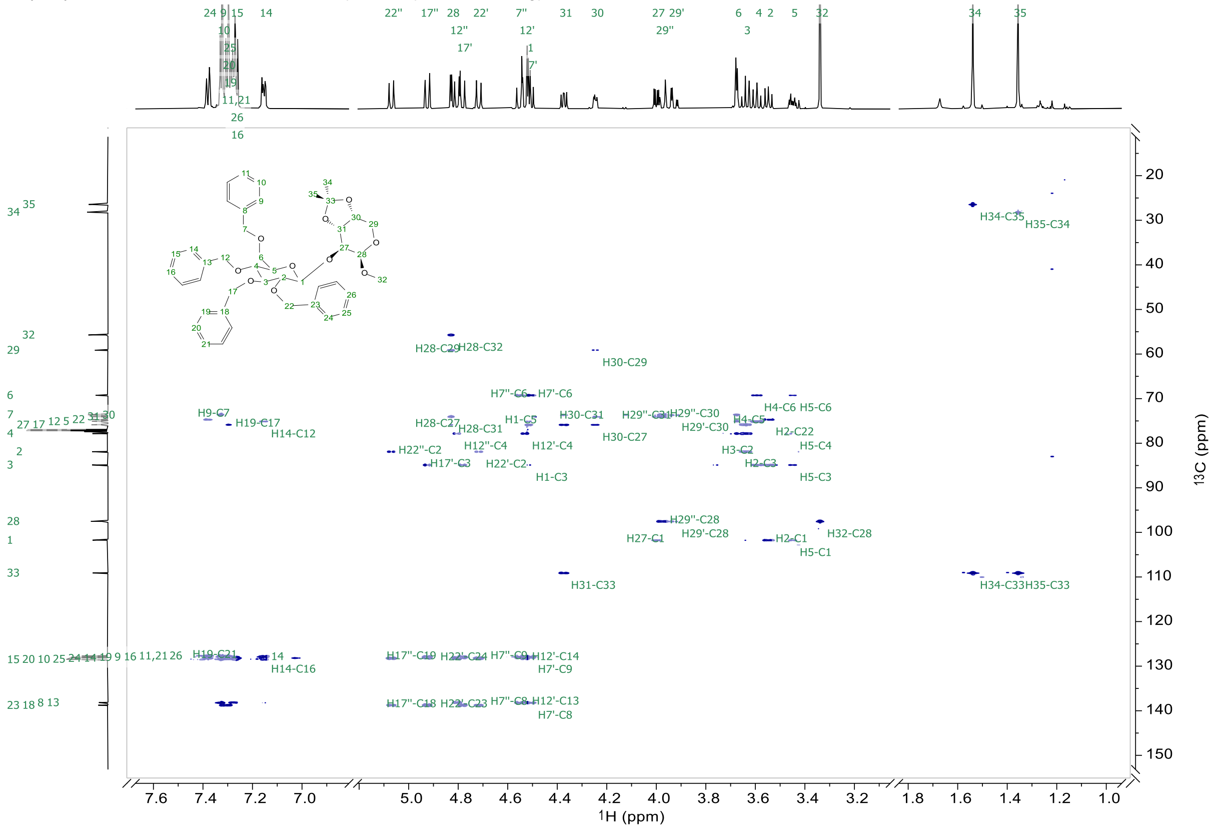

1H{off},COSY, 600.22 MHz,CDCl3,298.0K, pulse sequence: cosygpppqf

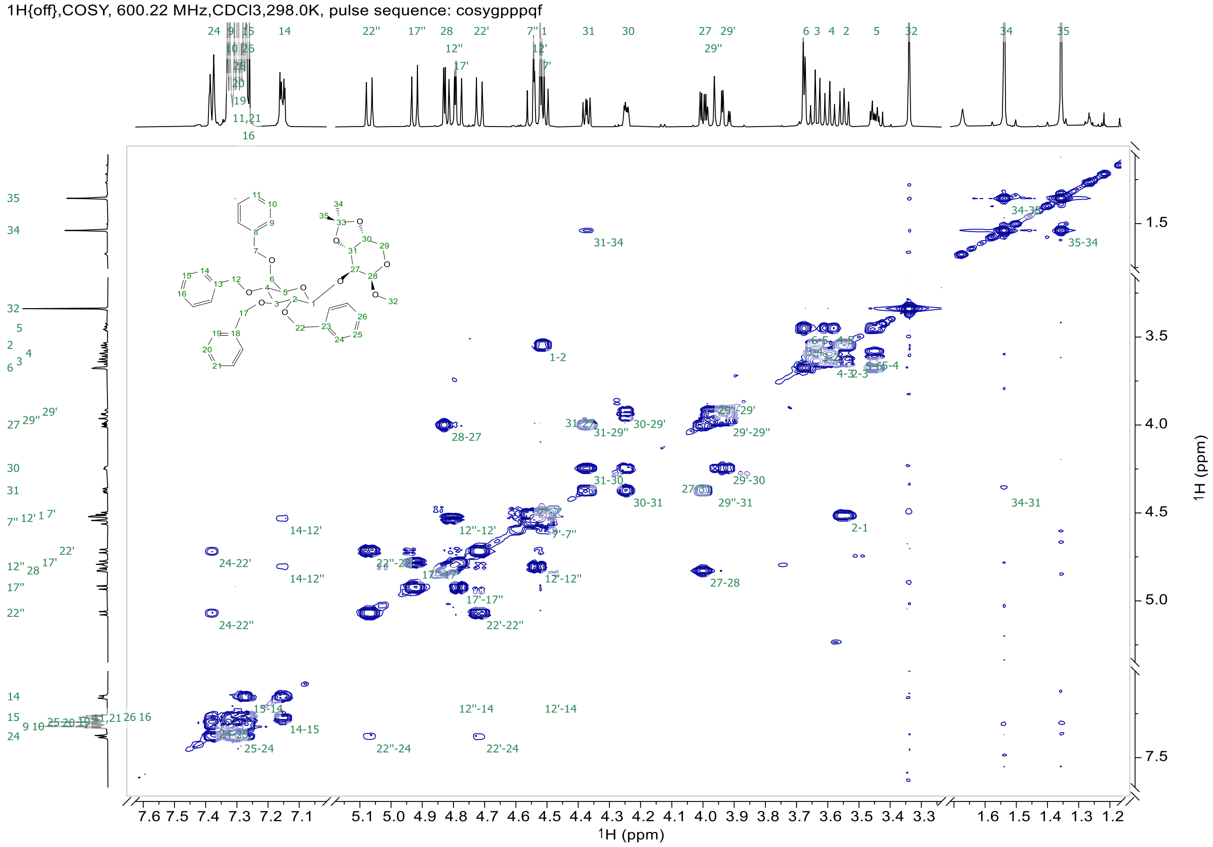

1H{off},NOESY, 600.22 MHz,CDCl3,298.0K, pulse sequence: noesygpph

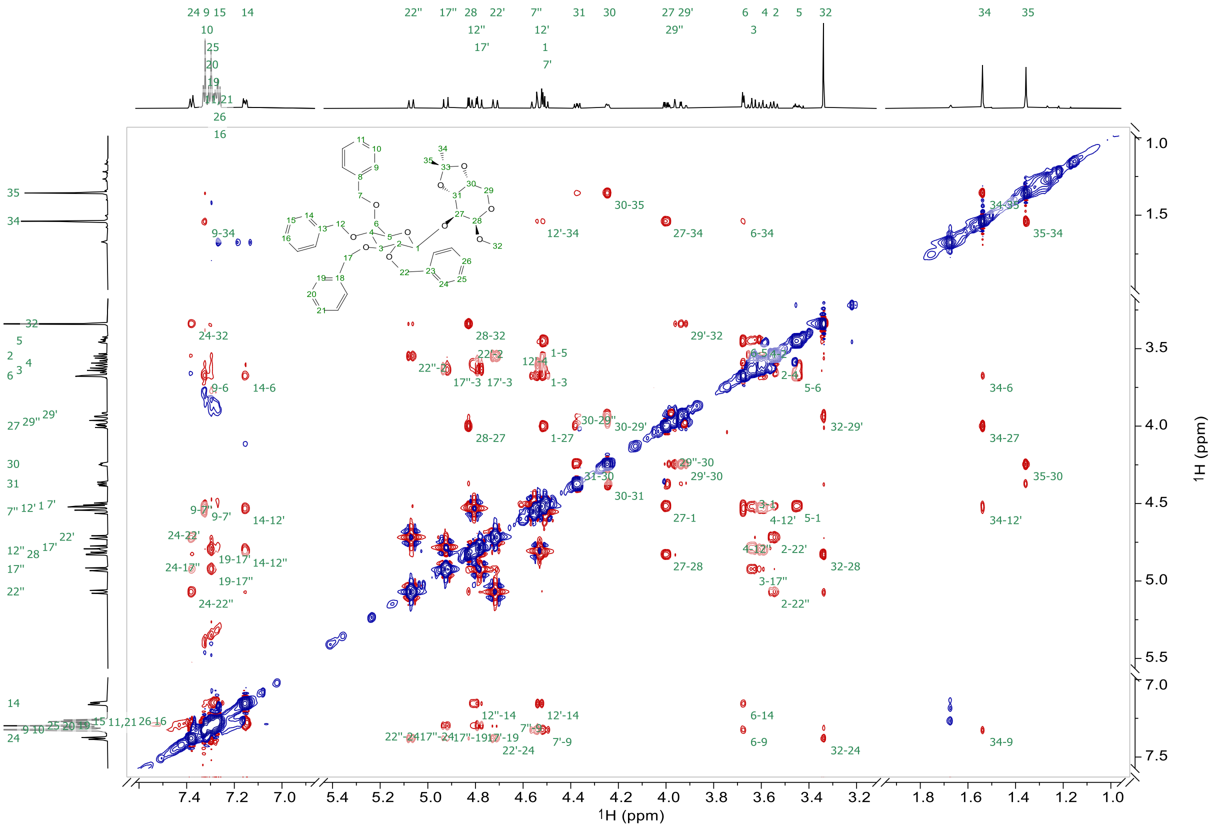

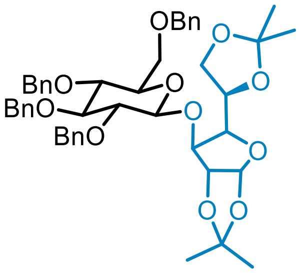

(3a*R*,5*R*,6*S*,6a*R*)-5-((*S*)-2,2-dimethyl-1,3-dioxolan-4-yl)-2,2-dimethyl-6-(((2*R*,3*S*,4*R*,6*S*)-3,4,5-tris(benzyloxy)-6-((benzyloxy)methyl)tetrahydro-2*H*-pyran-2-yl)oxy)tetrahydrofuro[2,3-d][1,3]dioxole (5d)

Following **General Procedure B**, glycosyl donor **1b** (34 mg, 0.050 mmol) and alcohol **2d** (20 mg, 0.075 mmol) were coupled using (*R*,*R*)-IDPi **3b** (8.2 mg,10 mol%). Reaction time: 72 h. β/α >95:5 was determined by crude ^{1}H NMR. Flash chromatography (hexane/EtOAc = 6/1 to 3/1) gave **5d** as white solid (36 mg, 92%). The NMR data of this compound are consistent with those reported in the literature.[9]

Following **General Procedure A** on 0.01 mmol scale, the glycosylation was carried out using (*S*,*S*)-IDPi **3b**. β/α > 95:5 was determined by crude ^{1}H NMR.

TLC: R_f = 0.32 (Hexane/EtOAc = 3:1)

^{1}H NMR (501 MHz, CDCl$_3$) δ 1.17 (s, 3H), 1.24 (s, 3H), 1.35 (s, 3H), 1.41 (s, 3H), 3.25 – 3.33 (m, 1H), 3.34 – 3.40 (m, 1H), 3.52 – 3.60 (m, 2H), 3.64 (d, *J* = 3.2 Hz, 2H), 4.00 (d, *J* = 6.4 Hz, 2H), 4.27 (d, *J* = 3.2 Hz, 1H), 4.31 (dd, *J* = 4.6, 3.1 Hz, 1H), 4.34 – 4.41 (m, 2H), 4.42 (d, *J* = 3.8 Hz, 1H), 4.46 – 4.57 (m, 3H), 4.66 (s, 2H), 4.75 (dd, *J* = 10.9, 6.2 Hz, 2H), 4.83 (d, *J* = 11.0 Hz, 1H), 5.70 (d, *J* = 3.8 Hz, 1H), 7.10 – 7.14 (m, 2H), 7.17 – 7.29 (m, 18H).

^{13}C NMR (126 MHz, CDCl$_3$) *δ* 25.50, 26.24, 26.70, 26.82, 66.00, 68.70, 73.64, 73.75, 75.13, 75.22, 75.51, 75.80, 77.81, 80.40, 80.42, 82.23, 82.75, 84.80, 101.47, 105.26, 108.64, 111.95, 127.77, 127.78, 127.79, 127.89, 127.97, 128.16, 128.52, 128.54, 128.57, 128.62, 138.13, 138.27, 138.35, 138.59.

$[\alpha]_D^{22}$ = -16.6 (CHCl$_3$, *c* = 1.0).

HRMS m/z (ESI): calcd. for C46 H54 Na1 O11 ([M+Na]$^+$): 805.35583; found: 805.35527.

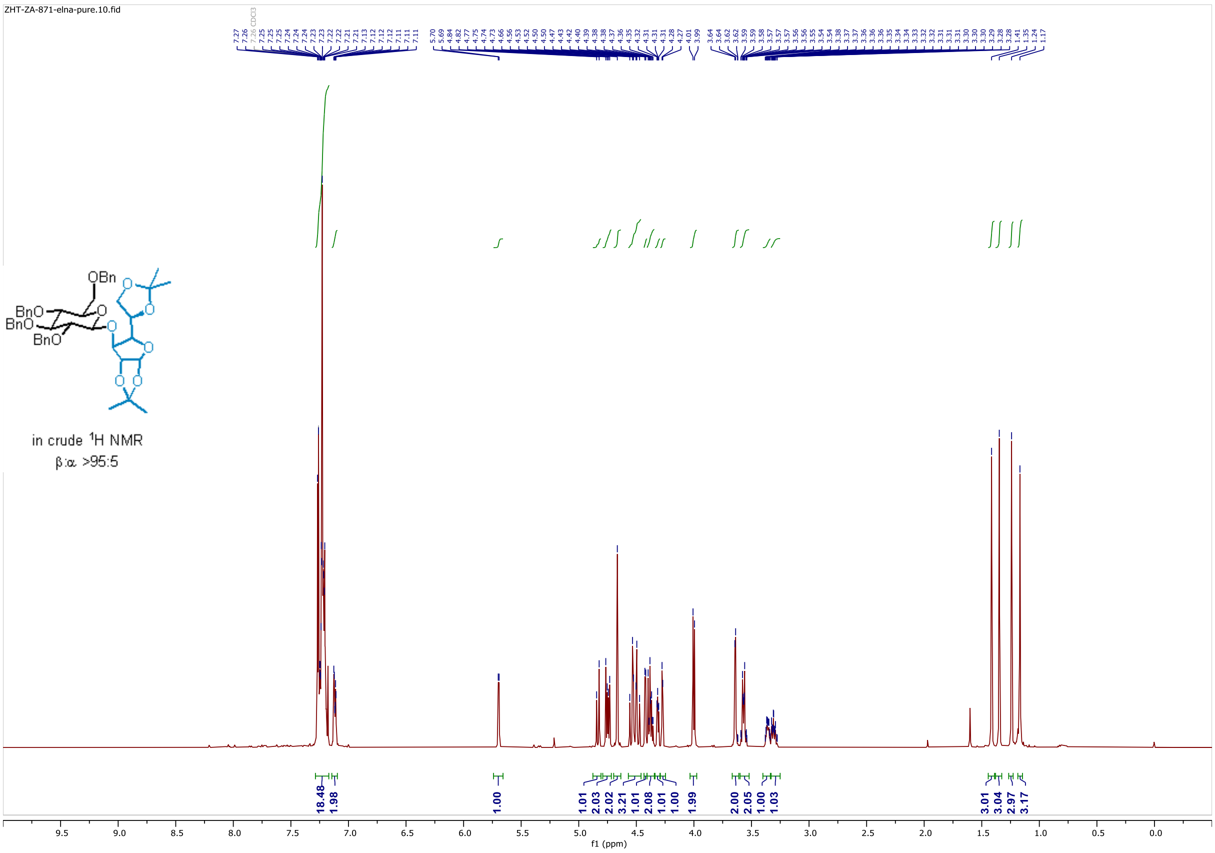
ZHT-ZA-871-elna-pure.10.fid
in crude ¹H NMR
β:α >95:5
f1 (ppm)

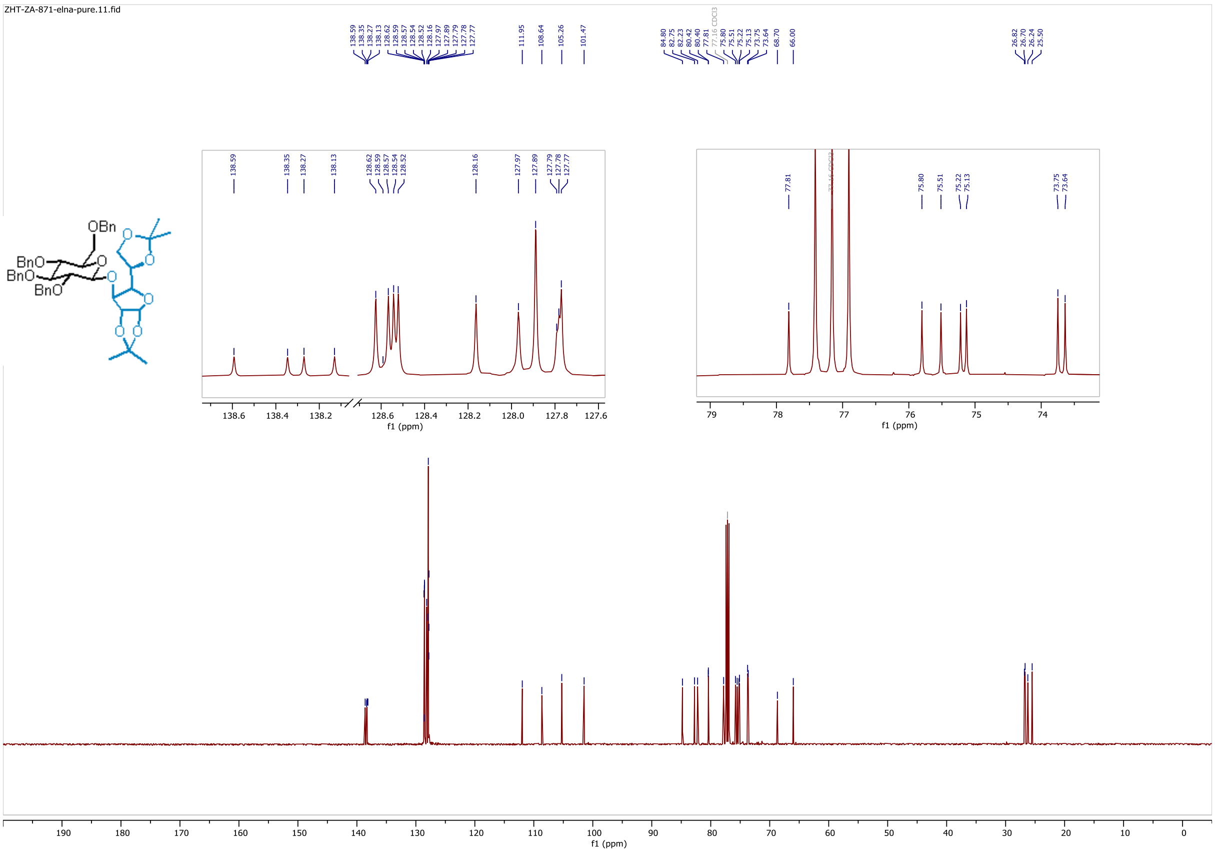
ZHT-ZA-871-elna-pure.11.fid
f1 (ppm)

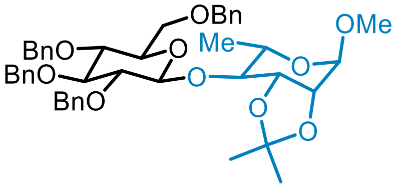

(3a*R*,4*R*,6*S*,7*S*,7a*R*)-4-methoxy-2,2,6-trimethyl-7-(((2*S*,3*R*,4*S*,5*R*,6*R*)-3,4,5-tris(benzyloxy)-6-((benzyloxy)methyl)tetrahydro-2*H*-pyran-2-yl)oxy)tetrahydro-4*H*-[1,3]dioxolo[4,5-c]pyran (5e)

Following **General Procedure B**, glycosyl donor **1b** (34 mg, 0.050 mmol) and alcohol **2e** (35 mg, 0.075 mmol) were coupled using (*R*,*R*)-IDPi **3b** (8.2 mg,10 mol%). Reaction time: 72 h. β/α >95:5 was determined by crude ^{1}H NMR. Flash chromatography (hexane/EtOAc = 8/1 to 4/1) gave **5e** as off-white foam (32 mg, 86%). The NMR data of this compound are consistent with those reported in the literature.[4]

Following **General Procedure A** on 0.01 mmol scale, the glycosylation was carried out using (*S*,*S*)-IDPi **3b**. β/α > 95:5 was determined by crude ^{1}H NMR.

TLC: R_f = 0.63 (Hexane/EtOAc = 3:1)

^{1}H NMR (501 MHz, $CDCl_3$) δ 1.26 (s, 3H), 1.28 (d, *J* = 6.0 Hz, 3H), 1.40 (s, 3H), 3.29 – 3.39 (m, 5H), 3.54 – 3.69 (m, 6H), 4.02 (d, *J* = 5.7 Hz, 1H), 4.15 (dd, *J* = 7.1, 5.6 Hz, 1H), 4.45 – 4.57 (m, 3H), 4.63 (d, *J* = 11.1 Hz, 1H), 4.71 (d, *J* = 11.0 Hz, 1H), 4.76 (d, *J* = 10.9 Hz, 1H), 4.80 (s, 1H), 4.83 – 4.89 (m, 3H), 7.09 – 7.16 (m, 2H), 7.16 – 7.24 (m, 12H), 7.26 (d, *J* = 4.5 Hz, 4H), 7.28 – 7.33 (m, 2H).

^{13}C NMR (126 MHz, $CDCl_3$) *δ* 17.93, 26.47, 27.98, 55.00, 64.40, 68.86, 73.60, 74.84, 75.02, 75.05, 75.73, 76.11, 78.00, 78.34, 78.37, 82.53, 84.97, 98.20, 101.77, 109.39, 127.63, 127.67, 127.68, 127.74, 127.80, 127.96, 128.04, 128.31, 128.41, 128.44, 128.49, 138.45, 138.81, 138.93.

$[\alpha]_D^{22}$ = -13.0 ($CHCl_3$, *c* = 1.0).

HRMS m/z (ESI): calcd. for C44 H52 O10 Na1 ($[M+Na]^+$): 763.345269; found: 763.345630.

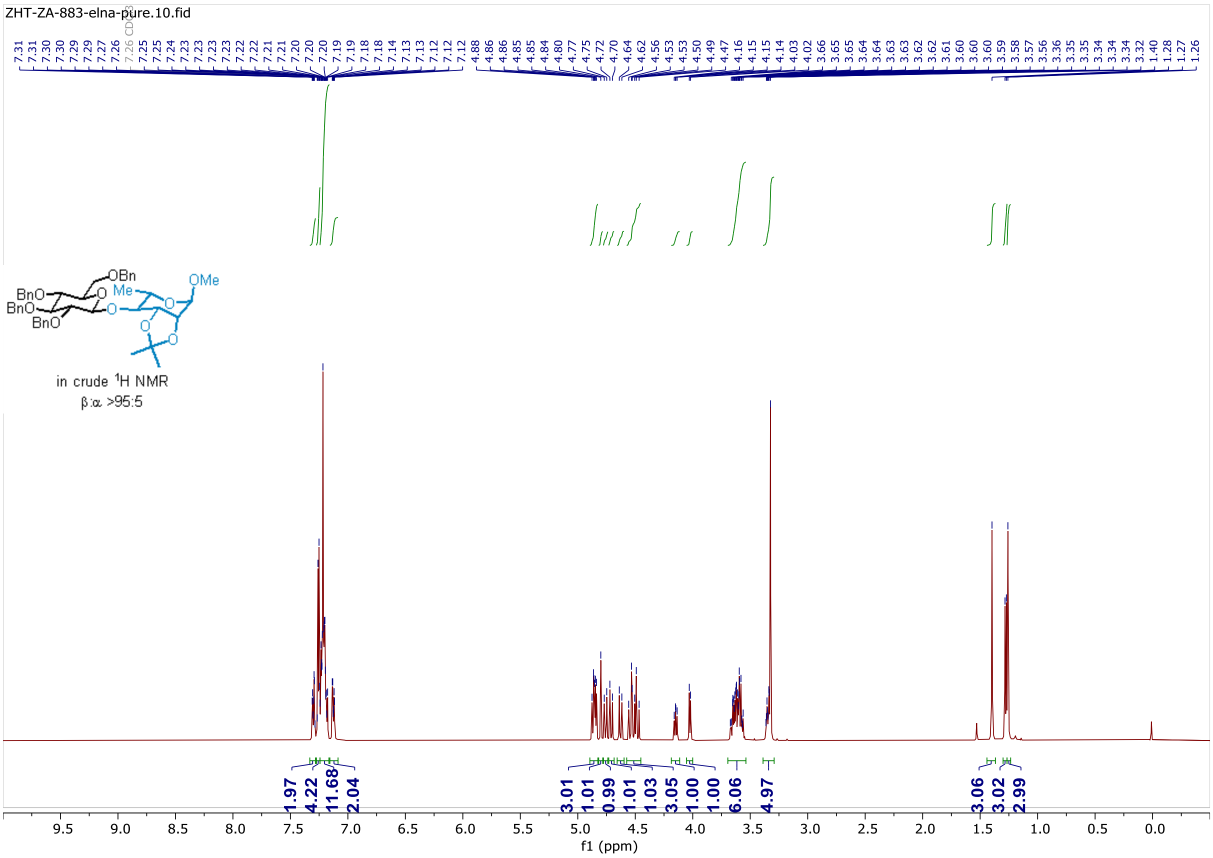
ZHT-ZA-883-elna-pure.10.fid
7.31 7.31 7.30 7.30 7.29 7.29 7.27 7.26 CDCl3 7.25 7.25 7.24 7.23 7.23 7.23 7.23 7.22 7.22 7.21 7.21 7.20 7.20 7.20 7.19 7.19 7.18 7.18 7.14 7.13 7.13 7.12 7.12 7.12 4.88 4.86 4.86 4.85 4.84 4.80 4.77 4.75 4.72 4.70 4.64 4.62 4.56 4.53 4.53 4.50 4.49 4.47 4.16 4.15 4.15 4.14 4.03 4.02 3.66 3.65 3.65 3.64 3.64 3.63 3.63 3.62 3.62 3.61 3.60 3.60 3.59 3.58 3.57 3.56 3.36 3.35 3.35 3.34 3.34 3.32 1.40 1.28 1.27 1.26
BnO
BnO
BnO
OBn
O
Me
O
OMe
O
O
O
in crude 1H NMR
β:α >95:5
1.97 4.22 11.68 2.04 3.01 1.01 0.99 1.01 1.03 3.05 1.00 1.00 6.06 4.97 3.06 3.02 2.99
9.5 9.0 8.5 8.0 7.5 7.0 6.5 6.0 5.5 5.0 4.5 4.0 3.5 3.0 2.5 2.0 1.5 1.0 0.5 0.0
f1 (ppm)

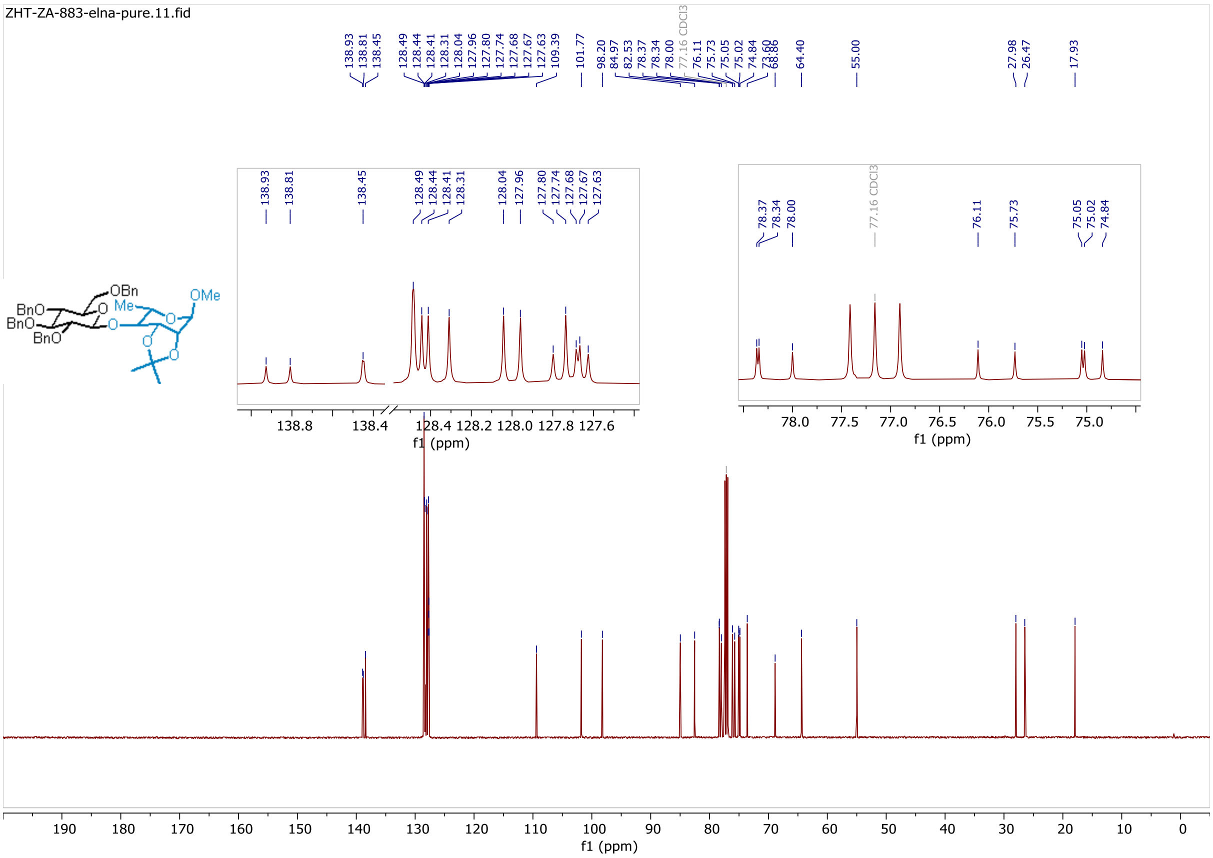
ZHT-ZA-883-elna-pure.11.fid
138.93 138.81 138.45 128.49 128.44 128.41 128.31 128.04 127.96 127.80 127.74 127.68 127.67 127.63 109.39 101.77 98.20 84.97 82.53 78.37 78.34 78.00 77.16 CDCl3 76.11 75.73 75.05 75.02 74.84 73.60 68.86 64.40 55.00 27.98 26.47 17.93
BnO
BnO
BnO
OBn
O
Me
O
OMe
O
O
O
138.8 138.4 128.4 128.2 128.0 127.8 127.6
f1 (ppm)
78.0 77.5 77.0 76.5 76.0 75.5 75.0
f1 (ppm)
190 180 170 160 150 140 130 120 110 100 90 80 70 60 50 40 30 20 10 0
f1 (ppm)

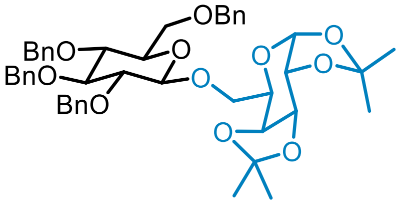

(3a*R*,5*R*,5a*S*,8a*S*,8b*R*)-2,2,7,7-tetramethyl-5-((((2*R*,3*R*,4*S*,5*R*,6*R*)-3,4,5-tris(benzyloxy)-6-((benzyloxy)methyl)tetrahydro-2*H*-pyran-2-yl)oxy)methyl)tetrahydro-5*H*-bis([1,3]dioxolo)[4,5-b:4',5'-d]pyran (5f)

Following **General Procedure B**, glycosyl donor **1b** (34 mg, 0.050 mmol) and alcohol **2f** (35 mg, 0.075 mmol) were coupled using (*R*,*R*)-IDPi **3b** (8.2 mg,10 mol%). Reaction time: 24 h. β/α = 97:3 was determined by crude ^{1}H NMR. Flash chromatography (hexane/EtOAc = 6/1 to 3/1) gave **5f** as off-white foam (38 mg, 97%). The NMR data of this compound are consistent with those reported in the literature.[8]

Following **General Procedure A** on 0.01 mmol scale, the glycosylation was carried out using (*S*,*S*)-IDPi **3b**. β/α > 95:5 was determined by crude ^{1}H NMR.

TLC: R_f = 0.31 (Hexane/EtOAc = 3:1)

^{1}H NMR (501 MHz, CDCl$_3$) δ 1.27 (d, *J* = 2.5 Hz, 6H), 1.41 (s, 3H), 1.46 (s, 3H), 3.35 – 3.47 (m, 2H), 3.53 – 3.62 (m, 2H), 3.62 – 3.74 (m, 3H), 4.02 – 4.09 (m, 1H), 4.12 (dd, *J* = 10.7, 3.7 Hz, 1H), 4.21 (dd, *J* = 7.9, 1.9 Hz, 1H), 4.28 (dd, *J* = 5.0, 2.4 Hz, 1H), 4.42 (d, *J* = 7.8 Hz, 1H), 4.48 (dd, *J* = 12.7, 11.5 Hz, 2H), 4.52 – 4.61 (m, 2H), 4.68 (d, *J* = 11.2 Hz, 1H), 4.75 (dd, *J* = 17.5, 10.9 Hz, 2H), 4.92 (d, *J* = 11.0 Hz, 1H), 5.01 (d, *J* = 11.1 Hz, 1H), 5.53 (d, *J* = 5.0 Hz, 1H), 7.10 (dd, *J* = 7.3, 2.2 Hz, 2H), 7.19 – 7.33 (m, 17H), 7.36 – 7.40 (m, 2H).

^{13}C NMR (126 MHz, CDCl$_3$) *δ* 24.59, 25.17, 26.14, 26.18, 67.49, 68.91, 69.85, 70.62, 70.93, 71.59, 73.64, 74.48, 74.90, 75.13, 75.80, 77.87, 81.77, 84.70, 96.53, 104.54, 108.71, 109.51, 127.61, 127.67, 127.74, 127.84, 128.00, 128.02, 128.09, 128.34, 128.47, 128.49, 128.50, 128.78, 138.30, 138.31, 138.85.

$[\alpha]_D^{22}$ = -27.8 (CHCl$_3$, *c* = 1.0).

HRMS m/z (ESI): calcd. for C46 H54 O11 Na1 ([M+Na]$^+$): 805.355834; found: 805.355920.

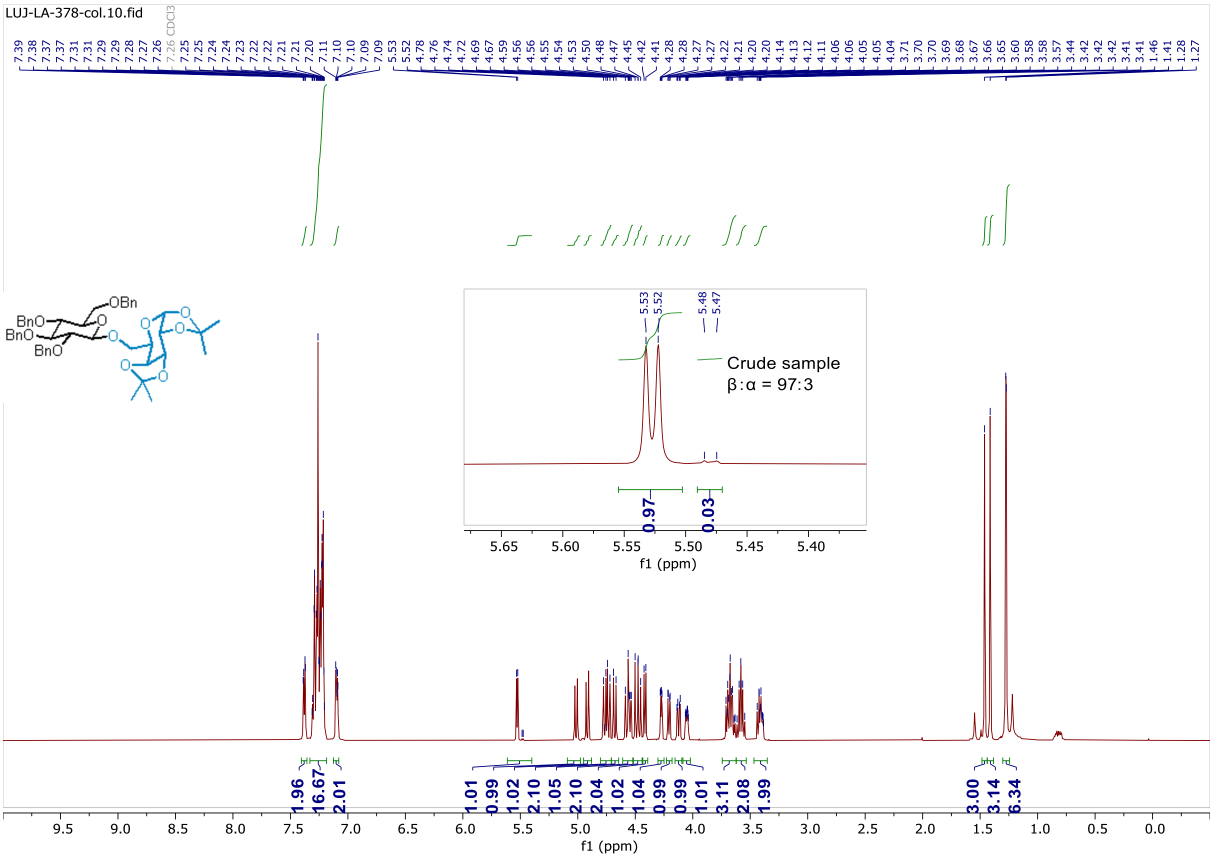
LUJ-LA-378-col.10.fid
7.39 7.38 7.37 7.37 7.31 7.31 7.29 7.29 7.28 7.27 7.26 CDCl3 7.25 7.25 7.24 7.24 7.23 7.22 7.22 7.21 7.21 7.20 7.11 7.10 7.10 7.09 7.09 5.53 5.52 4.78 4.76 4.74 4.72 4.69 4.67 4.59 4.56 4.56 4.55 4.54 4.53 4.50 4.48 4.47 4.45 4.42 4.41 4.28 4.28 4.27 4.22 4.21 4.20 4.20 4.14 4.13 4.12 4.11 4.06 4.06 4.05 4.05 4.04 3.71 3.70 3.69 3.68 3.67 3.66 3.65 3.60 3.58 3.58 3.57 3.44 3.42 3.42 3.42 3.41 1.46 1.41 1.28 1.27
OBn
BnO
BnO
BnO
O
Crude sample
β:α = 97:3
5.53
5.52
5.48
5.47
0.97
0.03
5.65 5.60 5.55 5.50 5.45 5.40
f1 (ppm)
1.96 16.67 2.01 1.01 0.99 1.02 2.10 1.05 2.10 2.04 1.02 1.04 0.99 0.99 1.01 3.11 2.08 1.99 3.00 3.14 6.34
9.5 9.0 8.5 8.0 7.5 7.0 6.5 6.0 5.5 5.0 4.5 4.0 3.5 3.0 2.5 2.0 1.5 1.0 0.5 0.0
f1 (ppm)

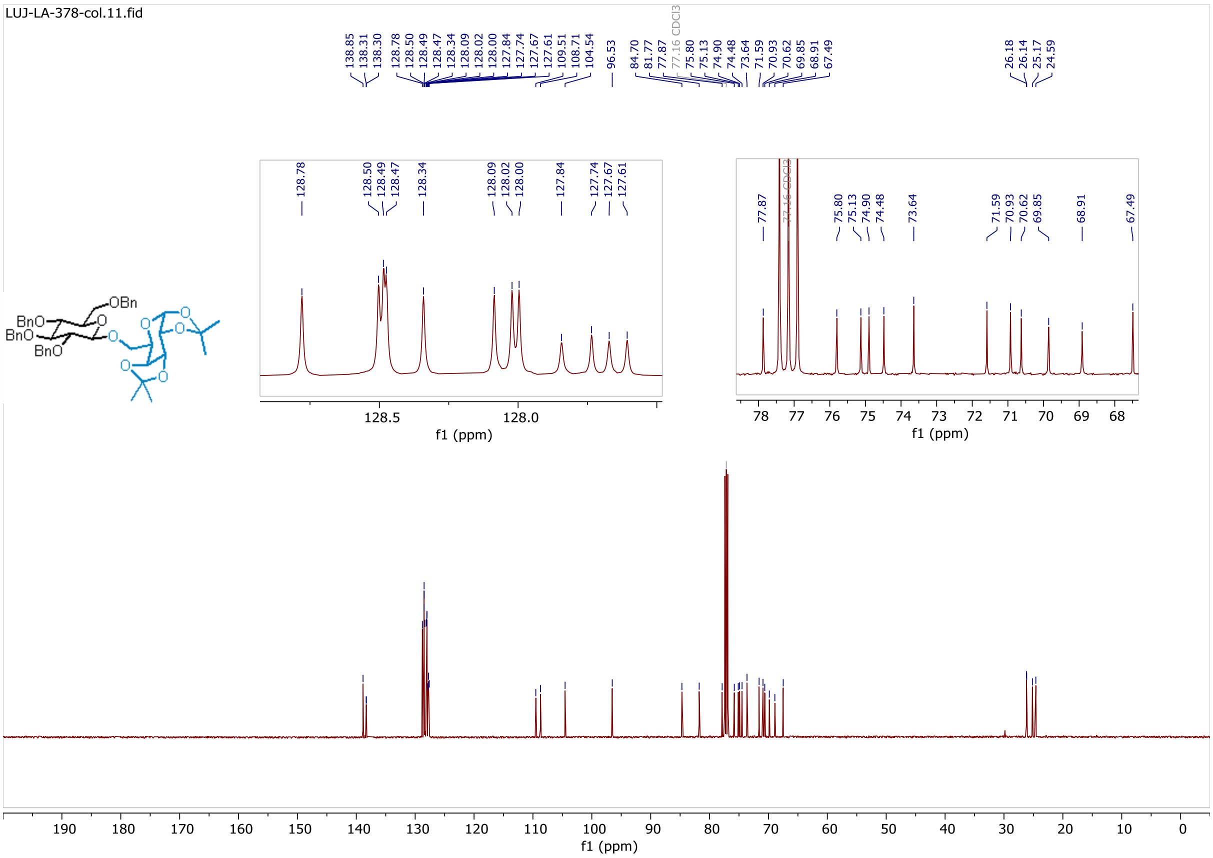
LUJ-LA-378-col.11.fid
138.85 138.31 138.30 128.78 128.50 128.49 128.47 128.34 128.09 128.02 128.00 127.84 127.74 127.67 127.61 109.51 108.71 104.54 96.53 84.70 81.77 77.87 77.16 CDCl3 75.80 75.13 74.90 74.48 73.64 71.59 70.93 70.62 69.85 68.91 67.49 26.18 26.14 25.17 24.59
OBn
BnO
BnO
BnO
O
128.78 128.50 128.49 128.47 128.34 128.09 128.02 128.00 127.84 127.74 127.67 127.61
128.5 128.0
f1 (ppm)
77.87 77.16 CDCl3 75.80 75.13 74.90 74.48 73.64 71.59 70.93 70.62 69.85 68.91 67.49
78 77 76 75 74 73 72 71 70 69 68
f1 (ppm)
190 180 170 160 150 140 130 120 110 100 90 80 70 60 50 40 30 20 10 0
f1 (ppm)

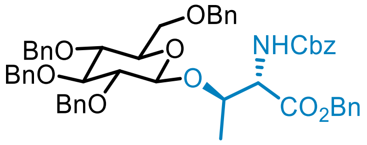

benzyl ***N*****-((*****benzyloxy*****)carbonyl)-*****O*****-((2*****R*****,3*****R*****,4*****S*****,5*****R*****,6*****R*****)-3,4,5-tris(*****benzyloxy*****)-6-((*****benzyloxy*****)methyl)tetrahydro-2*****H*****-pyran-2-yl)-L-threoninate (5g)**

Following **General Procedure B**, glycosyl donor **1b** (34 mg, 0.050 mmol) and alcohol **2f** (35 mg, 0.075 mmol) were coupled using (*R*,*R*)-IDPi **3b** (8.2 mg,10 mol%). Reaction time: 24 h. β/α = 97:3 was determined by crude ^{1}H NMR. Flash chromatography (hexane/EtOAc = 6/1 to 4/1) gave **5f** as colorless oil (25 mg, 59%).

Following **General Procedure A** on 0.01 mmol scale, the glycosylation was carried out using (*S*,*S*)-IDPi **3b**. β/α = 82:18 was determined by crude ^{1}H NMR.

TLC: R_f = 0.36 (Hexane/EtOAc = 3:1)

^{1}H NMR (501 MHz, $CDCl_3$) δ 1.30 (d, *J* = 6.4 Hz, 3H), 3.27 (dt, *J* = 9.7, 2.9 Hz, 1H), 3.34 (dd, *J* = 9.0, 7.8 Hz, 1H), 3.57 (t, *J* = 9.0 Hz, 1H), 3.59 – 3.67 (m, 3H), 4.33 – 4.39 (m, 2H), 4.41 (dd, *J* = 8.5, 3.2 Hz, 1H), 4.43 – 4.52 (m, 2H), 4.53 (d, *J* = 10.8 Hz, 1H), 4.67 (d, *J* = 11.0 Hz, 1H), 4.75 – 4.86 (m, 3H), 4.90 (d, *J* = 11.0 Hz, 1H), 5.10 – 5.23 (m, 4H), 5.81 (d, *J* = 8.5 Hz, 1H), 7.12 – 7.17 (m, 2H), 7.22 – 7.37 (m, 30H).

^{13}C NMR (126 MHz, $CDCl_3$) δ 170.37, 156.92, 138.72, 138.33, 138.25, 136.47, 135.60, 128.69, 128.63, 128.55, 128.52, 128.51, 128.50, 128.46, 128.45, 128.33, 128.25, 128.24, 128.17, 128.06, 127.90, 127.89, 127.87, 127.78, 127.73, 127.67, 101.57, 84.66, 82.01, 77.49, 75.73, 75.15, 74.87, 73.57, 68.73, 67.29, 67.22, 59.07, 17.84.

$[\alpha]_D^{22}$ = +1.0 ($CHCl_3$, *c* = 1.0).

HRMS m/z (ESI): calcd. for C53 H55 N1 O10 Na1 ($[M+Na]^+$): 888.371817; found: 888.371220.

User Report
LUJ-LA-377-01

The following compound was assigned in the sample

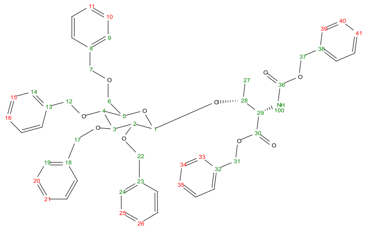

Remarks:
The relative stereochemistry at the anomeric center is supported by NOE correlations from H1 to the axial positioned protons H3 and H5. The J coupling of H1 to H2 is also large (7.8 Hz), which supports a *trans* configuration at positions 1 & 2.

1H{},NOESY, 500.81 MHz,CDCl3,298.0K, pulse sequence: noesygpphpp

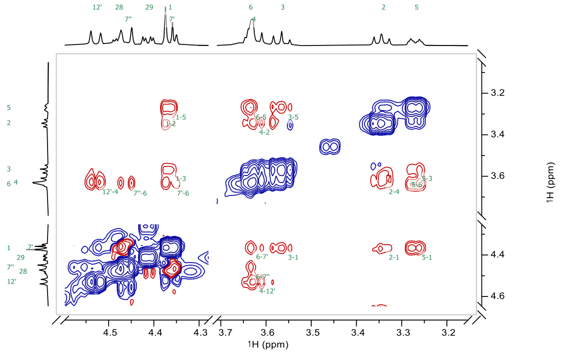

P-ID: ML000000
Measured on: 09/01/2026
CHIFFRE: LUJ-LA-377-01
ELNA#: 16038
Client: Jiaxiang Lu
Group: List
Spectroscopist: Leutzsch
Analysed on: 20/02/2026
Analysed by: Leutzsch
Amount: 20.0 mg
Solvent: CDCl3
Reference: 1H+13C on solvent,other nuclei w/ xiref
Temperature: 298 K
Spectrometer: AV500lis
Probe: prodigy-BBO
Experiments: 1H-1H-noesygpphpp, 1H-zg30, 13C-zgpg30, 1H-1H-cosygpppqf, 1H-13C-hsqcedetgpsisp2
1H-13C-hmbcetgpl3nd, 1H-1H-noesygpphpp

Atom	δ (ppm)	J	COSY	HSQC	HMBC	NOESY
1 C	101.568			1', 1''	2, 5	
H	4.366	7.80(2)	2	1	3, 5	2, 3, 5, 27
2 C	82.007			2	3, 22', 22''	
H	3.343	9.00(3), 7.80(1)	1, 3	2	1, 3, 22	1, 4, 22', 22''
3 C	84.660			3	1, 2, 4, 5, 17', 17''	
H	3.566	8.80(4), 9.00(2)	2, 4	3	2, 4, 17	1, 5, 17', 17''
4 C	77.493			4	3, 5, 6, 12', 12''	
H	3.629	8.80(3), 9.70(5)	3, 5	4	3, 5, 6	2, 12', 12''
5 C	74.867			5	1, 4, 6	
H	3.270	9.70(4), 2.90(6)	4, 6	5	1, 3, 4	1, 3, 6
6 C	68.729			6	4, 7', 7''	
H2	3.636	2.90(5)	5	6	4, 5, 7	5, 7', 7'', 9, 14
7 C	73.571			7', 7''	6, 9	
H'	4.363	12.20(7'')	7''	7	6, 8	6, 9
H''	4.460	12.20(7')	7'	7	6, 8	6, 9
8 C	138.246				7', 7''	
9 C						
H	7.239				7	6, 7', 7''
10 C						
H						
11 C						
H						
12 C	75.149			12', 12''	14	
H'	4.528	10.80(12'')	12''	12	4, 13	4, 14
H''	4.795	10.80(12')	12'	12	4, 13	4, 14
13 C	138.246				12', 12''	
14 C						
H	7.149				12	6, 12', 12''

Atom	δ (ppm)	J	COSY	HSQC	HMBC	NOESY
15 C						
H						
16 C						
H						
17 C	75.733			17', 17''	3, 19	
H'	4.792	10.90(17'')	17''	17	3, 18	3, 19
H''	4.899	10.90(17')	17'	17	3, 18	3, 19, 24
18 C	138.717				17', 17''	
19 C						
H	7.297				17	17', 17''
20 C						
H						
21 C						
H						
22 C	75.085			22', 22''	2, 24	
H'	4.674	11.00(22'')	22''	22	2, 23	2, 24
H''	4.819	11.00(22')	22'	22	2, 23	2, 24
23 C	138.329				22', 22''	
24 C						
H	7.311				22	17'', 22', 22''
25 C						
H						
26 C						
H						
27 C	17.837			27		
H3	1.296	6.40(28)	28	27	28, 29	1, 28, 29
28 C	74.843			28	27, 29	
H	4.479	3.20(29), 6.40(27)	27, 29	28		27, 100

Atom	δ (ppm)	J	COSY	HSQC	HMBC	NOESY
29 C	59.071			29	27	
H	4.413	8.50(100), 3.20(28)	28, 100	29	28, 30, 36	27, 100
30 C	170.366				29, 31', 31''	
31 C	67.292			31', 31''		
H'	5.165		31''	31	30, 32	
H''	5.165		31'	31	30, 32	
32 C	135.603				31', 31''	
33 C						
H						
34 C						
H						
35 C						
H						
36 C	156.924				29, 37	
37 C	67.217			37		
H2	5.132			37	36, 38	
38 C	136.466				37	
39 C						
H						
40 C						
H						
41 C						
H						
100 N						
H	5.808	8.50(29)	29			28, 29

1H{},1D, 500.81 MHz,CDCl3,298.0K, pulse sequence: zg30

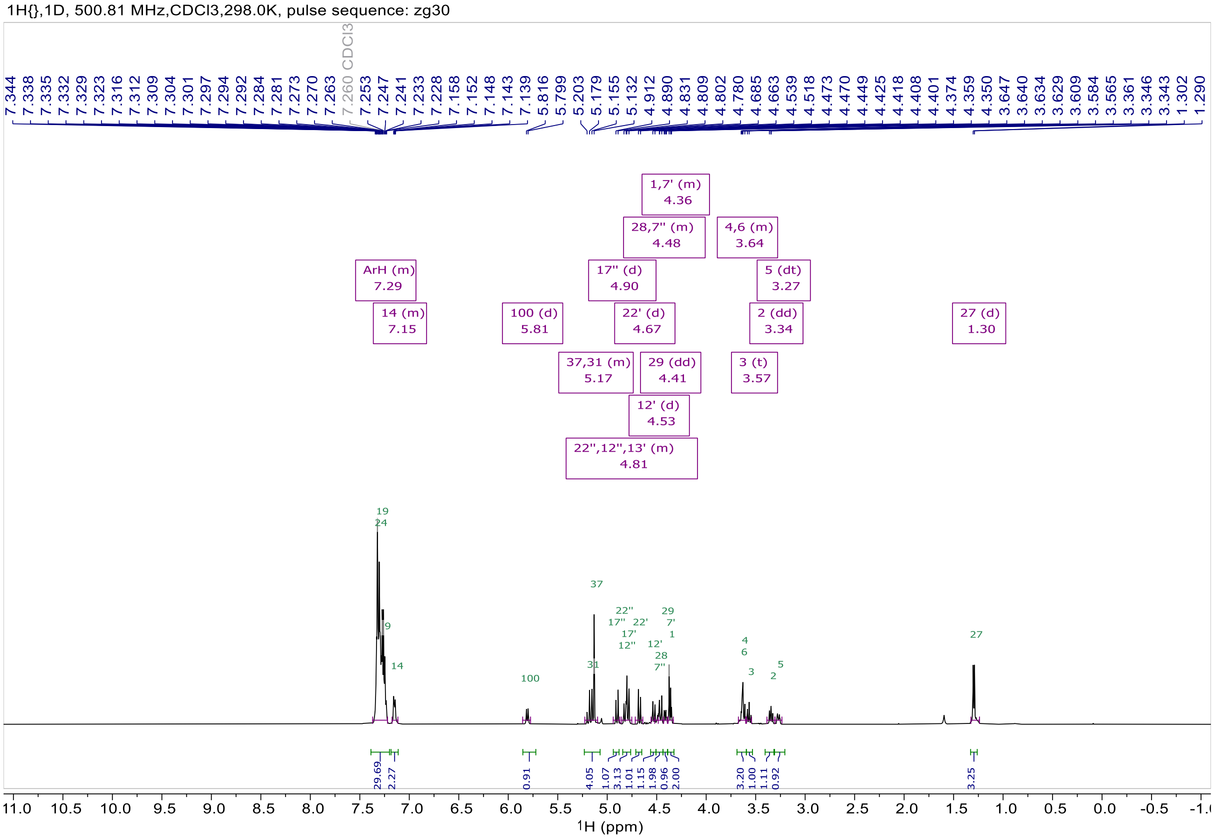

13C{},1D, 125.94 MHz,CDCl3,298.0K, pulse sequence: zgpg30

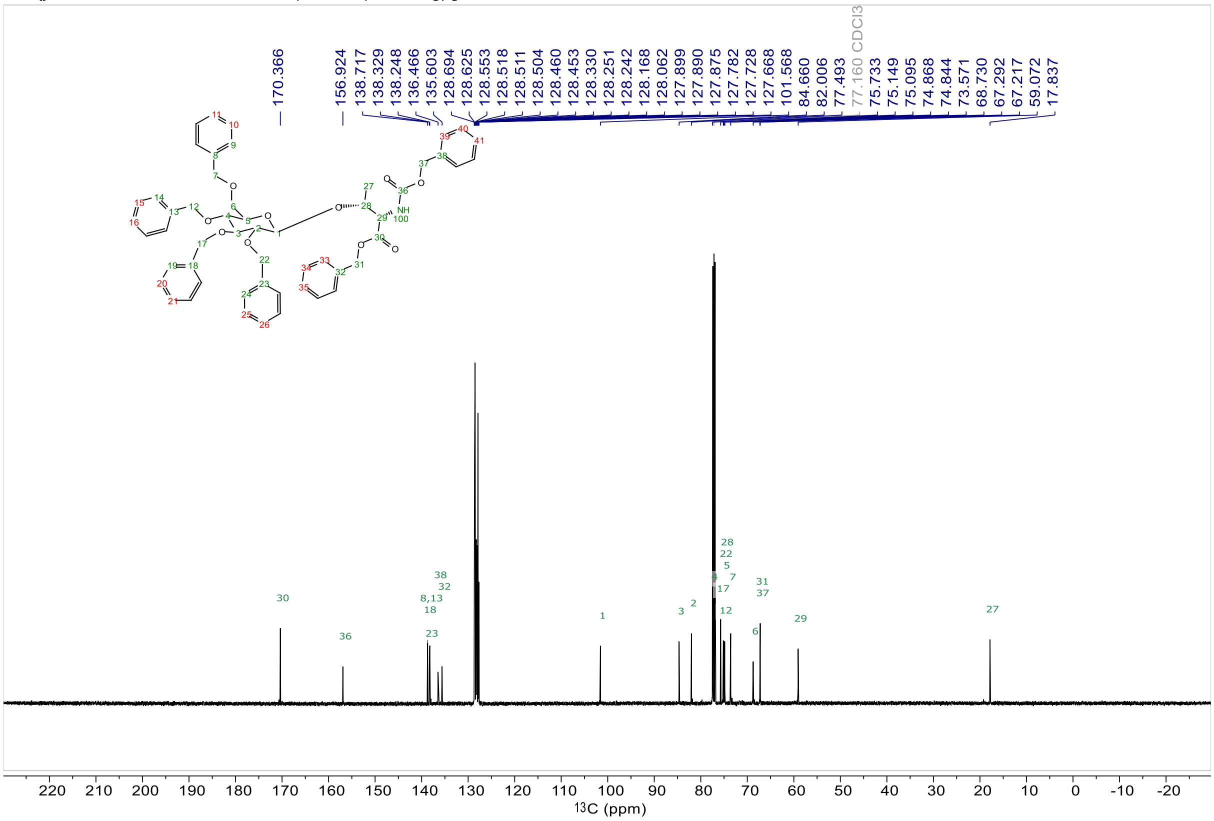

1H{},COSY, 500.81 MHz,CDCl3,298.0K, pulse sequence: cosygpppqf

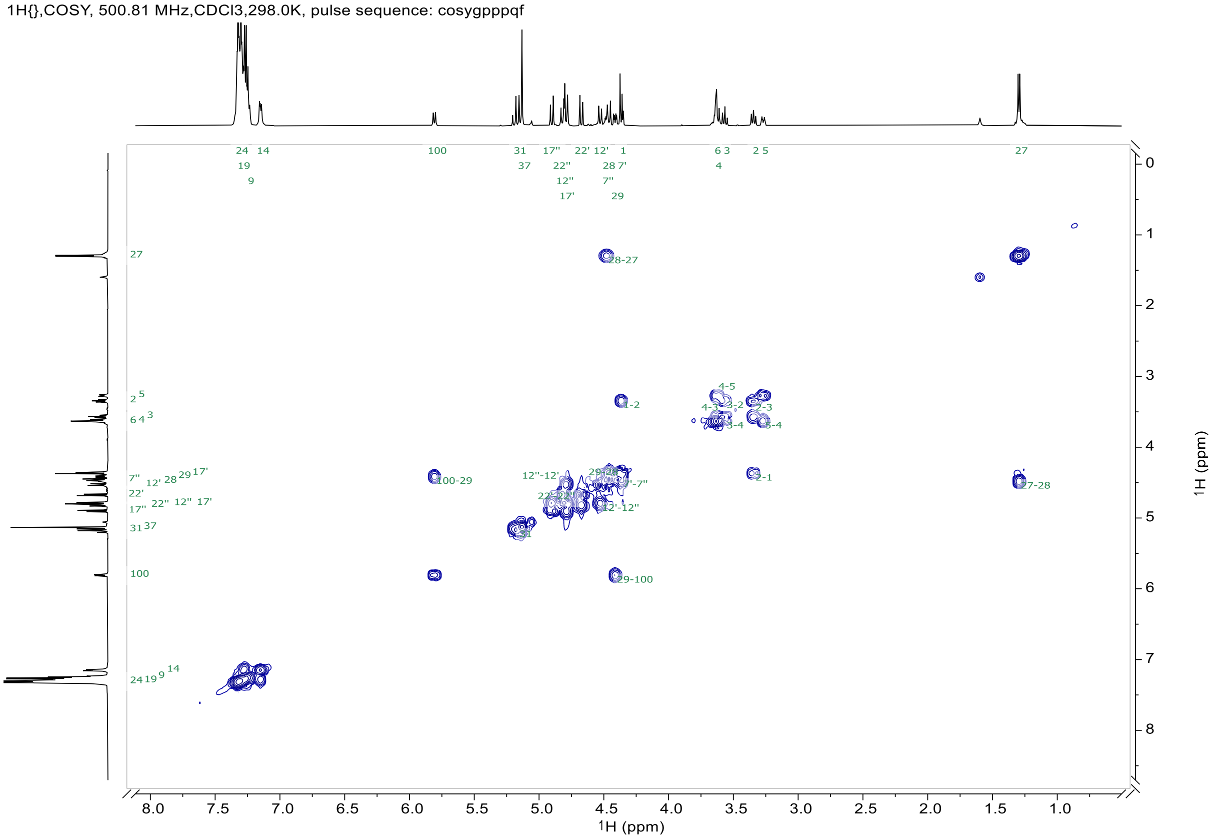

1H{},HSQC-EDITED, 500.81 MHz,CDCl3,298.0K, pulse sequence: hsqcedetgpsisp2.3

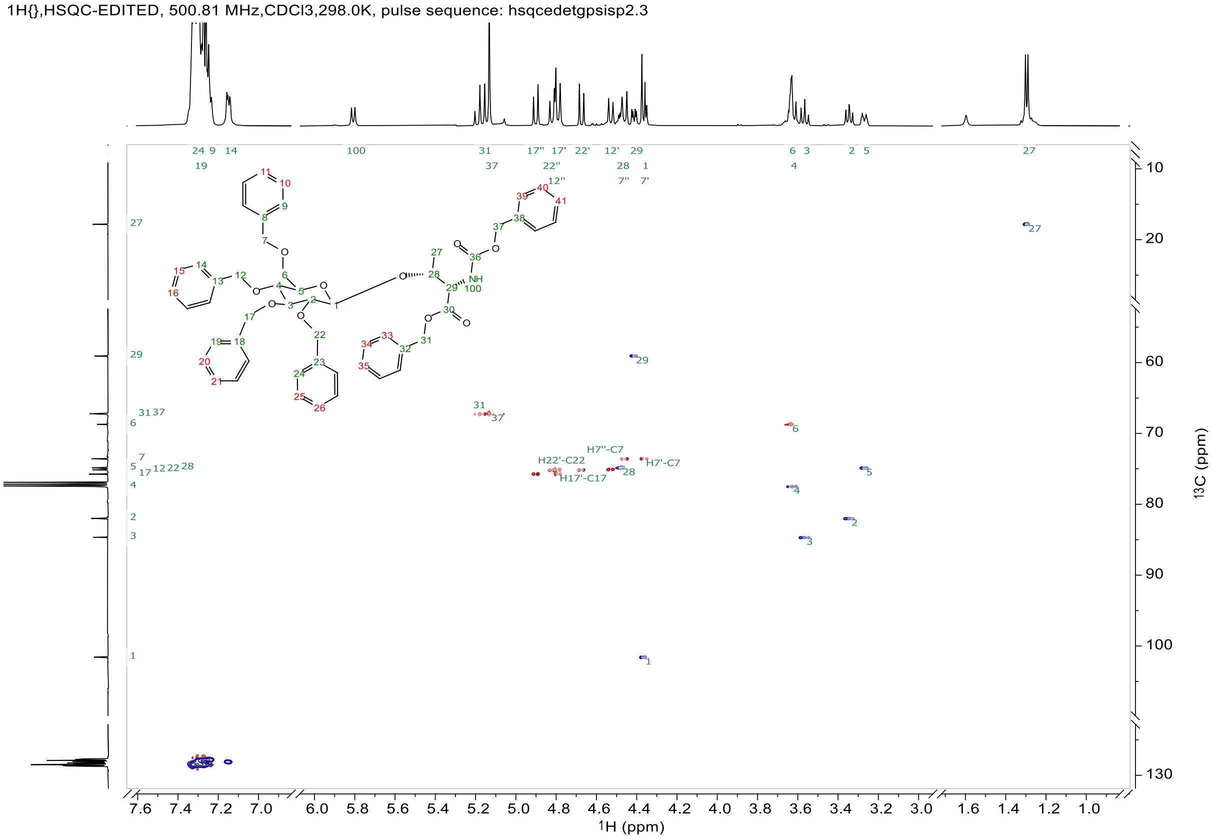

1H{},HMBC, 500.81 MHz,CDCl3,298.0K, pulse sequence: hmbcetgpl3nd

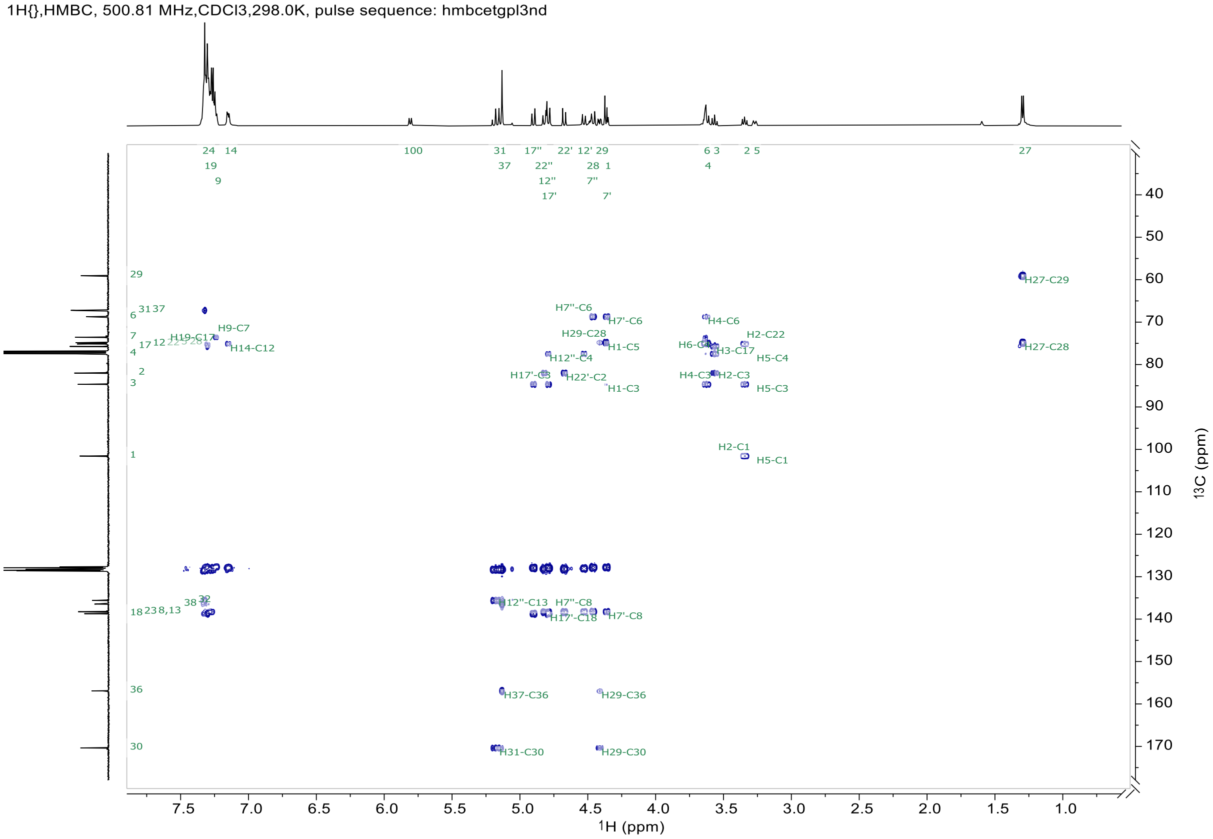

1H{},NOESY, 500.81 MHz,CDCl3,298.0K, pulse sequence: noesygpphpp

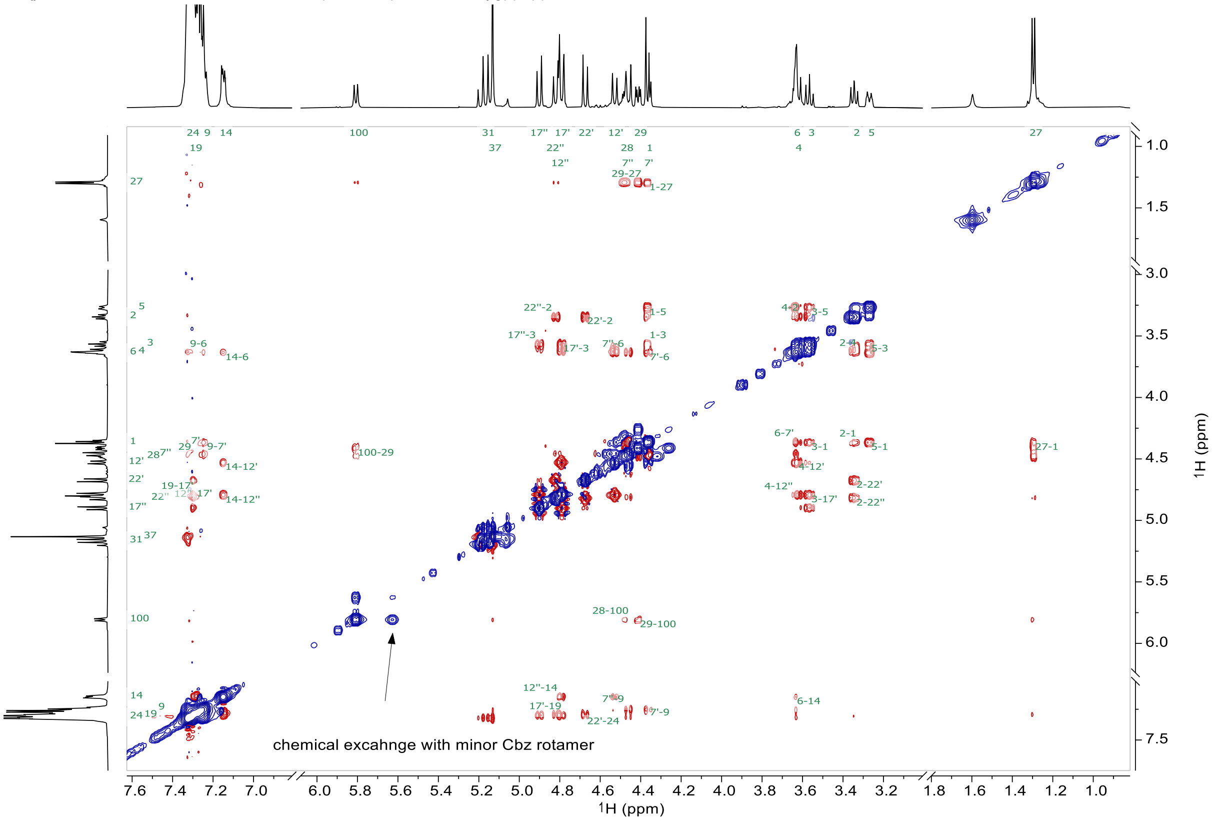

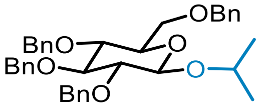

(2*R*,3*R*,4*S*,5*R*,6*R*)-3,4,5-tris(benzyloxy)-2-((benzyloxy)methyl)-6-isopropoxytetrahydro-2*H*-pyran (5h)

Following **General Procedure B**, glycosyl donor **1b** (34 mg, 0.050 mmol) and alcohol **2g** (4.5 mg, 0.075 mmol) were coupled using (*R*,*R*)-IDPi **3b** (8.2 mg,10 mol%). Reaction time: 72 h. β/α = 99:1 was determined by crude ^{1}H NMR. Flash chromatography (hexane/EtOAc = 10/1 to 6/1) gave **5g** as white solid (28 mg, 96%). The NMR data of this compound are consistent with those reported in the literature.[4]

Following **General Procedure A** on 0.01 mmol scale, the glycosylation was carried out using (*S*,*S*)-IDPi **3b**. β/α > 95:5 was determined by crude ^{1}H NMR.

TLC: R_f = 0.82 (Hexane/EtOAc = 3:1)

^{1}H NMR (501 MHz, CDCl$_3$) δ 1.17 (d, *J* = 6.1 Hz, 3H), 1.24 (d, *J* = 6.2 Hz, 3H), 3.33 – 3.41 (m, 2H), 3.47 (t, *J* = 9.3 Hz, 1H), 3.54 – 3.62 (m, 2H), 3.66 (dd, *J* = 10.7, 2.0 Hz, 1H), 3.95 (p, *J* = 6.2 Hz, 1H), 4.39 (d, *J* = 7.8 Hz, 1H), 4.44 – 4.51 (m, 2H), 4.54 (d, *J* = 12.2 Hz, 1H), 4.63 (d, *J* = 10.8 Hz, 1H), 4.73 (dd, *J* = 19.2, 10.8 Hz, 2H), 4.85 (d, *J* = 10.9 Hz, 1H), 4.90 (d, *J* = 10.8 Hz, 1H), 7.10 (dd, *J* = 7.5, 2.0 Hz, 2H), 7.16 – 7.24 (m, 12H), 7.24 – 7.31 (m, 6H).

^{13}C NMR (126 MHz, CDCl$_3$) *δ* 22.39, 23.88, 69.34, 72.51, 73.57, 74.98, 75.13, 75.83, 78.15, 82.47, 85.00, 102.34, 127.68, 127.71, 127.79, 127.84, 127.88, 128.03, 128.14, 128.36, 128.47, 128.50, 128.52, 138.29, 138.46, 138.69, 138.83.

$[\alpha]_D^{22}$ = +9.4 (CHCl$_3$, *c* = 1.0).

HRMS m/z (ESI): calcd. for C37 H42 Na1 O6 ([M+Na]$^+$): 605.28736; found: 605.28740.

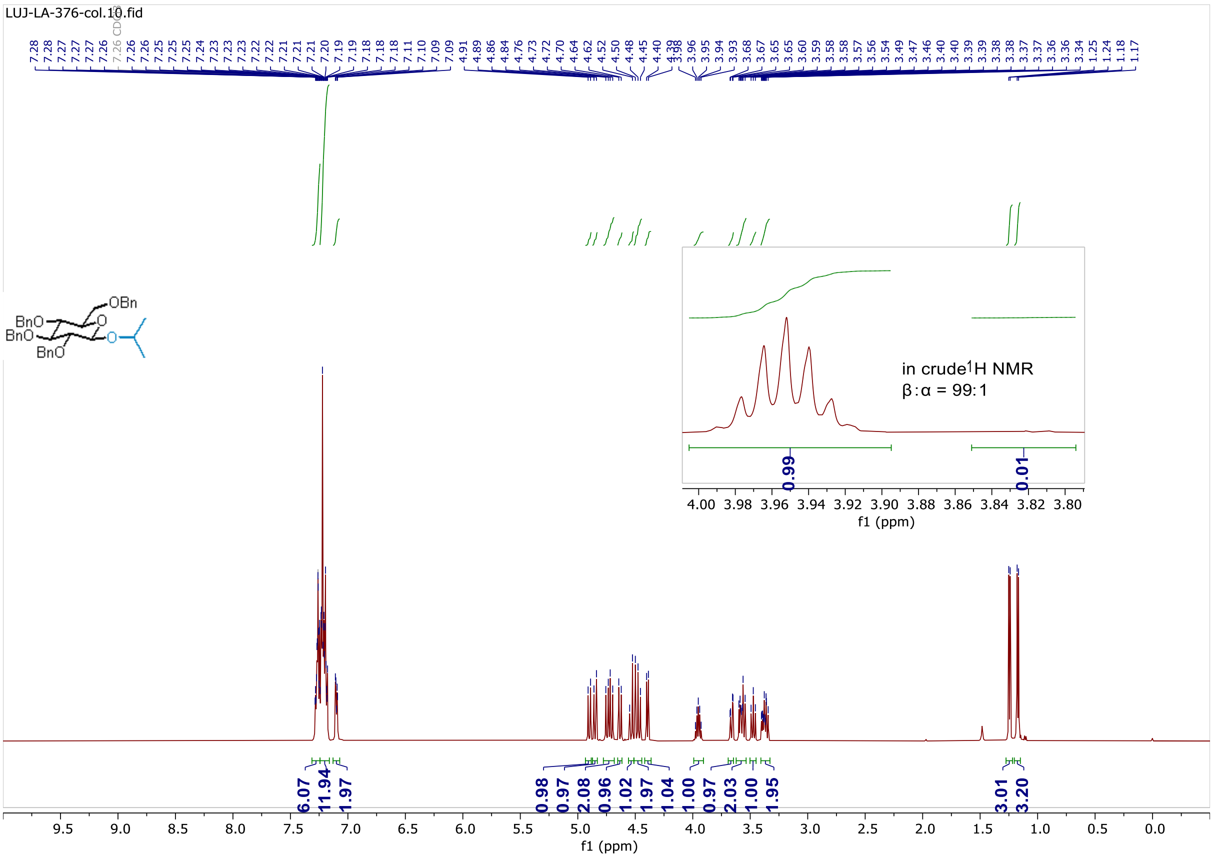
LUJ-LA-376-col.10.fid
in crude 1H NMR
β:α = 99:1
f1 (ppm)

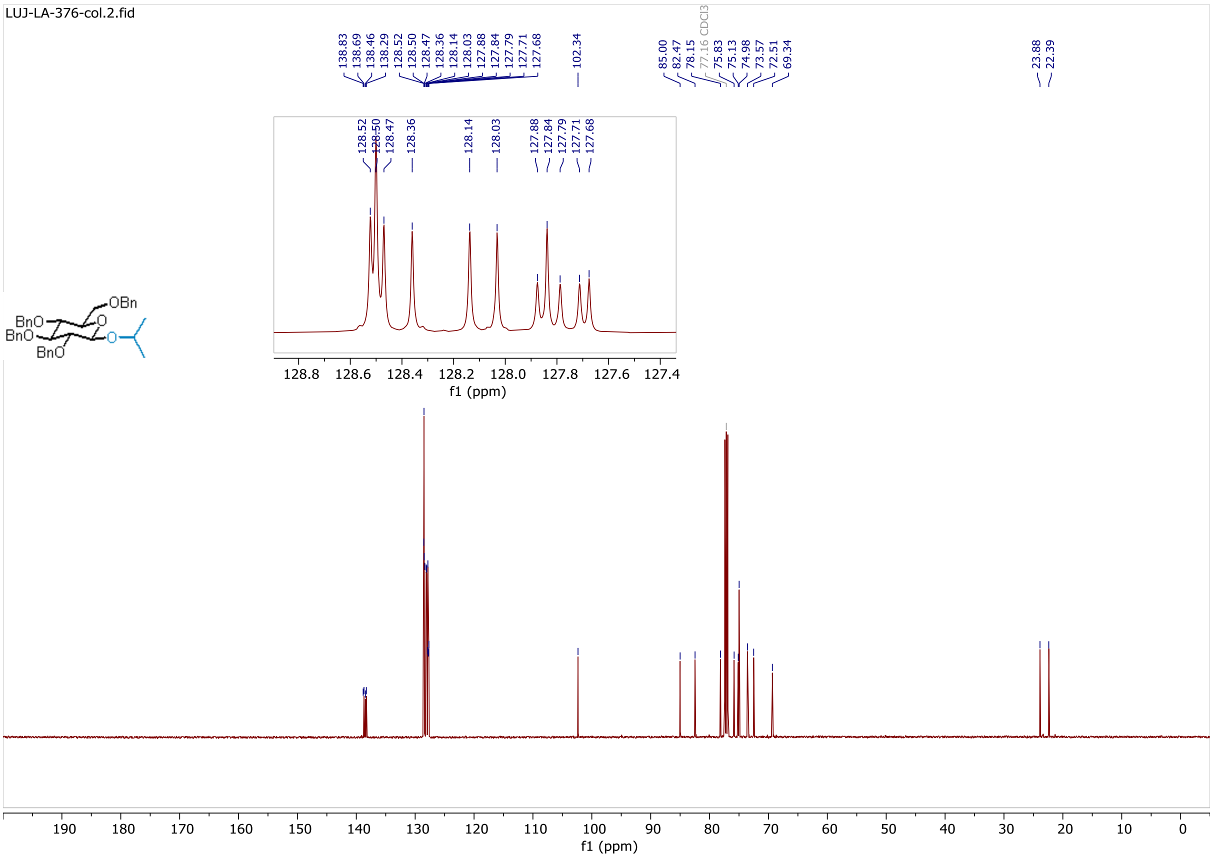
LUJ-LA-376-col.2.fid
f1 (ppm)

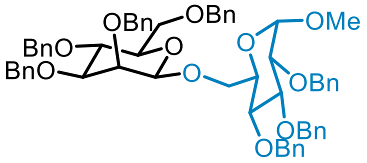

(2*R*,3*R*,4*S*,5*S*,6*R*)-3,4,5-tris(benzyloxy)-2-((benzyloxy)methyl)-6-(((2*R*,3*R*,4*S*,5*R*,6*S*)-3,4,5-tris(benzyloxy)-6-methoxytetrahydro-2*H*-pyran-2-yl)methoxy)tetrahydro-2*H*-pyran (6a)

Following **General Procedure B**, glycosyl donor **1c** (34 mg, 0.050 mmol) and alcohol **2a** (35 mg, 0.075 mmol) were coupled using (*S*,*S*)-IDPi **3c** (9.4 mg,10 mol%). Reaction time: 24 h. β/α = 95:5 was determined by crude ^{1}H NMR. Flash chromatography (hexane/EtOAc = 6/1 to 3/1) gave **6a** as light-yellow foam (48 mg, 97%). The NMR data of this compound are consistent with those reported in the literature.[9]

Following **General Procedure A** on 0.01 mmol scale, the glycosylation was carried out using (*R*,*R*)-IDPi **3c**. β/α = 83:17 was determined by crude ^{1}H NMR.

TLC: R_f = 0.28 (Hexane/EtOAc = 3:1)

^{1}H NMR (501 MHz, $CDCl_3$) δ 3.30 (s, 3H), 3.33 – 3.46 (m, 4H), 3.48 (dd, *J* = 9.7, 3.5 Hz, 1H), 3.66 – 3.72 (m, 2H), 3.72 – 3.83 (m, 3H), 3.99 (t, *J* = 9.2 Hz, 1H), 4.09 (s, 1H), 4.13 (dd, *J* = 10.5, 2.0 Hz, 1H), 4.42 – 4.58 (m, 7H), 4.63 (d, *J* = 12.1 Hz, 1H), 4.74 (d, *J* = 2.3 Hz, 1H), 4.75 – 4.82 (m, 3H), 4.85 (d, *J* = 10.8 Hz, 1H), 4.91 (d, *J* = 12.5 Hz, 1H), 4.99 (d, *J* = 10.9 Hz, 1H), 7.14 – 7.20 (m, 7H), 7.21 – 7.40 (m, 28H).

^{13}C NMR (126 MHz, $CDCl_3$) *δ* 55.17, 68.41, 69.86, 69.91, 71.68, 73.46, 73.61, 73.69, 73.79, 74.86, 75.09, 75.29, 75.84, 76.12, 77.77, 79.99, 82.28, 82.40, 97.91, 101.61, 127.53, 127.58, 127.68, 127.73, 127.75, 127.79, 127.80, 127.94, 128.07, 128.19, 128.22, 128.29, 128.31, 128.40, 128.41, 128.46, 128.48, 128.50, 128.53, 128.60, 138.19, 138.29, 138.41, 138.42, 138.58, 138.85, 138.96.

$[\alpha]_D^{22}$ = +19.4 ($CHCl_3$, *c* = 1.0).

HRMS m/z (ESI): calcd. for C62 H66 O11 Na1 ($[M+Na]^+$): 1009.449734; found: 1009.448710.

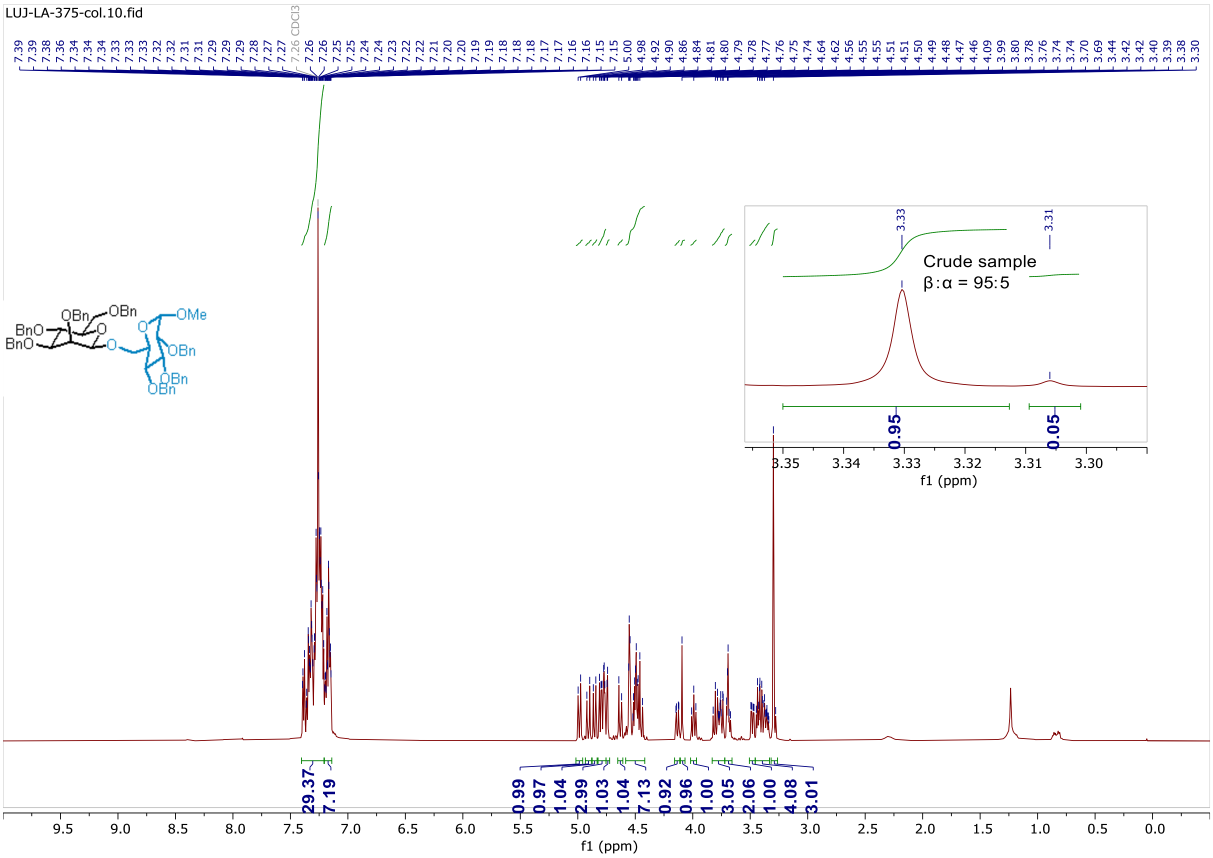
LUJ-LA-375-col.10.fid
Crude sample
β:α = 95:5
f1 (ppm)

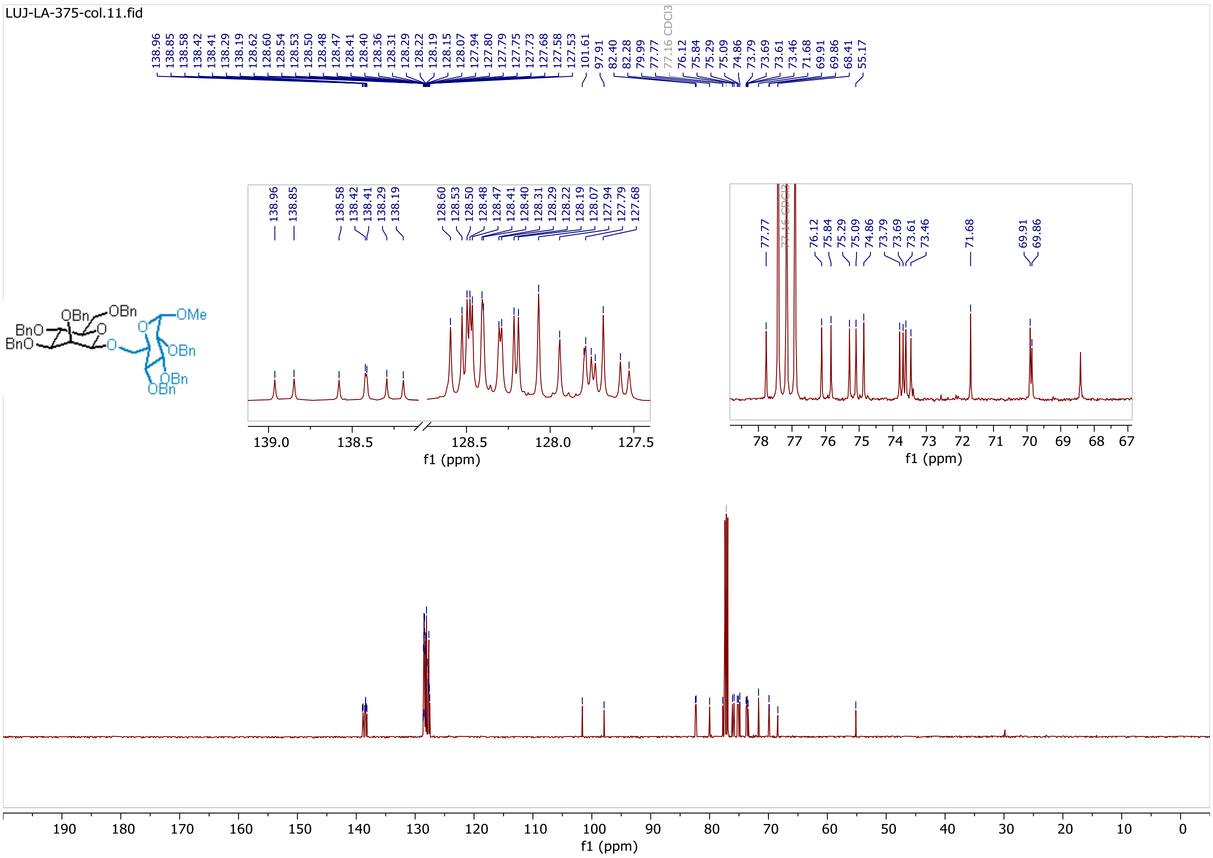
LUJ-LA-375-col.11.fid
f1 (ppm)

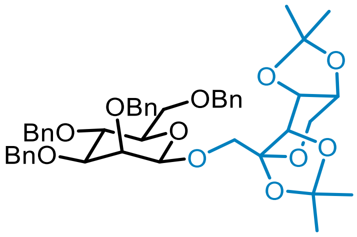

(3a*R*,5a*S*,8a*S*,8b*R*)-2,2,7,7-tetramethyl-3a-((((2*R*,3*S*,4*S*,5*R*,6*R*)-3,4,5-tris(benzyloxy)-6-((benzyloxy)methyl)tetrahydro-2*H*-pyran-2-yl)oxy)methyl)tetrahydro-5*H*-bis([1,3]dioxolo)[4,5-b:4',5'-d]pyran (6b)

Following **General Procedure B**, glycosyl donor **1c** (34 mg, 0.050 mmol) and alcohol **2b** (20 mg, 0.075 mmol) were coupled using (*S*,*S*)-IDPi **3c** (9.4 mg,10 mol%). Reaction time: 24 h. β/α = 95:5 was determined by crude ^{1}H NMR. Flash chromatography (hexane/EtOAc = 7/1 to 3/1) gave **6b** as light-yellow oil (33 mg, 86%).

Following **General Procedure A** on 0.01 mmol scale, the glycosylation was carried out using (*R*,*R*)-IDPi **3c**. β/α = 76:24 was determined by crude ^{1}H NMR.

TLC: R_f = 0.30 (Hexane/EtOAc = 3:1)

^{1}H NMR (501 MHz, CDCl$_3$) δ 7.46 – 7.41 (m, 2H), 7.35 – 7.22 (m, 16H), 7.21 – 7.16 (m, 2H), 4.94 (d, *J* = 12.3 Hz, 1H), 4.88 (d, *J* = 10.8 Hz, 1H), 4.84 (d, *J* = 12.3 Hz, 1H), 4.67 (d, *J* = 12.1 Hz, 1H), 4.59 (dd, *J* = 7.9, 2.7 Hz, 1H), 4.57 – 4.51 (m, 5H), 4.38 (d, *J* = 2.7 Hz, 1H), 4.22 (dd, *J* = 8.0, 1.6 Hz, 1H), 4.00 – 3.93 (m, 2H), 3.94 – 3.87 (m, 2H), 3.80 – 3.75 (m, 3H), 3.73 (d, *J* = 12.9 Hz, 1H), 3.50 (dd, *J* = 9.3, 2.9 Hz, 1H), 3.42 (dt, *J* = 9.7, 3.5 Hz, 1H), 1.51 (s, 3H), 1.45 (s, 3H), 1.34 (s, 3H), 1.21 (s, 3H).

^{13}C NMR (126 MHz, CDCl$_3$) δ 138.92, 138.60, 138.53, 138.31, 128.50, 128.44, 128.38, 128.22, 128.21, 128.14, 127.96, 127.80, 127.78, 127.74, 127.53, 127.47, 109.18, 108.54, 102.64, 101.92, 82.61, 76.13, 75.22, 74.98, 74.40, 74.04, 73.62, 72.07, 71.06, 70.20, 70.09, 61.32, 26.74, 26.02, 25.75, 24.16.

$[\alpha]_D^{22}$ = -28.2 (CHCl$_3$, *c* = 1.0).

HRMS m/z (ESI): calcd. for C46 H54 O11 Na1 ([M+Na]$^+$): 805.355834; found: 805.355650.

User Report
LUJ-LA-379-01

The following compound was assigned in the sample

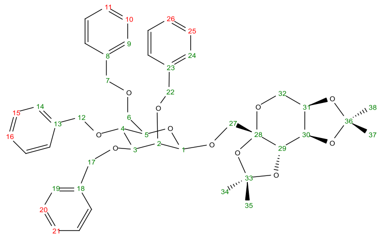

Remarks:
The relative stereochemistry at the anomeric center is supported by NOE correlations from H1 to the axial positioned protons H3 and H5. The J coupling of H1 to H2 could not be resolved and is therefore likely <1Hz.

1H{},NOESY, 500.81 MHz,CDCl3,298.0K, pulse sequence: noesygpphpp

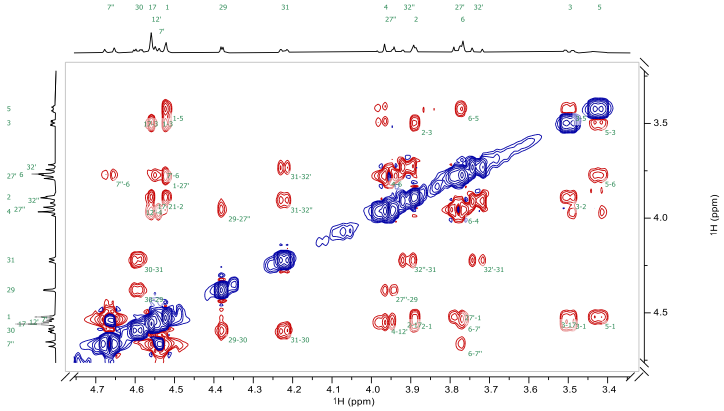

P-ID: ML00001
Measured on: 07/01/2026
CHIFFRE:LUJ-LA-379-01
ELNA#:16037
Client: Jiaxiang Lu
Group: List
Spectroscopist: Leutzsch
Analysed on: 21/02/2026
Analysed by: Leutzsch
Amount: 20.0 mg
Solvent: CDCl3
Reference: 1H+13C on solvent,other nuclei w/ xiref
Temperature: 298 K
Spectrometer: AV500lis
Probe: prodigy-BBO
Experiments: 1H-zg30, 13C-zgpg30, 1H-1H-cosygpppqf, 1H-13C-hsqcedetgpsisp2.3, 1H-13C-hmbcetgpl3nd, 1H-1H-noesygpphpp

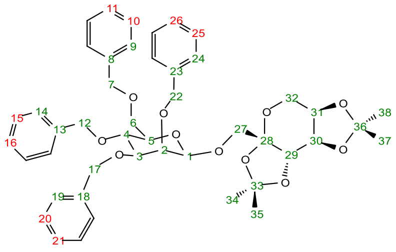

Atom	δ (ppm)	J	COSY	HSQC	HMBC	NOESY
1 C	101.920			1	2, 5, 27', 27''	
H	4.521			1	2, 3, 5	2, 3, 5, 27'
2 C	74.398			2	1, 3, 22', 22''	
H	3.889	2.90(3)	3	2	1, 3, 4, 22	1, 3, 17', 17'', 22', 22'', 35
3 C	82.605			3	1, 2, 4, 5, 17', 17''	
H	3.498	9.30(4), 2.90(2)	2, 4	3	2, 4, 17	1, 2, 5, 17', 17''
4 C	74.980			4	2, 3, 5, 6', 6'', 12', 12''	
H	3.966	9.30(3), 9.70(5)	3, 5	4	3, 5, 6, 12	6', 6'', 12', 12''
5 C	76.132			5	1, 4, 6', 6''	
H	3.423	9.70(4), 3.50(6'), 3.50(6'')	4, 6', 6''	5	1, 3, 4	1, 3, 6', 6''
6 C	69.353			6', 6''	4, 7', 7''	
H'	3.771	3.50(5)	5, 6''	6	4, 5, 7	4, 5, 7', 7''
H''	3.771	3.50(5)	5, 6'	6	4, 5, 7	4, 5, 7', 7''
7 C	73.618			7', 7''	6', 6'', 9	
H'	4.536	12.10(7'')	7''	7	6, 8	6', 6'', 9
H''	4.665	12.10(7')	7'	7	6, 8	6', 6'', 9
8 C	138.595				7', 7''	
9 C						
H	7.324				7	7', 7''
10 C						
H						
11 C						
H						
12 C	75.216			12', 12''	4, 14	
H'	4.549	10.80(7'')	12''	12	4, 13	4, 14
H''	4.885		12'	12	4, 13	4, 14

Atom	δ (ppm)	J	COSY	HSQC	HMBC	NOESY
13 C	138.525				12', 12''	
14 C						
H	7.192				12	12', 12''
15 C						
H						
16 C						
H						
17 C	72.074			17', 17''	3, 19	
H'	4.559		17''	17	3, 18	2, 3, 19
H''	4.559		17'	17	3, 18	2, 3, 19
18 C	138.305				17', 17''	
19 C						
H	7.288				17	17', 17''
20 C						
H						
21 C						
H						
22 C	74.036			22', 22''	2, 24	
H'	4.835	12.30(22'')	22''	22	2, 23	2, 24
H''	4.936	12.30(22')	22'	22	2, 23	2, 24
23 C	138.918				22', 22''	
24 C						
H	7.439				22	22', 22''
25 C						
H						
26 C						
H						

Atom	δ (ppm)	J	COSY	HSQC	HMBC	NOESY
27 C	69.500			27', 27''		
H'	3.779	12.20(27'')	27''	27	1, 28, 29	1
H''	3.954	12.20(27')	27'	27	1	29
28 C	102.643				27', 29, 30, 32', 32''	
29 C	70.094			29	27', 30	
H	4.380	2.70(30)		29	28, 30, 31	27'', 30, 35
30 C	70.201			30	29, 32'	
H	4.593	2.70(29), 7.90(31)	31	30	28, 29, 32	29, 31, 38
31 C	71.062			31	29, 32', 32''	
H	4.222	7.90(30), 1.90(32'')	30	31	32, 36	30, 32', 32'', 38
32 C	61.317			32', 32''	30, 31	
H'	3.731	12.90(32'')	32''	32	28, 30, 31	31
H''	3.908	1.90(31), 12.90(32')	32'	32	28, 31	31, 34
33 C	108.540				34, 35	
34 C	26.738			34	35	
H3	1.506			34	33, 35	32'', 35
35 C	25.752			35	34	
H3	1.209			35	33, 34	2, 29, 34
36 C	109.176				31, 37, 38	
37 C	26.021			37	38	
H3	1.450			37	36, 38	38
38 C	24.162			38	37	
H3	1.337			38	36, 37	30, 31, 37

1H{},1D, 500.81 MHz,CDCl3,298.0K, pulse sequence: zg30

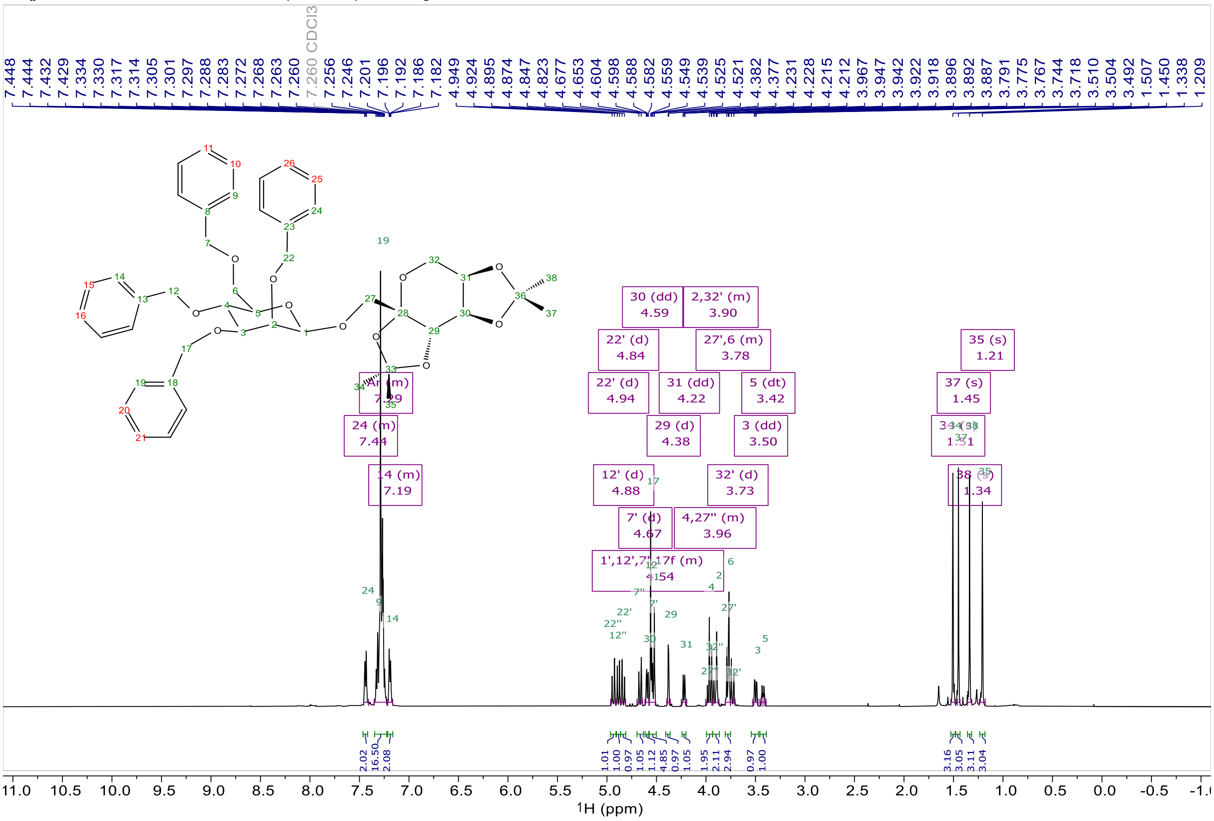

13C{},1D, 125.94 MHz,CDCl3,298.0K, pulse sequence: zgpg30

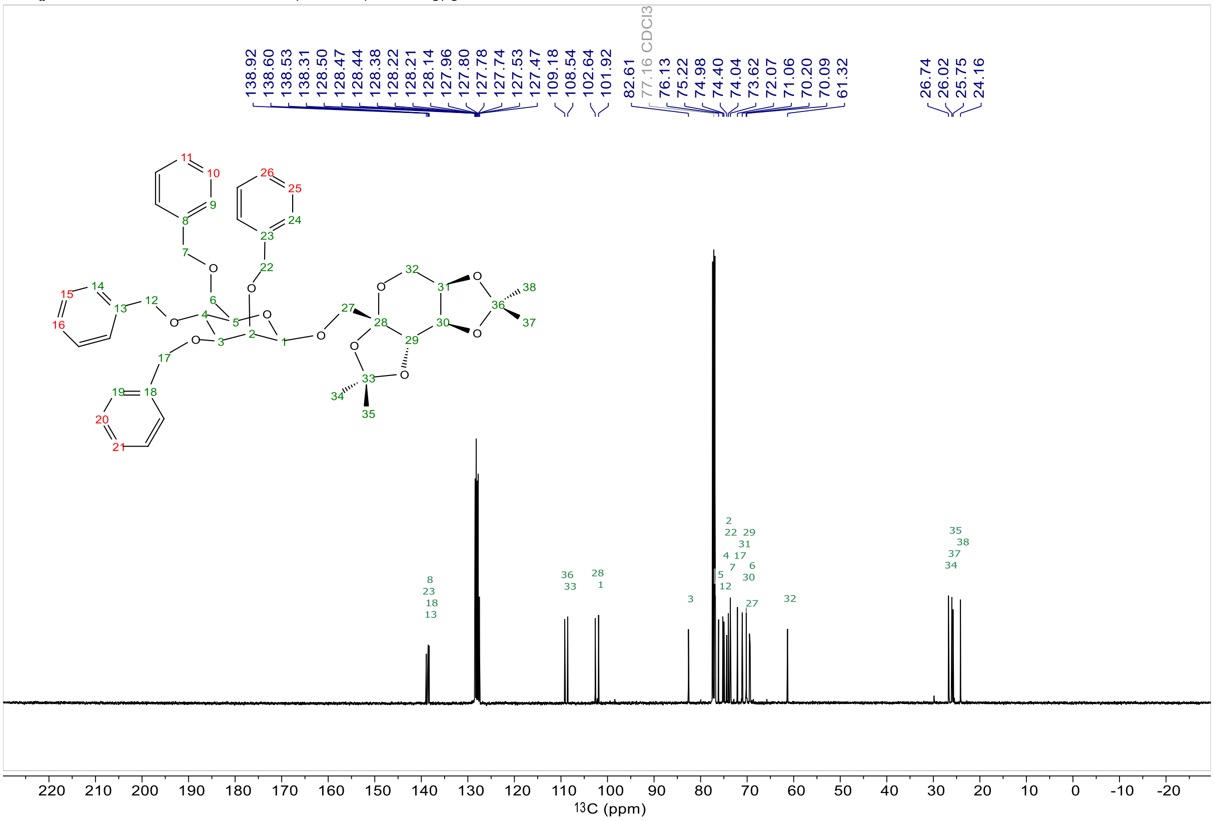

1H{},COSY, 500.81 MHz,CDCl3,298.0K, pulse sequence: cosygpppqf

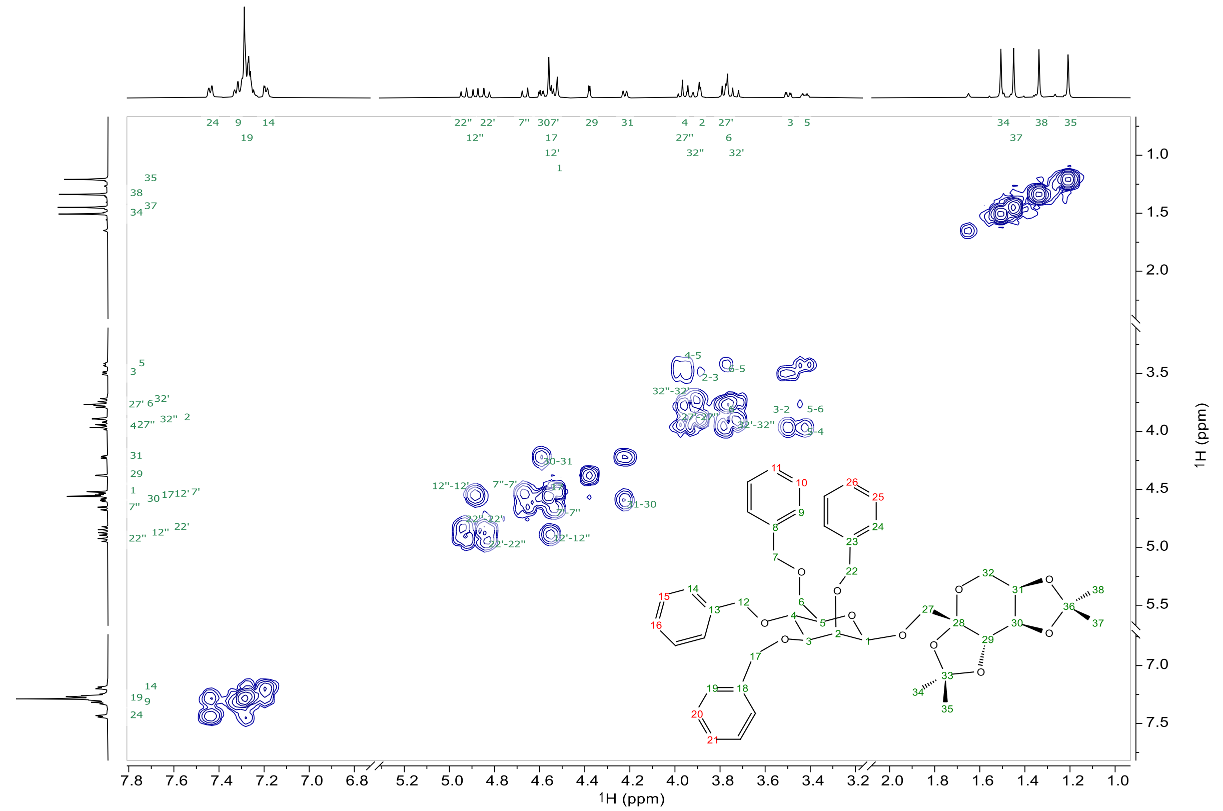

1H{},HSQC-EDITED, 500.81 MHz,CDCl3,298.0K, pulse sequence: hsqcedetgpsisp2.3

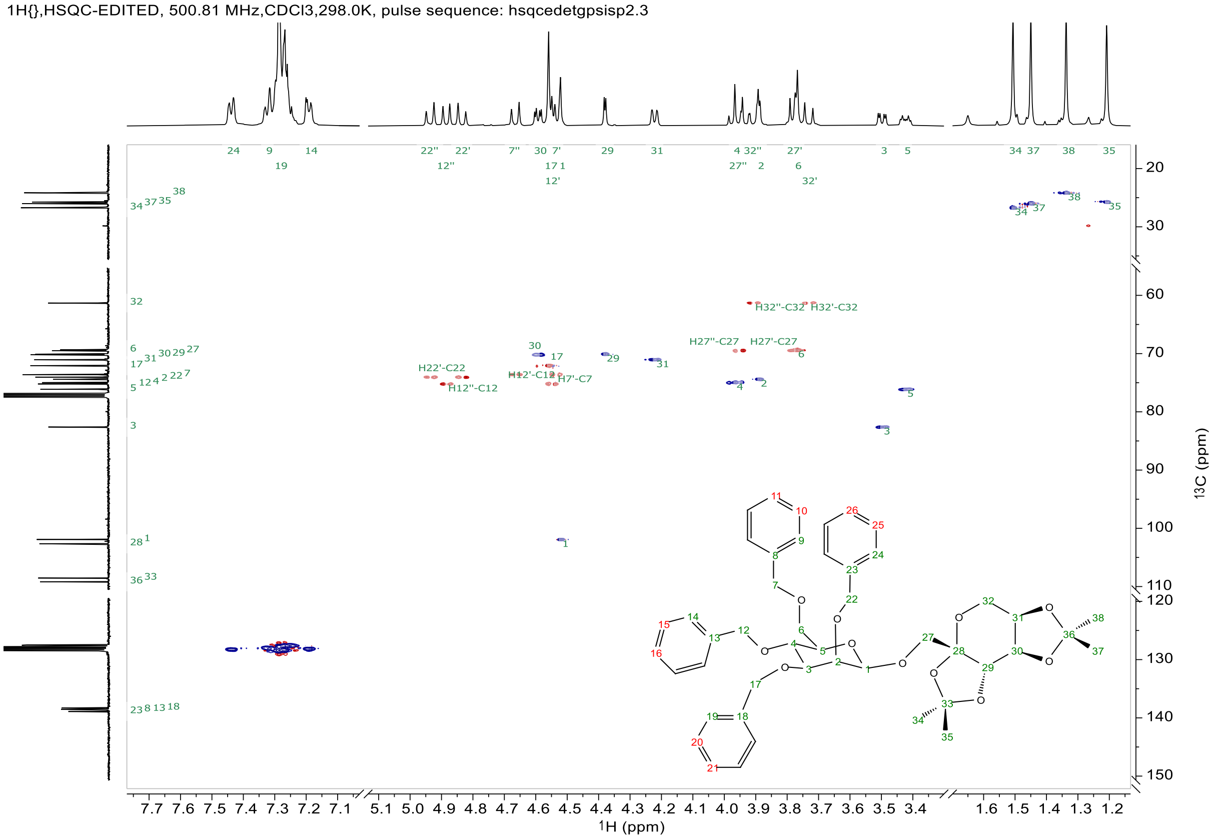

1H{},HMBC, 500.81 MHz,CDCl3,298.0K, pulse sequence: hmbcetgpl3nd

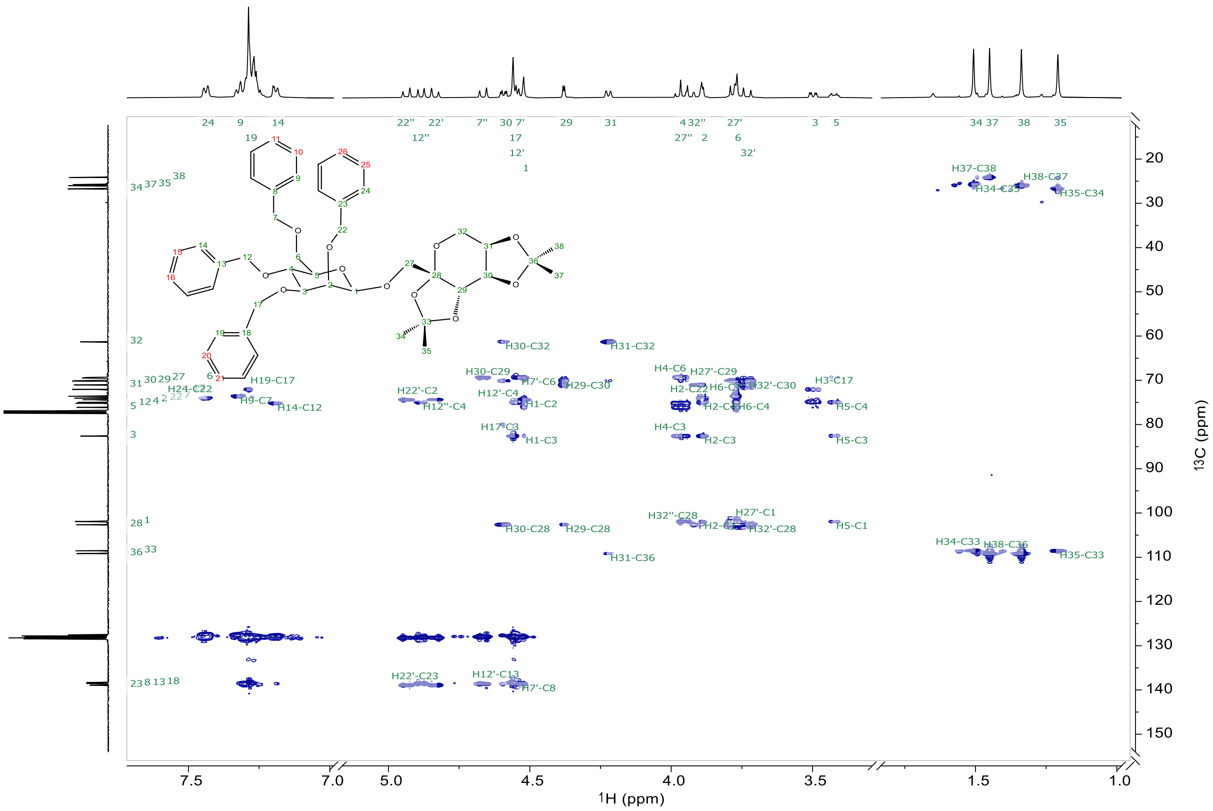

1H{},NOESY, 500.81 MHz,CDCl3,298.0K, pulse sequence: noesygpphpp

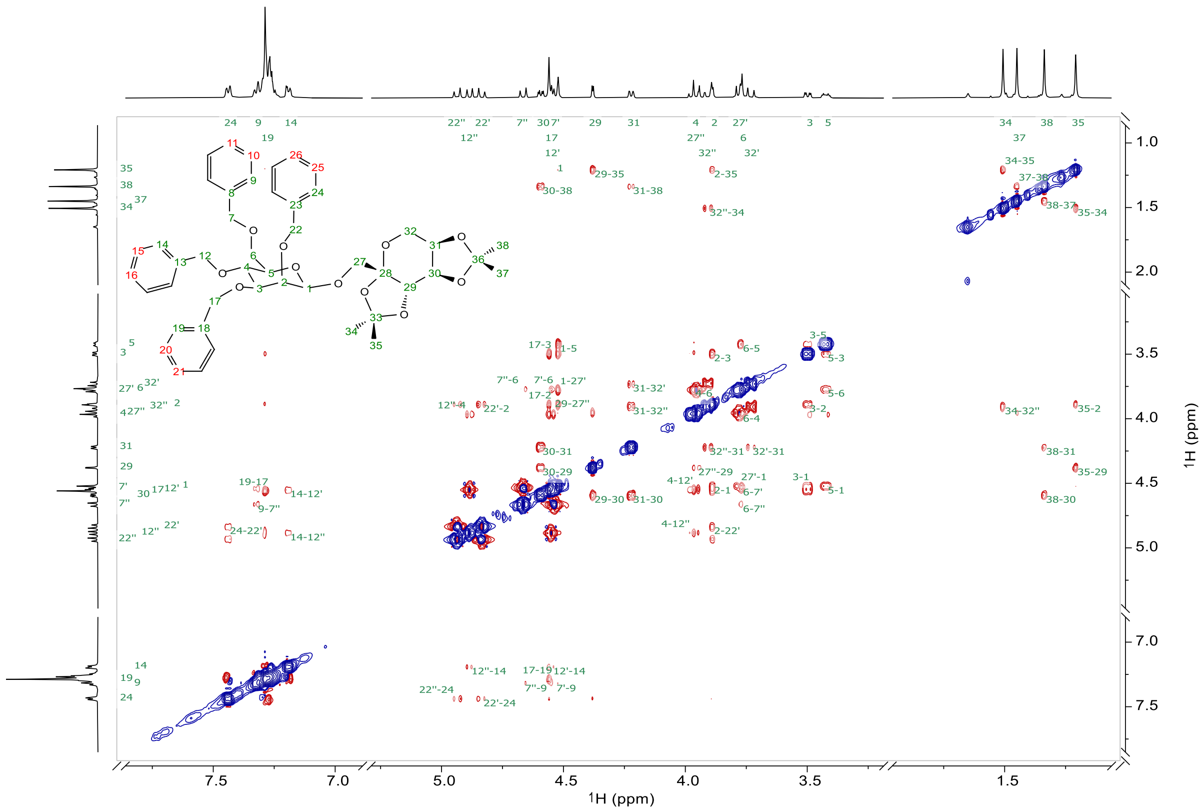

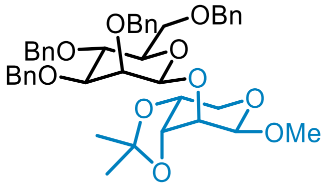

(3a*R*,6*R*,7*S*,7a*R*)-6-methoxy-2,2-dimethyl-7-(((2*S*,3*S*,4*S*,5*R*,6*R*)-3,4,5-tris(benzyloxy)-6-((benzyloxy)methyl)tetrahydro-2*H*-pyran-2-yl)oxy)tetrahydro-4*H*-[1,3]dioxolo[4,5-c]pyran (6c)

Following **General Procedure B**, glycosyl donor **1c** (34 mg, 0.050 mmol) and alcohol **2c** (35 mg, 0.075 mmol) were coupled using (*S*,*S*)-IDPi **3c** (9.4 mg,10 mol%). Reaction time: 72 h. β/α = 93:7 was determined by crude ^{1}H NMR. Flash chromatography (hexane/EtOAc = 6/1 to 3/1) gave **6c** as light-yellow oil (27 mg, 74%).

Following **General Procedure A** on 0.01 mmol scale, the glycosylation was carried out using (*R*,*R*)-IDPi **3c**. β/α = 81:19 was determined by crude ^{1}H NMR.

TLC: R_f = 0.21 (Hexane/EtOAc = 3:1)

^{1}H NMR (600 MHz, CDCl$_3$) δ 7.51 – 7.48 (m, 2H), 7.35 – 7.32 (m, 2H), 7.33 – 7.23 (m, 15H), 7.22 – 7.19 (m, 2H), 4.98 (d, *J* = 12.4 Hz, 1H), 4.91 (d, *J* = 10.6 Hz, 1H), 4.91 (d, *J* = 12.4 Hz, 1H), 4.76 (d, *J* = 3.1 Hz, 1H), 4.65 (d, *J* = 12.1 Hz, 1H), 4.59 (d, *J* = 0.7 Hz, 1H), 4.56 (d, *J* = 12.1 Hz, 1H), 4.56 (d, *J* = 10.8 Hz, 1H), 4.53 (d, *J* = 11.8 Hz, 1H), 4.45 (d, *J* = 11.8 Hz, 1H), 4.43 (t, *J* = 6.2 Hz, 1H), 4.19 (ddd, *J* = 6.1, 2.8, 1.7 Hz, 1H), 4.06 (dd, *J* = 6.2, 3.1 Hz, 1H), 3.98 (dd, *J* = 3.0, 0.7 Hz, 1H), 3.96 (dd, *J* = 12.9, 2.8 Hz, 1H), 3.91 (t, *J* = 9.5 Hz, 1H), 3.86 (dd, *J* = 12.9, 1.7 Hz, 1H), 3.81 – 3.76 (m, 2H), 3.51 (dd, *J* = 9.4, 3.0 Hz, 1H), 3.44 (s, 3H), 3.43 (ddd, *J* = 9.8, 4.4, 3.1 Hz, 1H), 1.51 (s, 3H), 1.33 (s, 3H).

^{13}C NMR (151 MHz, CDCl$_3$) δ 138.99, 138.63, 138.54, 138.33, 128.60, 128.46 (d, *J* = 2.5 Hz), 128.41, 128.17, 127.95, 127.75, 127.69, 127.55, 127.47, 109.27, 100.62, 97.63, 82.37, 76.24, 75.25, 74.96, 74.53, 74.18, 73.94, 73.66, 73.56, 72.95, 71.42, 69.72, 60.88, 55.94, 27.79, 25.94.

$[\alpha]_D^{22}$ = -33.0 (CHCl$_3$, *c* = 1.0).

HRMS m/z (ESI): calcd. for C43 H50 O10 Na1 ([M+Na]$^+$): 749.329619; found: 749.329430.

User Report
LUJ-LA-380-01

The following anomer was assiged in the sample:

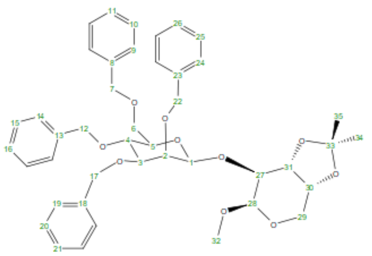

P-ID: ML00001
Measured on: 25/02/2026
CHIFFRE: LUJ-LA-380-01
ELNA#: 16100
Client: Jiaxiang Lu
Group: List
Spectroscopist: Leutzsch
Analysed on: 25/02/2026
Analysed by: Leutzsch
Amount: 15.0 mg
Solvent: CDCl3
Reference: 1H+13C on solvent,other nuclei w/ xiref
Temperature: 298 K
Spectrometer: av600neo
Probe: cryoQCI
Experiments: 1H-zg30, 13C-zgpg30, 1H-13C-hsqcedetgpsisp2.3, 1H-13C-hmbcetgpl3nd, 1H-1H-cosygpppqf, 1H-1H-noesygpphpp, 1H-seldigpzs, 1H-13C-shsqcetgpsisp2.2, 1H-13C-shmbcctetgpl2nd, 1H-13C-hsqcetgpipjcsp.2

Remarks:

The relative stereochemistry at the anomeric center of the major β-anomer is supported by NOE correlations from H1 to the axial positioned protons H3 and H5. The *J*-coupling of H1 to H2 is small (0.7 Hz). Furthermore, the $^{1}J_{CH}$ couplings at the anomeric position 1 were determined based on a CLIP-HSQC. The $^{1}J_{C1\text{-}H1}$ value for the β-anomer is 155.6 Hz. These values are in good agreement with the typical $^{1}J_{CH}$ values for anomeric protons in axial positions of ~ 160 Hz in carbhohydrates (Ref: https://onlinelibrary.wiley.com/doi/pdf/10.1002/cmr.a.10080, https://doi.org/10.1016/S0065-2318(08)60191-2)

The aryl ring signals of the OBn groups were assigned based on cross peaks in band-selective HSQC and HMBC spectra (see later pages of this report).

An overview of all assigments is shown on the next page.

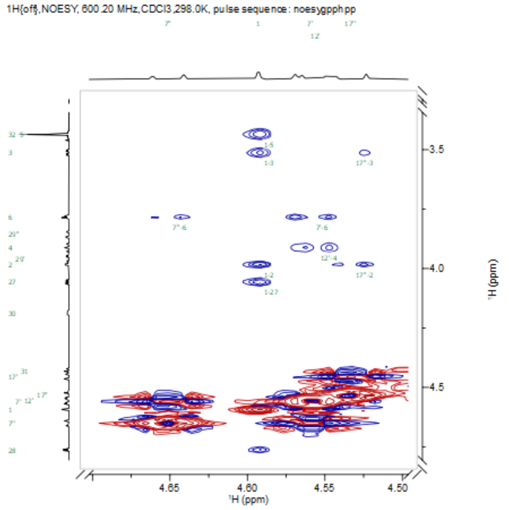

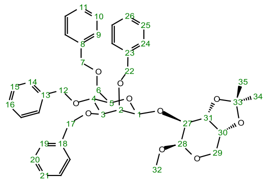

Atom	δ (ppm)	J	COSY	HSQC	HMBC	NOESY
1 C	100.616	155.60(1H)		1	2, 5, 27	
H	4.593	0.70(2), 155.60(1C)	2	1	2, 3, 5, 27	2, 3, 5, 27
2 C	73.662			2	1, 3, 22', 22"	
H	3.983	0.70(1), 3.00(3)	1, 3	2	1, 3, 4, 22	1, 3, 17', 17", 19, 22', 22", 24
3 C	82.375			3	1, 2, 4, 5, 17', 17"	
H	3.513	9.40(4), 3.00(2)	2, 4	3	2, 4, 5, 17	1, 2, 5, 17', 17"
4 C	74.955			4	2, 3, 5, 6', 6", 12', 12"	
H	3.912	9.80(5), 9.40(3)	3, 5	4	3, 5, 6, 12	6', 6", 12', 12"
5 C	76.241			5	1, 3, 4, 6', 6"	
H	3.436	9.80(4), 4.40(6'), 3.10(6")	4, 6', 6"	5	1, 3, 4, 6	1, 3, 6', 6"
6 C	69.720			6', 6"	4, 5, 7', 7"	
H'	3.783	4.40(5)	5, 6"	6	4, 5, 7	4, 5, 7', 7", 9, 14
H"	3.783	3.10(5)	5, 6'	6	4, 5, 7	4, 5, 7', 7", 9, 14
7 C	73.556			7', 7"	6', 6", 9	
H'	4.559	12.10(31)	7", 9	7	6, 8, 9	6', 6", 9
H"	4.651	12.10(31)	7', 9	7	6, 8, 9	6', 6", 9
8 C	138.635				7', 7", 10	
9 C	127.956			9	7', 7", 9	
H	7.338		7', 7", 10	9	7, 9, 11	6', 6", 7', 7"
10 C	128.406			10	10	
H	7.302		9	10	8, 10	
11 C	127.551			11	9	
H	7.264			11		
12 C	75.251			12', 12"	4, 14	
H'	4.556	10.80(12")	12"	12	4, 13, 14	4, 14
H"	4.909	10.80(12')	12'	12	4, 13, 14	4, 14, 19

Atom	δ (ppm)	J	COSY	HSQC	HMBC	NOESY
13 C	138.541				12', 12", 15	
14 C	128.168			14	12', 12", 14	
H	7.202		15	14	12, 14, 16	6', 6", 12', 12"
15 C	128.448			15	15	
H	7.282		14	15	13, 15	
16 C	127.746			16	14	
H	7.262			16		
17 C	71.416			17', 17"	3, 19	
H'	4.455	11.80(17")	17"	17	3, 18, 19	2, 3, 19
H"	4.533	11.80(17')	17'	17	3, 18, 19	2, 3, 19
18 C	138.331				17', 17", 20	
19 C	127.687			19	17', 17", 19	
H	7.282			19	17, 19	2, 12", 17', 17"
20 C	128.464			20	20	
H	7.295			20	18, 20	
21 C	127.683			21		
H	7.273			21		
22 C	73.937			22', 22"	2, 24	
H'	4.907	12.30(7")	22", 24	22	2, 23, 24	2, 24
H"	4.980	12.30(7')	22', 24	22	2, 23, 24	2, 24
23 C	138.989				22', 22", 25	
24 C	128.600			24	22', 22", 24	
H	7.495		22', 22", 25	24	22, 24, 26	2, 22', 22"
25 C	128.175			25	25	
H	7.296		24	25	23, 25	

Atom	δ (ppm)	J	COSY	HSQC	HMBC	NOESY
26 C	127.471			26	24	
H	7.270			26		
27 C	74.534			27	1, 28, 30, 31	
H	4.057	3.10(28), 6.20(31)	28, 31	27	1, 30, 31	1, 28, 31, 34
28 C	97.634	166.10(28H)		28	29', 29", 31, 32	
H	4.763	3.10(27), 166.10(28C)	27	28	27, 29, 31, 32	27, 29", 32
29 C	60.876			29', 29"	28, 30, 31	
H'	3.959	12.90(29"), 2.80(30)	29", 30	29	28, 30	30, 31, 32
H"	3.857	12.90(29'), 1.70(30)	29', 30	29	28, 30, 31	28, 30
30 C	72.951			30	27, 29', 29", 31	
H	4.187	2.80(29'), 1.70(29"), 6.10(31)	29', 29", 31	30	27, 29, 31	29', 29", 31, 35
31 C	74.184			31	27, 28, 29", 30	
H	4.432	6.20(27), 6.10(30)	27, 30	31	27, 28, 29, 30, 33	27, 29', 30, 35
32 C	55.943			32	28	
H3	3.436			32	28	28, 29'
33 C	109.265				31, 34, 35	
34 C	27.787			34	35	
H3	1.506		35	34	33, 35	27, 35
35 C	25.942			35	34	
H3	1.330		34	35	33, 34	30, 31, 34

1H{off},1D, 600.20 MHz,CDCl3,298.0K, pulse sequence: zg30

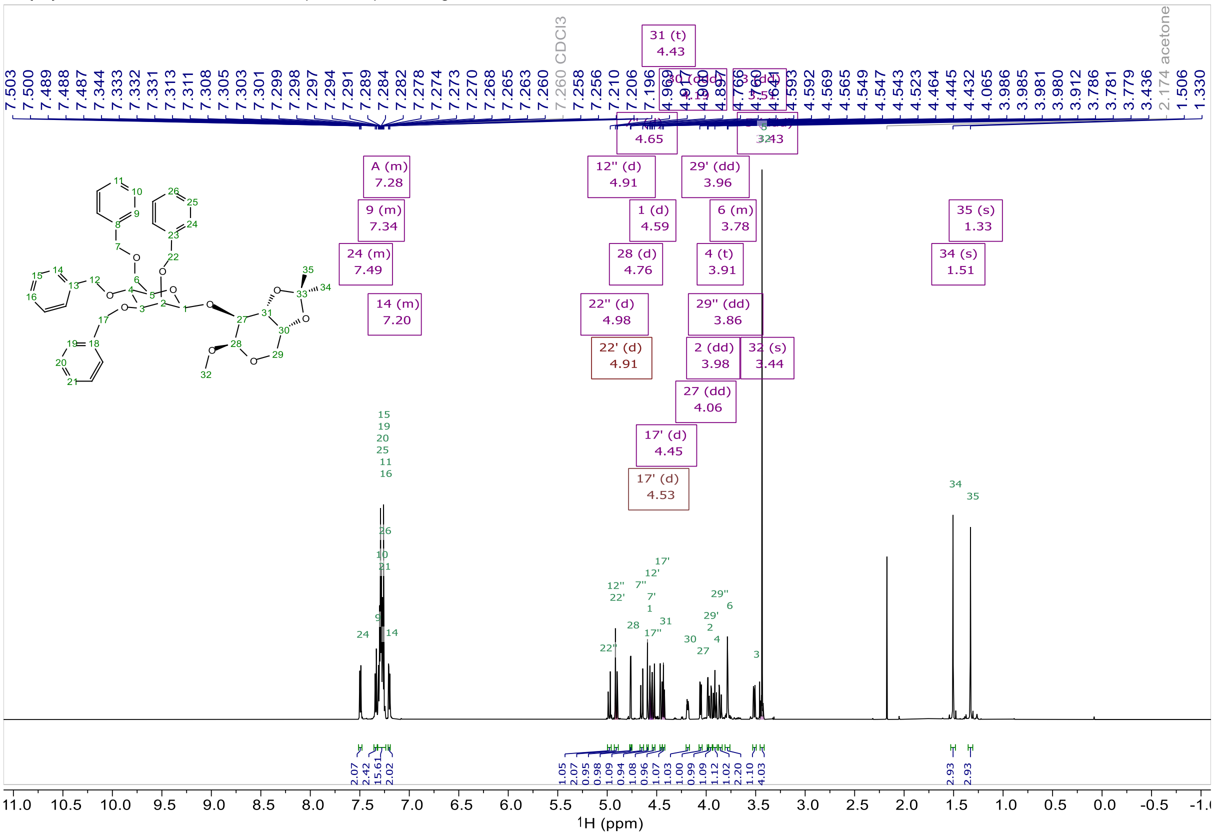

13C{1H},1D, 150.94 MHz,CDCl3,298.0K, pulse sequence: zgpg30

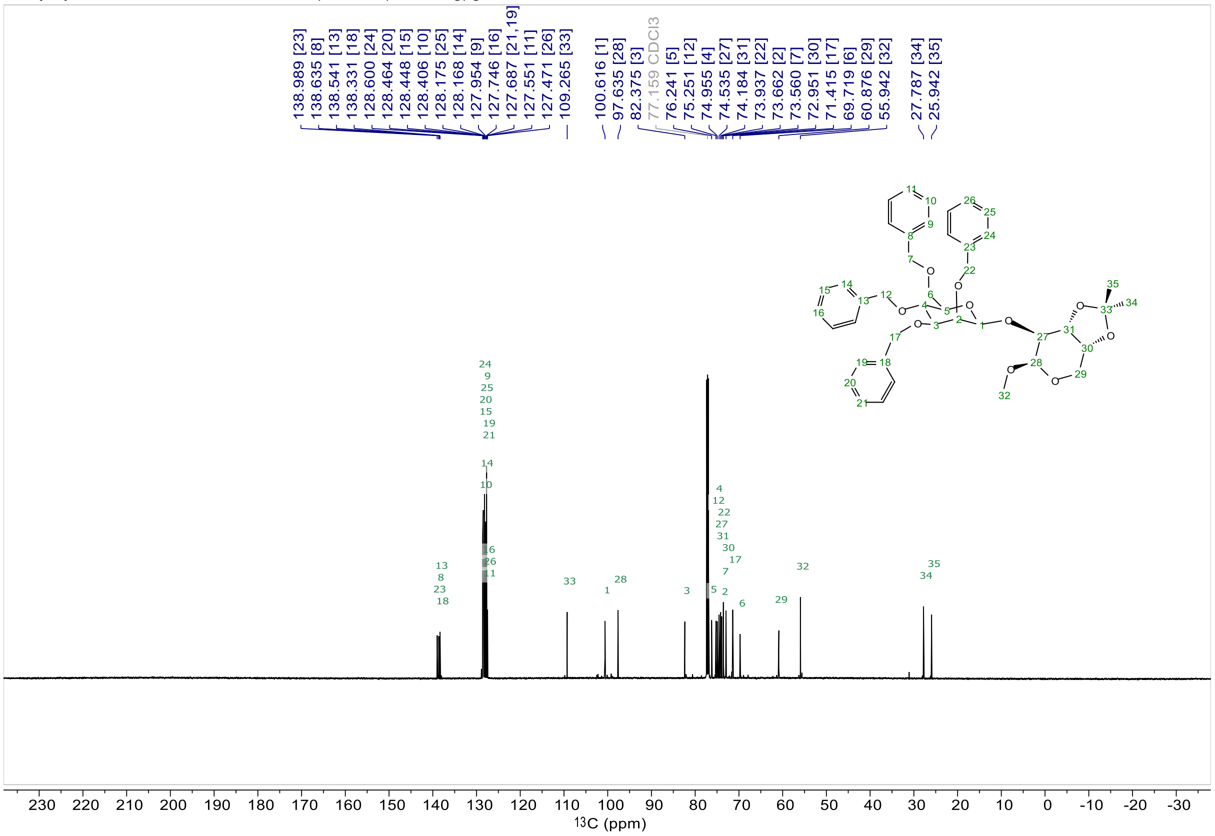

1H{13C},HSQC-EDITED, 600.20 MHz,CDCl3,298.0K, pulse sequence: hsqcedetgpsisp2.3

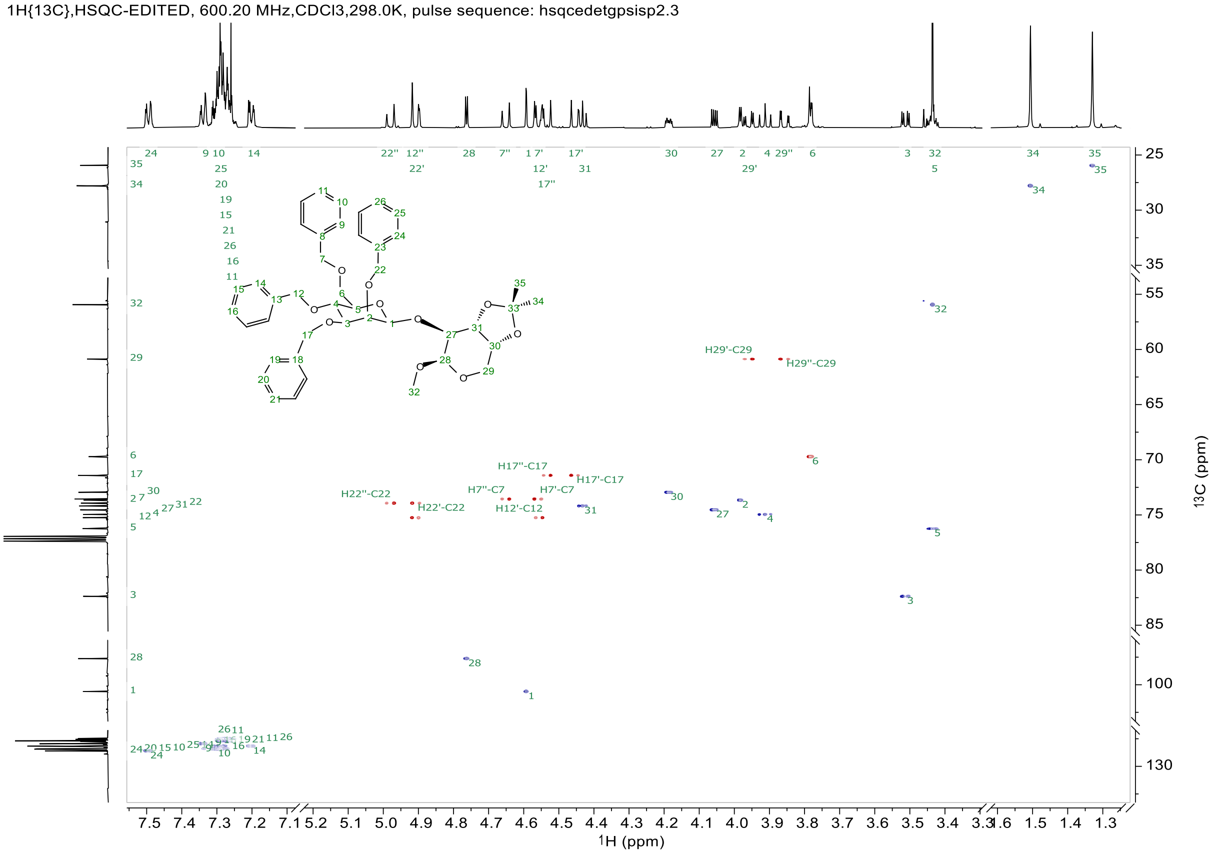

1H{13C},HMBC, 600.20 MHz,CDCl3,298.0K, pulse sequence: hmbcetgpl3nd

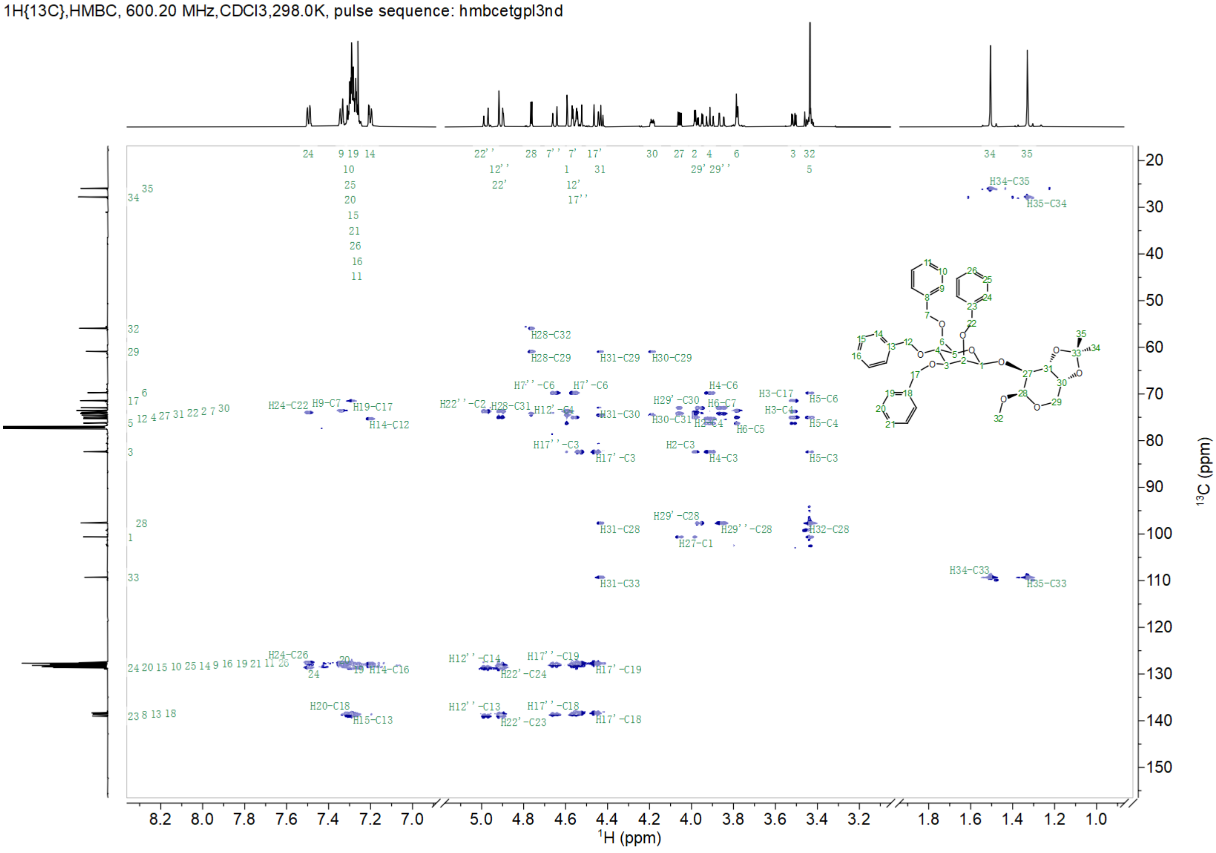

1H{off},COSY, 600.20 MHz,CDCl3,298.0K, pulse sequence: cosygpppqf

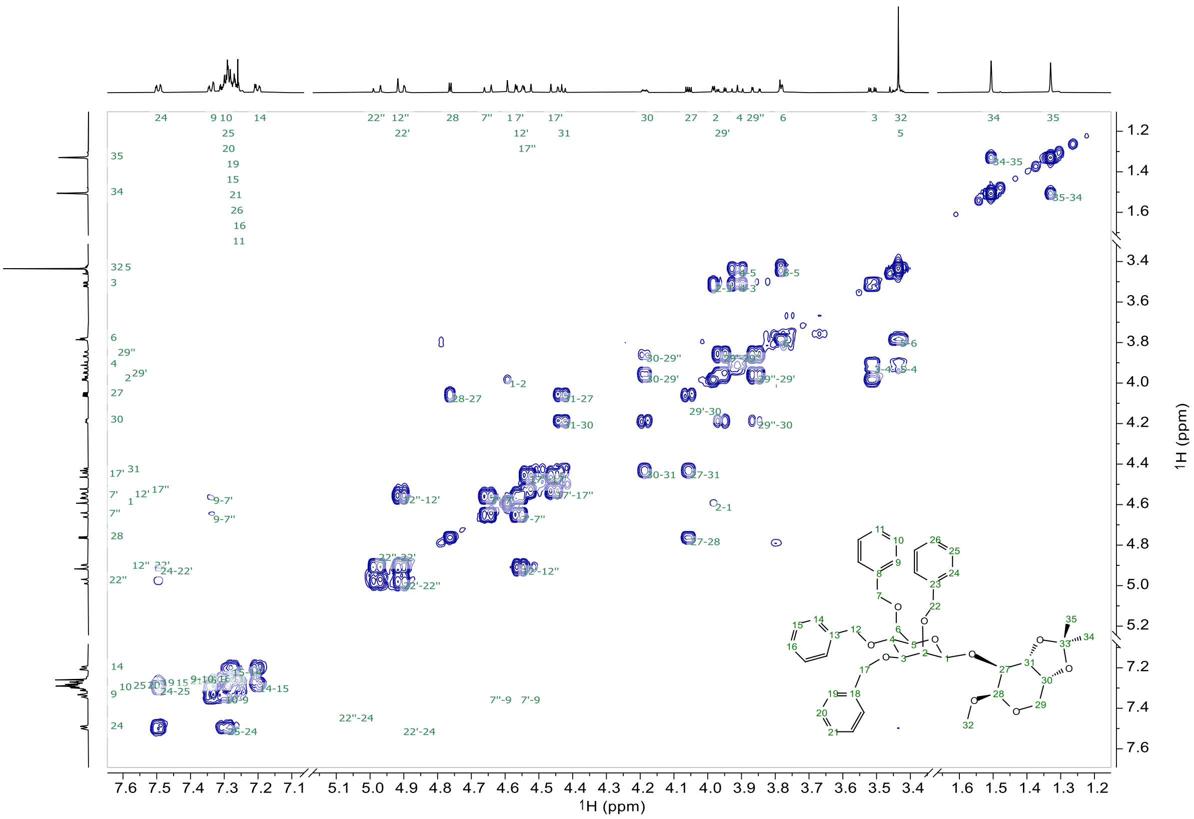

1H{off},NOESY, 600.20 MHz,CDCl3,298.0K, pulse sequence: noesygpphpp

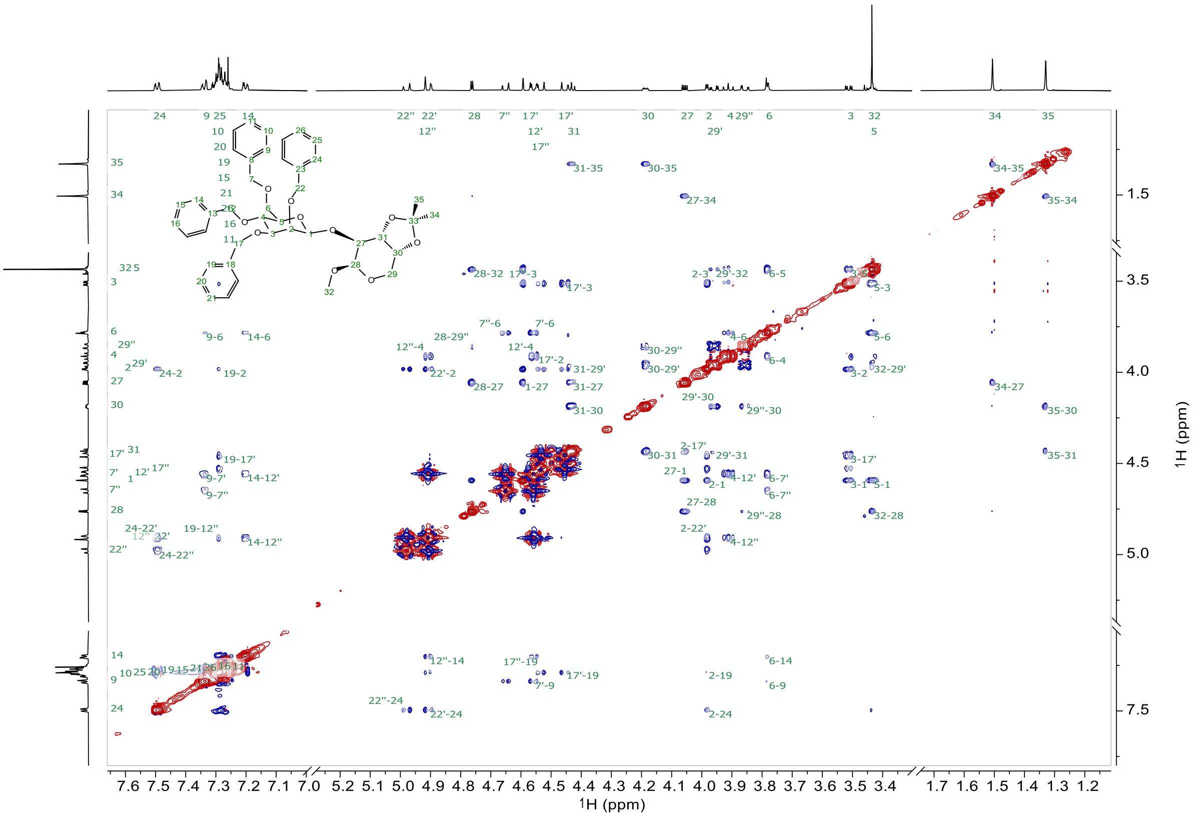

1H{off},1D, 600.20 MHz,CDCl3,298.0K, pulse sequence: seldigpzs

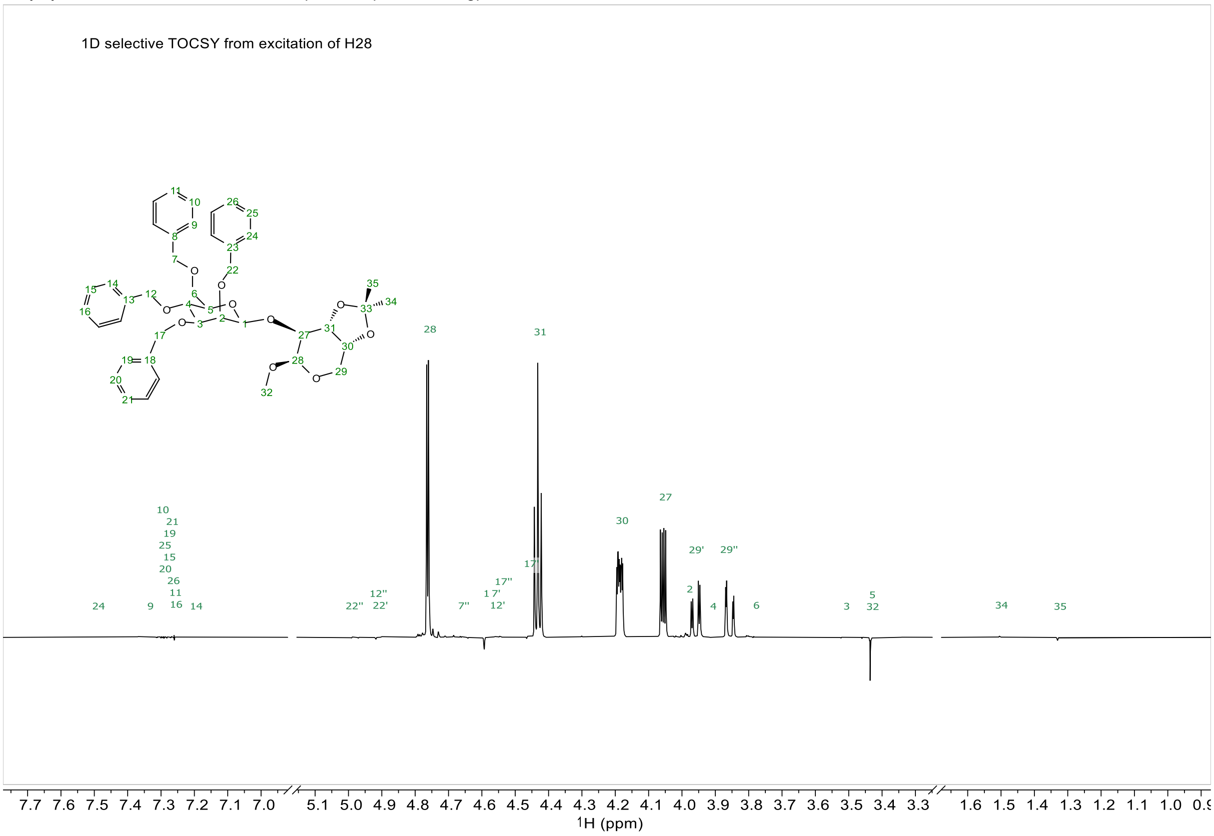

1H{off},1D, 600.20 MHz,CDCl3,298.0K, pulse sequence: seldigpzs

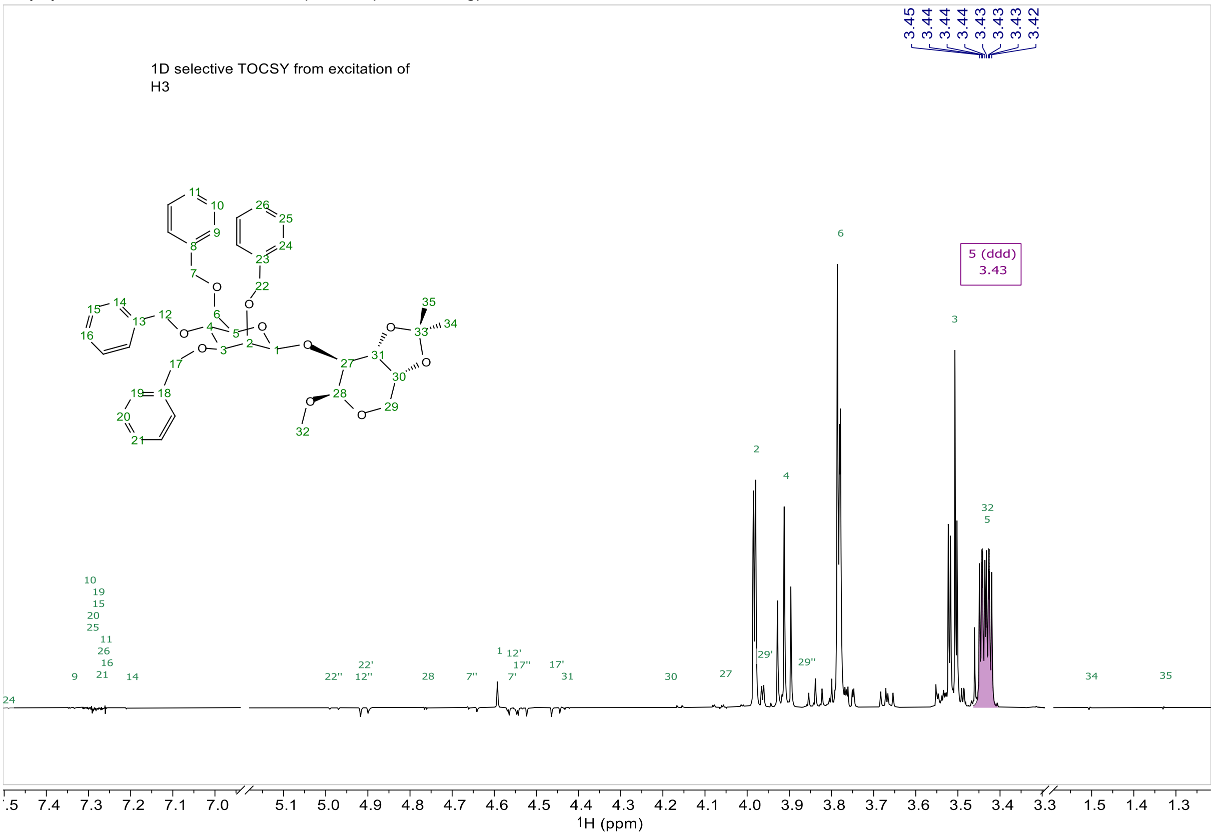

1H{13C},HSQC, 600.20 MHz,CDCl3,298.0K, pulse sequence: shsqcetgpsisp2.2

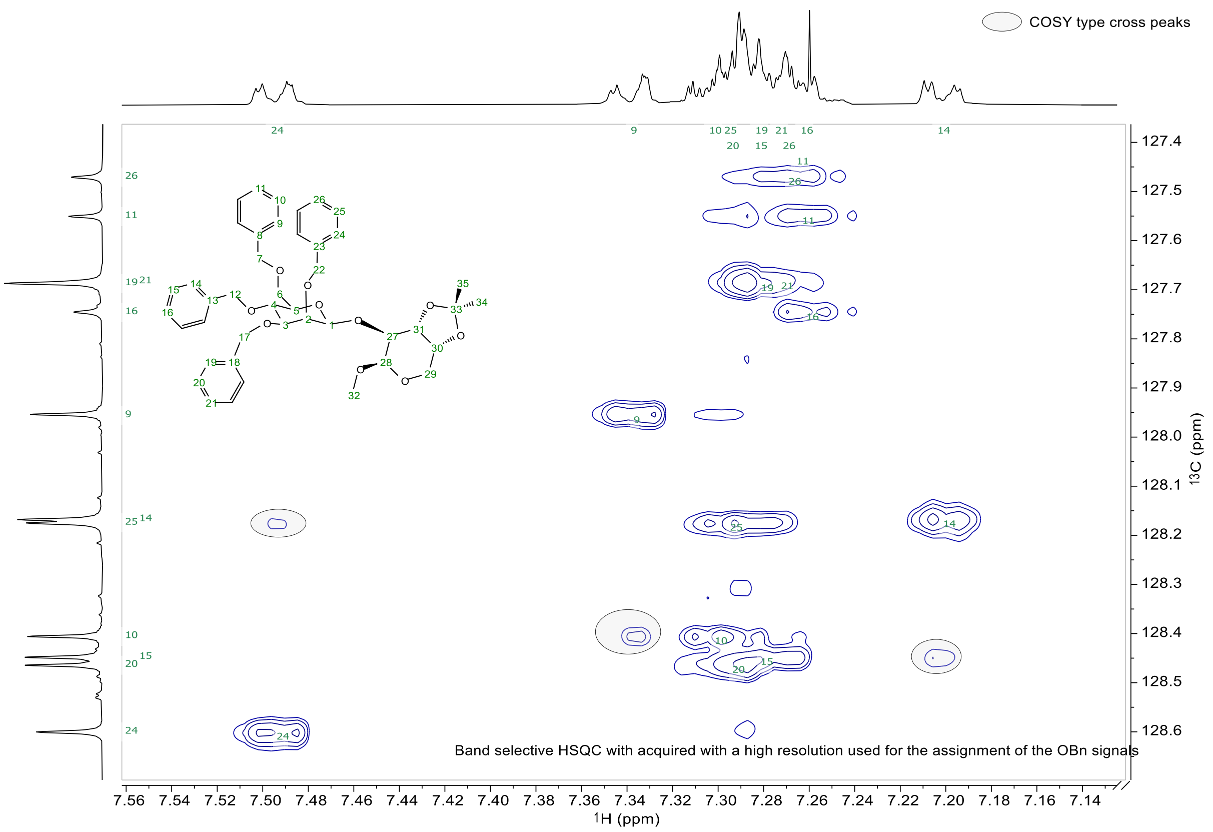

1H{13C},HMBC, 600.20 MHz,CDCl3,298.0K, pulse sequence: shmbcctetgpl2nd

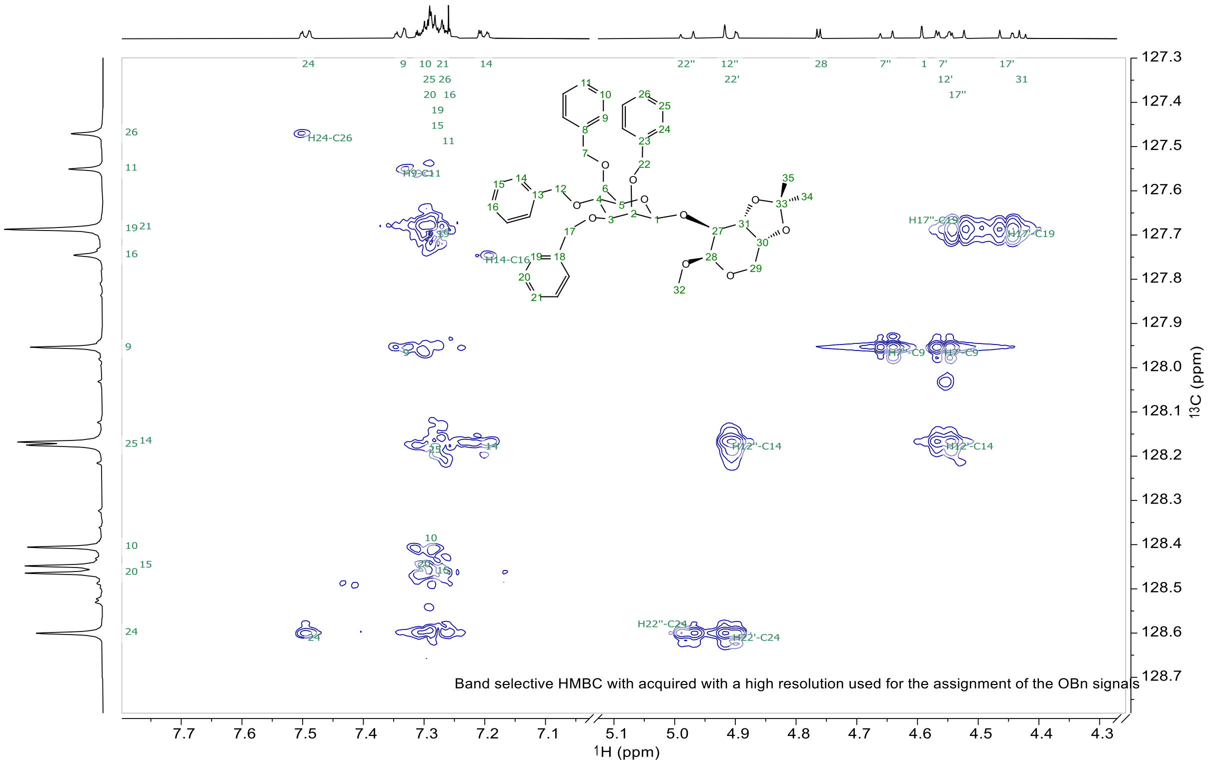

1H{13C},HSQC, 600.20 MHz,CDCl3,298.0K, pulse sequence: hsqcetgpipjcsp.2

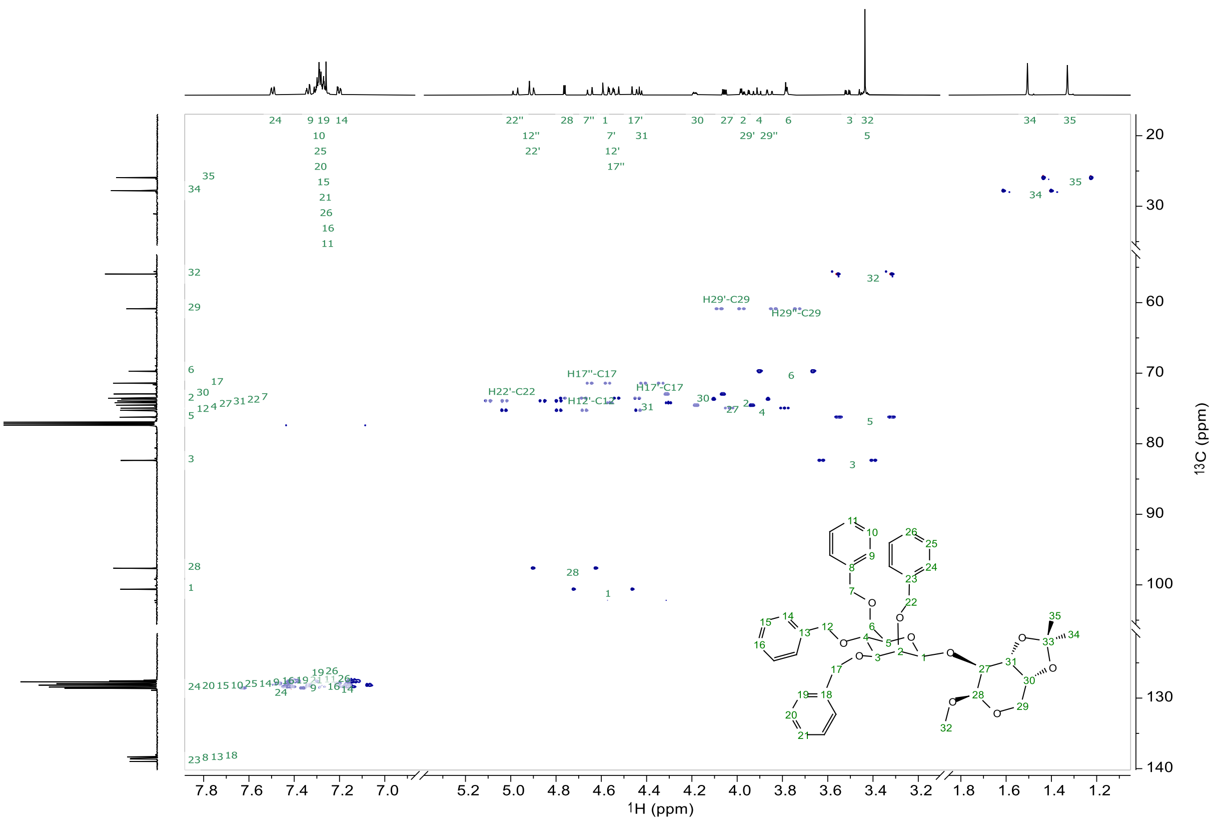

1H{},1D, 600.20 MHz,CDCl3,0K, pulse sequence:

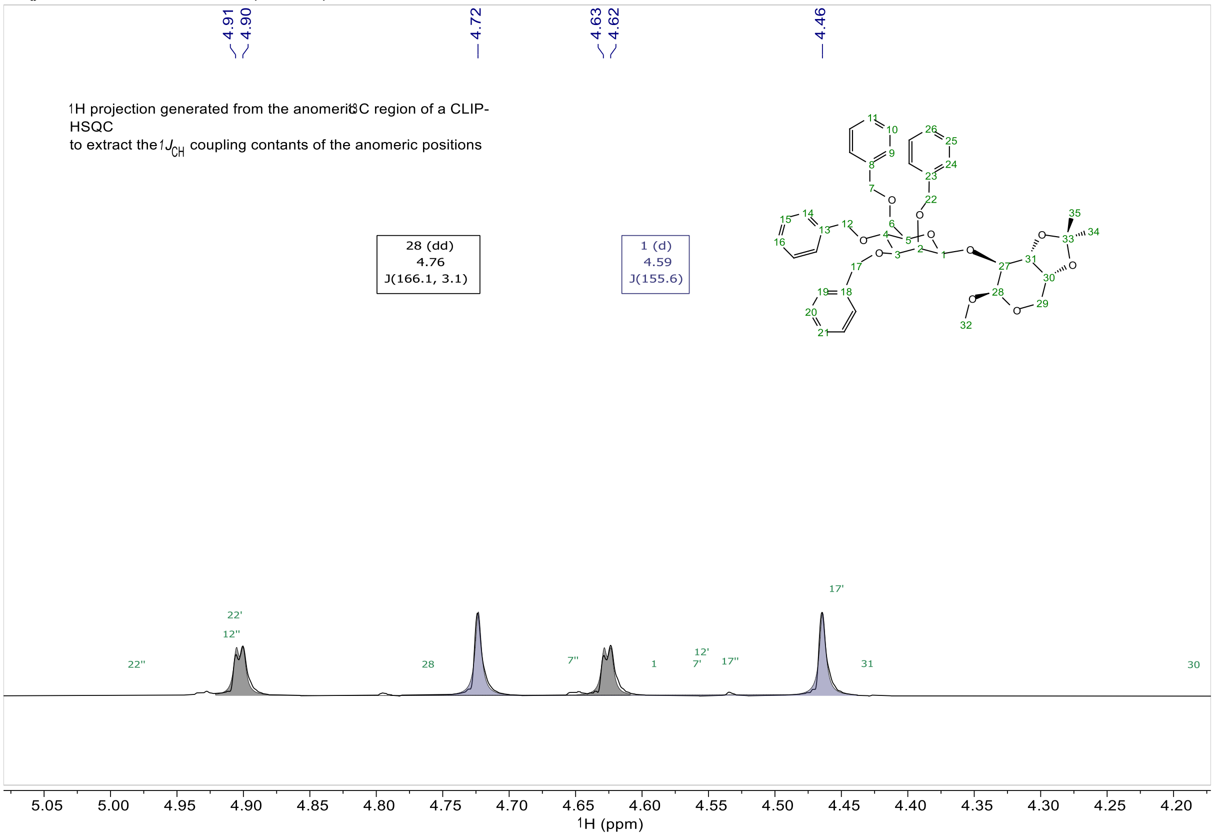

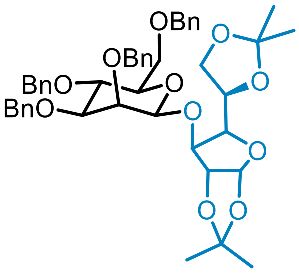

(3a*R*,5*R*,6*S*,6a*R*)-5-((*S*)-2,2-dimethyl-1,3-dioxolan-4-yl)-2,2-dimethyl-6-(((2*R*,4*R*,6*S*)-3,4,5-tris(benzyloxy)-6-((benzyloxy)methyl)tetrahydro-2*H*-pyran-2-yl)oxy)tetrahydrofuro[2,3-d][1,3]dioxole (6d)

Following **General Procedure B**, glycosyl donor **1c** (34 mg, 0.050 mmol) and alcohol **2d** (35 mg, 0.075 mmol) were coupled using (*S*,*S*)-IDPi **3c** (9.4 mg,10 mol%). Reaction time: 72 h. β/α = 80:20 was determined by crude ^{1}H NMR. Flash chromatography (hexane/EtOAc = 6/1 to 4/1) gave **6d** as colorless oil (32 mg, 82%). The NMR data of this compound are consistent with those reported in the literature.[9]

Following **General Procedure A** on 0.01 mmol scale, the glycosylation was carried out using (*R*,*R*)-IDPi **3c**. β/α = 80:20 was determined by crude ^{1}H NMR.

TLC: R_f = 0.33 (Hexane/EtOAc = 3:1)

^{1}H NMR (501 MHz, $CDCl_3$) δ 1.14 (s, 3H), 1.23 (s, 3H), 1.31 (s, 3H), 1.43 (s, 3H), 3.35 (ddd, J = 9.7, 4.7, 2.4 Hz, 1H), 3.44 (dd, J = 9.4, 2.9 Hz, 1H), 3.66 – 3.76 (m, 2H), 3.77 (d, J = 2.9 Hz, 1H), 3.89 (t, J = 9.5 Hz, 1H), 3.97 (dd, J = 8.5, 5.9 Hz, 1H), 4.03 (dd, J = 8.5, 6.5 Hz, 1H), 4.19 – 4.30 (m, 2H), 4.35 – 4.42 (m, 3H), 4.43 – 4.55 (m, 4H), 4.64 (d, J = 11.9 Hz, 1H), 4.71 (d, J = 12.1 Hz, 1H), 4.81 (dd, J = 11.4, 6.2 Hz, 2H), 5.84 (d, J = 3.7 Hz, 1H), 7.14 (dd, J = 7.6, 1.9 Hz, 2H), 7.16 – 7.24 (m, 14H), 7.25 – 7.28 (m, 2H), 7.31 – 7.36 (m, 2H).

^{13}C NMR (126 MHz, $CDCl_3$) *δ* 25.36, 26.45, 26.74, 26.92, 66.25, 69.43, 71.94, 73.41, 73.91, 74.18, 74.56, 74.77, 75.34, 76.62, 80.73, 80.75, 82.49, 83.08, 99.81, 105.19, 108.66, 112.05, 127.62, 127.64, 127.65, 127.79, 127.84, 128.03, 128.23, 128.32, 128.44, 128.50, 128.54, 138.26, 138.42, 138.63, 138.68.

$[\alpha]_D^{22}$ = -12.8 ($CHCl_3$, *c* = 1.0).

HRMS m/z (ESI): calcd. for C46 H54 O11 Na1 ($[M+Na]^+$): 805.355834; found: 805.355210.

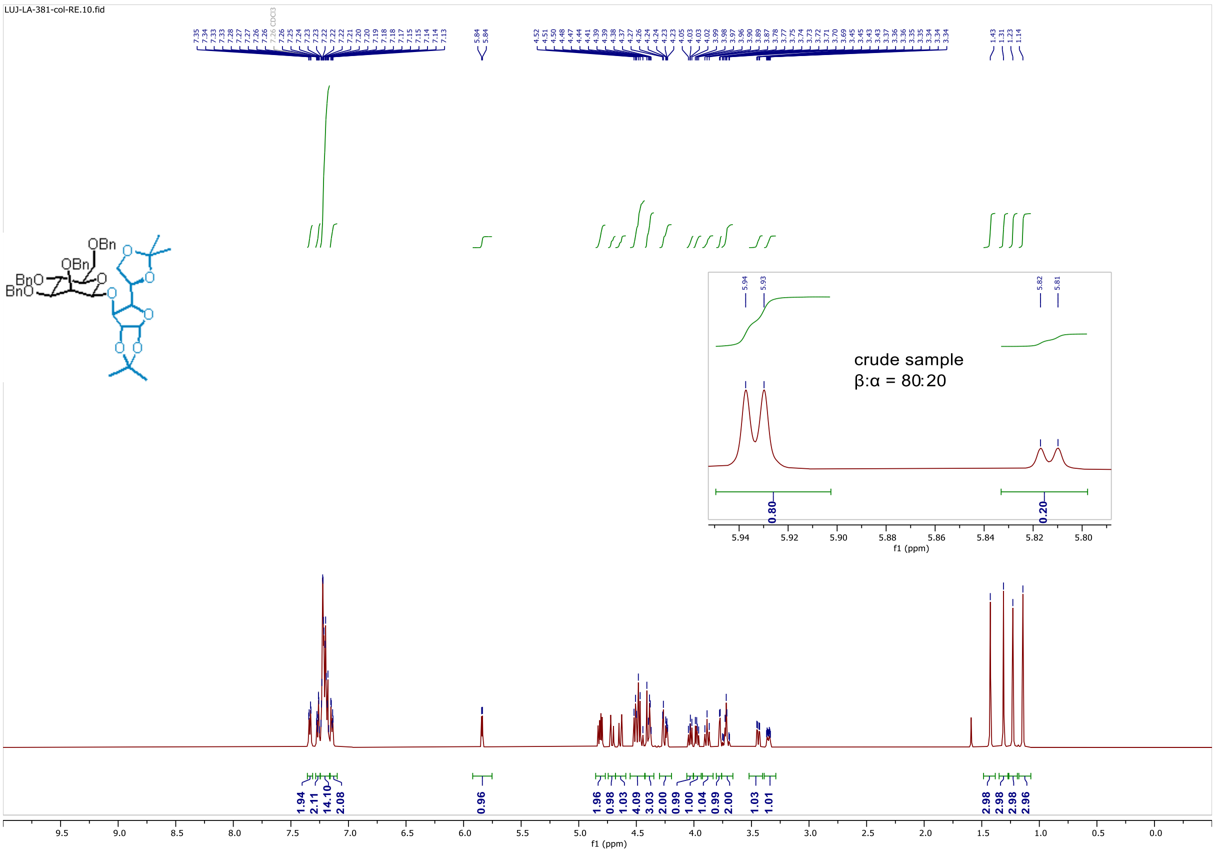
LUJ-LA-381-col-RE.10.fid
crude sample
β:α = 80:20
f1 (ppm)

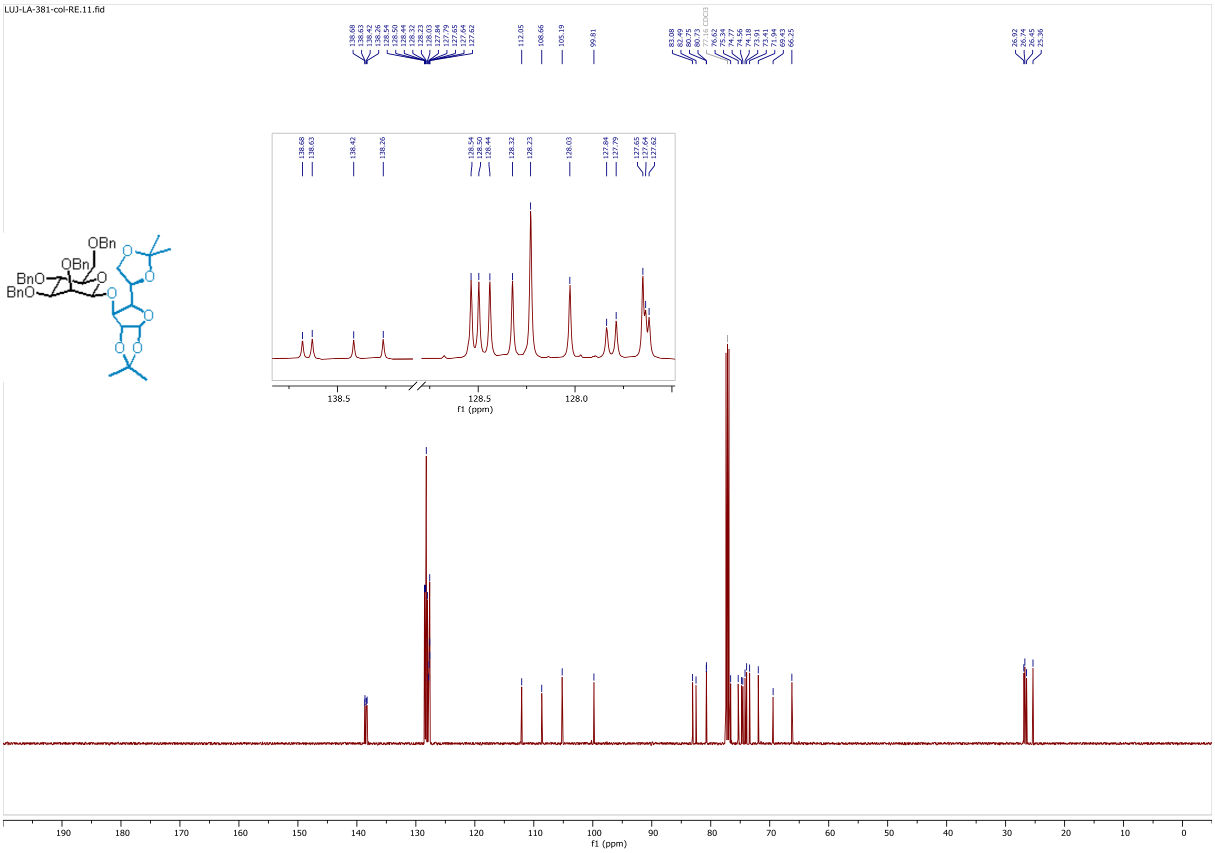
LUJ-LA-381-col-RE.11.fid
f1 (ppm)

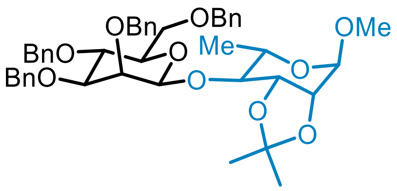

(3a*R*,4*R*,6*S*,7*S*,7a*R*)-4-methoxy-2,2,6-trimethyl-7-(((2*S*,3*S*,4*S*,5*R*,6*R*)-3,4,5-tris(benzyloxy)-6-((benzyloxy)methyl)tetrahydro-2*H*-pyran-2-yl)oxy)tetrahydro-4*H*-[1,3]dioxolo[4,5-c]pyran (6e)

Following **General Procedure B**, glycosyl donor **1c** (34 mg, 0.050 mmol) and alcohol **2e** (35 mg, 0.075 mmol) were coupled using (*S*,*S*)-IDPi **3c** (9.4 mg,10 mol%). Reaction time: 72 h. β/α = 78:22 was determined by crude ^{1}H NMR. Flash chromatography (hexane/EtOAc = 6/1 to 4/1) gave **6e** as colorless oil (27 mg, 73%).

Following **General Procedure A** on 0.01 mmol scale, the glycosylation was carried out using (*R*,*R*)-IDPi **3c**. β/α = 64:36 was determined by crude ^{1}H NMR.

TLC: R_f = 0.63 (Hexane/EtOAc = 3:1)

^{1}H NMR (600 MHz, CDCl$_3$) δ 7.5 – 7.4 (m, 2H), 7.4 – 7.4 (m, 1H), 7.4 – 7.3 (m, 2H), 7.3 – 7.2 (m, 12H), 7.3 – 7.2 (m, 2H), 7.2 (dd, *J* = 7.8, 1.7 Hz, 0H), 4.9 (d, *J* = 12.3 Hz, 1H), 4.9 (d, *J* = 1.7 Hz, 0H), 4.9 (d, *J* = 10.8 Hz, 1H), 4.9 (s, 1H), 4.9 (d, *J* = 10.7 Hz, 0H), 4.9 (s, 1H), 4.8 (s, 0H), 4.8 (d, *J* = 12.3 Hz, 1H), 4.8 (d, *J* = 12.6 Hz, 0H), 4.7 (d, *J* = 12.1 Hz, 0H), 4.7 (d, *J* = 12.0 Hz, 1H), 4.7 (d, *J* = 12.6 Hz, 0H), 4.7 (d, *J* = 11.7 Hz, 0H), 4.6 (d, *J* = 11.7 Hz, 0H), 4.6 (d, *J* = 10.8 Hz, 1H), 4.6 (d, *J* = 12.0 Hz, 1H), 4.6 (d, *J* = 11.6 Hz, 1H), 4.5 (d, *J* = 12.1 Hz, 0H), 4.5 (d, *J* = 11.8 Hz, 1H), 4.2 (t, *J* = 9.8 Hz, 0H), 4.2 (dd, *J* = 7.5, 5.5 Hz, 1H), 4.1 (dd, *J* = 5.5, 0.7 Hz, 1H), 4.0 (dd, *J* = 5.6, 0.7 Hz, 0H), 4.0 (dt, *J* = 9.9, 2.9 Hz, 0H), 4.0 (dd, *J* = 7.5, 5.6 Hz, 0H), 4.0 (d, *J* = 3.1 Hz, 1H), 3.9 (t, *J* = 9.6 Hz, 1H), 3.9 (dd, *J* = 10.8, 2.9 Hz, 0H), 3.8 (dd, *J* = 11.3, 5.2 Hz, 1H), 3.8 (dd, *J* = 11.3, 1.8 Hz, 1H), 3.7 (dd, *J* = 10.0, 7.5 Hz, 1H), 3.7 (dd, *J* = 10.8, 1.9 Hz, 0H), 3.7 (dq, *J* = 10.0, 6.2 Hz, 1H), 3.6 (dd, *J* = 9.4, 3.1 Hz, 1H), 3.5 (dq, *J* = 10.0, 6.3 Hz, 0H), 3.4 (ddd, *J* = 9.8, 5.2, 1.8 Hz, 1H), 3.4 (s, 2H), 3.3 – 3.3 (m, 1H), 3.3 (dd, *J* = 10.0, 7.5 Hz, 0H), 1.5 (s, 2H), 1.4 (d, *J* = 6.2 Hz, 2H), 1.3 (s, 2H), 1.0 (d, *J* = 6.3 Hz, 1H)..

^{13}C NMR (151 MHz, CDCl$_3$) δ 17.42, 17.85, 26.45, 26.59, 27.98, 28.25, 54.92, 55.03, 64.50, 64.83, 68.75, 69.55, 71.42, 71.90, 72.46, 72.73, 73.59, 73.65, 74.04, 74.18, 74.36, 74.84, 75.05, 75.27, 75.33, 75.99, 76.22, 76.31, 76.89, 77.80, 78.75, 80.18, 80.32, 82.57, 98.02, 98.11, 99.01, 100.19, 109.16, 109.52, 127.45, 127.52, 127.54, 127.66, 127.67, 127.71, 127.73, 127.82, 127.88, 128.01, 128.15, 128.18, 128.19, 128.21, 128.22, 128.37, 128.41, 128.42, 128.45, 128.47, 128.49, 128.49, 138.37, 138.41, 138.61, 138.63, 138.75, 138.76, 138.85, 139.08.

$[\alpha]_D^{22}$ = -34.4 (CHCl$_3$, *c* = 1.0).

HRMS m/z (ESI): calcd. for C44 H52 O10 Na1 ([M+Na]$^+$): 763.345269; found: 763.344770.

User Report
LUJ-LA-382-01

The following anomers (ratio ~ 1:0.3) were assiged in the sample:

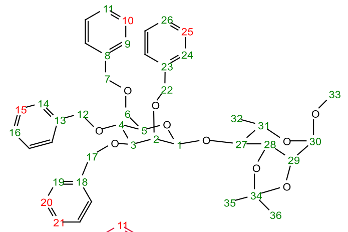

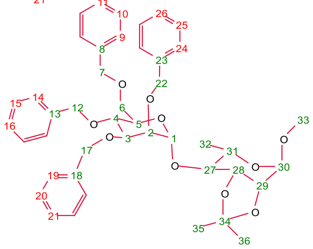

P-ID: ML00001, ML00002
Measured on: 23/02/2026
CHIFFRE: LUJ-LA-382-01
ELNA#: 16087
Client: Jiaxiang Lu
Group: List
Spectroscopist: Leutzsch
Analysed on: 25/02/2026
Analysed by: Leutzsch
Amount: 15.0 mg
Solvent: CDCl3
Reference: 1H+13C on solvent,other nuclei w/ xiref
Temperature: 298 K
Spectrometer: av600a
Probe: cryoBBO
Experiments: 1H-zg30, 1H-zg30, 13C-zgpg30, 1H-13C-hsqcedetgpsisp2.3, 1H-13C-hmbcetgpl3nd, 1H-1H-cosygpppqf, 1H-1H-noesygpph, 13C-deptsp135, 1H-13C-hsqcetgpipjcsp.2, 1H-, 1H-selcssfdizs

Remarks:

The relative stereochemistry at the anomeric center of the major β-anomer is supported by NOE correlations from H1 to the axial positioned protons H3 and H5. The J-coupling of H1 to H2 could not be resolved and is therefore likely <1Hz. In contrast to this, the α-anomer lacks the NOEs to H3 & H5 and a J-coupling between H1 and H2 of approx. 1.7 Hz is observed. Furthermore, the $^{1}J_{CH}$ couplings at the anomeric position 1 were determined based on a CLIP-HSQC. The $^{1}J_{C1\text{-}H1}$ value for the β-anomer is 157.8 Hz and for the α-anomer 169.3 Hz. These values are in excellent agreement with the typical $^{1}J_{CH}$ values for anomeric protons in equatorial position of ~ 170 Hz and in axial positions of ~ 160 Hz in carbhohydrates (Ref: https://onlinelibrary.wiley.com/doi/pdf/10.1002/cmr.a 10080, https://doi.org/10.1016/S0065-2318(08)60191-2)

The aryl ring signals were not assigned in detail due to a lack of resolution and significant overlap in the dataset.

An overview of all assigments is shown on the next 2 pages.

1H{off},NOESY, 600.22 MHz,CDCl3,298.0K, pulse sequence: noesygpph

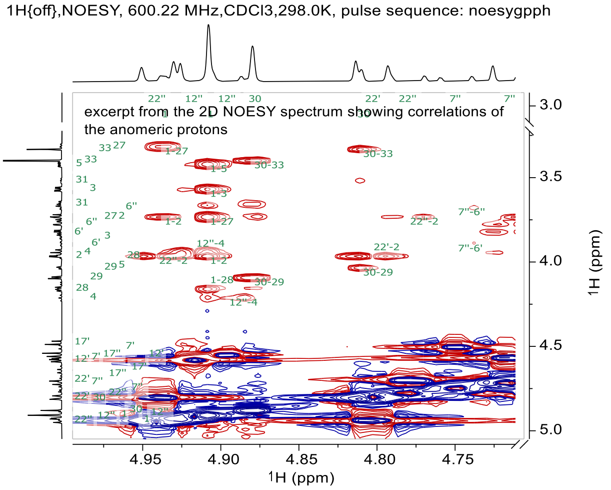

Atom	δ (ppm)	J	COSY	HSQC	HMBC	NOESY
1 C	100.188	157.80(1H)		1	2, 5, 27	
H	4.908	157.80(1C)	2	1	2, 3, 5, 27	2, 3, 5, 27, 28, 35
2 C	74.355			2	1, 3, 22', 22''	
H	3.966	3.10(3)	1, 3	2	1, 3, 4, 22	1, 3, 17', 17'', 19, 22', 22'', 24
3 C	82.567			3	1, 2, 4, 5, 17', 17''	
H	3.569	9.40(4), 3.10(2)	2, 4	3	2, 4, 5, 17	1, 2, 5, 17', 17''
4 C	75.050			4	2, 3, 5, 6', 6'', 12', 12''	
H	3.945	9.80(5), 9.40(3)	3, 5	4	3, 5, 6, 12	6', 6'', 7'', 12', 12''
5 C	76.305			5	1, 3, 4, 6', 6''	
H	3.422	9.80(4), 1.80(6''), 5.20(6')	4, 6', 6''	5	1, 3, 4	1, 3, 6', 6''
6 C	69.546			6', 6''	4, 7', 7''	
H'	3.827	5.20(5), 11.30(6'')	5, 6''	6	4, 5, 7	4, 5, 7', 7''
H''	3.770	1.80(5), 11.30(6')	5, 6'	6	4, 5, 7	4, 5, 7', 7''
7 C	73.651			7', 7''	6', 6'', 9	
H'	4.568	12.10(7'')	7''	7	6, 8	6', 6'', 9
H''	4.715	12.10(7')	7'	7	6, 8	4, 6', 6'', 9
8 C	138.747				7', 7''	
9 C	127.881					
H	7.338				7	7', 7''
10 C						
H						
11 C	127.521					
H						
12 C	75.273			12', 12''	4, 14	
H'	4.582	10.80(12'')	12''	12	4, 13	4, 14
H''	4.917	10.80(12')	12'	12	4, 13	4, 14

Atom	δ (ppm)	J	COSY	HSQC	HMBC	NOESY
13 C	138.606				12', 12''	
14 C	128.152					
H	7.227				12	12', 12''
15 C						
H						
16 C	127.729					
H						
17 C	71.425			17', 17''	3, 19	
H'	4.479	11.80(17'')	17''	17	3, 18	2, 3, 19
H''	4.550	11.80(17')	17'	17	3, 18	2, 3, 19
18 C	138.411				17', 17''	
19 C	127.674					
H	7.306				17	2, 17', 17''
20 C						
H						
21 C						
H						
22 C	74.036			22', 22''	2, 24	
H'	4.803	12.30(22'')	22''	22	2, 23	2, 24
H''	4.941	12.30(22')	22'	22	2, 23	2, 24
23 C	139.077				22', 22''	
24 C	128.179					
H	7.438				22	2, 22', 22''
25 C						
H						

Atom	δ (ppm)	J	COSY	HSQC	HMBC	NOESY
26 C	127.453					
H						
27 C	77.796			27	1, 28, 29, 31, 32	
H	3.735	10.00(31), 7.50(28)	28, 31	27	1, 28, 31, 32	1, 32, 35
28 C	78.751			28	27, 29, 30, 31	
H	4.161	5.50(29), 7.50(27)	27, 29, 30, 35	28	27, 34	1, 31
29 C	76.225			29	30	
H	4.093	0.70(30), 5.50(28)	28, 30	29	27, 28	30, 36
30 C	98.025	167.50(30H)		30	31, 33	
H	4.880	0.70(29), 167.50(30C)	28, 29, 31	30	28, 29, 31, 33	29, 33
31 C	64.485			31	27, 30, 32	
H	3.655	6.20(32), 10.00(27)	27, 30, 32	31	27, 28, 30, 32	28, 32, 33
32 C	17.848			32	27, 31	
H3	1.361	6.20(31)	31	32	27, 31	27, 31
33 C	55.034			33	30	
H3	3.400			33	30	30, 31
34 C	109.516				28, 35, 36	
35 C	27.979			35	36	
H3	1.468		28	35	34, 36	1, 27
36 C	26.593			36	35	
H3	1.325			36	34, 35	29

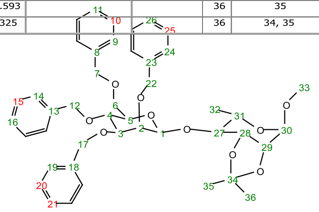

Atom	δ (ppm)	J	COSY	HSQC	HMBC	NOESY
1 C	99.013	169.30(1H)		1	2, 5, 27	
H	4.937	1.70(2), 169.30(1C)	2	1	2, 3, 5, 27	2, 27, 32
2 C	74.180			2	1, 3, 22', 22"	
H	3.734	3.20(3), 1.70(1)	1, 3	2	1, 3, 4, 22	1, 3, 17', 17", 22', 22"
3 C	80.318			3	1, 2, 4, 5, 17', 17"	
H	3.851	9.40(4), 3.20(2)	2, 4	3	2, 4, 5, 17	2, 5, 17', 17"
4 C	74.839			4	2, 3, 5, 6", 12', 12"	
H	4.213	9.40(3), 9.90(5)	3, 5	4	3, 5, 6, 12	6', 6", 12', 12"
5 C	71.905			5	1, 3, 4, 6', 6"	
H	4.023	9.90(4), 2.90(6'), 1.90(6")	4, 6', 6"	5	1, 3, 4	3, 6', 6"
6 C	68.754			6', 6"	4, 7', 7"	
H'	3.893	10.80(6"), 2.90(5)	5, 6"	6	5, 7	4, 5, 7', 7"
H"	3.678	10.80(6'), 1.90(5)	5, 6'	6	4, 5, 7	4, 5, 7', 7"
7 C	73.588			7', 7"	6', 6"	
H'	4.504	12.10(7")	7"	7	6, 8	6', 6"
H"	4.749	12.10(7')	7'	7	6, 8	6', 6"
8 C	138.632				7', 7"	
9 C						
H						
10 C						
H						
11 C						
H						
12 C	75.328			12', 12"	4	
H'	4.550	10.70(12")	12"	12	4, 13	4
H"	4.896	10.70(12')	12'	12	4, 13	4

Atom	δ (ppm)	J	COSY	HSQC	HMBC	NOESY
13 C	138.845				12', 12"	
14 C						
H						
15 C						
H						
16 C						
H						
17 C	72.463			17', 17"	3	
H'	4.628	11.70(17")	17"	17	3, 18	2, 3
H"	4.666	11.70(17')	17'	17	3, 18	2, 3
18 C	138.763				17', 17"	
19 C						
H						
20 C						
H						
21 C						
H						
22 C	72.733			22', 22"	2	
H'	4.700	12.60(22")	22"	22	2, 23	2
H"	4.780	12.60(22')	22'	22	2, 23	2
23 C	138.369				22', 22"	
24 C						
H						
25 C						
H						

Atom	δ (ppm)	J	COSY	HSQC	HMBC	NOESY
26 C						
H						
27 C	80.177			27	1, 28, 29, 31, 32	
H	3.317	10.00(31), 7.50(28)	28, 31	27	1, 28, 31, 32	1, 32, 35
28 C	76.895			28	27, 29, 30, 31	
H	3.967	7.50(27), 5.60(29)	27, 29, 30, 35	28	27, 34	31
29 C	75.992			29	30	
H	4.035	5.60(28), 0.70(30)	28, 30	29	27, 28	30, 36
30 C	98.114	168.00(30H)		30	31, 33	
H	4.810	168.00(30C), 0.70(29)	28, 29, 31	30	28, 29, 31, 33	29, 33
31 C	64.832			31	27, 30, 32	
H	3.519	10.00(27), 6.30(32)	27, 30, 32	31	27, 28, 30, 32	28, 32, 33
32 C	17.422			32	27, 31	
H3	1.039	6.30(31)	31	32	27, 31	1, 27, 31
33 C	54.922			33	30	
H3	3.333			33	30	30, 31
34 C	109.159				28, 35, 36	
35 C	28.253			35	36	
H3	1.477		28	35	34, 36	27
36 C	26.448			36	35	
H3	1.260			36	34, 35	29

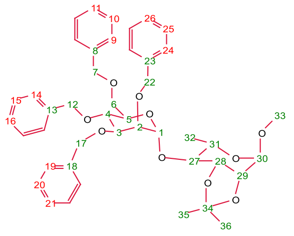

1H{off},1D, 600.23 MHz,CDCl3,298.0K, pulse sequence: zg30

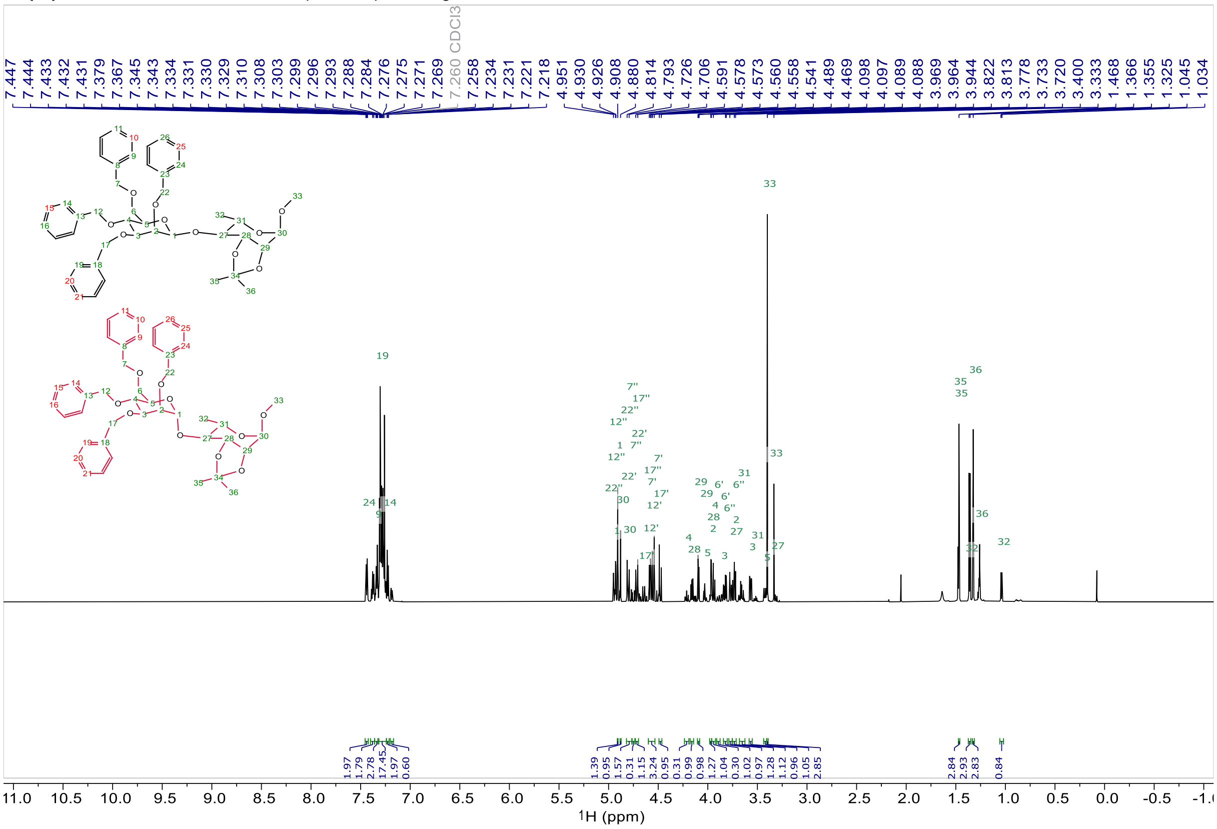

1H{off},1D, 600.23 MHz,CDCl3,298.0K, pulse sequence: zg30

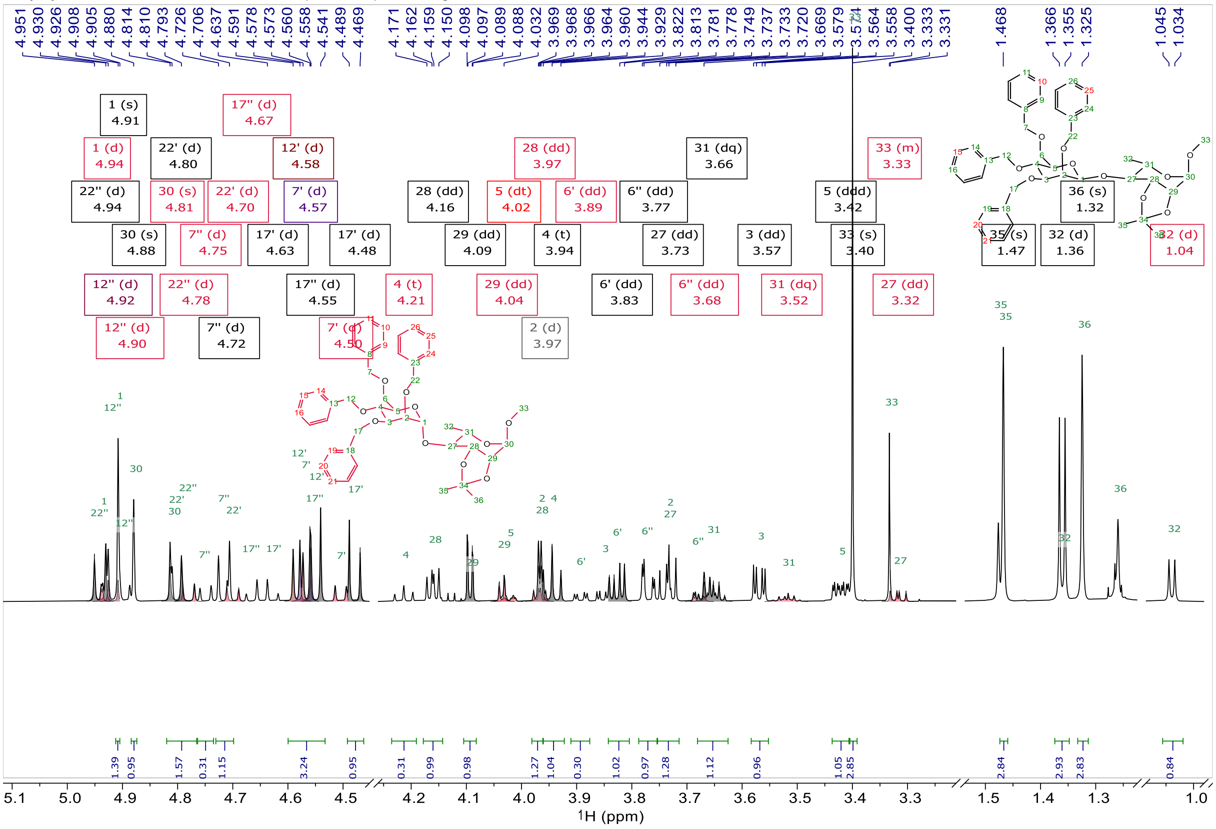

13C{1H},1D, 150.94 MHz,CDCl3,298.0K, pulse sequence: zgpg30

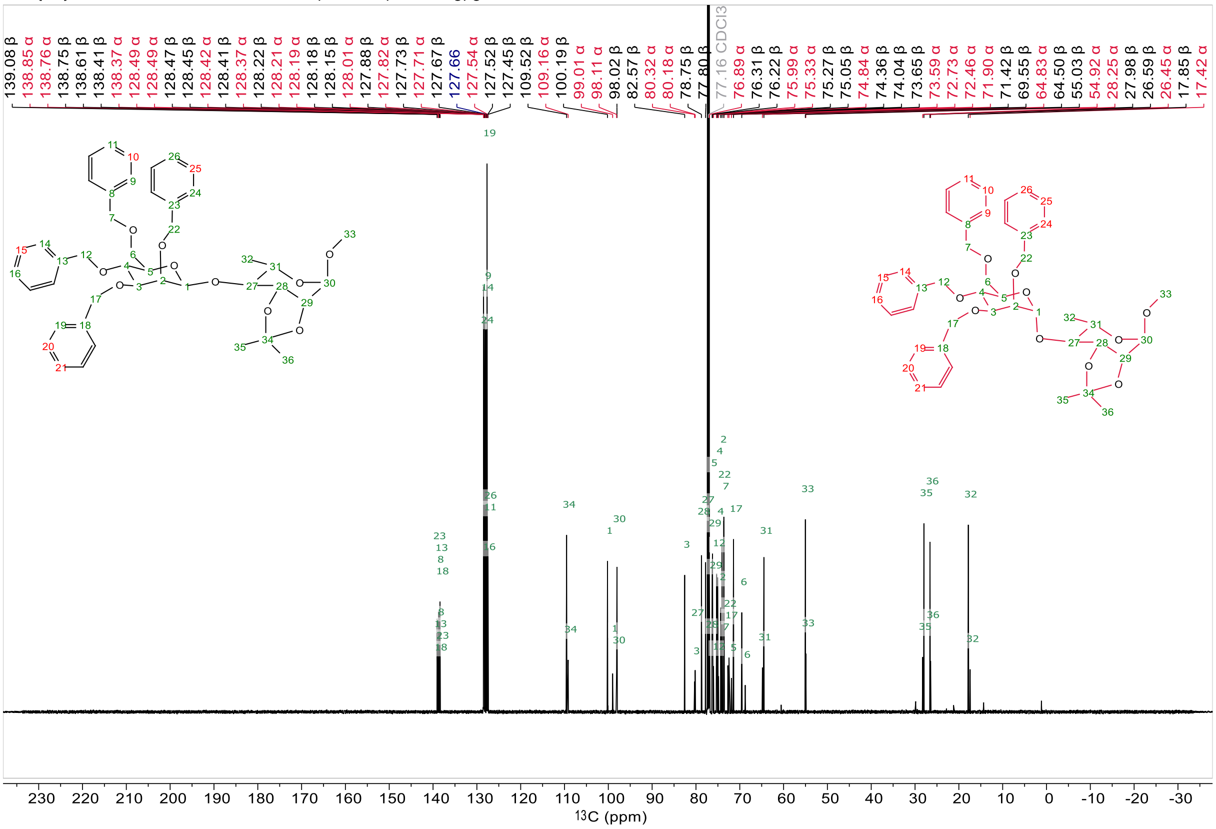

1H{13C},HSQC-EDITED, 600.22 MHz,CDCl3,298.0K, pulse sequence: hsqcedetgpsisp2.3

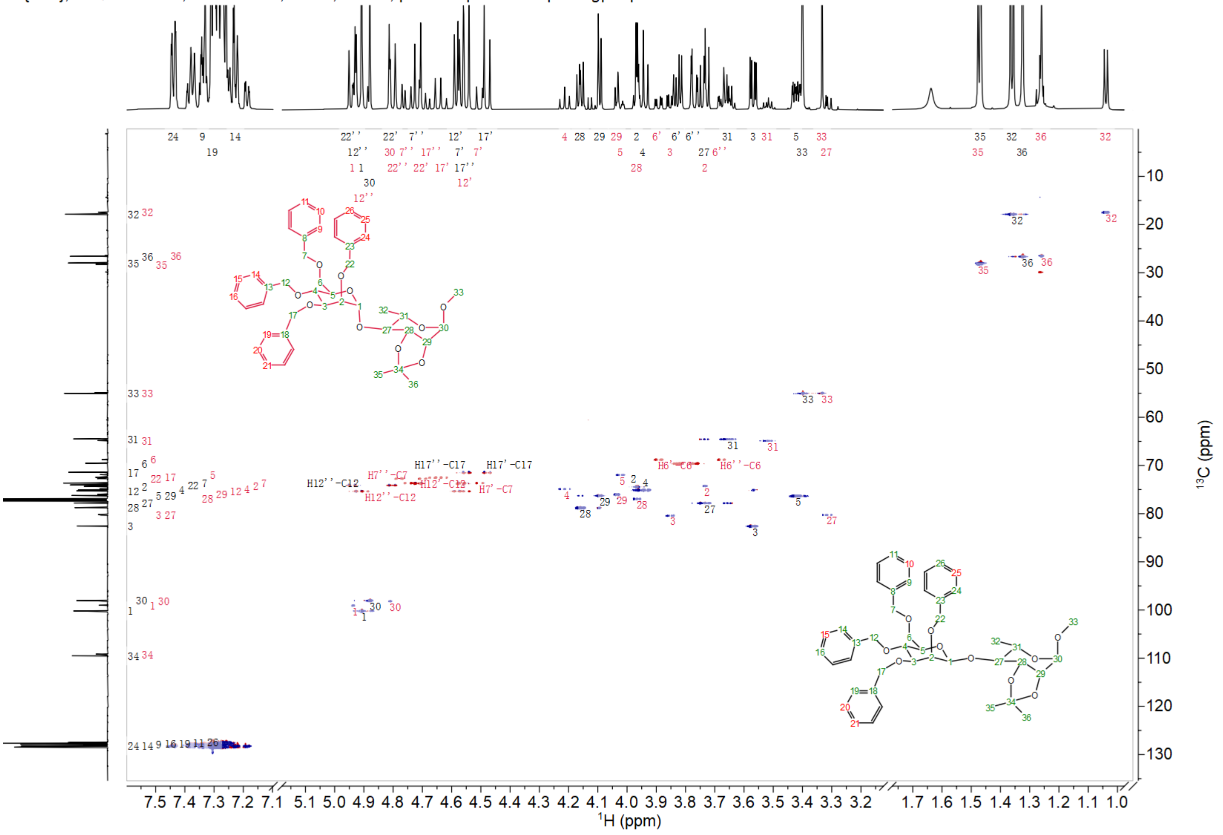

1H{13C},HMBC, 600.22 MHz,CDCl3,298.0K, pulse sequence: hmbcetgpl3nd

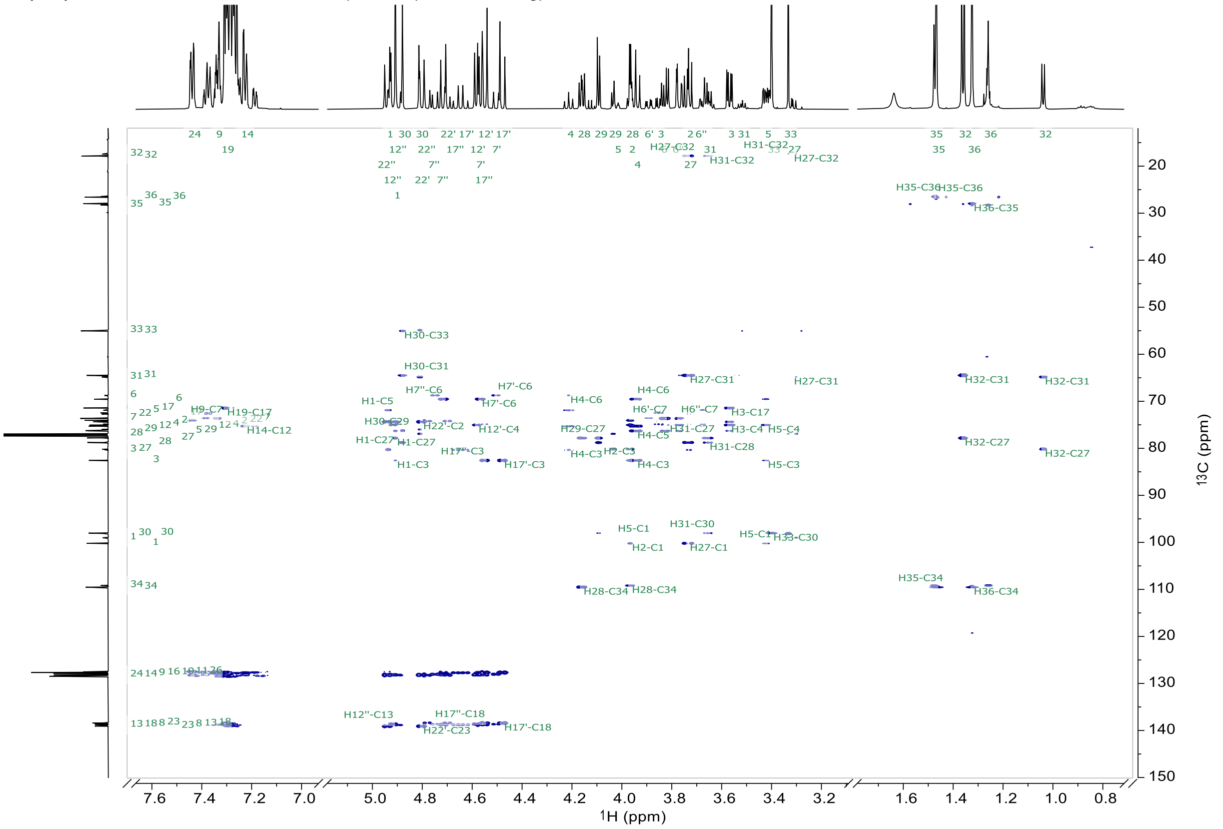

1H{off},COSY, 600.22 MHz,CDCl3,298.0K, pulse sequence: cosygpppqf

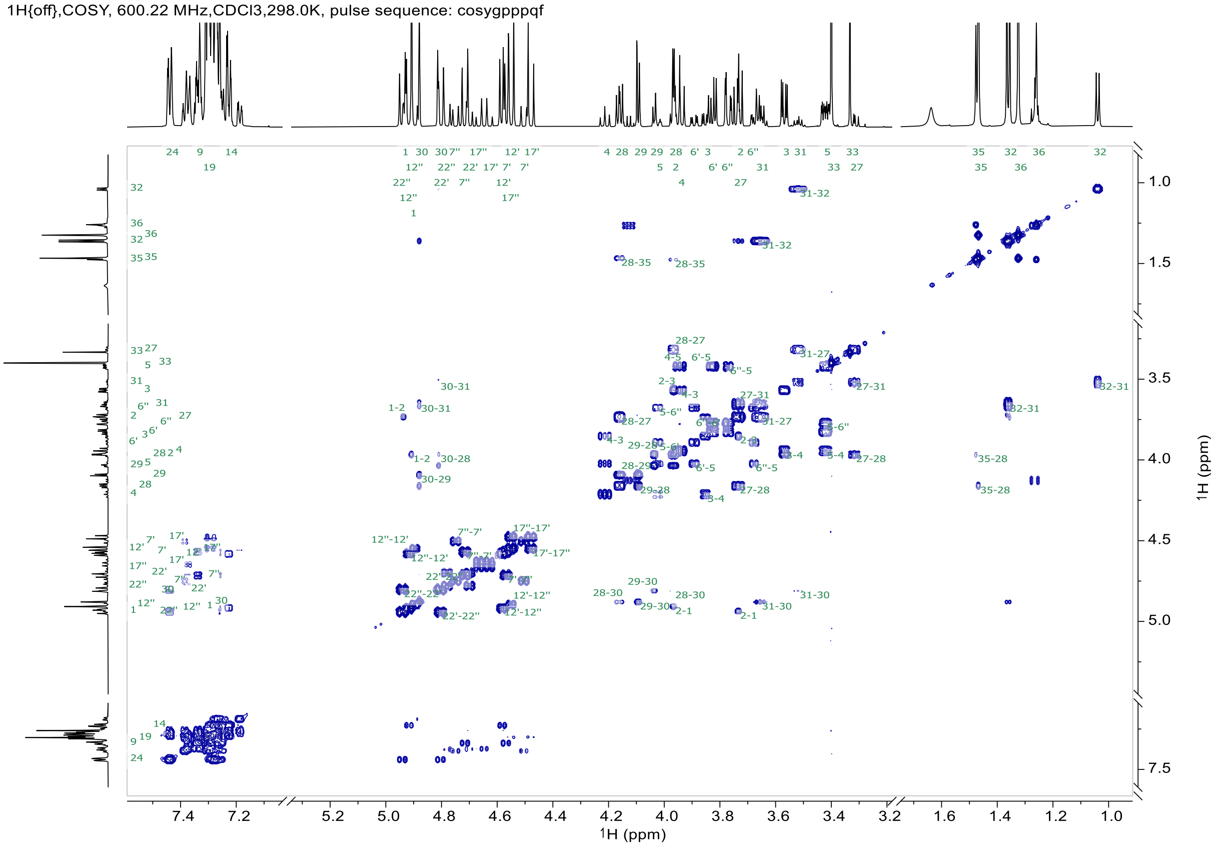

1H{off},NOESY, 600.22 MHz,CDCl3,298.0K, pulse sequence: noesygpph

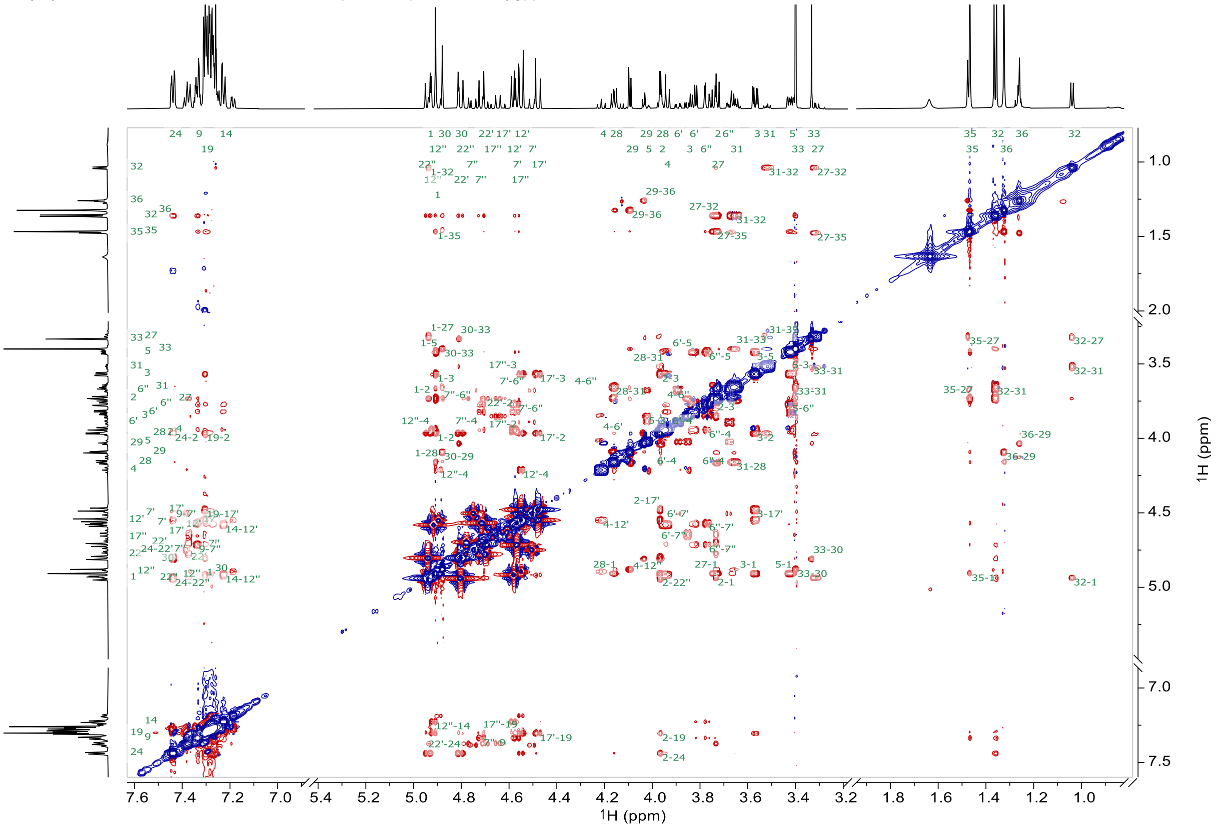

13C{1H},DEPT-135, 150.94 MHz,CDCl3,298.0K, pulse sequence: deptsp135

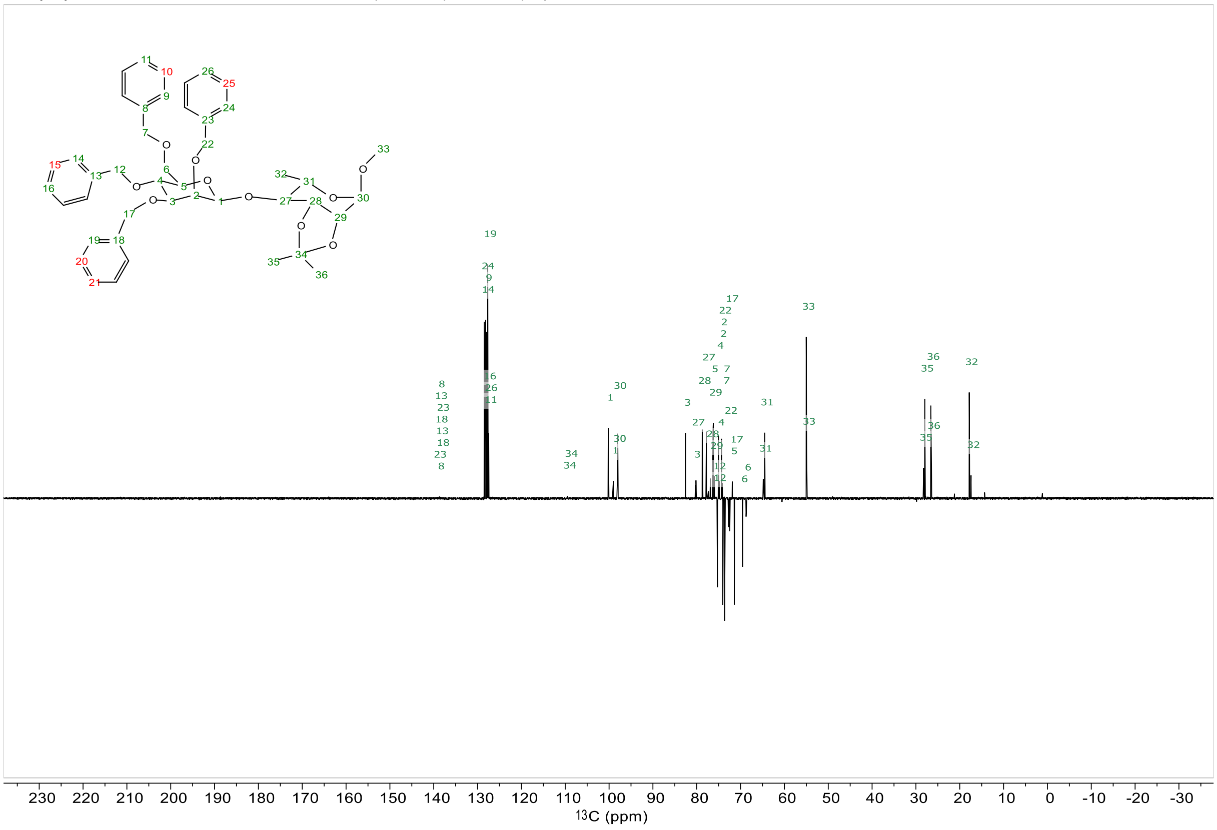

1H{13C},HSQC, 600.22 MHz,CDCl3,298.0K, pulse sequence: hsqcetgpipjcsp.2

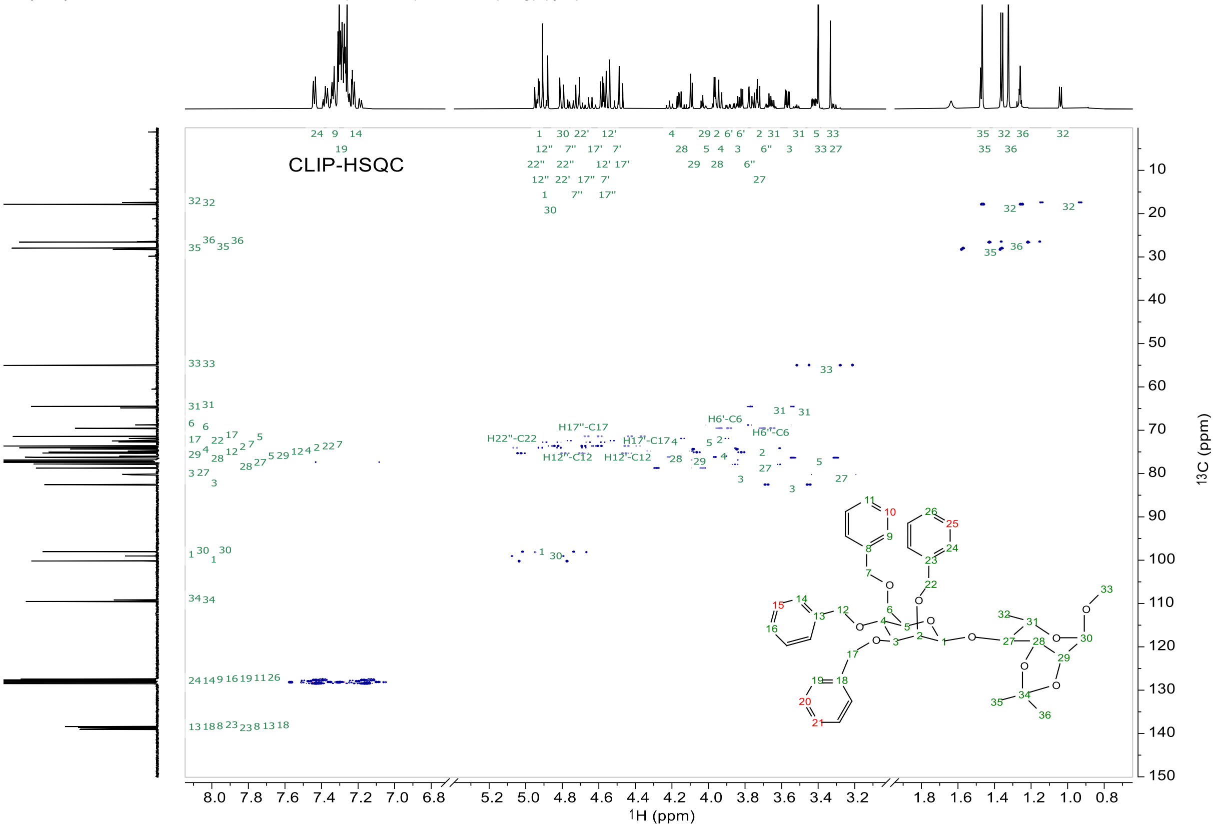

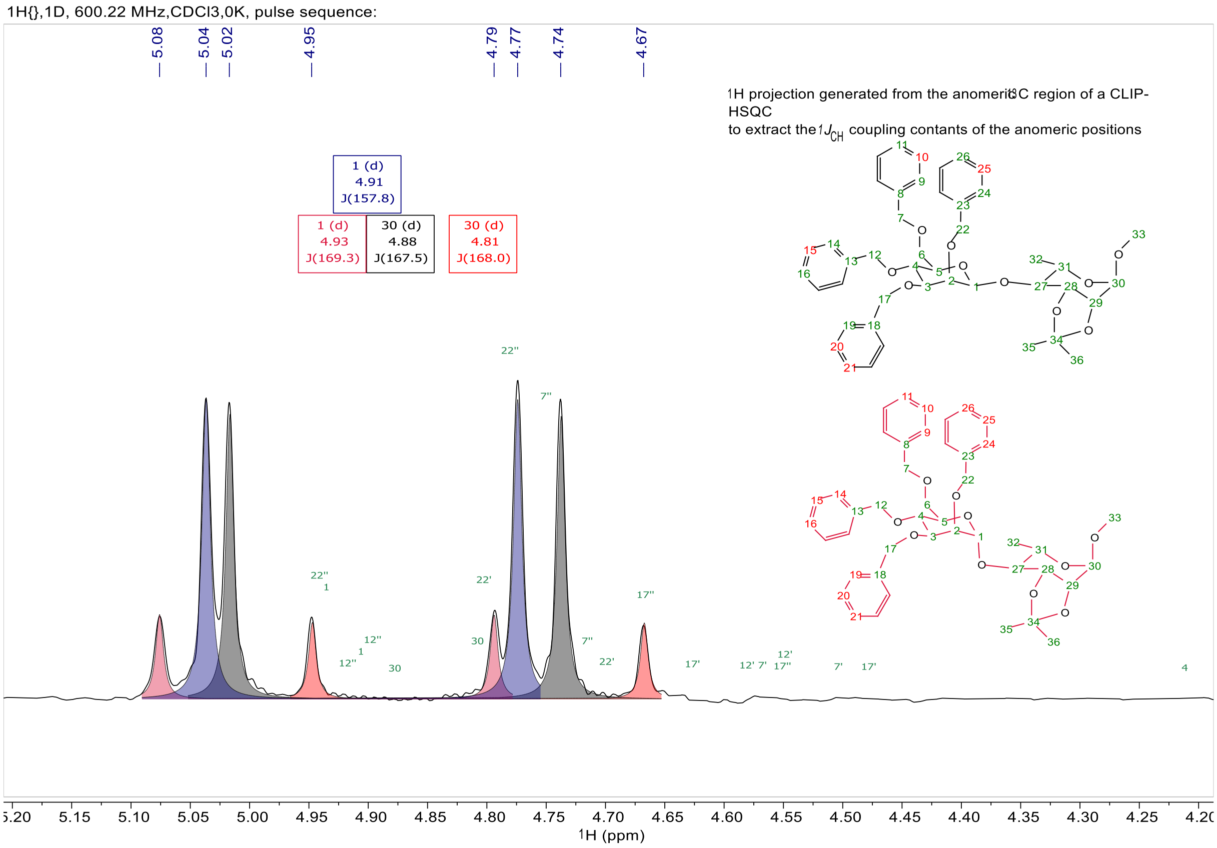
1H{},1D, 600.22 MHz,CDCl3,0K, pulse sequence:
5.08
5.04
5.02
4.95
4.79
4.77
4.74
4.67
1H projection generated from the anomeric 13C region of a CLIP-HSQC
to extract the 1JCH coupling contants of the anomeric positions
1 (d)
4.91
J(157.8)
1 (d)
4.93
J(169.3)
30 (d)
4.88
J(167.5)
30 (d)
4.81
J(168.0)
1H (ppm)

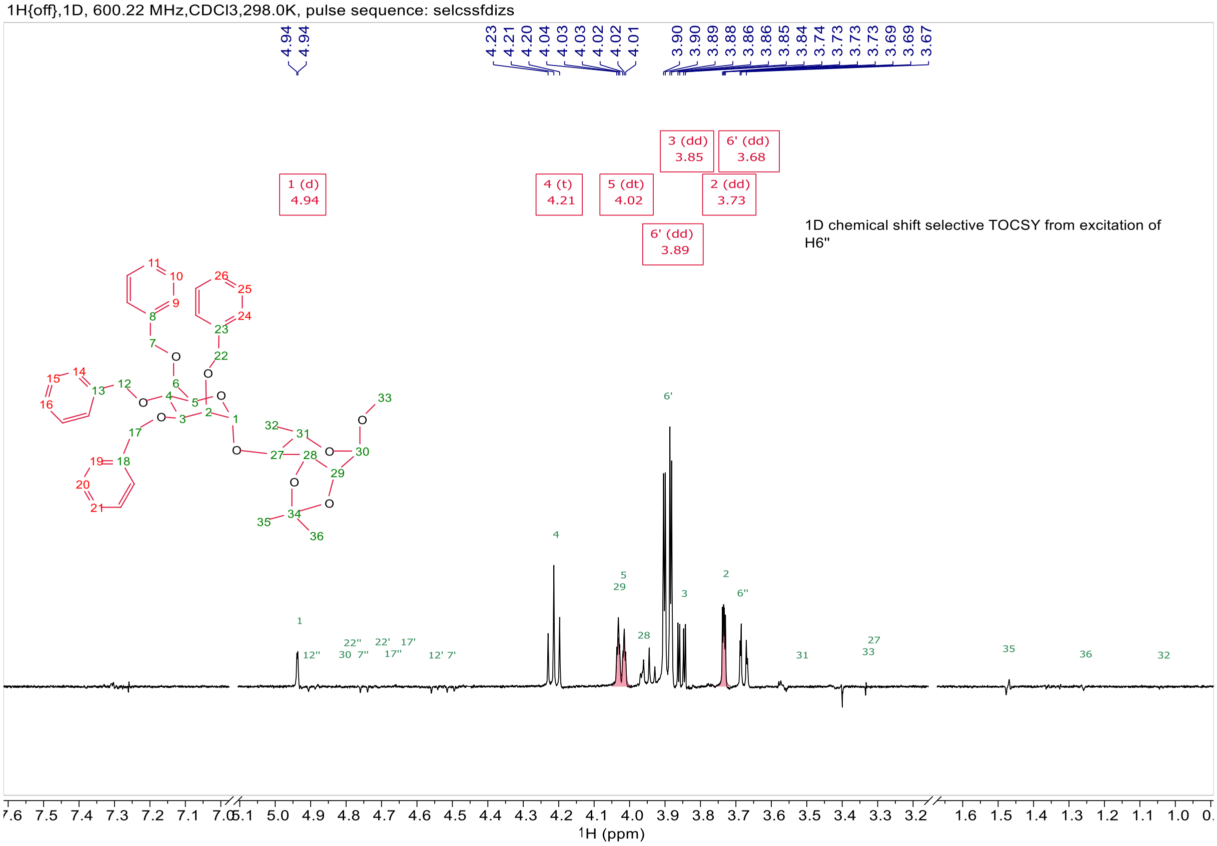
1H{off},1D, 600.22 MHz,CDCl3,298.0K, pulse sequence: selcssfdizs
1 (d)
4.94
4 (t)
4.21
5 (dt)
4.02
3 (dd)
3.85
6' (dd)
3.68
2 (dd)
3.73
6' (dd)
3.89
1D chemical shift selective TOCSY from excitation of H6''
1H (ppm)

1H{off},1D, 600.22 MHz,CDCl3,298.0K, pulse sequence: selcssfdizs

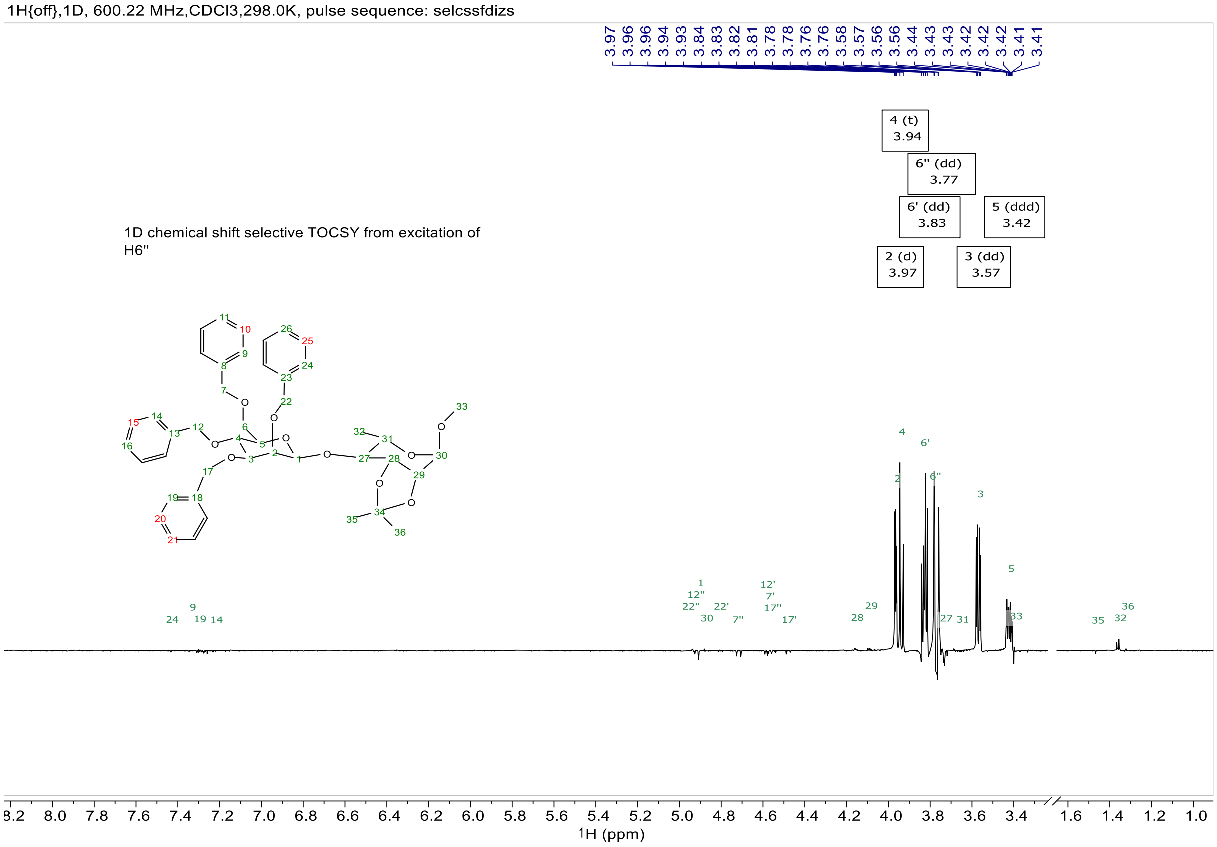

1H{off},1D, 600.22 MHz,CDCl3,298.0K, pulse sequence: selcssfdizs

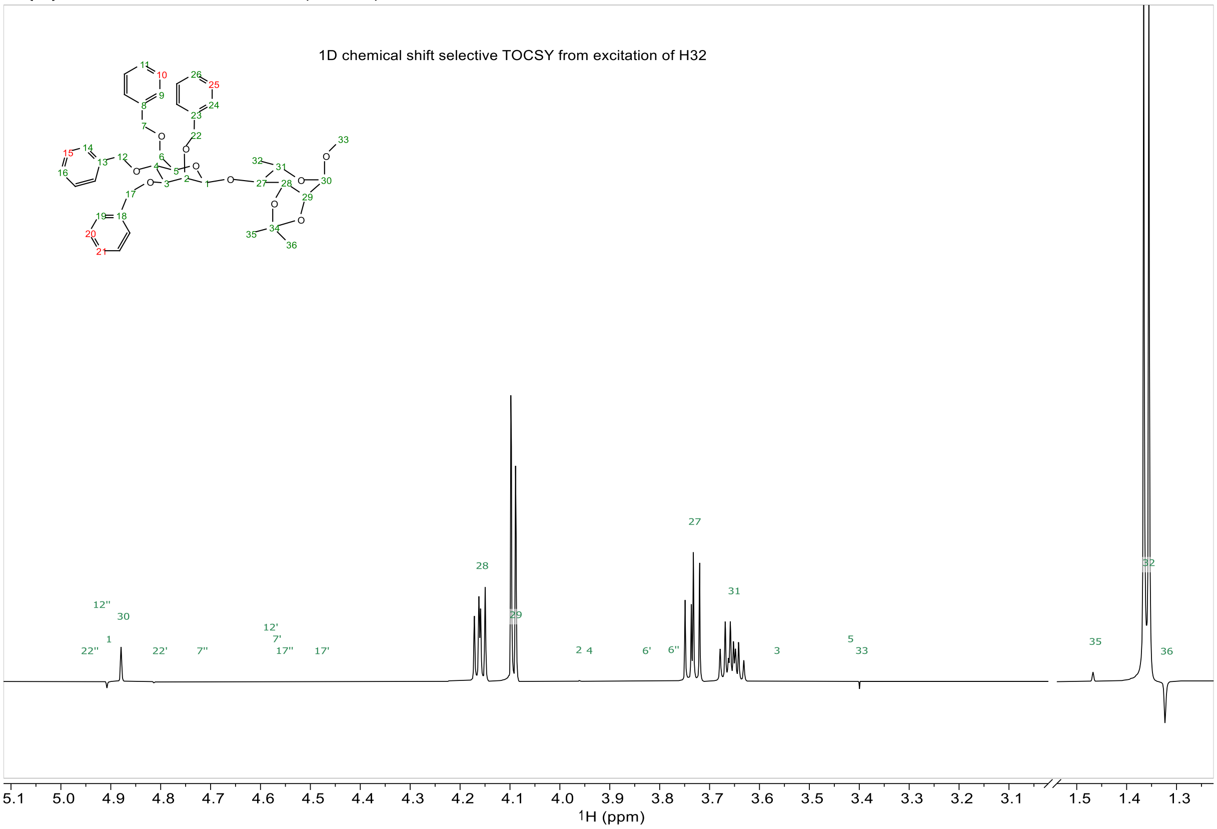

1H{off},1D, 600.22 MHz,CDCl3,298.0K, pulse sequence: selcssfdizs

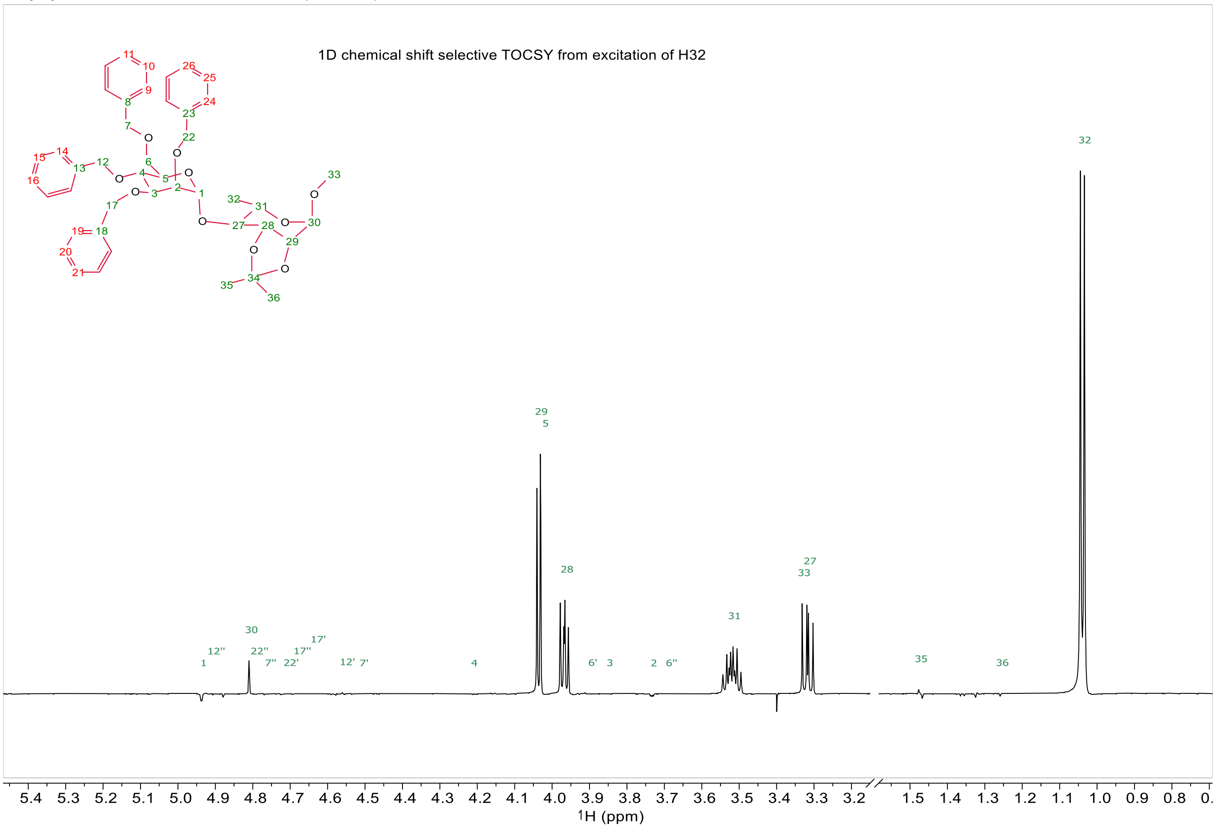

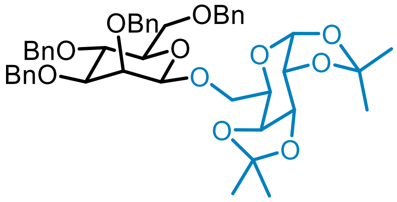

(3a*R*,5*R*,5a*S*,8a*S*,8b*R*)-2,2,7,7-tetramethyl-5-((((2*R*,3*S*,4*S*,5*R*,6*R*)-3,4,5-tris(benzyloxy)-6-((benzyloxy)methyl)tetrahydro-2*H*-pyran-2-yl)oxy)methyl)tetrahydro-5*H*-bis([1,3]dioxolo)[4,5-b:4',5'-d]pyran (6f)

Following **General Procedure B**, glycosyl donor **1c** (34 mg, 0.050 mmol) and alcohol **2f** (35 mg, 0.075 mmol) were coupled using (*S*,*S*)-IDPi **3c** (9.4 mg,10 mol%). Reaction time: 24 h. β/α = 91:9 was determined by crude ^{1}H NMR. Flash chromatography (hexane/EtOAc = 6/1 to 3/1) gave **6f** as off-white foam (30 mg, 77%).

Following **General Procedure A** on 0.01 mmol scale, the glycosylation was carried out using (*R*,*R*)-IDPi **3c**. β/α = 78:22 was determined by crude ^{1}H NMR.

TLC: R_f = 0.31 (Hexane/EtOAc = 3:1)

^{1}H NMR (501 MHz, $CDCl_3$) δ 1.34 (d, *J* = 6.3 Hz, 6H), 1.47 (d, *J* = 16.3 Hz, 6H), 3.39 – 3.51 (m, 2H), 3.63 (dd, *J* = 10.8, 8.3 Hz, 1H), 3.73 – 3.83 (m, 2H), 3.90 (t, *J* = 9.6 Hz, 1H), 4.01 (d, *J* = 3.0 Hz, 1H), 4.12 (dt, *J* = 8.3, 2.1 Hz, 1H), 4.20 – 4.26 (m, 2H), 4.32 – 4.38 (m, 2H), 4.43 – 4.49 (m, 2H), 4.51 (d, *J* = 10.7 Hz, 1H), 4.56 (d, *J* = 12.1 Hz, 1H), 4.60 – 4.69 (m, 2H), 4.88 – 4.96 (m, 2H), 5.02 (d, *J* = 12.4 Hz, 1H), 5.60 (d, *J* = 5.0 Hz, 1H), 7.17 (dd, *J* = 7.3, 2.2 Hz, 2H), 7.22 – 7.34 (m, 14H), 7.35 (d, *J* = 7.0 Hz, 2H), 7.48 – 7.55 (m, 2H).

^{13}C NMR (126 MHz, $CDCl_3$) δ 138.76, 138.56, 138.55, 138.23, 128.84, 128.43, 128.41, 128.29, 128.15, 128.06, 127.75, 127.72, 127.67, 127.60, 127.52, 109.62, 108.88, 102.50, 96.56, 82.01, 75.94, 75.25, 74.91, 73.69, 73.58, 72.73, 71.78, 71.18, 70.92, 70.65, 70.04, 69.66, 68.20, 26.18, 26.12, 25.23, 24.55.

$[\alpha]_D^{22}$ = -56.8 ($CHCl_3$, *c* = 1.0).

HRMS m/z (ESI): calcd. for C46 H54 O11 Na1 ($[M+Na]^+$): 805.355834; found: 805.355330.

LUJ-LA-383-01 20mg $CDCl_3$ 298 K

The following compound was assigned in the sample:

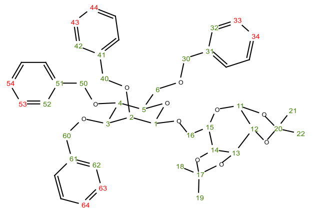

P-ID CW0XXXX
Measured on: 10/01/2026
CHIFFRE: LUJ-LA-383-CO
ELNA#: 16034
Client: Jiaxiang Lu
Group: List
Spectroscopist: Wirtz
Analysed on: 20/02/2026
Analysed by: Wirtz
Amount: 20.0 mg
Solvent: CDCl3
Reference: 1H+13C on solvent,other nuclei w/ xiref
Temperature: 298 K
Spectrometer: AV500lis
Probe: prodigy -BBO
Experiments: 1H-1H-noesygpphpp, 1H-zg30, 13C-zgpg30, 1H-13C-hsqcedetgpsisp2 1H-13C-hmbcetgpl3nd, 1H-1H-cosygpppqf, 1H-1H-noesygpphpp

Remarks:
The relative stereochemistry at the anomeric center is supported by NOE correlations from H1 to the axial positioned protons H3 and H5. The J-coupling of H1 to H2 could not be resolved and is therefore likely <1Hz.
The aryl rings signals were not assigned in detail due to a lack of resolution of the provided dataset.

1H{},NOESY, 500.81 MHz,CDCl3,298.0K, pulse se

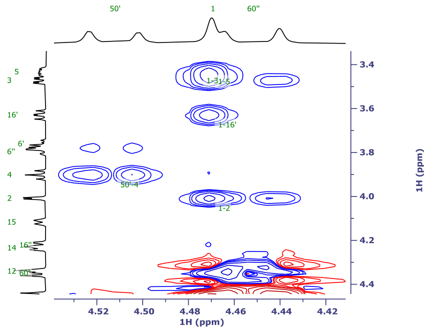

LUJ-LA-383-01 20mg $CDCl_3$ 298 K

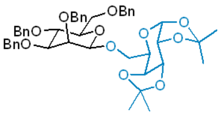

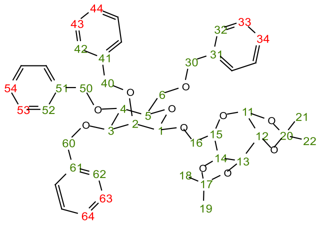

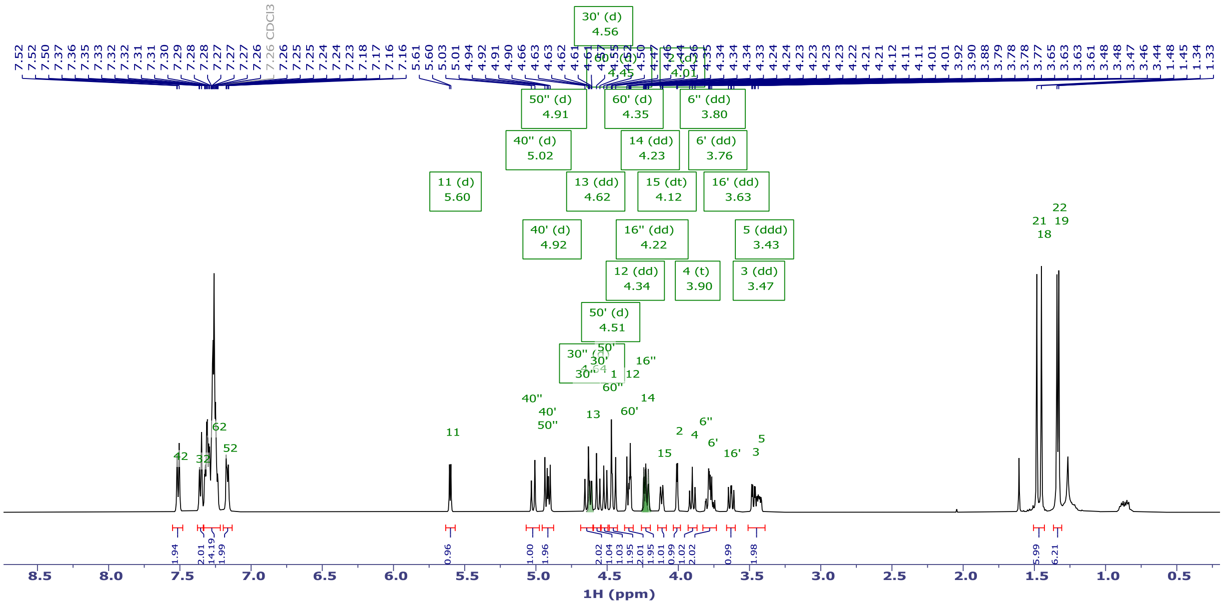

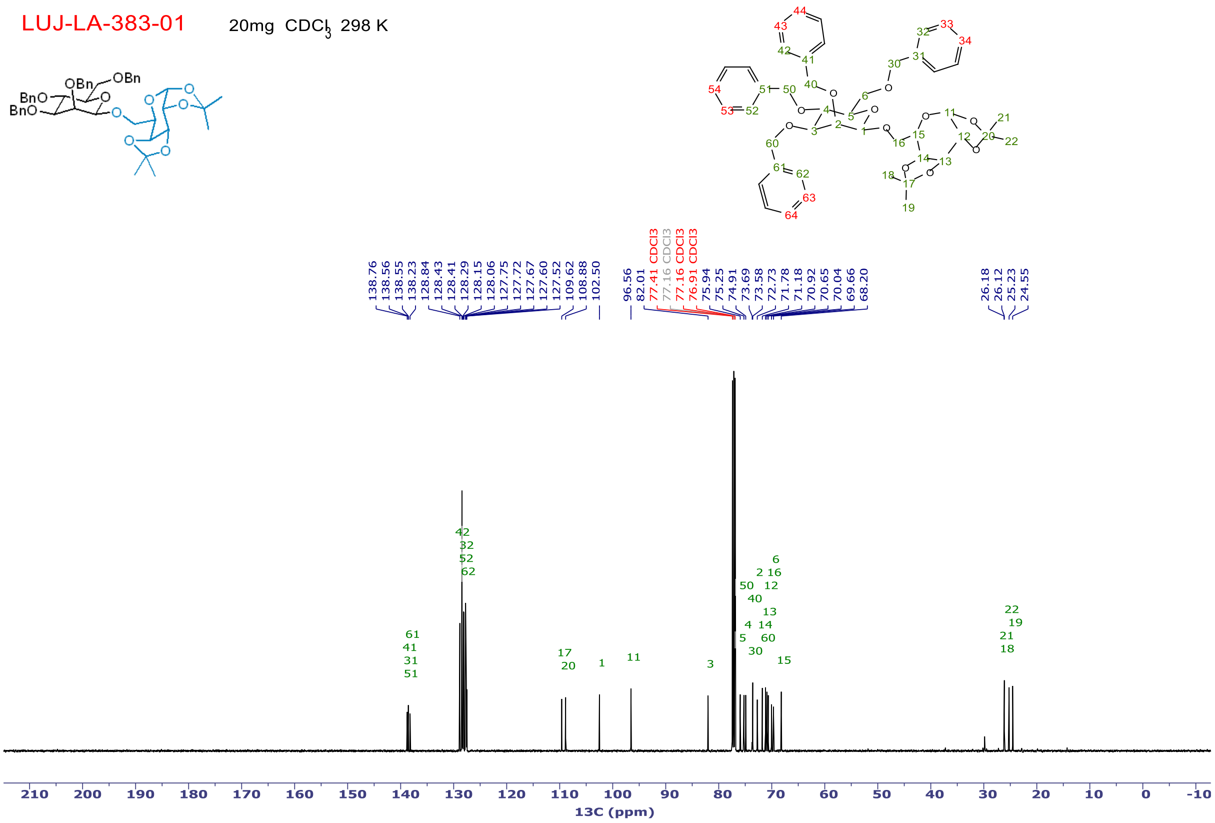
LUJ-LA-383-01
20mg CDCl3 298 K
138.76
138.56
138.55
138.23
128.84
128.43
128.41
128.29
128.15
128.06
127.75
127.72
127.67
127.60
127.52
109.62
108.88
102.50
96.56
82.01
77.41 CDCl3
77.16 CDCl3
77.16 CDCl3
76.91 CDCl3
75.94
75.25
74.91
73.69
73.58
72.73
71.78
71.18
70.92
70.65
70.04
69.66
68.20
26.18
26.12
25.23
24.55
13C (ppm)

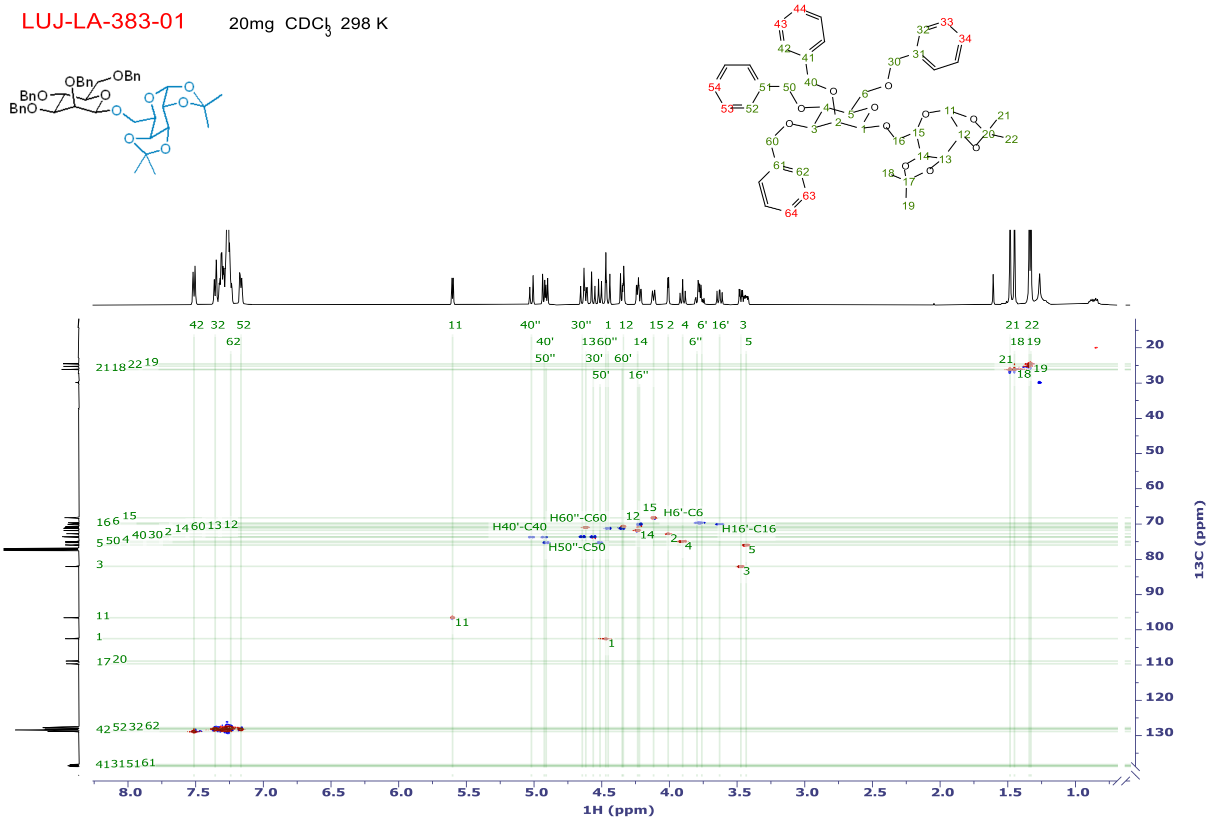
LUJ-LA-383-01
20mg CDCl3 298 K
H40'-C40
H60''-C60
H50''-C50
H6'-C6
H16'-C16
1H (ppm)
13C (ppm)

LUJ-LA-383-01 20mg $CDCl_3$ 298 K

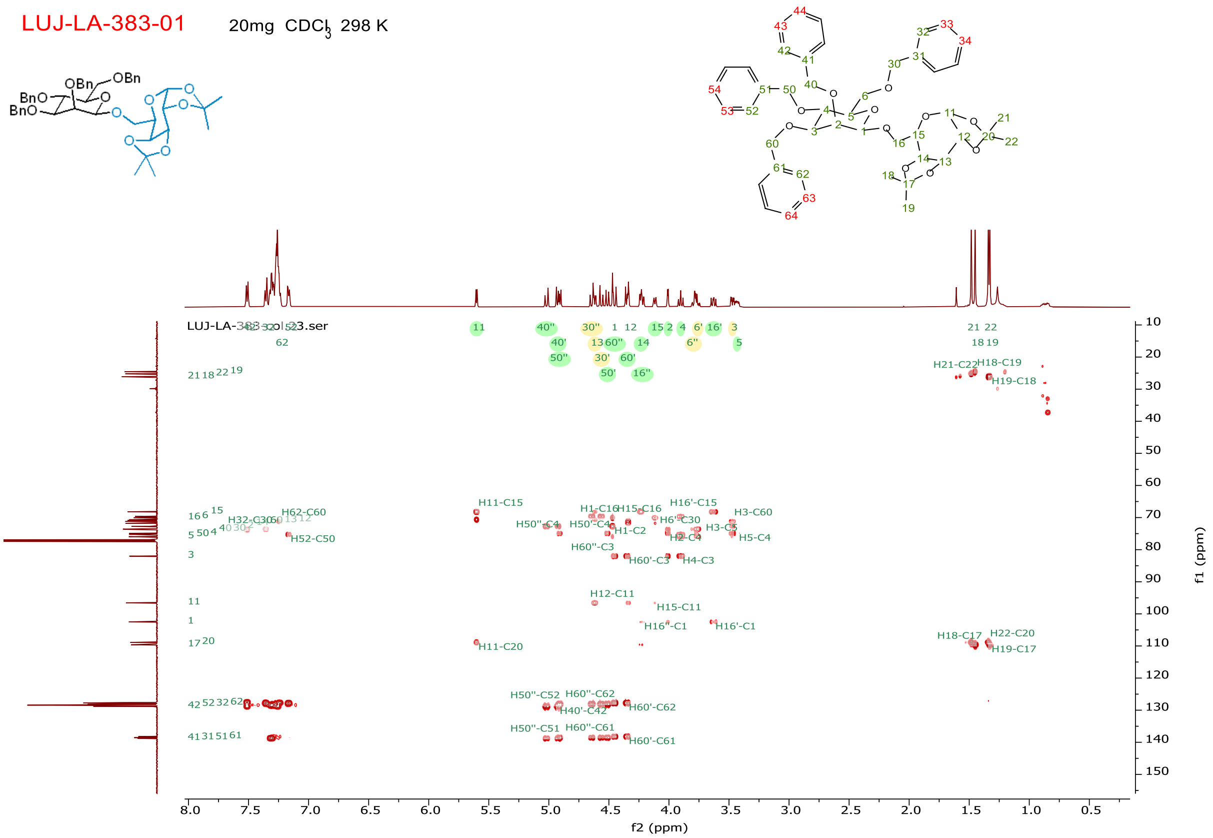

LUJ-LA-383-01 20mg $CDCl_3$ 298 K

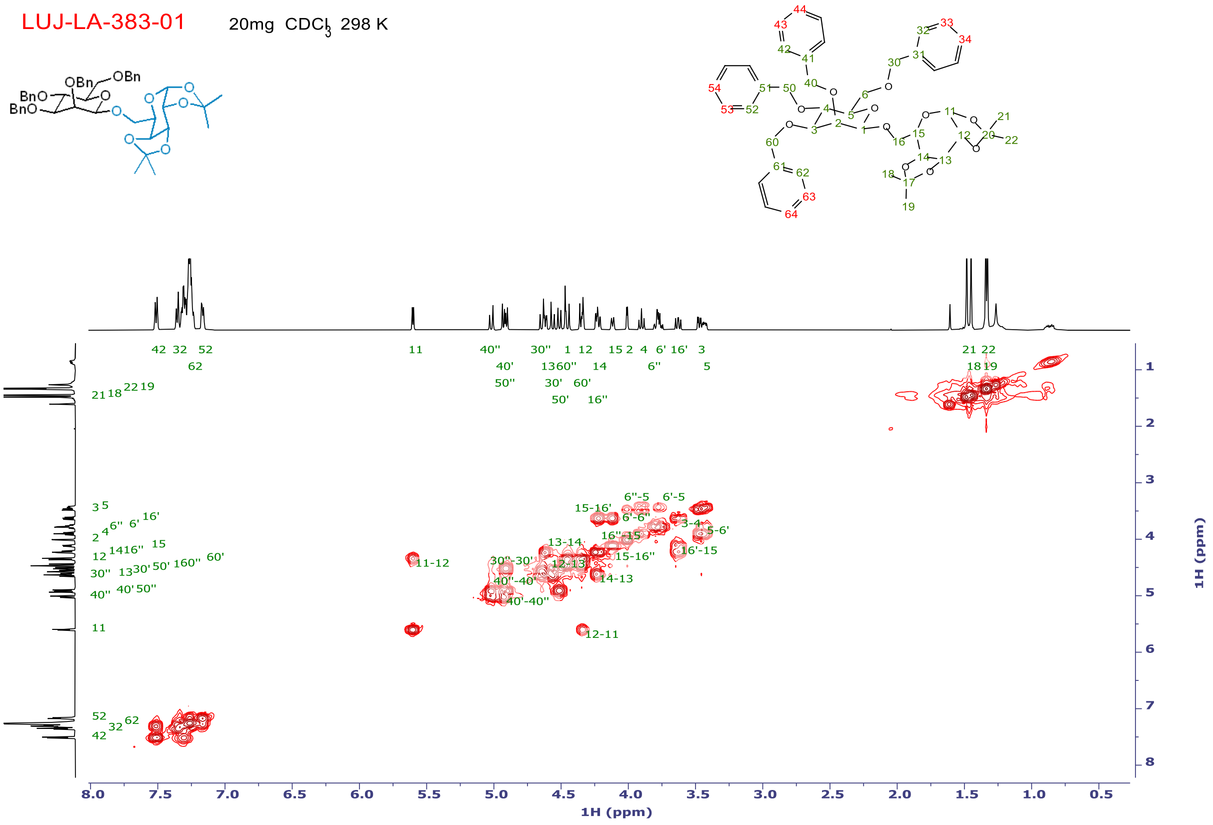

LUJ-LA-383-01 20mg $CDCl_3$ 298 K

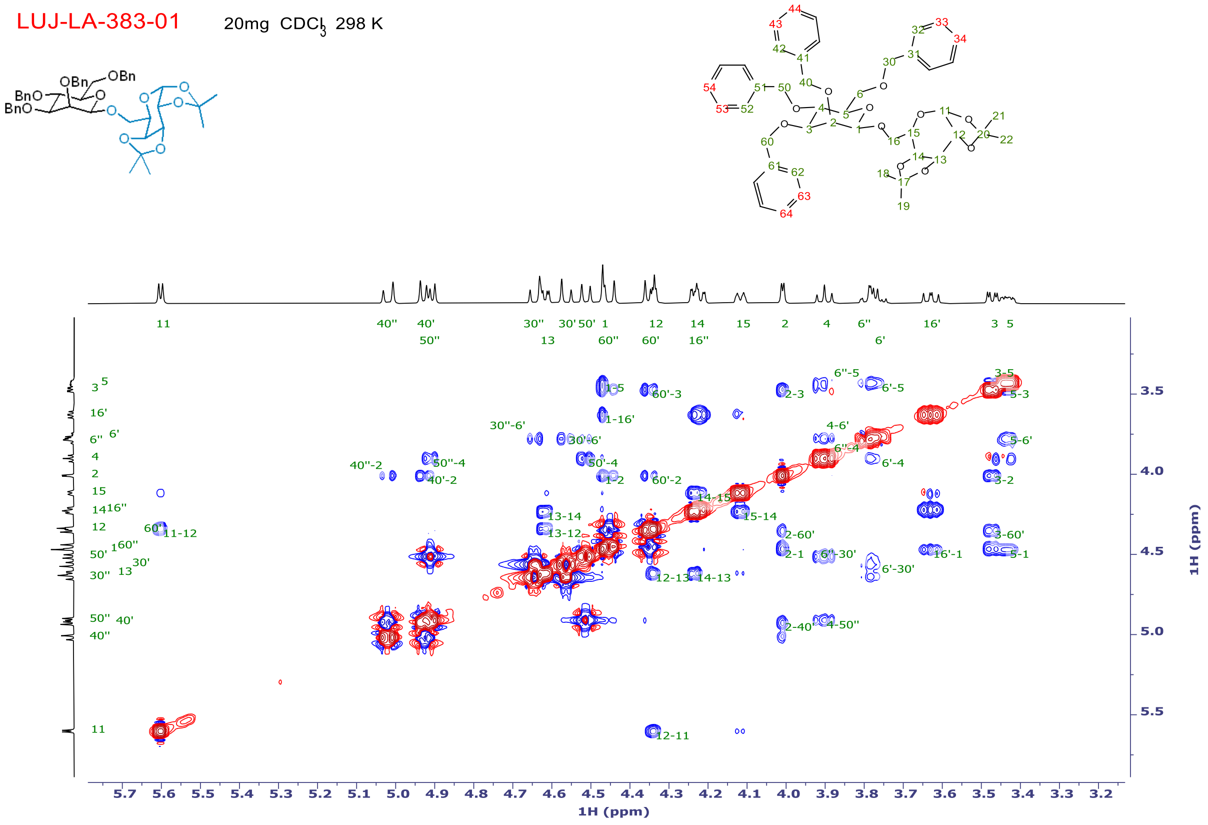

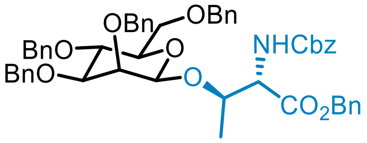

benzyl *N*-((*benzyloxy*)carbonyl)-*O*-((2*R*,3*S*,4*S*,5*R*,6*R*)-3,4,5-tris(benzyloxy)-6-((*benzyloxy*)methyl)tetrahydro-2*H*-pyran-2-yl)-L-threoninate (6g)

Following **General Procedure B**, glycosyl donor **1c** (34 mg, 0.050 mmol) and alcohol **2g** (35 mg, 0.075 mmol) were coupled using (*S*,*S*)-IDPi **3c** (9.4 mg,10 mol%). Reaction time: 72 h. β/α = 88:12 was determined by crude ^{1}H NMR. Flash chromatography (hexane/EtOAc = 6/1 to 3/1) gave **6g** as colorless oil (29 mg, 68%).

Following **General Procedure A** on 0.01 mmol scale, the glycosylation was carried out using (*R*,*R*)-IDPi **3c**. β/α = 77:23 was determined by crude ^{1}H NMR.

TLC: R_f = 0.32 (Hexane/EtOAc = 3:1)

^{1}H NMR (501 MHz, $CDCl_3$) δ 1.22 (d, *J* = 6.4 Hz, 3H), 3.31 (ddd, *J* = 9.7, 4.2, 2.6 Hz, 1H), 3.47 (dd, *J* = 9.4, 3.0 Hz, 1H), 3.66 – 3.75 (m, 2H), 3.83 (d, *J* = 2.9 Hz, 1H), 3.95 (t, *J* = 9.5 Hz, 1H), 4.37 – 4.46 (m, 3H), 4.48 – 4.63 (m, 5H), 4.74 (d, *J* = 12.4 Hz, 1H), 4.85 (d, *J* = 12.4 Hz, 1H), 4.89 (d, *J* = 10.8 Hz, 1H), 5.12 (d, *J* = 2.1 Hz, 4H), 5.77 (d, *J* = 8.7 Hz, 1H), 7.17 – 7.36 (m, 29H), 7.37 – 7.44 (m, 2H).

^{13}C NMR (126 MHz, $CDCl_3$) δ 170.42, 156.92, 138.93, 138.57, 138.54, 138.28, 136.46, 135.61, 128.64, 128.53, 128.45, 128.39, 128.30, 128.26, 128.25, 128.21, 128.14, 128.12, 127.84, 127.78, 127.76, 127.72, 127.52, 127.47, 98.73, 82.34, 76.11, 75.27, 74.66, 74.34, 73.92, 73.63, 73.45, 71.68, 69.46, 67.32, 67.22, 17.01.

$[\alpha]_D^{22}$ = -25.0 ($CHCl_3$, *c* = 1.0).

HRMS m/z (ESI): calcd. for C53 H55 N1 O10 Na1 ($[M+Na]^+$): 888.371817; found: 888.370860.

User Report
LUJ-LA-384-01

The following compound was assigned in the sample

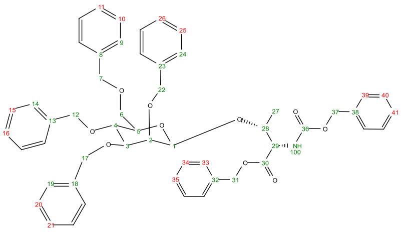

Remarks:
The relative stereochemistry at the anomeric center is supported by NOE correlations from H1 to the axial positioned protons H3 and H5. The J coupling of H1 to H2 could not be resolved and is therefore likely <1Hz.

1H{},NOESY, 500.81 MHz,CDCl3,298.0K, pulse sequence: noesygpphpp

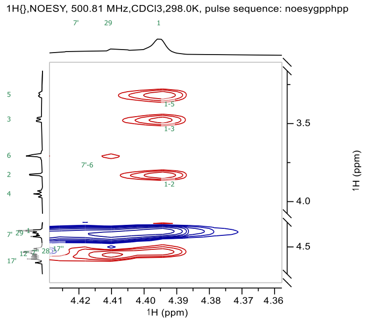

P-ID: ML00000
Measured on: 10/01/2026
CHIFFRE: LUJ-LA-384-01
ELNA#: 16033
Client: Jiaxiang Lu
Group: List
Spectroscopist: Leutzsch
Analysed on: 20/02/2026
Analysed by: Leutzsch
Amount: 20.0 mg
Solvent: CDCl3
Reference: 1H+13C on solvent,other nuclei w/ xiref
Temperature: 298 K
Spectrometer: AV500lis
Probe: prodigy-BBO
Experiments: 1H-1H-noesygpphpp, 1H-zg30, 13C-zgpg30, 1H-1H-cosygpppqf, 1H-13C-hsqcedetgpsisp2.3, 1H-13C-hmbcetgpl3nd, 1H-1H-noesygpphpp

Atom	δ (ppm)	J	COSY	HSQC	HMBC	NOESY
1 C	98.728			1	2, 5, 28	
H	4.396			1	2, 3, 5, 28	2, 3, 5
2 C	74.341			2	1, 3, 22', 22''	
H	3.828	2.90(3)	3	2	1, 3, 4, 22	1, 3, 17', 17'', 22', 22''
3 C	82.336			3	1, 2, 4, 5, 17', 17''	
H	3.473	9.40(4), 2.90(2)	2, 4	3	2, 4, 17	1, 2, 5, 17', 17''
4 C	74.664			4	2, 3, 5, 6', 6'', 12', 12''	
H	3.951	9.40(3), 9.70(5)	3, 5	4	3, 5, 6, 12	6', 6'', 12', 12''
5 C	76.114			5	1, 4, 6', 6''	
H	3.314	9.70(4), 4.20(6'), 2.60(6'')	4, 6', 6''	5	1, 3, 4	1, 3, 6', 6''
6 C	69.458			6', 6''	4, 7', 7''	
H'	3.705	4.20(5)	5, 6''	6	4, 5, 7	4, 5, 7', 7''
H''	3.705	2.60(5)	5, 6'	6	4, 5, 7	4, 5, 7', 7''
7 C	73.627			7', 7''	6', 6'', 9	
H'	4.421	12.00(7'')	7''	7	6, 8	6', 6''
H''	4.537	12.00(7')	7'	7	6, 8	6', 6''
8 C	138.575				7', 7''	
9 C						
H	7.241				7	
10 C						
H						
11 C						
H						
12 C	75.255			12', 12''	4, 14	
H'	4.544	10.80(19'')	12''	12	4, 13	4
H''	4.894	10.80(19')	12'	12	4, 13	4
13 C	138.535				12', 12''	
14 C						
H	7.201				12	

Atom	δ (ppm)	J	COSY	HSQC	HMBC	NOESY
15 C						
H						
16 C						
H						
17 C	71.682			17', 17''	3, 19	
H'	4.590	11.90(17'')	17''	17	3, 18	2, 3, 19
H''	4.512	11.90(17')	17'	17	3, 18	2, 3, 19
18 C	138.283				17', 17''	
19 C						
H	7.314				17	17', 17''
20 C						
H						
21 C						
H						
22 C	73.922			22', 22''	2, 24	
H'	4.735	12.40(22'')	22''	22	2, 23	2
H''	4.848	12.40(22')	22'	22	2, 23	2
23 C	138.930				22', 22''	
24 C						
H	7.406				22	
25 C						
H						
26 C						
H						
27 C	17.012			27	28, 29	
H3	1.221	6.40(28)	28	27	28, 29	28, 29, 100
28 C	73.454			28	1, 27'', 27''', 29	
H	4.527	6.40(27), 3.20(29)	27, 29	28	1	27, 100

Atom	δ (ppm)	J	COSY	HSQC	HMBC	NOESY
29 C	59.075			29	27	
H	4.411	3.20(28), 8.70(100)	28, 100	29	27, 28, 30, 36	27, 100
30 C	170.421				29, 31', 31''	
31 C	67.319			31', 31''		
H'	5.122		31''	31	30, 32	
H''	5.122		31'	31	30, 32	
32 C	135.608				31', 31''	
33 C						
H						
34 C						
H						
35 C						
H						
36 C	156.921				29, 37	
37 C	67.217			37		
H2	5.122			37	36, 38	
38 C	136.462				37	
39 C						
H						
40 C						
H						
41 C						
H						
100 N						
H	5.769	8.70(29)	29			27, 28, 29

1H{},1D, 500.81 MHz,CDCl3,298.0K, pulse sequence: zg30

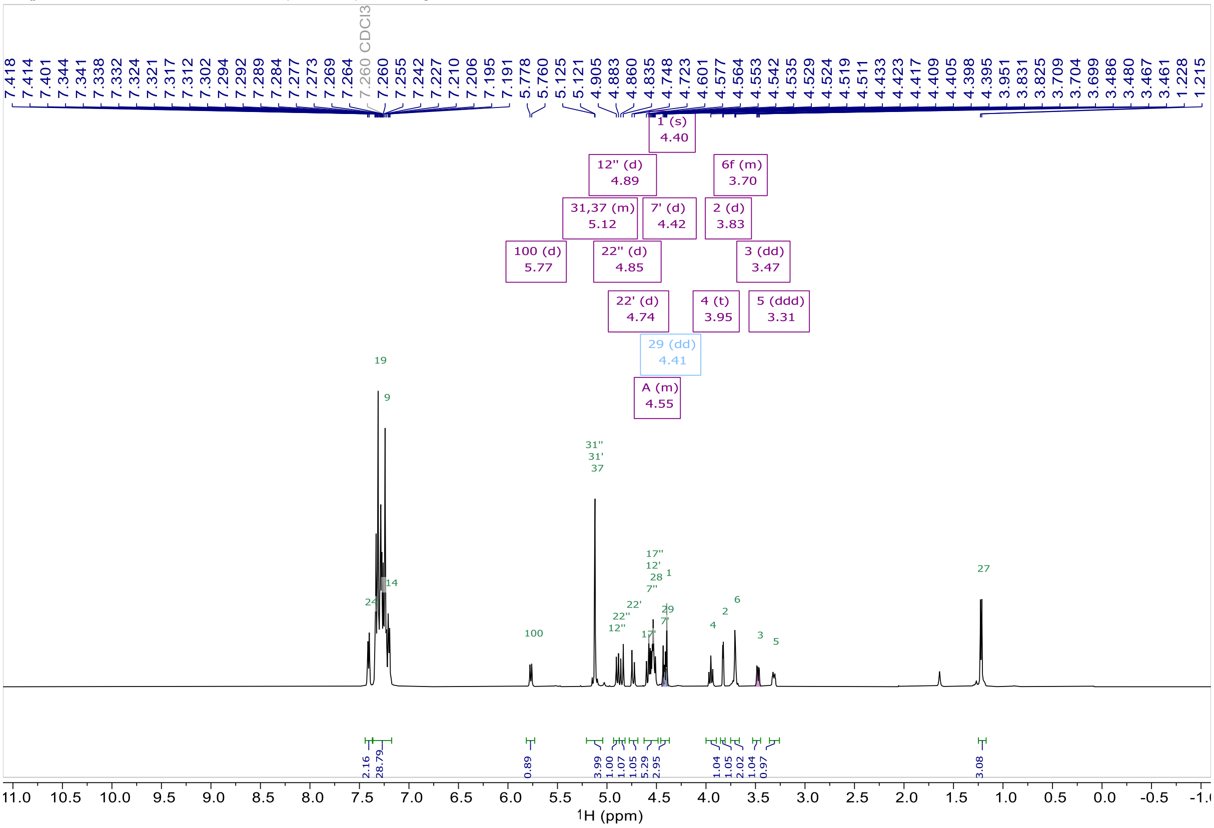

13C{},1D, 125.94 MHz,CDCl3,298.0K, pulse sequence: zgpg30

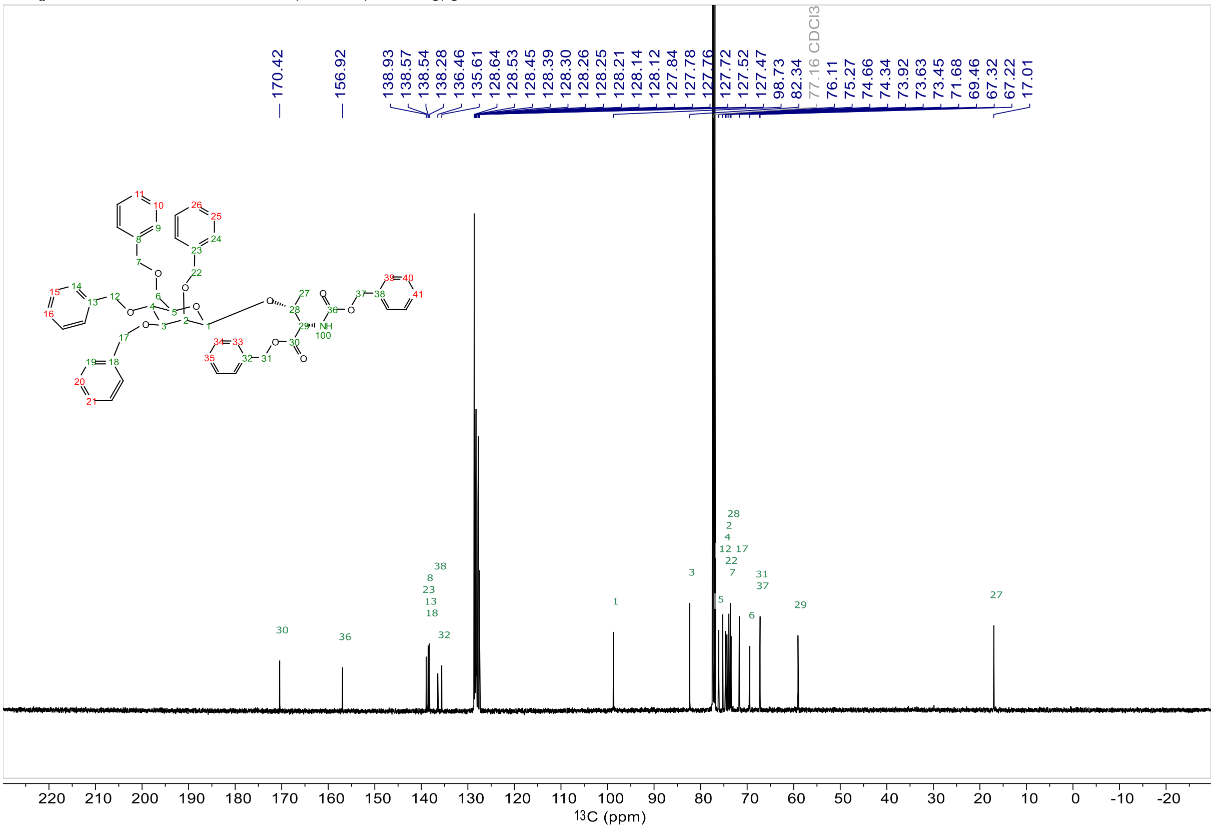

1H{},COSY, 500.81 MHz,CDCl3,298.0K, pulse sequence: cosygpppqf

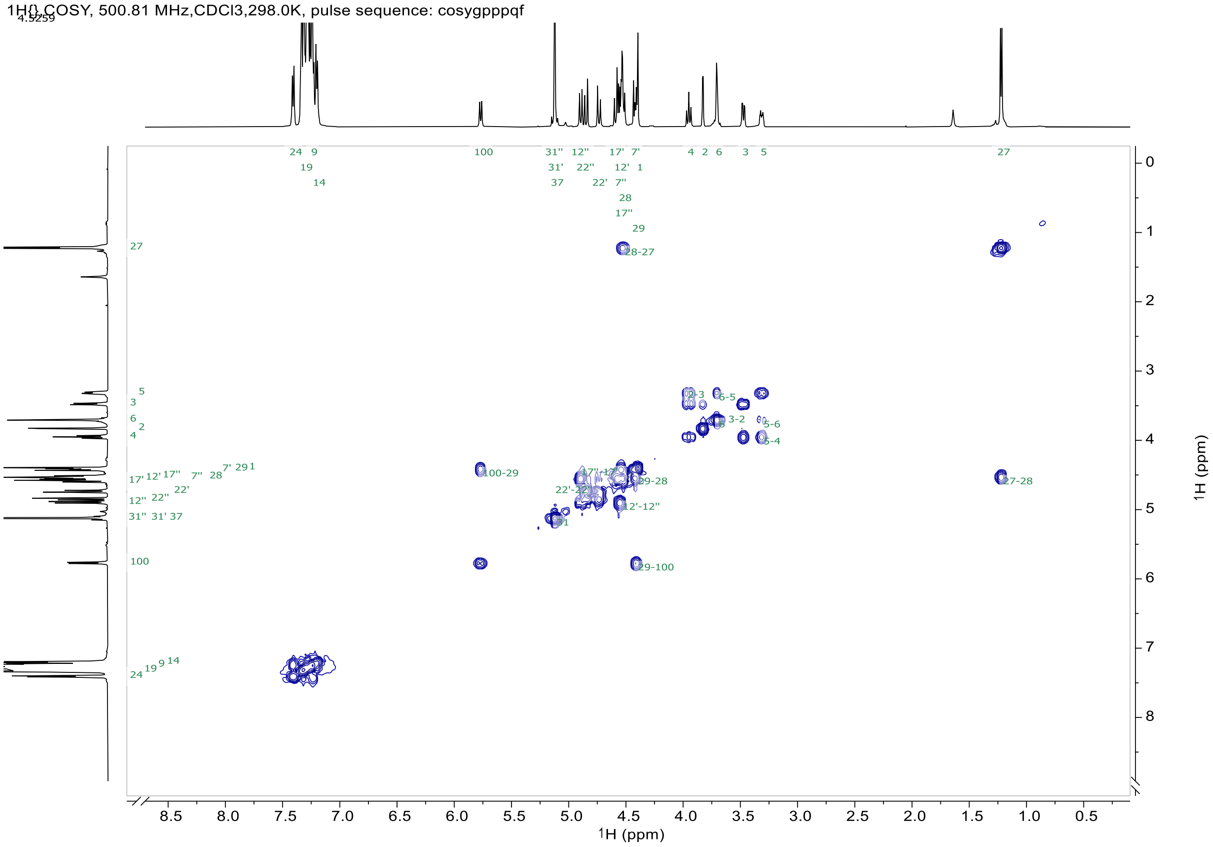

1H{},HSQC-EDITED, 500.81 MHz,CDCl3,298.0K, pulse sequence: hsqcedetgpsisp2.3

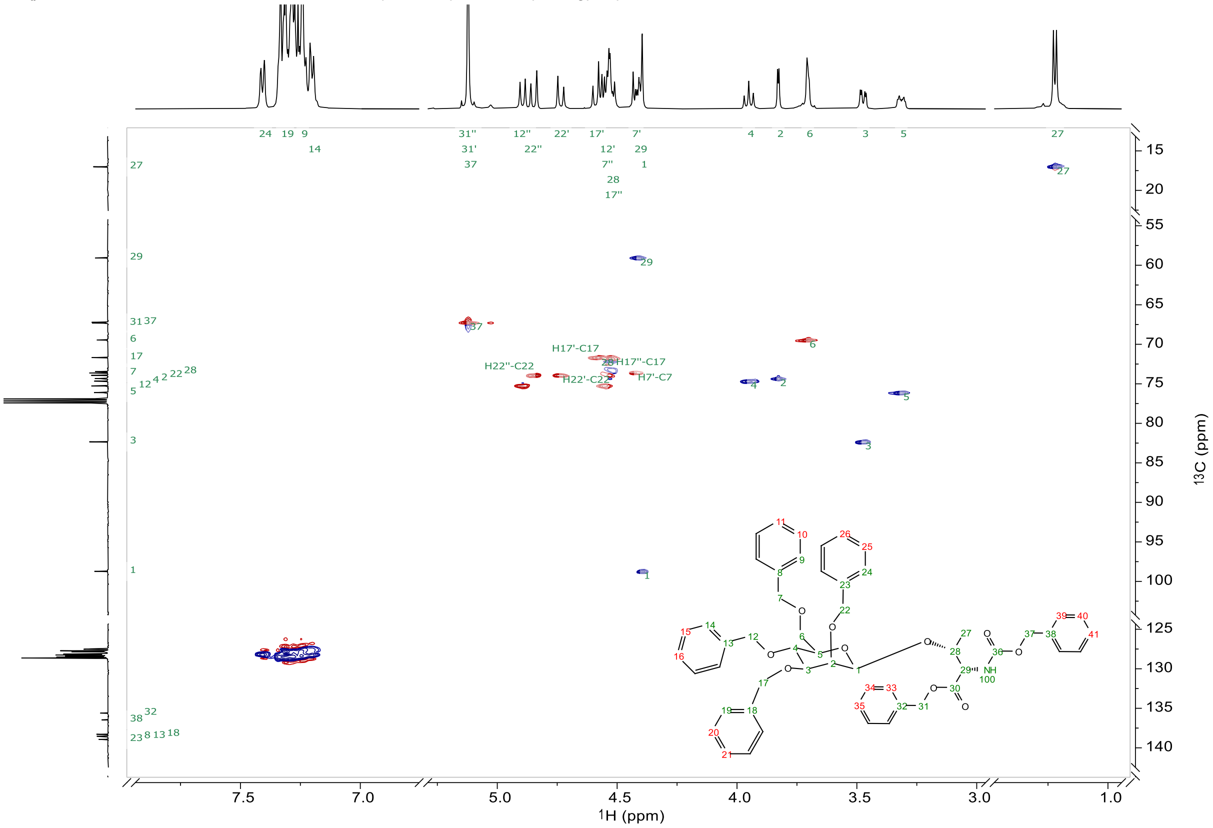

1H{},HMBC, 500.81 MHz,CDCl3,298.0K, pulse sequence: hmbcetgpl3nd

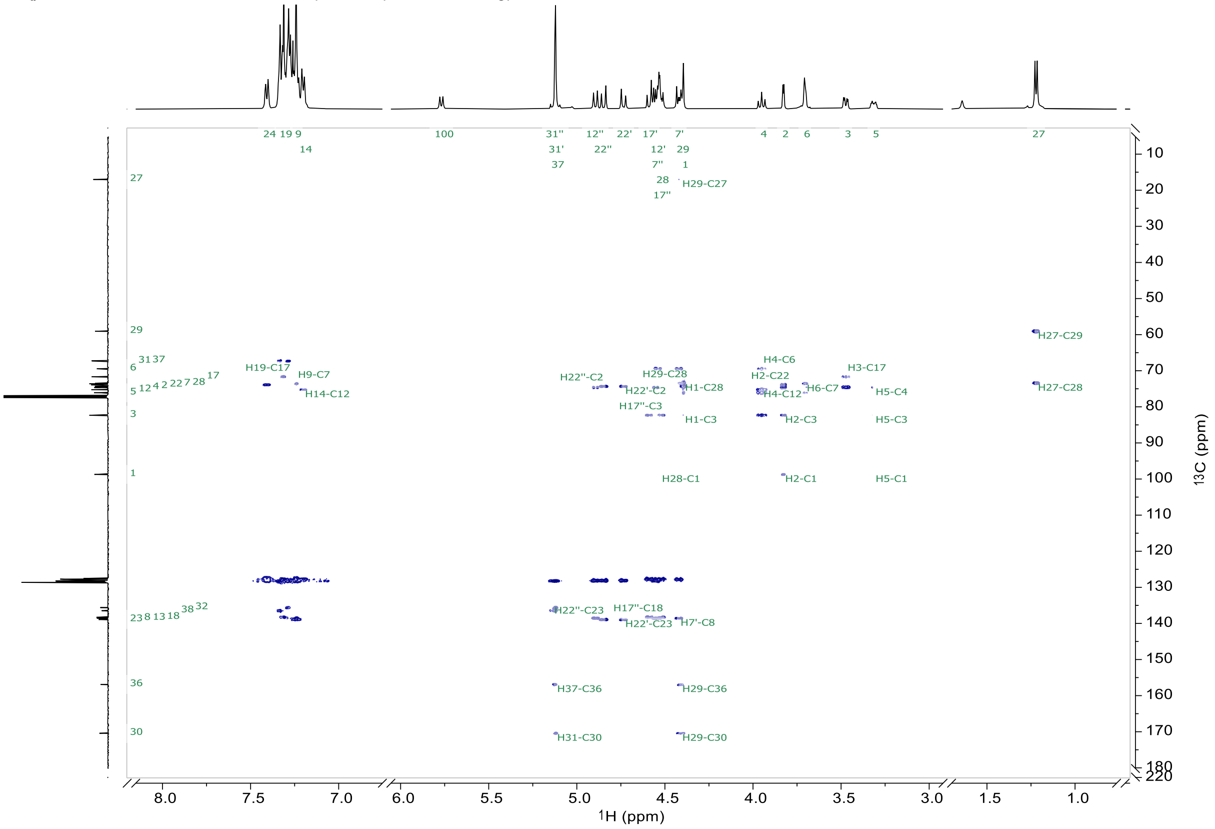

1H{},NOESY, 500.81 MHz,CDCl3,298.0K, pulse sequence: noesygpphpp

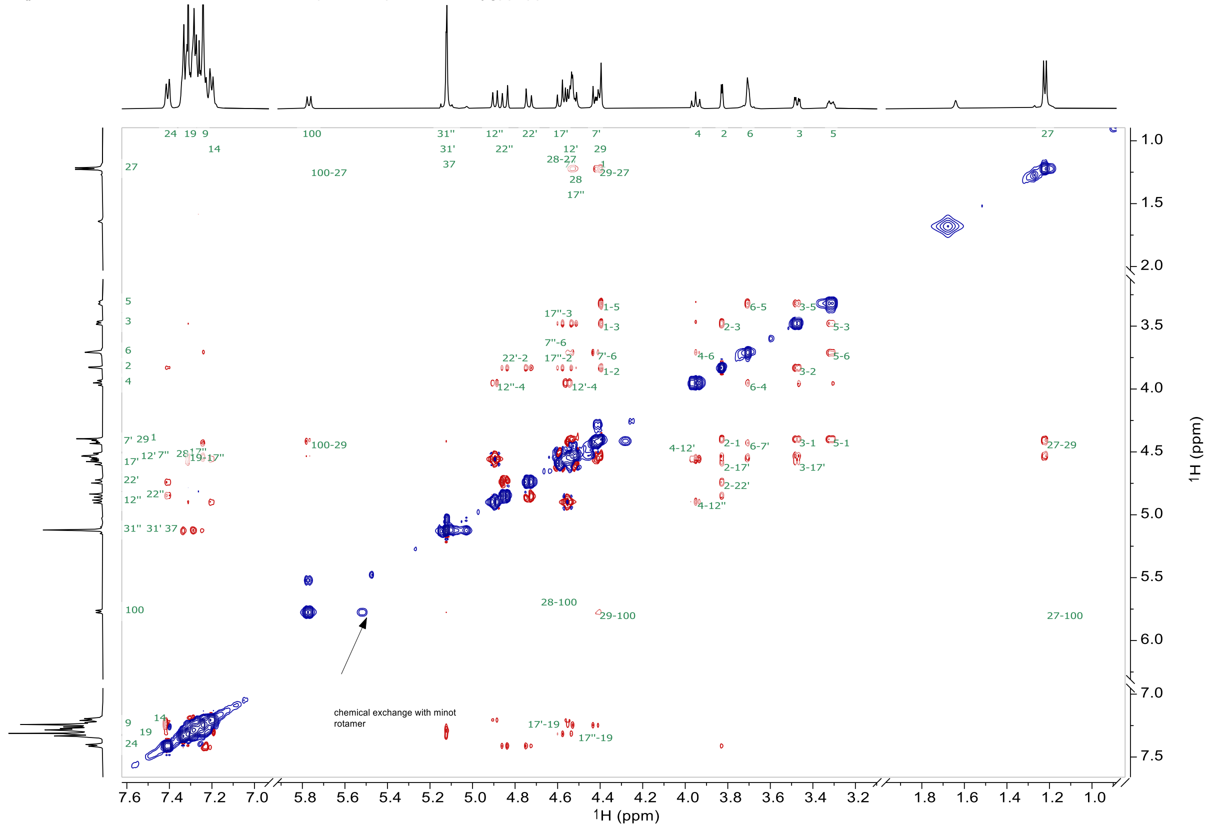

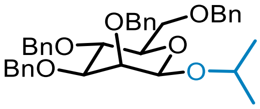

(2*R*,3*R*,4*S*,5*S*,6*R*)-3,4,5-tris(benzyloxy)-2-((benzyloxy)methyl)-6-isopropoxytetrahydro-2*H*-pyran (6h)

Following **General Procedure B**, glycosyl donor **1c** (34 mg, 0.050 mmol) and alcohol **2h** (5 mg, 0.075 mmol) were coupled using (*S*,*S*)-IDPi **3c** (9.4 mg,10 mol%). Reaction time: 72 h. β/α = 86:14 was determined by crude ^{1}H NMR. Flash chromatography (hexane/EtOAc = 12/1 to 8/1) gave **6h** as white solid (24 mg, 82%).

Following **General Procedure A** on 0.01 mmol scale, the glycosylation was carried out using (*R*,*R*)-IDPi **3c**. β/α = 69:31 was determined by crude ^{1}H NMR.

TLC: R_f = 0.76 (Hexane/EtOAc = 3:1)

^{1}H NMR (501 MHz, $CDCl_3$) δ 7.51 – 7.46 (m, 2H), 7.37 – 7.22 (m, 16H), 7.23 – 7.17 (m, 2H), 5.00 (d, *J* = 12.6 Hz, 1H), 4.91 (d, *J* = 10.8 Hz, 1H), 4.90 (d, *J* = 12.6 Hz, 1H), 4.63 (d, *J* = 12.0 Hz, 1H), 4.59 (d, *J* = 12.1 Hz, 1H), 4.54 (d, *J* = 10.8 Hz, 1H), 4.51 (d, *J* = 11.8 Hz, 1H), 4.46 (s, 1H), 4.44 (d, *J* = 11.8 Hz, 1H), 4.02 (hept, *J* = 6.2 Hz, 1H), 3.85 (d, *J* = 3.2 Hz, 1H), 3.84 (t, *J* = 9.4 Hz, 1H), 3.81 (dd, *J* = 10.8, 1.9 Hz, 1H), 3.74 (dd, *J* = 10.8, 6.4 Hz, 1H), 3.51 (dd, *J* = 9.4, 3.2 Hz, 1H), 3.45 (ddd, *J* = 9.7, 6.2, 1.9 Hz, 1H), 1.30 (d, *J* = 6.2 Hz, 3H), 1.16 (d, *J* = 6.1 Hz, 3H).

^{13}C NMR (126 MHz, $CDCl_3$) δ 139.07, 138.71, 138.54, 138.39, 128.62, 128.47, 128.46, 128.42, 128.20, 128.16, 127.94, 127.76, 127.70, 127.67, 127.57, 127.45, 99.77, 82.71, 76.06, 75.26, 75.19, 74.22, 73.85, 73.56, 71.48, 71.29, 70.02, 23.80, 21.89.

$[\alpha]_D^{22}$ = -44.8 ($CHCl_3$, *c* = 1.0).

HRMS m/z (ESI): calcd. for C37 H42 O6 Na1 ($[M+Na]^+$): 605.287359; found: 605.287390.

User Report
LUJ-LA-385-01

The following compound was assigned in the sample

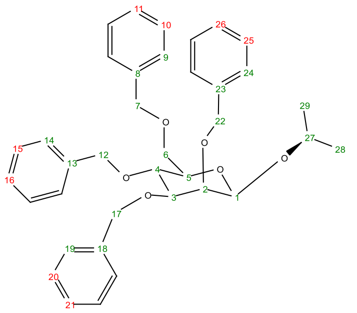

Remarks:
The relative stereochemistry at the anomeric center is supported by NOE correlations from H1 to H3 and H5. The coupling of H1 to H2 could not be resolved and is therefor likely <1Hz.
The aryl rings signals were not assigned in detail due to a lack of resolution of the provided dataset.

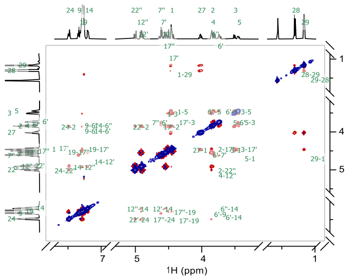

Atom	δ (ppm)	J	COSY	HSQC	HMBC	NOESY
1 C	99.765			1	2, 5, 27	
H	4.463			1	3, 5, 27	2, 3, 5, 27, 29
2 C	74.222			2	3, 22', 22"	
H	3.850	3.20(3)	3	2	1, 3, 22	1, 3, 17', 17", 22', 22", 24
3 C	82.710			3	1, 2, 4, 5, 17', 17"	
H	3.507	9.40(4), 3.20(2)	2, 4	3	2, 4, 17	1, 2, 5, 17', 17"
4 C	75.181			4	3, 5, 6', 6", 12', 12"	
H	3.844	9.70(5), 9.40(3)	3, 5	4	3, 5, 6	12', 12"
5 C	76.065			5	1, 4, 6', 6"	
H	3.450	9.70(4), 6.20(6'), 1.90(6")	4, 6', 6"	5	1, 3, 4, 6	1, 3, 6', 6"
6 C	70.024			6', 6"	4, 5, 7', 7"	
H'	3.735	10.80(6"), 6.20(5)	5, 6"	6	4, 5, 7	5, 7', 7", 9, 14
H"	3.813	10.80(6'), 1.90(5)	5, 6'	6	4, 5, 7	5, 7', 7", 9, 14
7 C	73.563			7', 7"	6', 6", 9	
H'	4.592	12.20(7")	7"	7	6, 8, 9	6', 6", 9
H"	4.627	12.20(7')	7'	7	6, 8, 9	6', 6", 9
8 C	138.703				7', 7"	
9 C	127.944			9	7', 7", 9	
H	7.343			9	7, 9	6', 6", 7', 7"
10 C						
H						
11 C						
H						
12 C	75.255			12', 12"	14	
H'	4.544	10.80(12")	12"	12	4, 13, 14	4, 14
H"	4.911	10.80(12')	12'	12	4, 13, 14	4, 14
13 C	138.543				12', 12"	
14 C	128.199			14	12', 12", 14	
H	7.199			14	12, 14	6', 6", 12', 12"
15 C						
H						
16 C						
H						
17 C	71.484			17', 17"	3, 19	
H'	4.440	11.80(17")	17"	17	3, 18	2, 3
H"	4.512	11.80(17')	17'	17	3, 18	2, 3, 24
18 C	138.391				17', 17"	
19 C						
H	7.285				17	
20 C						
H						
21 C						
H						
22 C	73.847			22', 22"	2, 24	
H'	4.899	12.60(22")	22"	22	2, 23, 24	2, 24
H"	5.003	12.60(22')	22'	22	2, 23, 24	2, 24
23 C	139.068				22', 22"	
24 C	128.621			24	22', 22", 24	
H	7.484			24	22, 24	2, 17", 22', 22"
25 C						
H						
26 C						
H						
27 C	71.292			27	1, 28, 29	
H	4.019	6.20(28), 6.10(29)	28, 29	27	1	1
28 C	23.802			28	29	
H3	1.304	6.20(27)	27	28	27, 29	29
29 C	21.891			29	28	
H3	1.164	6.10(27)	27	29	27, 28	1, 28

P-ID: ML00000
Measured on: 10/01/2026
CHIFFRE: LUJ-LA-385-01
ELNA#: 16032
Client: Jiaxiang Lu
Group: List
Spectroscopist: Leutzsch
Analysed on: 19/02/2026
Analysed by: Leutzsch
Amount: 20.0 mg
Solvent: CDCl3
Reference: 1H+13C on solvent,other nuclei w/ xiref
Temperature: 298 K
Spectrometer: AV500iis
Probe: prodigy-BBO
Experiments: 1H-1H-noesygpphpp, 1H-zg30, 13C-zgpg30, 1H-1H-cosygpppqf, 1H-13C-hsqcedetgpsisp2.3, 1H-13C-hmbcetgpl3nd, 1H-1H-noesygpphpp

1H{},1D, 500.81 MHz,CDCl3,298.0K, pulse sequence: zg30

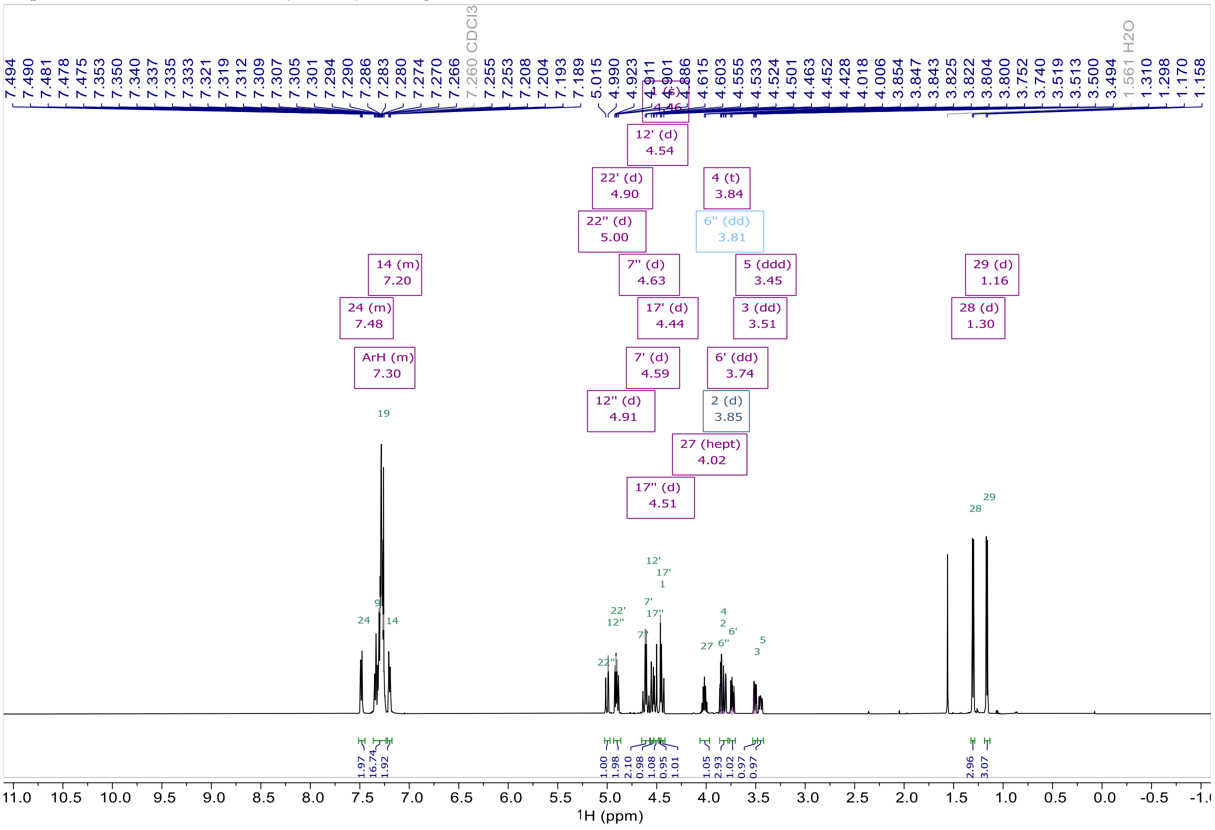

1H{},1D, 500.81 MHz,CDCl3,298.0K, pulse sequence: zg30

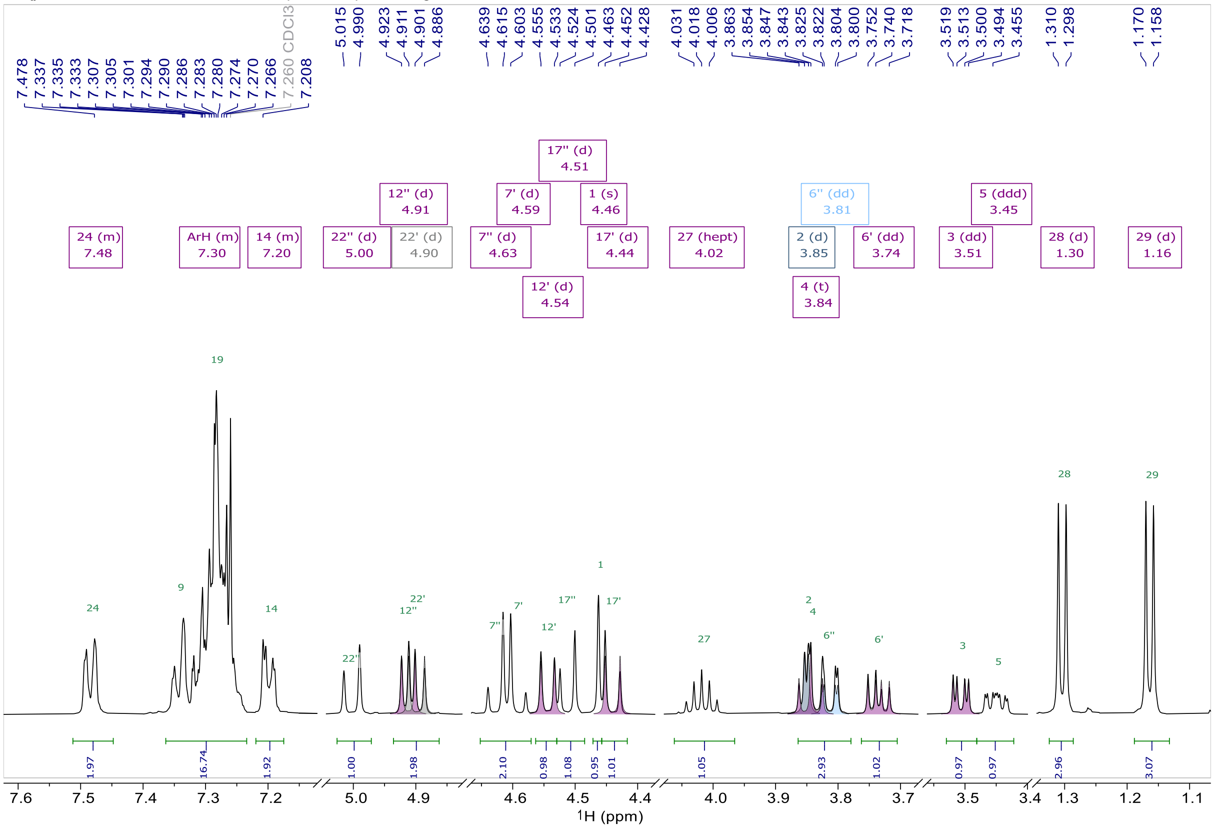

13C{},1D, 125.94 MHz,CDCl3,298.0K, pulse sequence: zgpg30

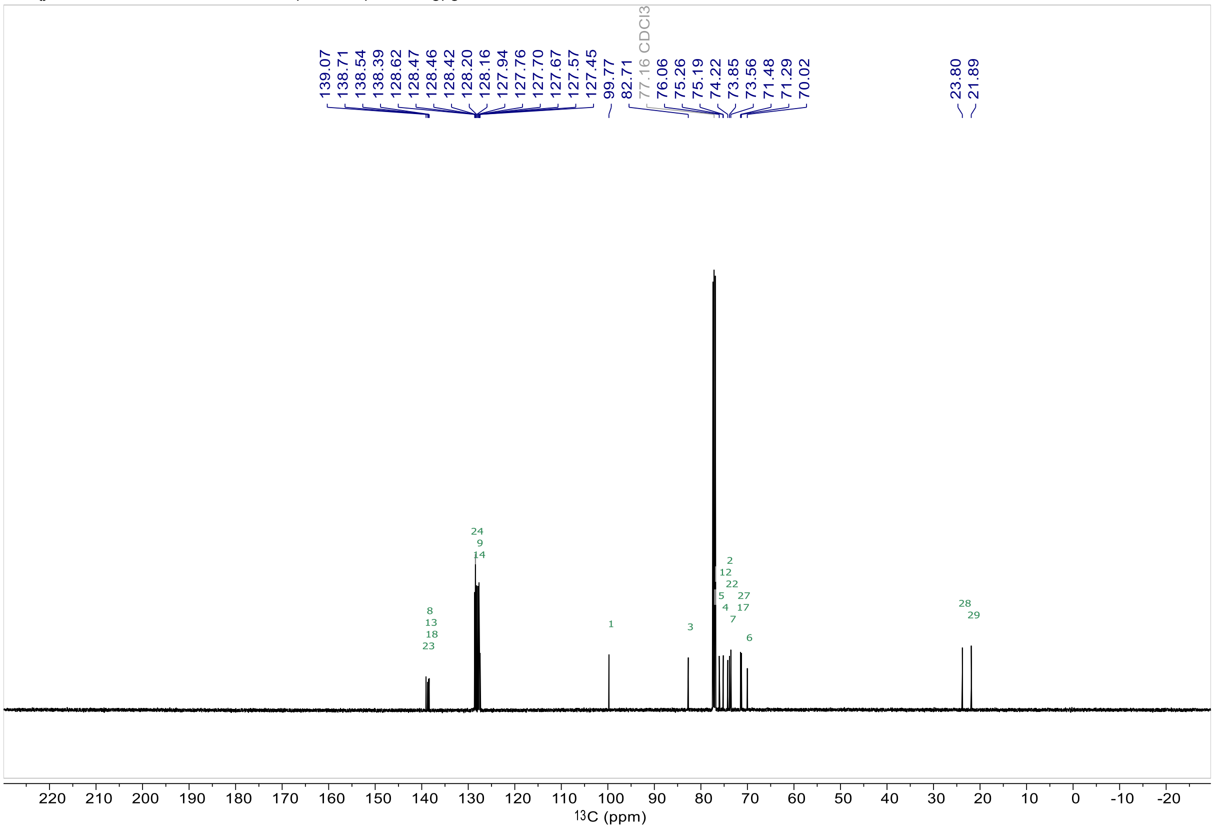

1H{},COSY, 500.81 MHz,CDCl3,298.0K, pulse sequence: cosygpppqf

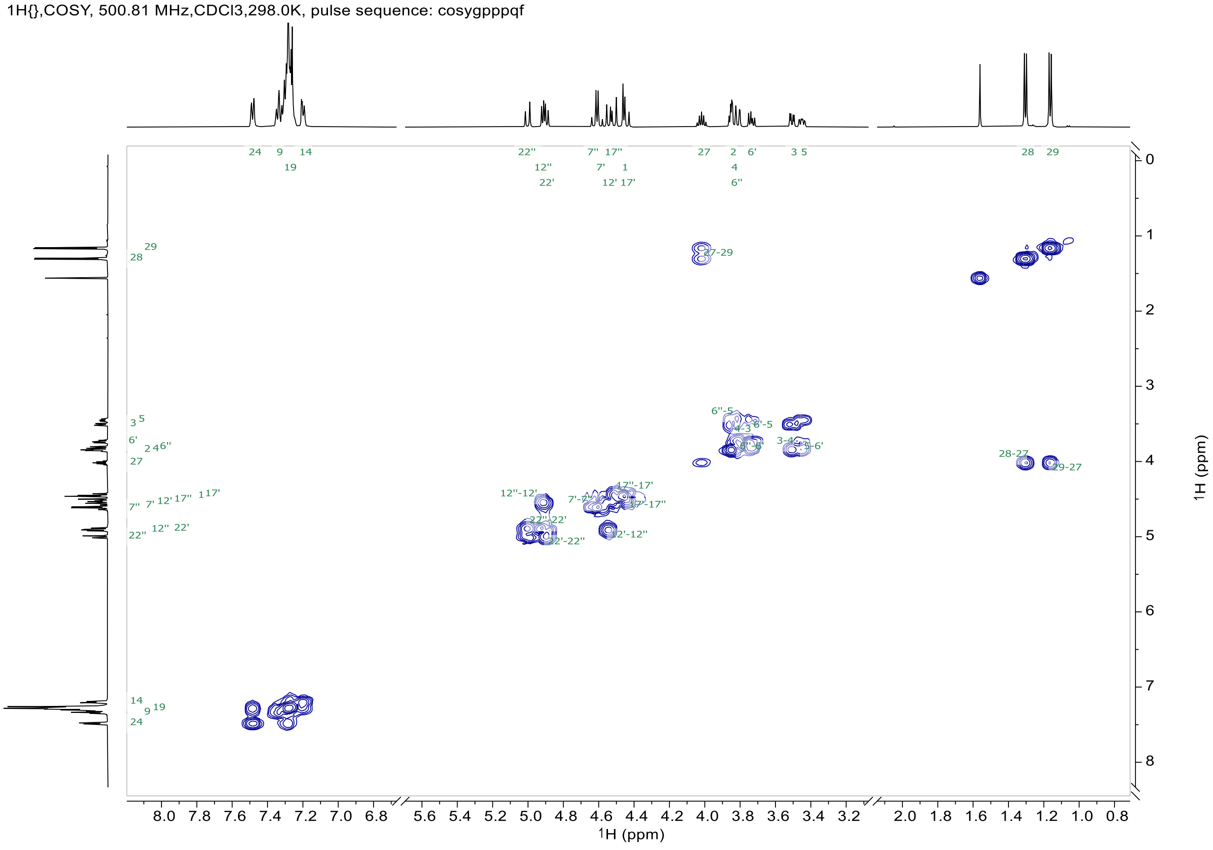

1H{},HSQC-EDITED, 500.81 MHz,CDCl3,298.0K, pulse sequence: hsqcedetgpsisp2.3

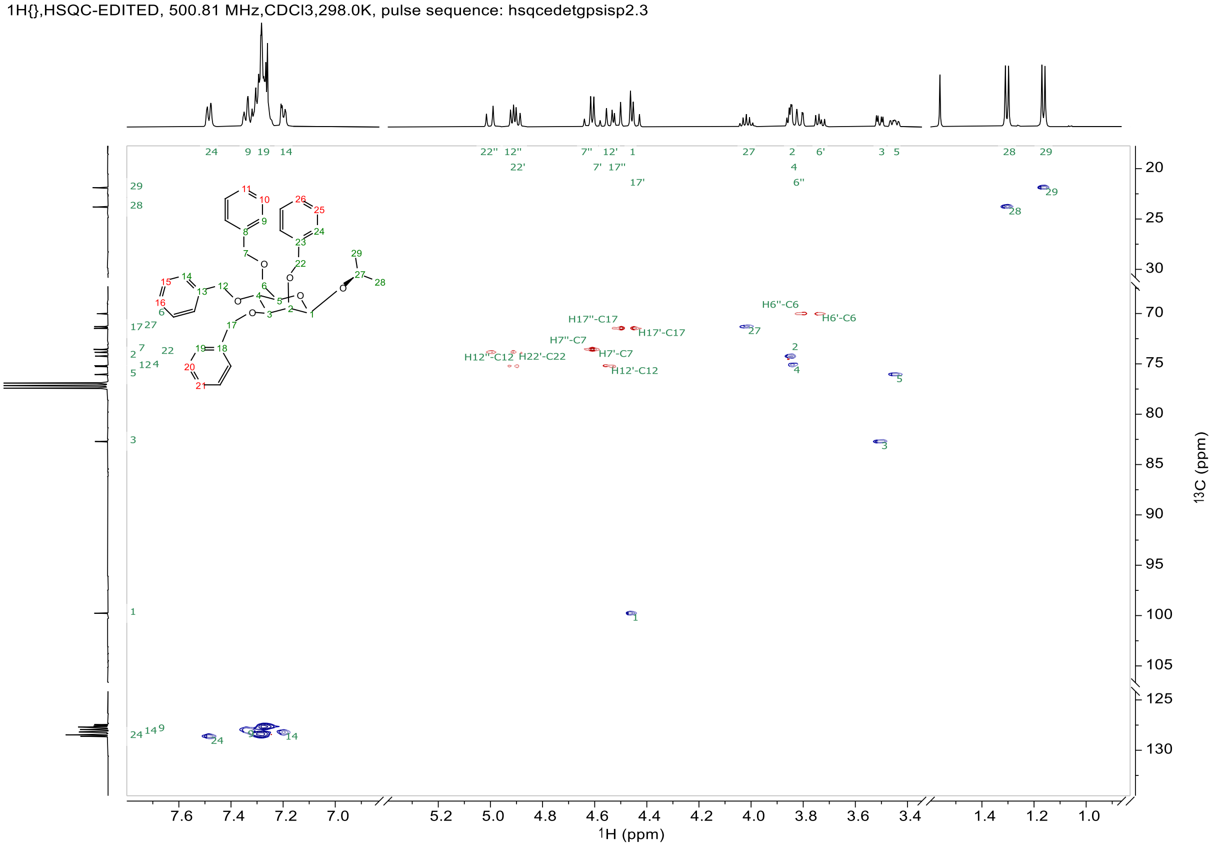

1H{},HMBC, 500.81 MHz,CDCl3,298.0K, pulse sequence: hmbcetgpl3nd

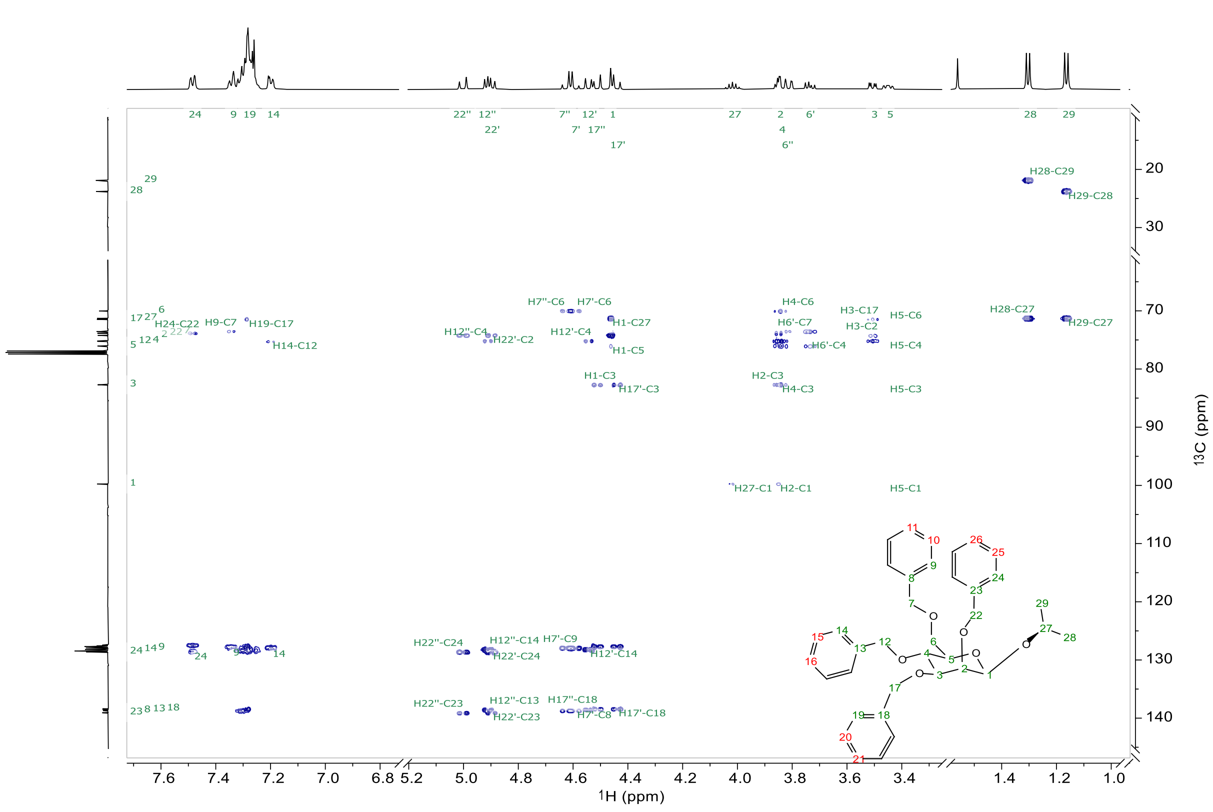

1H{},NOESY, 500.81 MHz,CDCl3,298.0K, pulse sequence: noesygpphpp

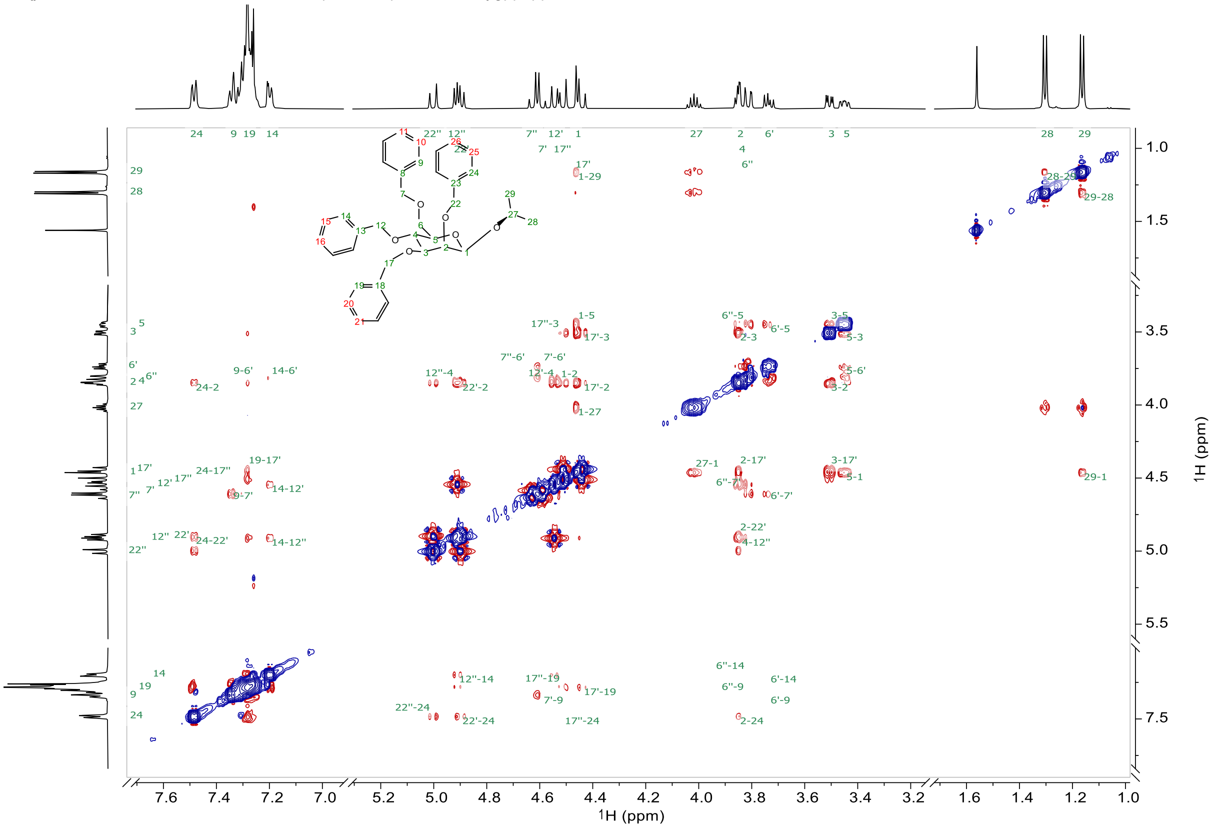

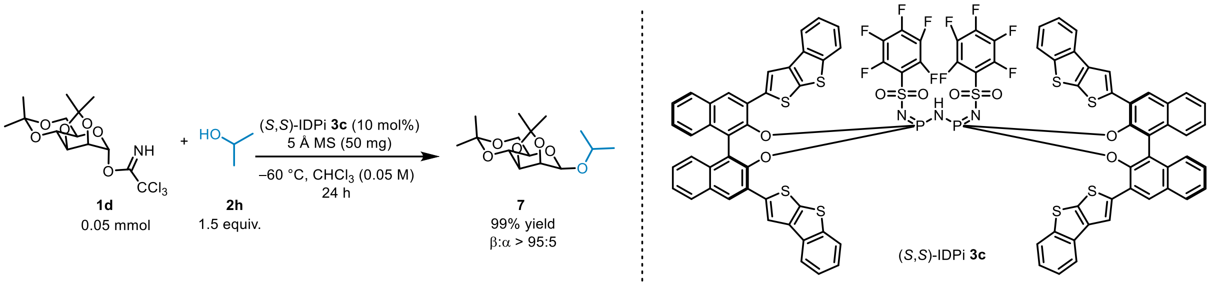

Following **General Procedure B**, glycosyl donor **1d** (20 mg, 0.050 mmol) and alcohol **2h** (5 mg, 0.075 mmol) were coupled using (*S*,*S*)-IDPi **3c** (9.4 mg,10 mol%). Reaction time: 24 h. β/α = > 95:5 was determined by crude ^{1}H NMR. Flash chromatography (hexane/EtOAc = 10/1 to 6/1) gave (3a*S*,4*R*,5a*R*,9a*R*,9b*S*)-4-isopropoxy-2,2,8,8-tetramethylhexahydro-[1,3]dioxolo[4',5':4,5]pyrano[3,2-*d*][1,3]dioxine **7** as colorless oil (15 mg, 99%). The NMR data of this compound are consistent with those reported in the literature.[10]

TLC: R_f = 0.74 (Hexane/EtOAc = 3:1)

^{1}H NMR (501 MHz, CDCl$_3$) δ 1.20 (d, *J* = 6.1 Hz, 3H), 1.29 (d, *J* = 6.2 Hz, 3H), 1.38 (s, 3H), 1.42 (s, 3H), 1.51 (s, 3H), 1.57 (s, 3H), 3.24 (td, *J* = 10.3, 5.5 Hz, 1H), 3.78 (t, *J* = 10.5 Hz, 1H), 3.91 (dd, *J* = 10.8, 5.5 Hz, 1H), 3.95 – 4.03 (m, 1H), 4.06 (dd, *J* = 10.3, 7.5 Hz, 1H), 4.14 (dd, *J* = 7.5, 6.0 Hz, 1H), 4.23 (dd, *J* = 6.0, 2.6 Hz, 1H), 4.85 (d, *J* = 2.6 Hz, 1H).

^{13}C NMR (126 MHz, CDCl$_3$) *δ* 19.07, 21.87, 23.73, 26.30, 27.89, 29.17, 62.78, 66.09, 72.21, 72.23, 75.08, 76.59, 97.46, 99.76, 111.24.

$[\alpha]_D^{22}$ = -113.4 (CHCl$_3$, *c* = 1.0).

HRMS m/z (ESI): calcd. for C15 H26 O6 Na1 ([M+Na]$^+$): 325.162159; found: 325.162460.

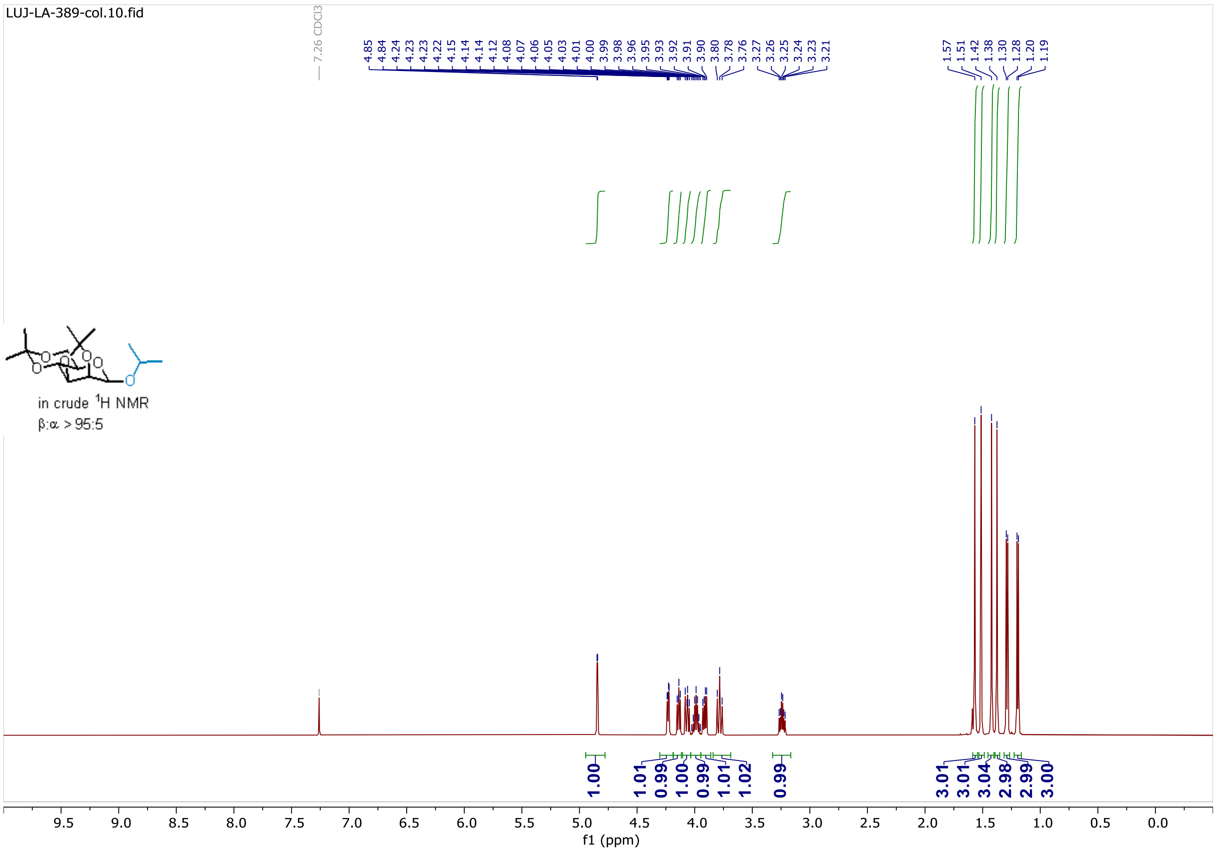
LUJ-LA-389-col.10.fid
in crude ¹H NMR
β:α > 95:5
f1 (ppm)

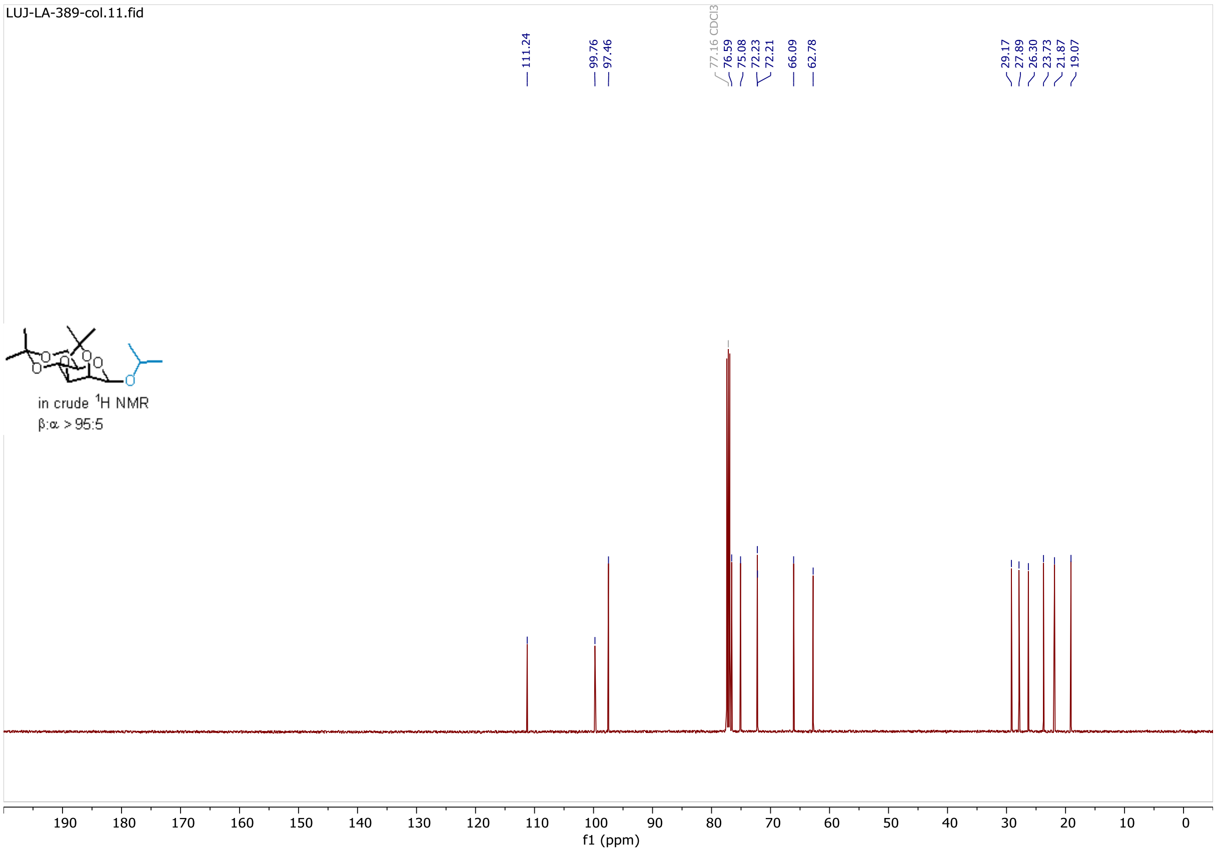
LUJ-LA-389-col.11.fid
in crude ¹H NMR
β:α > 95:5
f1 (ppm)

5. Mechanistic studies

Starting from β-Glycosides

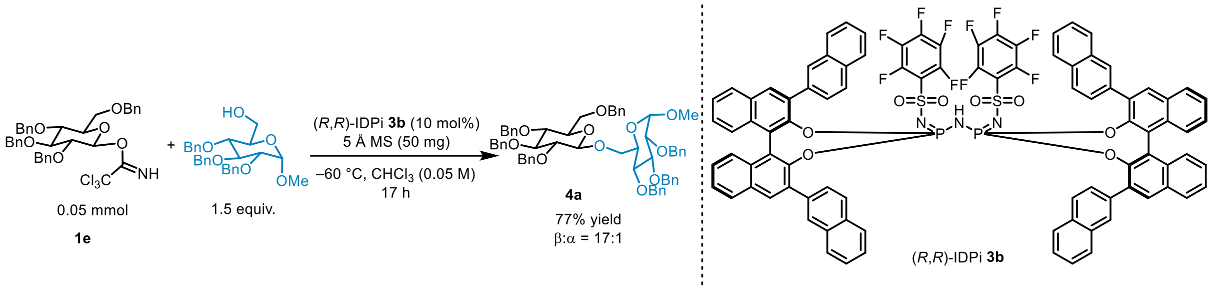

Following **General Procedure B**, glycosyl donor **1e** (34 mg, 0.050 mmol) and alcohol **2a** (35 mg, 0.075 mmol) were coupled using (*R*,*R*)-IDPi **3b** (7.5 mg,10 mol%). Reaction time: 17 h. β/α = 94:6 was determined by crude ^{1}H NMR. Flash chromatography (hexane/EtOAc = 6/1 to 3/1) gave **4a** as white solid (38 mg, 77%).

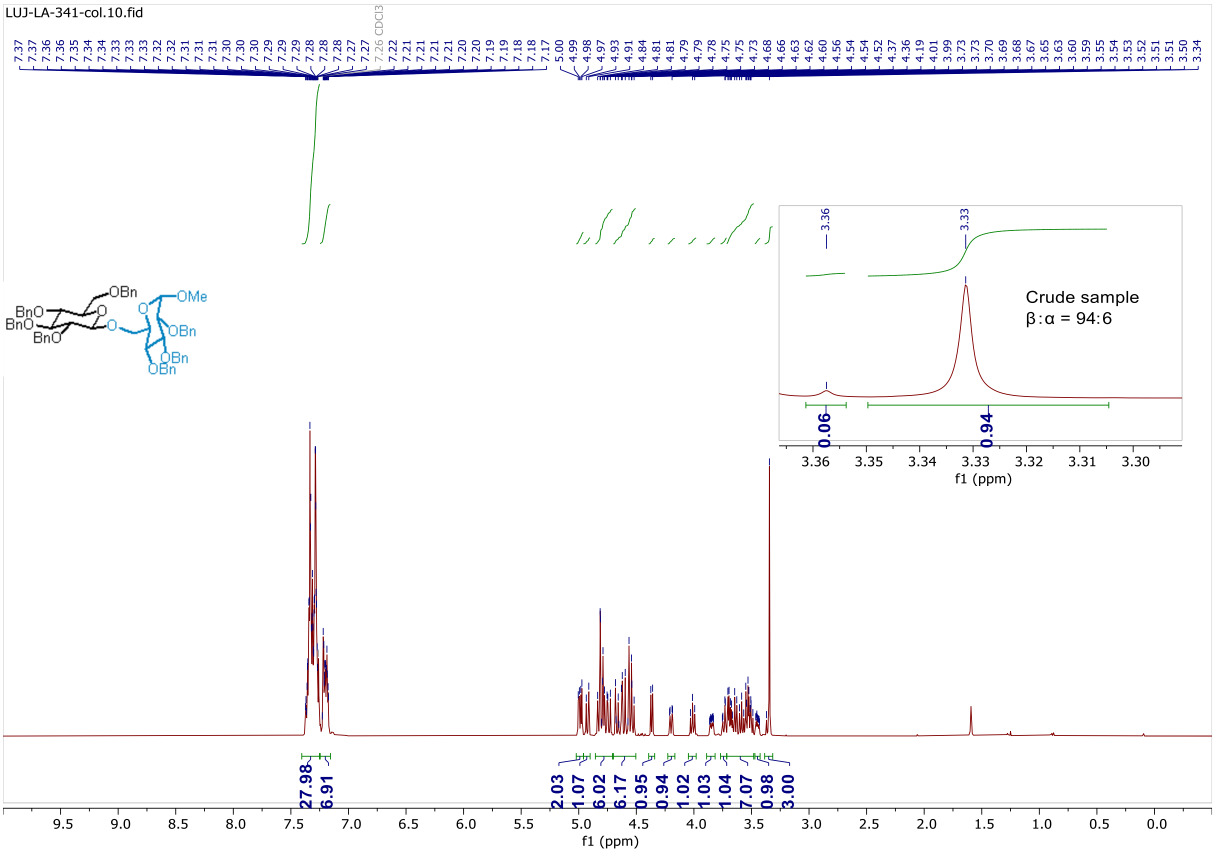

2nd parallel KIE

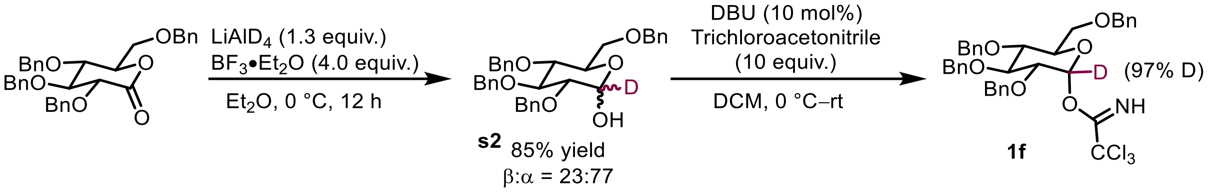

Step i: A suspension of $LiAlD_4$ (300 mg, 7.16 mmol) in anhydrous Et_2O (50 mL) was cooled to 0 °C and stirred under an inert atmosphere. A solution of the substrate (3.0 g, 5.54 mmol) and BF_3Et_2O (3mL, 24.5 mmol) in anhydrous Et_2O (50 mL) was added dropwise to the suspension at 0 °C. The reaction mixture was stirred for 3 h at 0 °C. Excess $LiAlD_4$ was carefully quenched by dropwise addition of MeOH. The mixture was washed with H_2O (3 × 30 mL), saturated aqueous $NaHCO_3$, and brine, dried over Na_2SO_4, filtered, and concentrated under reduced pressure to afford a colorless solid. Recrystallization from cyclohexane/EtOH (3:1, v/v) gave the pure product **s2** as white solid.

Step ii: To a solution of the glycosyl hemiacetal **s2** (1.8 mmol,1.0 equiv) in dry CH_2Cl_2 (0.1 M) were added CCl_3CN (1.8 mL,10.0 equiv) and DBU (270 μL,10 mol%) at ice-bath. The reaction mixture was stirred at room temperature for 4 h. Upon completion, the mixture was concentrated in vacuo and purified by silica gel column chromatography (EtOAc/hexanes = 99:1 to 95:5, 1% TEA) to afford 1.15 g white solid of glycosyl β-trichloroacetimidates **1f**, 91% yield.

TLC: R_f = 0.77 (Hexane/EtOAc = 3:1)

^{1}H NMR (501 MHz, $CDCl_3$) δ 3.60 (dd, *J* = 11.0, 2.0 Hz, 1H), 3.67 – 3.77 (m, 3H), 3.93 (ddd, *J* = 10.1, 3.2, 2.0 Hz, 1H), 3.99 (t, *J* = 9.3 Hz, 1H), 4.39 (d, *J* = 12.1 Hz, 1H), 4.46 (d, *J* = 10.7 Hz, 1H), 4.52 (d, *J* = 12.0 Hz, 1H), 4.60 (d, *J* = 11.7 Hz, 1H), 4.67 (d, *J* = 11.7 Hz, 1H), 4.78 (dd, *J* = 14.6, 10.8 Hz, 2H), 4.89 (d, *J* = 11.0 Hz, 1H), 7.04 – 7.12 (m, 2H), 7.12 – 7.31 (m, 18H), 8.51 (s, 1H).

^{13}C NMR (126 MHz, $CDCl_3$) δ 161.5, 138.8, 138.2, 138.1, 138.0, 128.6, 128.5, 128.5, 128.5, 128.2, 128.1, 128.1, 128.0, 128.0, 127.9, 127.9, 127.9, 127.8, 127.7, 81.5, 79.4, 76.9, 75.8, 75.5, 73.6, 73.3, 73.0, 68.2.

HRMS m/z (ESI): calcd. for C36 H35 D1 Cl3 N O6 Na1 ($[M+Na]^+$): 707.15632; found: 707.15635.

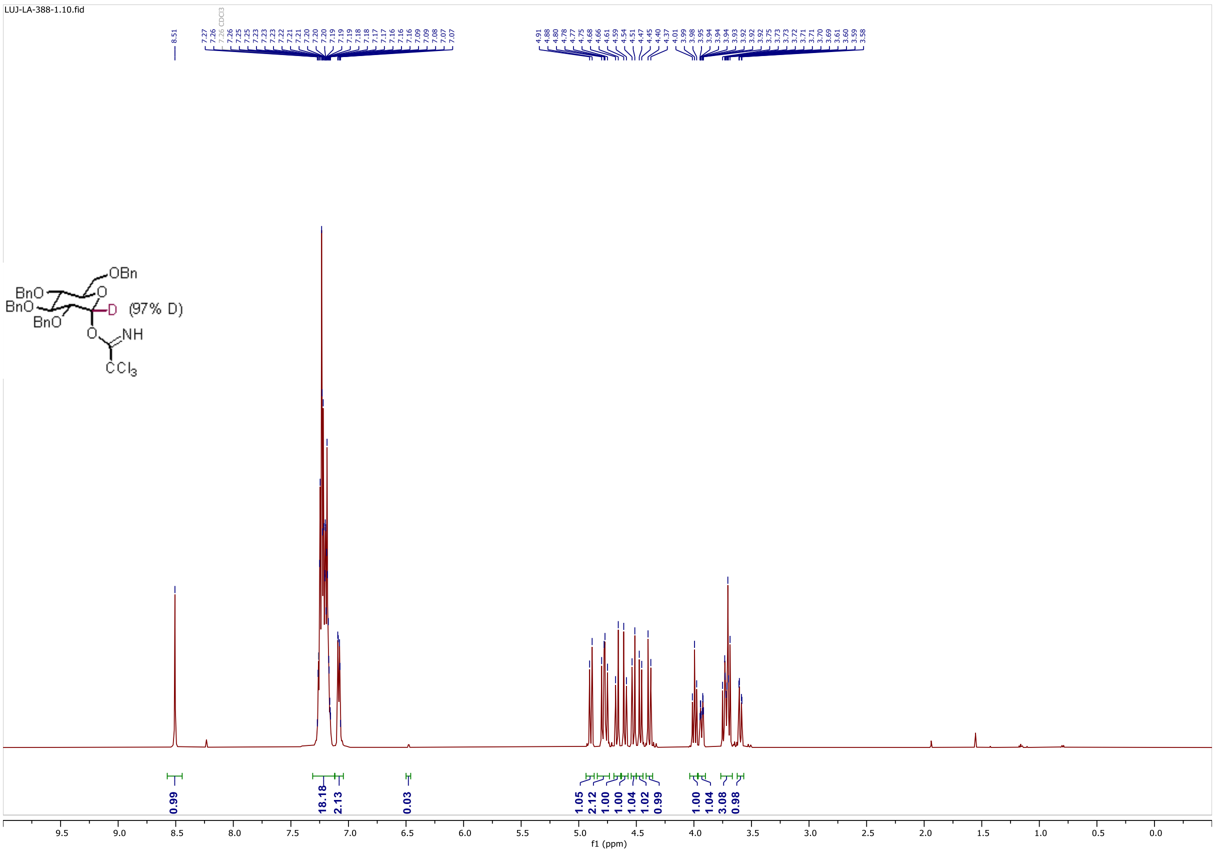
LUJ-LA-388-1.10.fid
8.51
7.27 7.26 CDCl3 7.26 7.25 7.25 7.23 7.23 7.22 7.21 7.21 7.20 7.20 7.19 7.19 7.19 7.18 7.18 7.17 7.17 7.16 7.16 7.09 7.09 7.08 7.07 7.07
4.91 4.88 4.80 4.78 4.77 4.75 4.68 4.66 4.61 4.59 4.54 4.51 4.47 4.45 4.40 4.37 4.01 3.99 3.98 3.95 3.94 3.94 3.93 3.92 3.92 3.92 3.75 3.73 3.73 3.72 3.71 3.71 3.70 3.69 3.61 3.60 3.59 3.58
BnO
BnO
BnO
OBn
O
D
(97% D)
NH
CCl3
0.99
18.18
2.13
0.03
1.05
2.12
1.00
1.00
1.04
1.02
0.99
1.00
1.04
3.08
0.98
9.5 9.0 8.5 8.0 7.5 7.0 6.5 6.0 5.5 5.0 4.5 4.0 3.5 3.0 2.5 2.0 1.5 1.0 0.5 0.0
f1 (ppm)

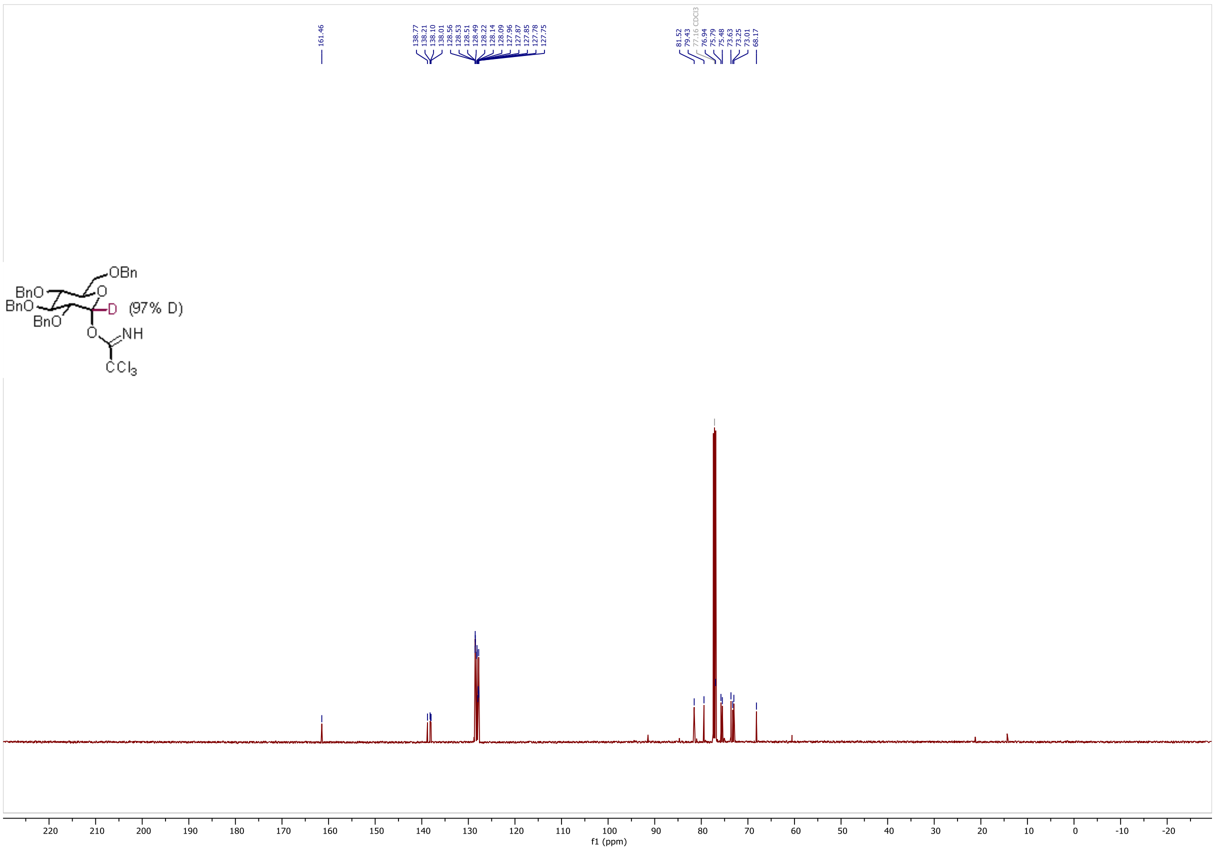
161.46
138.77 138.21 138.10 138.01 128.56 128.53 128.51 128.49 128.22 128.14 128.09 127.96 127.87 127.85 127.78 127.75
81.52 79.43 77.16 CDCl3 76.94 75.79 75.48 73.63 73.25 73.01 68.17
BnO
BnO
BnO
OBn
O
D
(97% D)
NH
CCl3
220 210 200 190 180 170 160 150 140 130 120 110 100 90 80 70 60 50 40 30 20 10 0 -10 -20
f1 (ppm)

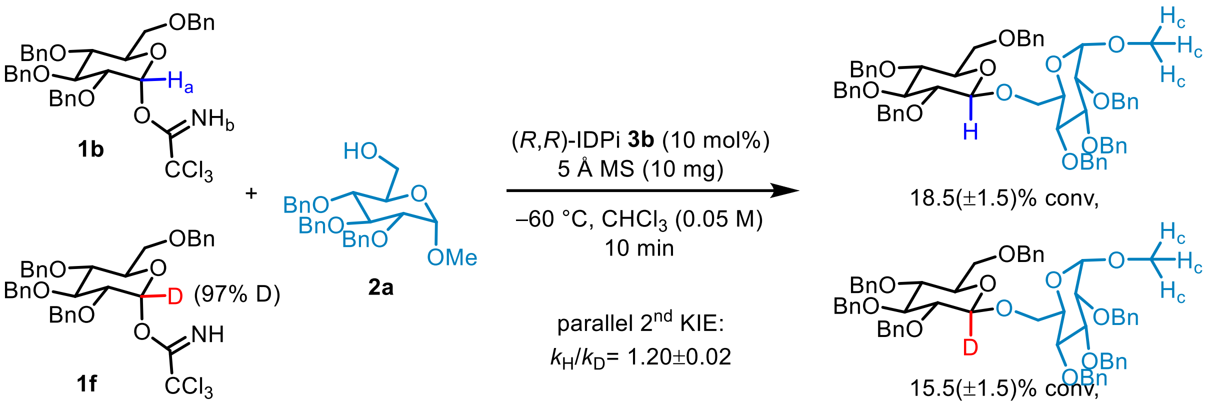

In an oven-dried 2 mL vial equipped with a magnetic stir bar were placed the glycosyl donor **1b** or **1f** (0.01 mmol, 1.0 equiv.), alcohol **2a** (0.015 mmol, 1.5 equiv.), and activated 5 Å molecular sieves (10 mg). Anhydrous $CDCl_3$ (80 μL) was added, and the resulting suspension was stirred at −60 °C for 5 min (1000 rpm). In parallel, the IDPi catalyst (10 mol%) was dissolved in anhydrous $CHCl_3$ (20 μL), precooled to −60 °C, and transferred via syringe to the reaction vial. The mixture was stirred at −60 °C for 10 min. Et_3N as internal standard (in $CDCl_3$) was added to quench the reaction, and the mixture was stirred for an additional 5 min at −60 °C. The reaction mixture was filtered through a short pad of Celite and rinsed with $CDCl_3$ (0.6 mL). The filtrate was analyzed directly by ^1H NMR to determine the conversion and β/α anomeric ratio.

Start material:

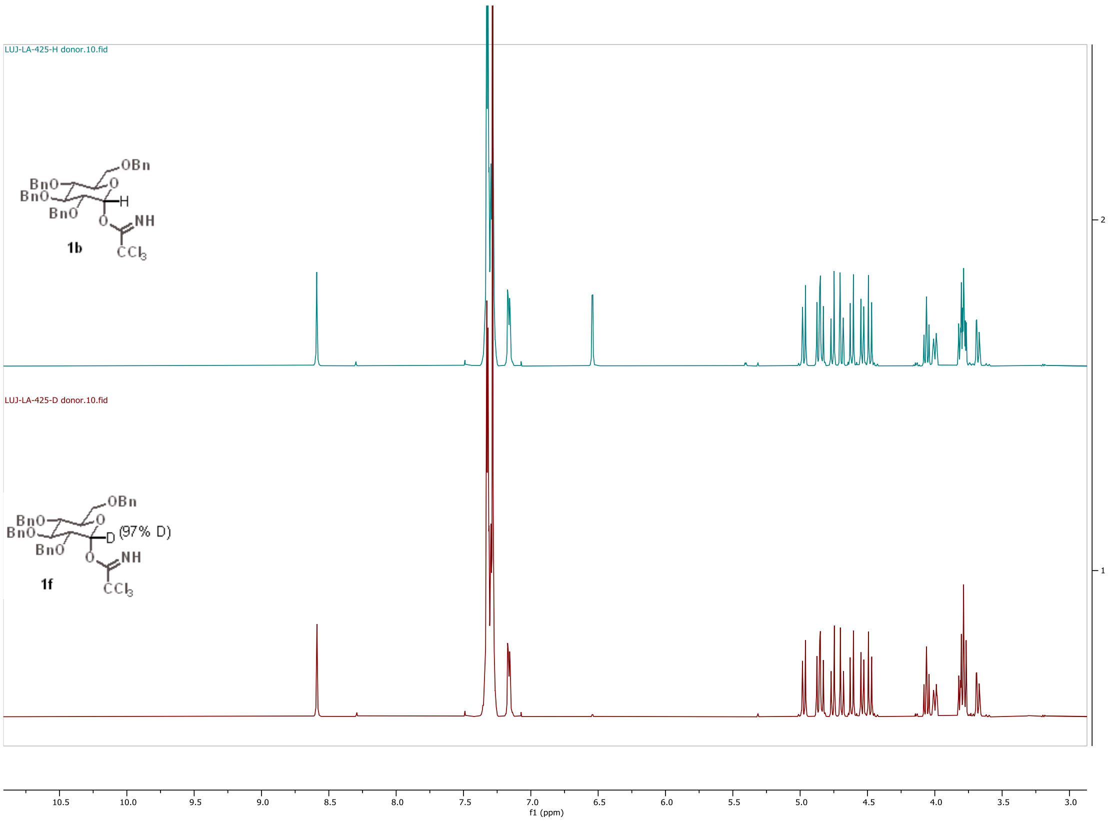

After reaction:

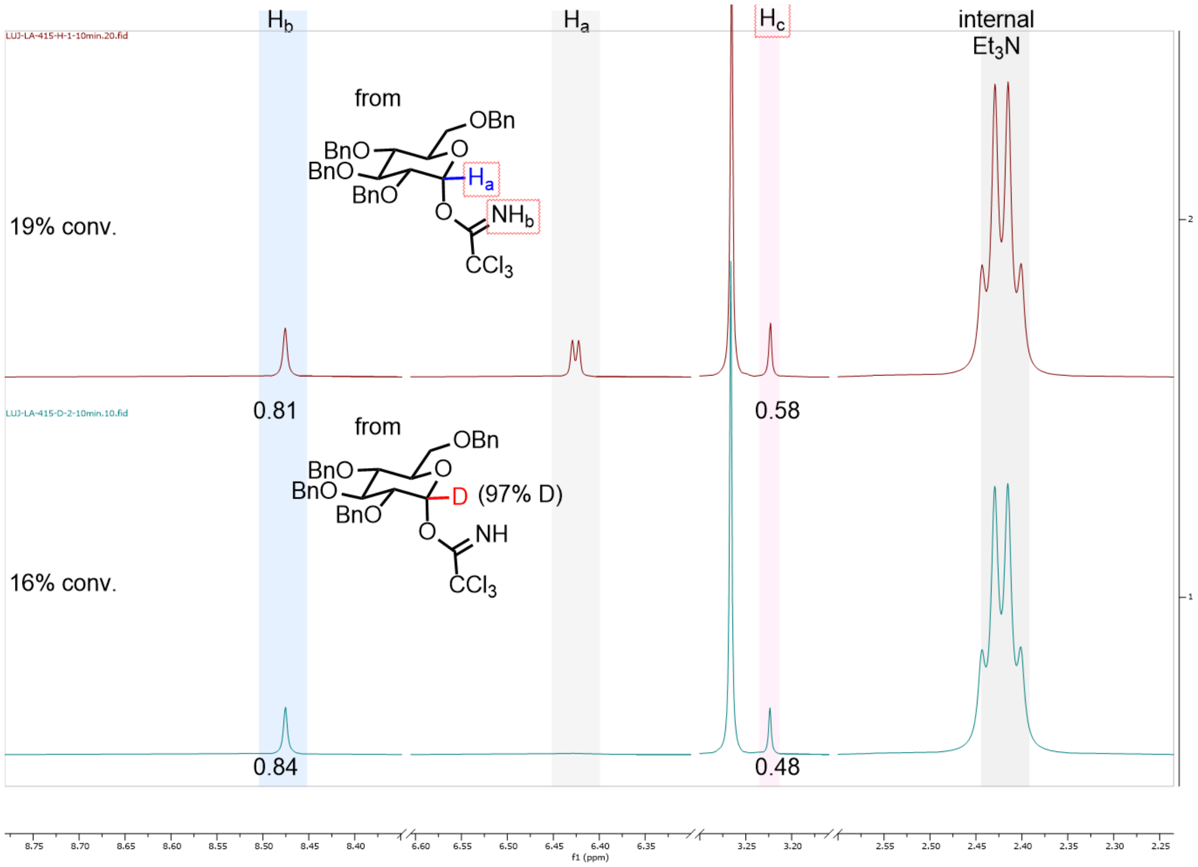

entry	time/ min	conv. (%) of 1b	conv. (%) of 1f	2nd KIE
1	10	19	16	1.19
2	10	20	17	1.18
3	10	17	14	1.22
4	10	19	16	1.19

After four independent measurements, a secondary H/D KIE of 1.20 ± 0.02 was obtained.

Computational details

Based on experimental results, glycosyl donor **1d** reacted with isopropanol **2h** in the presence of (*S*,*S*)-IDPi **3c** to afford product **7** in 99% yield with a β/α ratio of >95:5. This transformation was therefore selected for theoretical calculations.

Method:

Conformations of all equilibrium and transition-state structures were sampled using the artificial force-induced reaction (AFIR) method implemented in the global reaction route mapping (GRRM) program.[11–13] Extensive conformational sampling of possible catalyst-substrate orientations was performed at the GFN2-xTB level of theory implemented in ORCA 4.2.0 using the single-component AFIR (SC-AFIR) method with constraints.[14,15] Molecular geometries were optimized at the r^2SCAN-3c level of theory as implemented in ORCA 5.0.3.[16,17] Thermal free-energy corrections were computed at the same level using ORCA 5.0.4, with the temperature set to 213.15 K and the pressure set to 1 atm.[7] Transition-state structures were verified by the presence of a single imaginary vibrational frequency. Solvation effects were accounted for using the CPCM (chloroform) solvation model implemented in ORCA 5.0.4.[18]

The independent gradient model based on the Hirshfeld partition (IGMH) method, as implemented in the Multiwfn program, was employed with ωB97X-D/def2-TZVPP level of theory obtained using Gaussian 16 package.[19] IGMH analysis was performed using Multiwfn 3.8 (dev) with default parameters and a grid resolution of 0.15 Bohr and an isovalue of 0.005 a.u.[20-22] Visualizations were generated using ChimeraX (version 1.7), followed by rendering in Blender (version 4.0).[23-25]

Energy diagram for *O*-glycosidic bond formation

In addition to the stepwise pathway, another possible pathway that does not involve intermediate **c**, namely an asynchronous concerted pathway, is also considered. This pathway is depicted as **TS1″** in **Figure S1**. Its energy is higher than that of the stepwise pathway, suggesting that this reaction likely proceeds via a dissociative S_N1-like mechanism, although the possibility of an associative S_N2-like pathway cannot be fully excluded.

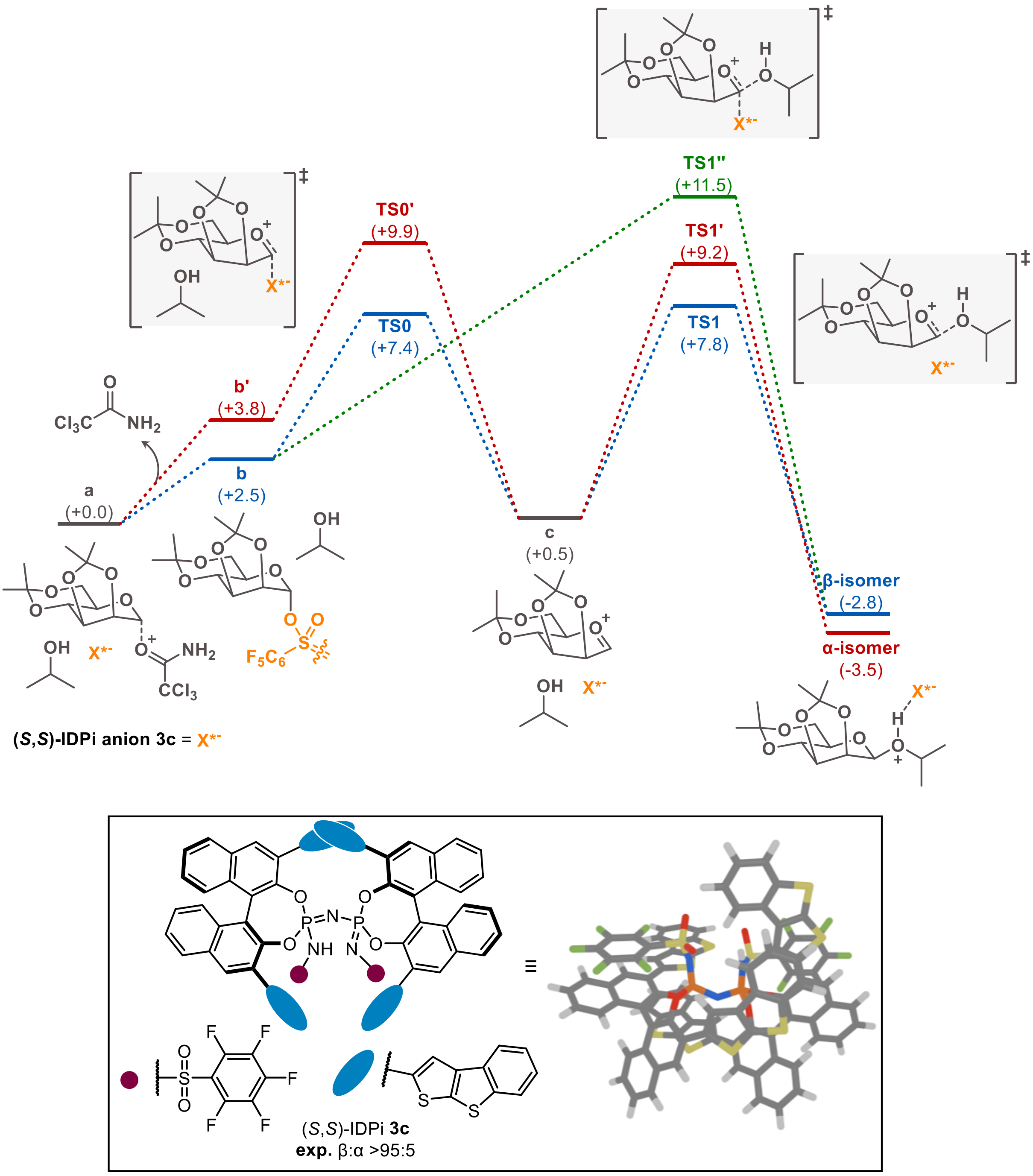

Figure S1: Energy diagram of the *O*-glycosidic bond formation step leading to the β- and α-glycoside product. The dotted line in blue is the pathway leading to the β-

glycoside product and dotted line in red is the pathway leading to the α-glycoside product.

Options for Minimally augmented def2-TZVPP basis set:

In order to better describe diffuse electron density, minimally augmented def2-TZVPP basis sets were applied to chemically important heteroatoms in the single-point calculations. Specifically, 15 heteroatoms, including P, N, S, and O atoms in the IDPi catalyst, were calculated at the ωB97M-V/ma-def2-TZVPP level of theory, while all other atoms were calculated at the ωB97M-V/def2-TZVPP level of theory (**Figure S2**).[26,27]

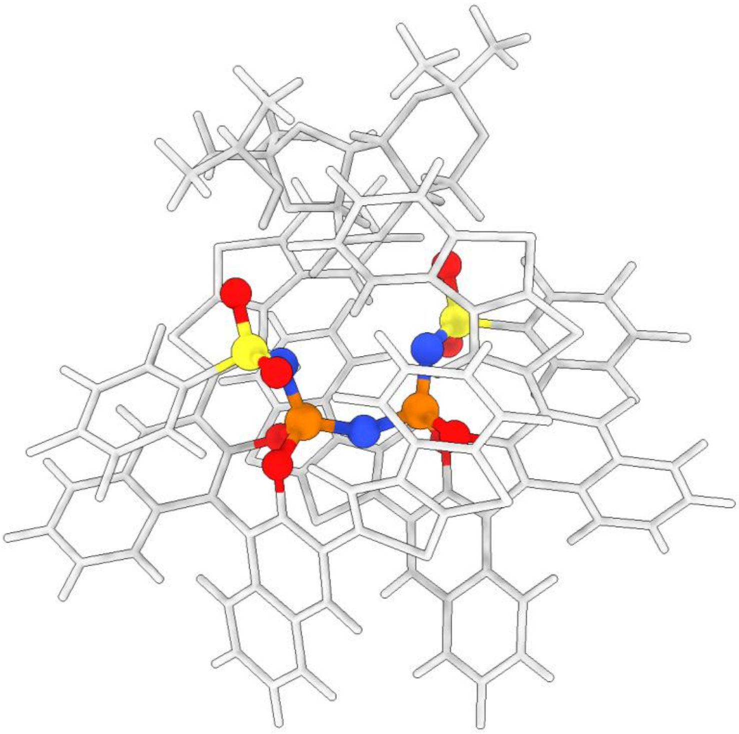

Figure S2: Atoms specified for the application of minimally augmented def2-TZVPP basis sets. The colored spherical atoms indicate heteroatoms that were calculated at the ωB97M-V/ma-def2-TZVPP level of theory, while all other atoms were calculated at the ωB97M-V/def2-TZVPP level of theory.

Distortion interaction analysis

The distortion-interaction analysis following Houk-Bickelhaupt protocol was conducted on **TS1** and **TS1′** to analyze deeper into the origin of stereoselectivity.[28] The optimized TS conformation was separated into oxocarbenium ion-glycol acceptor complex and anionic IDPi catalyst, followed by single-point energy calculations performed on these ionic structures. The calculations suggested that the predominant factor influencing stereoselectivity was the distortion effect, mainly attributed from the distortion of the catalyst anion, which had a more significant impact than the interaction effect (**Table S8**). In other words, the energy difference between the oxocarbenium ions in the two

TSs is only 1.79 kcal/mol, which is much smaller than the corresponding energy difference of the catalyst anion (6.05 kcal/mol). Therefore, it can be concluded that the conformation of the oxocarbenium ion is not a predominant factor in determining stereoselectivity; rather, the conformation of the catalyst plays the primary role.

The IDPi anion geometries of **TS1** and **TS1′** were extracted and superimposed to compare structural differences (**Figure S3**). It seems that there are relatively strong π-π interactions between heteroaromatic BINOL substituents in **TS1** (shown in green) compared with the distorted substituents observed in **TS1′** (shown in pink). This distortion likely arises from the oxocarbenium ion attempting to align itself within the catalytic pocket to form an *O*-glycosidic bond with *i*-PrOH, sacrificing the π- π interactions and thereby increasing the energy of **TS1′**.

Table S8: Distortion interaction analysis at the ωB97M-V/(ma)-def2-TZVPP level of theory using an implicit solvent model of chloroform for **TS1** and **TS1′**.

	Single Point Energy (Hartree)	Relative Energy (kcal/mol)
**TS1**	-10998.927917150770	-0.933084279
**TS1′**	-10998.926430187526	
Substrate Only		
Substrate_**TS1**	-1038.962564562675	-1.789257961
Substrate_**TS1′**	-1038.959713201008	
Catalyst Only		
Catalyst_**TS1**	-9959.851785757570	-6.049267954
Catalyst_**TS1′**	-9959.842145643544	
Total Distortion	(predominant factor)	-7.838525916
Total Interaction		+6.905441637

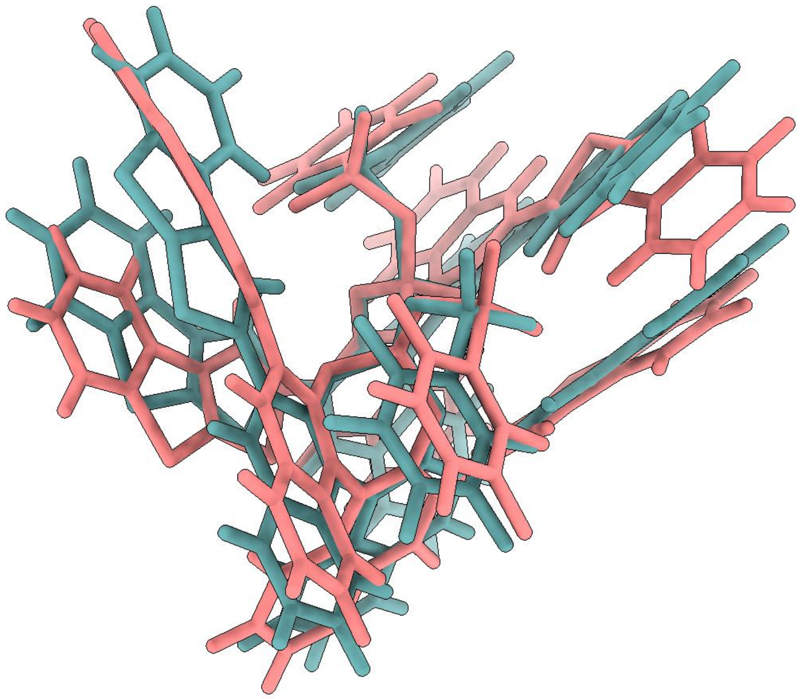

Figure S3: Superimposed IDPi anion structures of **TS1** (colored green) and **TS1′** (colored in pink).

IGMH analysis

The IGMH analysis was conducted on the two IDPi anion structures from **TS1** and **TS1′** to examine how distortion of the BINOL substituents affects the weak interactions. The IDPi anion was divided into five parts, and the intrinsic bond strength index for weak interactions (IBSIW) obtained from the IGMH analysis between parts 1 and 4, and parts 2 and 3, was calculated and compared for the two IDPi anions (**Figure S4**). Parts 1-4 consist of 32 atoms in which half a BINOL and corresponding BINOL substituent is included, and part 5 being the rest of the IDPi anion. The sum of IBSIW for the two pairs showed that the interactions are stronger in **TS1** than in **TS1′**, indicating that distortion of the IDPi anion arises from the absence of π- π interactions between heteroaromatic BINOL substituents (**Table S9**). The calculated weak interactions between the BINOL substituents are visualized in **Figure S5**.

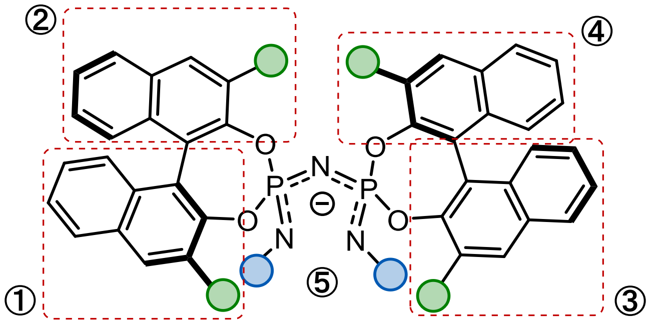

Figure S4: IDPi anion geometry from **TS1** and **TS1′** was divided into 5 parts and the weak interactions between parts 1 and 4, and parts 2 and 3 were calculated using the IGMH analysis.

Table S9: Results of the IBSIW index from the IGMH analysis between the two BINOL substituents for the IDPi anion derived from **TS1** and **TS1′** (units: a.u./Å^2). The value in each entry represents the sum of the IBSIW index values between two pairs.

	IBSIW between 1 and 4	IBSIW between 2 and 3	Sum of IBSIW
**TS1**	10.68733	9.722875	20.410205
**TS1′**	8.0406	8.081191	16.121791

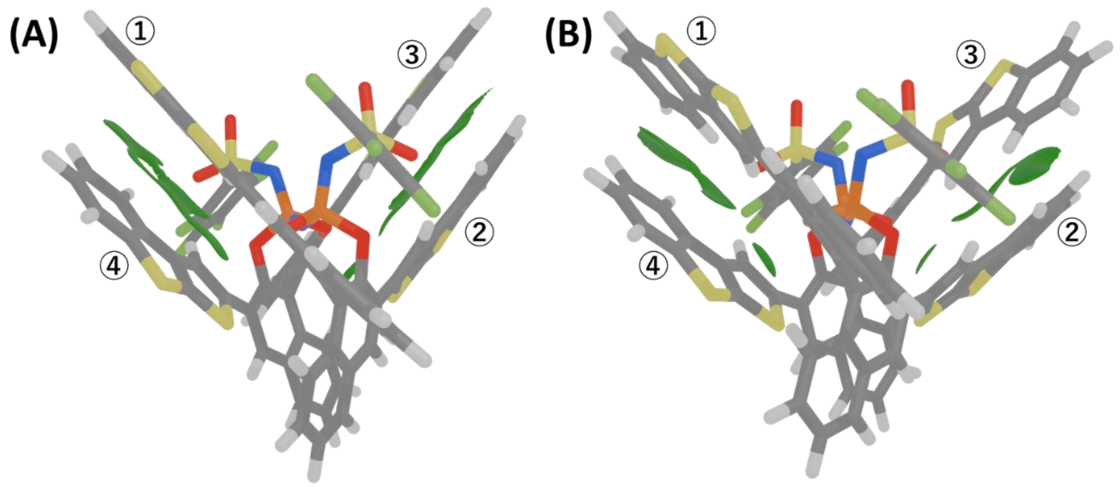

Figure S5: Visualization of noncovalent interactions between two adjacent BINOL substituents. (A) IDPi anion structure derived from **TS1** and (B) IDPi anion structure derived from **TS1′**. The color scale range from -0.05 to 0.05 au.

Comparison of the two oxocarbenium ion structures taken from the stereodetermining step shows that the oxocarbenium ion from **TS1** adopts a twist-boat conformation, whereas that from **TS1′** adopts a half-chair conformation (**Figure S6**). This conformational difference may contribute to the observed energy difference, although its contribution to the stereoselectivity is rather limited (**Table S8**).

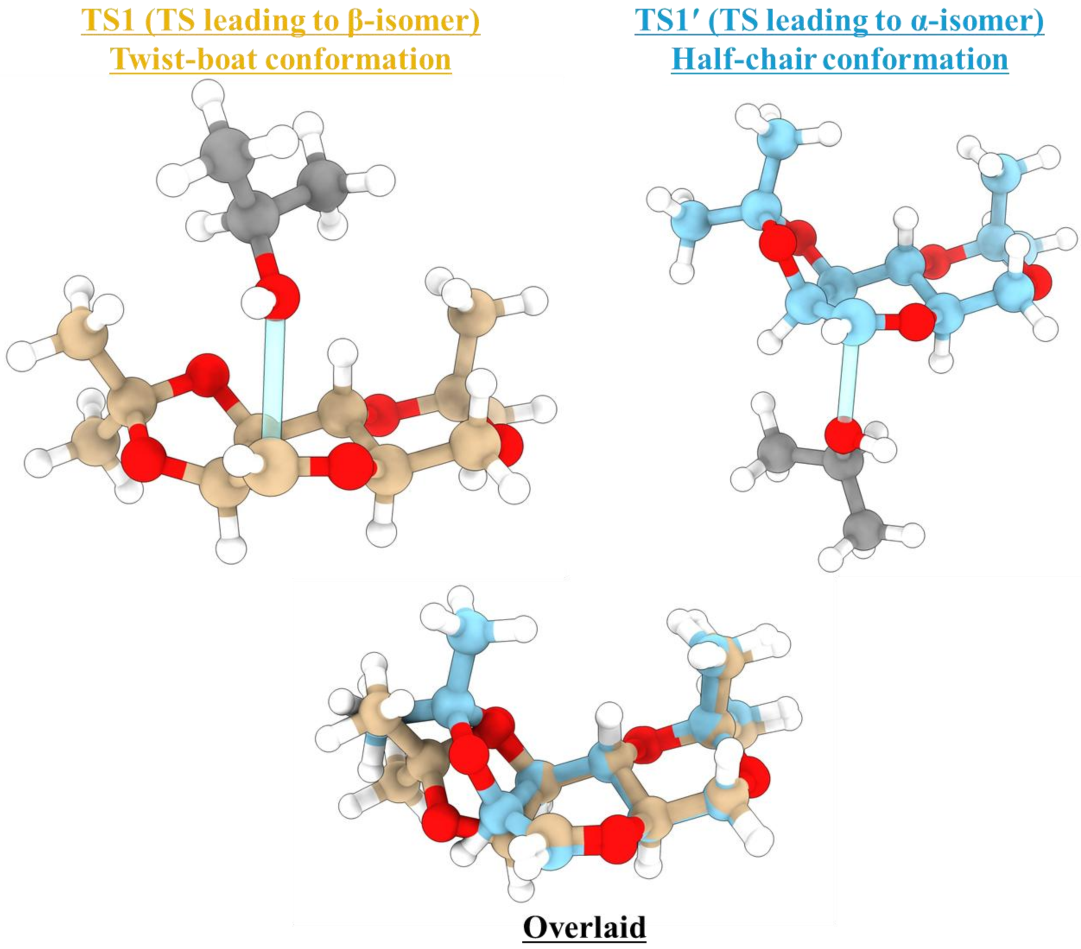

Figure S6: Conformations of the oxocarbenium ion and isopropanol from **TS1** and **TS1′**. The structure in brown corresponds to the oxocarbenium ion from **TS1**, which adopts a twist-boat conformation. In contrast, the structure in blue corresponds to the oxocarbenium ion from **TS1′**, which adopts a half-chair conformation.

Associative S_N2-like pathway

The S_N2-like pathway was calculated, in which *i*-PrOH attacks the β-face of the oxocarbenium ion-trichloroacetimidate complex to yield the β-isomer (**Figure S7**). We observed that higher energy is required to proceed via S_N2-like compared to that of the S_N1-like pathway (7.4 kcal/mol vs 23.4 kcal/mol).

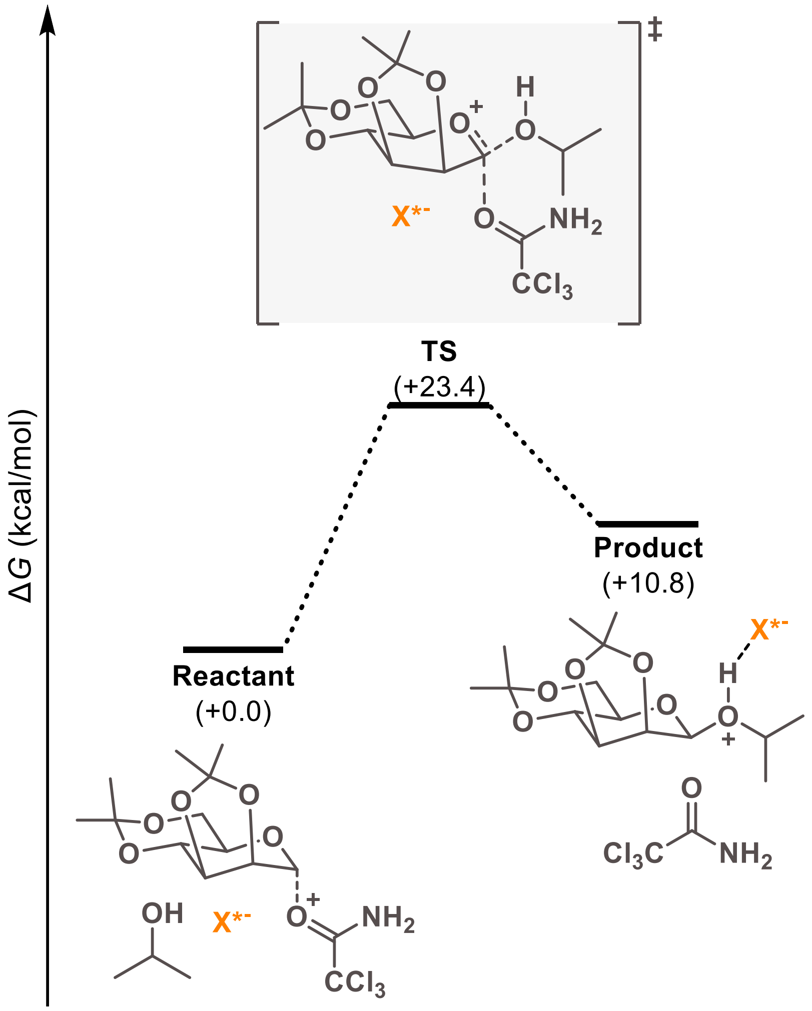

Figure S7: Energy diagram of SN2-like pathway. The transition state structure was taken from the highest point along the calculated SN2-like pathway.

We have additionally calculated an associative SN2-like pathway involving the covalent adduct between the substrate and the sulfur atom of the thiophene substituents of IDPi catalyst. The preliminary calculated pathway was significantly higher than that of SN1-like pathway depicted in **Figure S8**, indicating that this pathway is unlikely to occur during the *O*-glycosidic bond formation (21.9 kcal/mol vs 2.1 kcal/mol). The energies of this pathway are electronic energies, relative to the electronic energy of the intermediate **a**.

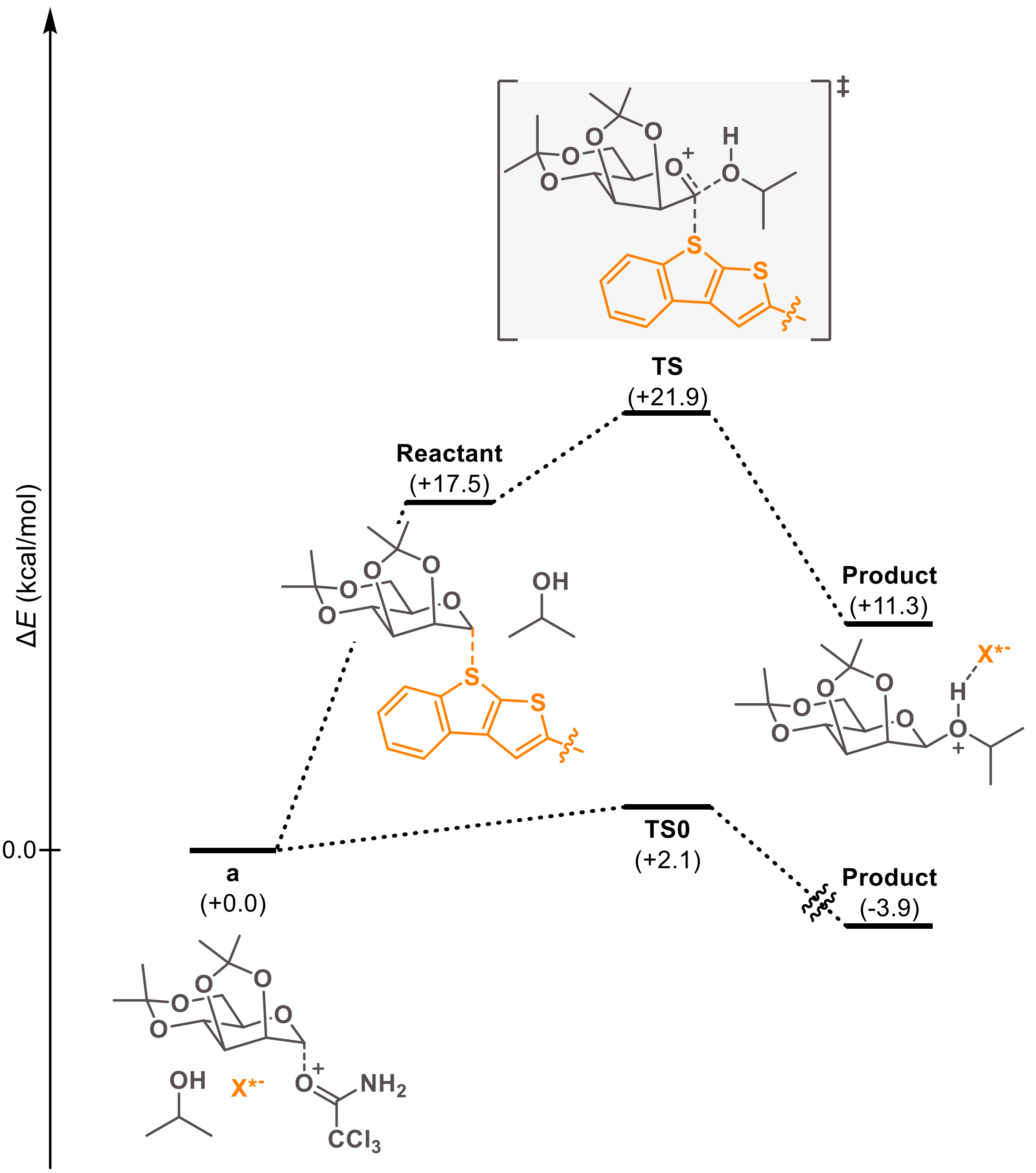

Figure S8: SN2-like pathway involving the sulfur atoms of the thiophene substituents of IDPi catalyst. The energies are computed relative to the electronic energy of intermediate **a**.

Comparing energies between the ionic intermediate and the covalent donor

The covalently bonded intermediate between the oxocarbenium ion and the IDPi anion was also investigated (**Figure S9**). Our calculations indicate that the ion pair is energetically favored, being lower in energy by 2.0 kcal/mol than the covalent intermediate (Free energies, calculated at CPCM(chloroform)-ωB97M-V/(ma)-def2-TZVPP//r^{2}SCAN-3c level of theory).

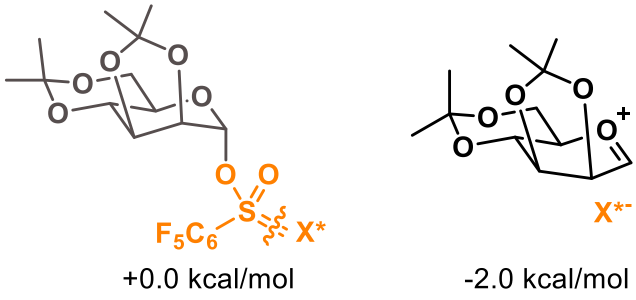

Figure S9: Comparing free energy between covalent donor and ionic intermediate.

Comparing the C-O distances for two pathways

Two pathways, namely a stepwise pathway and an asynchronous concerted pathway, were investigated in this study. To further analyze the differences between these pathways, the C-O distances between the oxocarbenium ion and the sulfonyl oxygen of the catalyst, as well as between the oxocarbenium ion and isopropanol, were calculated. The results are summarized in **Figure S10**. It is worth noting that, upon analysis of **TS1** (see **Figure S10**), the C-O distances between the oxocarbenium ion and the sulfonyl oxygen of the catalyst, as well as between the oxocarbenium ion and isopropanol, are longer (3.32 Å and 2.71 Å, respectively) than those in the TS structure reported for β-glucoside in a previous study.[29] In that study, the corresponding C-O distances were 2.38 Å between the oxocarbenium ion and trifluoromethanesulfonate, and 2.51 Å between the oxocarbenium ion and isopropanol. We speculate that the difference in C-O distances arises from the nature of counteranions; unlike triflate, the IDPi anion provides well-organized microenvironment, which likely contributes to stabilization of the corresponding oxocarbenium ion.

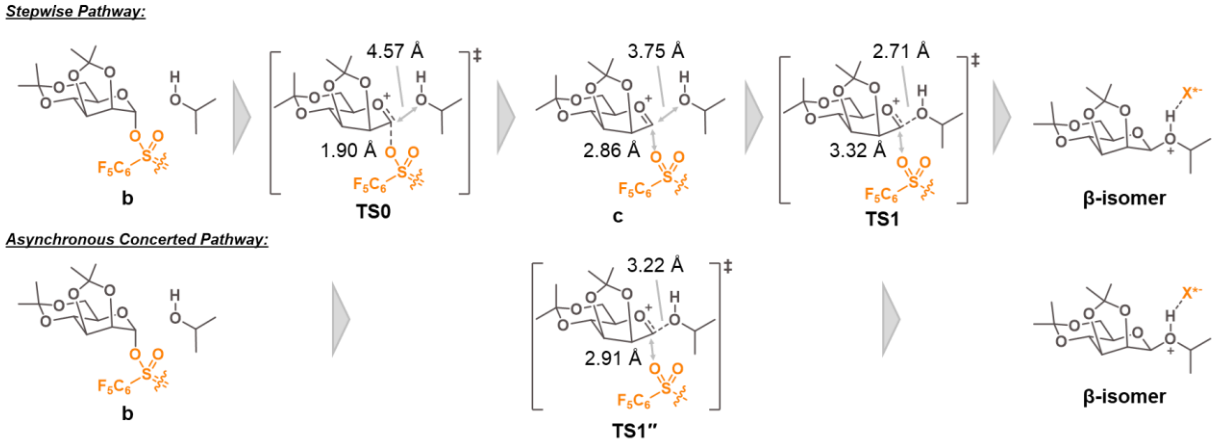

Figure S10: Comparing C-O bond length for stepwise and asynchronous concerted pathway.

Calculating KIE

The deuterium KIE was calculated for **TS1** using PyQuiver.[30] The ground-state structure was obtained from a structure connected to **TS1** via the IRC pathway.

Frequency calculations were performed at 213.15 K using the ORCA 5.0.3 program. The calculated deuterium KIE value was 1.13, which is in good agreement with the experimental value of 1.20. It should be noted that a previous studies reported KIEs for S_N2-like α- and β-gluco- and mannosylation reactions, yielding similar values (^2H KIE of 1.20 for the β-glucoside) to our computed result.[29,31] Since many factors influence the calculated KIE values, it is difficult to clearly distinguish whether this reaction proceeds via an S_N1-like or S_N2-like pathway based solely on computational results.

Summary of all EQs and TSs

Structures	Electronic Energy (Hartree)	Thermal Corrections (Hartree)	Gibbs Free Energy (kcal/mol)	Imaginary Frequency
**a**	-12586.9874	1.47565446	-7897408.617	
**b**	-10998.95482	1.43242921	-6900945.303	
**b′**	-10998.95229	1.43203156	-6900943.961	
**TS0**	-10998.94589	1.43138183	-6900940.355	-66.72667590
**TS0′**	-10998.9426	1.43198828	-6900937.908	-84.48083958
**c**	-10998.955413617581	1.42982327	-6900947.308	
**TS1**	-10998.944511009009	1.43052053	-6900940.029	-29.52036942
**TS1′**	-10998.942975537630	1.43121656	-6900938.629	-71.25045078
**β-isomer**	-10998.964460424302	1.43361650	-6900950.605	
**α-isomer**	-10998.964862250270	1.43293552	-6900951.284	
**TS1″**	-10998.93711	1.42911154	-6900936.27	-28.59203845
**Trichloroace timidate**	-1588.005314	0.01993744	-996460.8239	
**Isopropanol**	-194.3426774	0.08667644	-121895.6406	

Cartesian coordinates of computed structures

a:

O	5.020294	1.581876	9.867528
O	3.284229	0.978779	11.614099
O	5.440208	-1.907438	13.615407
O	6.967637	-2.406078	11.637252
S	3.542942	-4.058354	12.373622
S	3.909999	-0.518824	7.866412
O	2.948553	-3.534716	13.610799
O	2.578471	-4.701862	11.432287
O	5.312191	-0.965487	7.807592
O	2.911775	-1.287465	7.101192
N	4.370294	-3.017946	11.501388
P	5.449840	-1.939551	11.995744
N	3.364589	-0.351776	9.375634
H	2.118910	-3.755916	9.978523
N	5.346246	-0.513263	11.353749
P	4.285041	0.295436	10.523846
C	4.669285	-5.406932	12.845060
C	5.211357	-5.491086	14.131025
C	4.972832	-6.423377	11.933958
C	5.738150	-7.517748	12.310491
C	3.874486	1.183050	7.181911
C	2.715274	1.947736	7.309610
C	4.988915	1.795322	6.609404
C	4.952717	3.126620	6.206740
F	6.143033	1.152860	6.425132
F	6.034224	3.694852	5.669858
F	1.607515	1.431838	7.845493
F	4.539496	-6.392471	10.676598
F	5.995638	-8.494333	11.440151
F	5.010204	-4.546324	15.045206
C	2.658605	3.270382	6.896913
C	3.788971	3.867278	6.354965
C	5.988893	-6.577568	14.510395
C	6.248687	-7.591892	13.599158
F	7.009971	-8.625179	13.953414
F	6.492690	-6.646355	15.741682
F	3.750155	5.139146	5.963886
F	1.527666	3.967167	7.008801
C	2.476510	2.017487	11.159128
C	5.452999	2.587959	10.726246
C	6.448328	-1.152234	14.216118
C	7.616180	-3.365606	12.406879
C	1.080148	1.767810	11.065004
C	7.978718	-4.603947	11.791757
C	6.832083	2.636552	11.069367
C	6.103430	0.108427	14.772674
C	1.983314	6.443076	9.282731
C	2.800684	5.476242	9.815995
C	2.259454	4.267771	10.316137
C	0.850277	4.053113	10.218239
C	0.033714	5.084049	9.686976
C	0.585557	6.253957	9.233073
H	2.416765	7.358133	8.890502
H	3.874763	5.628552	9.853492

C	3.067354	3.238391	10.880480	C	9.637590	-6.234652	14.726746
C	0.299549	2.824666	10.621418	C	8.991296	-5.254830	13.932299
H	-1.039089	4.919253	9.626093	C	8.594280	-4.021985	14.528325
H	-0.775365	2.707150	10.535608	C	8.809532	-3.841209	15.914720
C	5.817031	6.435855	13.937091	C	9.423094	-4.817207	16.663018
C	6.724038	5.599343	13.336785	H	8.989516	-6.456836	12.171232
C	6.302028	4.599134	12.426300	H	9.940171	-7.168780	14.260008
C	4.912644	4.495437	12.100081	H	8.474129	-2.924702	16.388043
C	4.002334	5.360260	12.752501	H	9.568853	-4.665159	17.728216
C	4.443419	6.303394	13.651111	C	8.688288	-5.496423	12.581602
H	8.271376	3.766866	12.173705	C	7.971222	-3.037068	13.702188
H	7.783679	5.676467	13.567040	H	-0.048466	7.030641	8.816089
C	7.223995	3.674110	11.895922	H	6.152488	7.190029	14.642707
C	4.500300	3.461953	11.200502	H	11.626281	1.467751	16.046282
H	2.941666	5.267585	12.549833	H	10.342528	-6.778059	16.667043
H	3.724468	6.948808	14.147197	C	4.739657	0.622495	14.748387
C	11.143678	-0.418681	15.098528	C	3.528955	-0.021512	14.830787
C	10.148054	-1.250767	14.643398	C	2.827718	2.199153	14.685672
C	8.788099	-0.872283	14.737688	C	2.427162	0.876522	14.817810
C	8.473443	0.405645	15.293523	H	3.436437	-1.099378	14.898591
C	9.521136	1.225833	15.779142	C	0.459261	0.487138	11.402180
C	10.829757	0.824861	15.683667	C	-0.887262	0.200806	11.357647
H	12.181917	-0.722353	15.002811	C	-0.076019	-1.864909	12.038160
H	10.404690	-2.202330	14.192026	C	-1.204666	-1.131951	11.707080
C	7.720266	-1.678300	14.233540	H	-1.640237	0.928572	11.081909
C	7.136525	0.846312	15.323181	C	7.622468	-4.966458	10.424965
H	9.267930	2.191058	16.210477	C	6.723416	-4.425396	9.538489
H	6.919260	1.806059	15.785298	C	7.412098	-6.285016	8.317301
C	9.855367	-6.018053	16.063551	C	6.579224	-5.182339	8.349648

H	6.159135	-3.525461	9.724427
C	7.742806	1.569131	10.677442
C	7.738284	0.743296	9.581909
C	9.411834	-0.260377	10.852327
C	8.683044	-0.307883	9.673013
H	7.058696	0.856482	8.749217
S	8.955687	1.054460	11.867544
S	10.538004	-1.573253	11.053459
S	4.537436	2.376937	14.615662
S	1.522365	3.349154	14.608402
S	8.363300	-6.443361	9.742622
S	7.316199	-7.218738	6.851333
S	1.376786	-0.943675	11.934570
S	-0.398605	-3.501167	12.539630
C	0.361637	2.019095	14.771587
C	-1.022662	2.150238	14.781672
C	0.993980	0.759433	14.888641
C	-1.791081	1.000398	14.915837
H	-1.492025	3.125090	14.687529
C	0.195366	-0.380937	15.035916
C	-1.184603	-0.254869	15.046028
H	-2.874157	1.078796	14.922808
H	0.661605	-1.358552	15.124574
H	-1.803576	-1.142021	15.145270
C	-2.408372	-1.907788	11.830829
C	-2.128543	-3.222498	12.274158
C	-3.737775	-1.550561	11.580442
C	-3.136928	-4.162845	12.459604
C	-4.744898	-2.485851	11.764442

H	-3.973406	-0.545825	11.240639
C	-4.447825	-3.783026	12.199305
H	-2.909631	-5.168820	12.800812
H	-5.776669	-2.210386	11.568396
H	-5.248034	-4.503573	12.336680
C	10.007646	-2.249053	9.500006
C	10.476017	-3.425512	8.927410
C	9.013700	-1.459850	8.877361
C	9.935541	-3.822743	7.710368
H	11.233280	-4.027101	9.421599
C	8.481751	-1.883883	7.654498
C	8.945506	-3.058645	7.082576
H	10.277024	-4.745450	7.250692
H	7.695938	-1.306212	7.178444
H	8.521724	-3.399048	6.142027
C	6.054132	-6.123514	6.253143
C	5.768426	-5.084885	7.168203
C	5.384057	-6.233762	5.040565
C	4.778401	-4.151819	6.846208
C	4.401995	-5.293889	4.743869
H	5.618466	-7.031031	4.341399
C	4.102260	-4.260158	5.640339
H	4.571000	-3.331079	7.523864
H	3.872209	-5.360115	3.797978
H	3.348125	-3.519938	5.390404
C	2.070152	-6.960148	7.547817
C	1.013864	-8.086852	7.575454
C	0.283539	-8.157546	8.907466
C	-0.146348	-6.754399	9.289478

H	2.204027	-6.561696	6.529262
H	0.268783	-7.972297	6.776999
H	0.962934	-8.536963	9.685970
H	-0.687558	-6.280182	8.457011
O	3.240741	-7.612650	8.008085
O	1.781686	-9.247938	7.308930
O	-0.864226	-8.981444	8.757974
C	-1.029010	-6.781513	10.522326
H	-1.483822	-5.802316	10.701684
H	-0.423597	-7.061705	11.396979
O	1.053566	-6.008461	9.595350
C	3.164802	-8.998994	7.632927
C	3.981045	-9.268382	6.381925
H	3.826410	-10.302453	6.060625
H	3.663987	-8.598903	5.577574
H	5.043360	-9.108696	6.584864
C	3.612103	-9.820802	8.826199
H	3.007268	-9.580910	9.704571
H	3.519027	-10.886024	8.597339
H	4.658050	-9.594828	9.054147
O	-2.103115	-7.689047	10.285385
C	-1.693286	-9.015170	9.927912
C	-2.958802	-9.734831	9.510202
H	-2.717716	-10.751934	9.192068
H	-3.657066	-9.773964	10.349661
H	-3.424825	-9.199648	8.679489
C	-0.988066	-9.714372	11.089110
H	-1.672340	-9.778128	11.940403
H	-0.705743	-10.726068	10.783843
H	-0.085116	-9.190795	11.411902
C	1.854388	-5.790067	8.513146
H	2.824948	-5.409905	8.851968
O	1.200240	-4.693629	7.683146
C	-0.026898	-2.673926	7.536651
C	1.041478	-3.534058	8.239593
N	1.606035	-3.132146	9.326775
H	1.519184	-2.158174	9.617944
Cl	-0.109119	-1.028466	8.171482
Cl	0.227949	-2.673957	5.781879
Cl	-1.579463	-3.524853	7.951029
C	2.069534	-9.321976	13.762514
H	2.206770	-9.486920	14.835584
H	2.861698	-9.851047	13.223424
H	1.104943	-9.749330	13.466532
C	2.111199	-7.838962	13.443528
H	3.083733	-7.437113	13.776253
C	1.004092	-7.055194	14.135476
H	1.098097	-7.130679	15.224428
H	1.053165	-5.993417	13.868762
H	0.021901	-7.448560	13.845905
O	2.016724	-7.721128	12.014840
H	2.042464	-6.779896	11.777953

b:

O	5.453247	1.737253	10.101277
O	3.799521	1.081241	11.909127
O	6.208635	-1.473641	14.094049
O	7.875831	-1.828094	12.195183
S	4.617598	-3.799915	12.805635

S	4.461308	-0.511813	8.276130	F	7.458863	-5.938894	16.505902
O	3.814589	-3.337966	13.936395	F	1.273550	3.988925	6.022881
O	3.744832	-4.620035	11.791846	F	0.040373	1.839053	7.161590
O	5.879108	-0.292549	7.942098	C	2.848557	1.914484	11.319283
O	3.798718	-1.688681	7.691554	C	5.700340	2.883882	10.851032
N	5.422642	-2.772050	11.961804	C	7.113812	-0.587352	14.699554
P	6.313041	-1.518387	12.479169	C	8.563392	-2.722690	13.015820
N	4.149507	-0.514642	9.852843	C	1.511791	1.438169	11.240661
N	6.043829	-0.134961	11.812157	C	9.044990	-3.941381	12.449058
P	4.896803	0.440082	10.887757	C	7.040893	3.168773	11.223633
C	5.730059	-5.083867	13.421885	C	6.636273	0.667062	15.160924
C	6.174029	-5.036782	14.749364	C	1.764393	5.907910	8.822692
C	6.219767	-6.099696	12.594581	C	2.686870	5.180063	9.535001
C	7.066900	-7.079757	13.093194	C	2.319280	3.967899	10.165995
C	3.536969	0.931812	7.645945	C	0.978374	3.494427	10.021676
C	2.142066	0.894867	7.680275	C	0.048009	4.280409	9.294487
C	4.145915	2.062022	7.100108	C	0.429651	5.462558	8.713697
C	3.383323	3.098031	6.569247	H	2.066748	6.827690	8.331226
F	5.468680	2.208466	7.052864	H	3.714062	5.523063	9.609163
F	3.980138	4.170838	6.044632	C	3.248170	3.167676	10.890805
F	1.498763	-0.132794	8.234850	C	0.609805	2.254536	10.573429
F	5.897094	-6.167418	11.306777	H	-0.970659	3.916626	9.189150
F	7.491223	-8.067473	12.309061	H	-0.414174	1.923106	10.435154
F	5.795523	-4.078202	15.586958	C	5.372725	7.045892	13.643759
C	1.374097	1.913797	7.138833	C	6.416435	6.293209	13.166454
C	1.998412	3.015942	6.569514	C	6.182912	5.152125	12.360616
C	7.044255	-5.999118	15.243908	C	4.837354	4.812633	12.009368
C	7.486476	-7.021572	14.415976	C	3.783846	5.595572	12.537296
F	8.330009	-7.935492	14.883272	C	4.045931	6.683469	13.336907

H	8.261783	4.589568	12.250135
H	7.442639	6.550864	13.415914
C	7.249629	4.322715	11.955118
C	4.615031	3.647270	11.211982
H	2.757962	5.326109	12.312731
H	3.221563	7.265765	13.737872
C	11.687968	0.622784	15.685856
C	10.789158	-0.319924	15.246158
C	9.398889	-0.058521	15.268751
C	8.949581	1.216756	15.731172
C	9.900988	2.155299	16.200009
C	11.242085	1.866167	16.178816
H	12.751936	0.409076	15.647652
H	11.144424	-1.269276	14.861455
C	8.427919	-0.986265	14.781775
C	7.577623	1.532653	15.688811
H	9.547720	3.118562	16.558747
H	7.253007	2.494834	16.076744
C	10.786117	-5.101130	16.858223
C	10.644551	-5.375491	15.521736
C	9.972367	-4.467247	14.665250
C	9.470157	-3.245089	15.202844
C	9.615034	-3.001118	16.588586
C	10.255329	-3.910280	17.396308
H	10.188723	-5.692479	12.936619
H	11.030958	-6.300975	15.102249
H	9.205088	-2.090311	17.012101
H	10.347982	-3.713029	18.459983
C	9.768812	-4.760654	13.306383
C	8.820458	-2.335555	14.316624
H	-0.288453	6.047726	8.147206
H	5.565510	7.912503	14.269052
H	11.963952	2.597941	16.528828
H	11.293743	-5.807266	17.508429
C	5.231581	1.046381	15.074424
C	4.096748	0.308158	15.296080
C	3.169583	2.399464	14.845878
C	2.909755	1.081690	15.195682
H	4.120798	-0.748632	15.535933
C	1.052204	0.202361	11.866424
C	-0.262802	-0.118425	12.123627
C	0.784873	-2.001716	12.985522
C	-0.428565	-1.373227	12.749915
H	-1.086968	0.551475	11.911326
C	8.812726	-4.345188	11.068636
C	8.146917	-3.745283	10.023454
C	8.825567	-5.716954	8.984429
C	8.148502	-4.518364	8.834828
H	7.684076	-2.771995	10.086515
C	8.101981	2.206889	10.951202
C	8.287805	1.370996	9.880612
C	9.944894	0.589753	11.323050
C	9.342460	0.444443	10.081458
H	7.663120	1.394713	8.995763
S	9.246920	1.856208	12.260109
S	11.196586	-0.574995	11.656201
S	4.852758	2.730904	14.685972
S	1.749852	3.393688	14.669750

S	9.480992	-5.922246	10.560740	C	9.570695	-1.125258	8.061064
S	8.868169	-6.713749	7.561299	C	10.244929	-2.221494	7.545752
S	2.130859	-1.074770	12.457404	H	11.743366	-3.736388	7.874357
S	0.651875	-3.520305	13.830998	H	8.782029	-0.641034	7.491656
C	0.740604	2.007326	15.122457	H	9.981466	-2.596638	6.561474
C	-0.644642	2.018517	15.241548	C	7.929209	-5.482464	6.694028
C	1.502628	0.845134	15.383049	C	7.626336	-4.364702	7.503567
C	-1.279025	0.849867	15.645726	C	7.485149	-5.579876	5.379822
H	-1.216224	2.918325	15.033918	C	6.886176	-3.313631	6.955248
C	0.838442	-0.315326	15.795287	C	6.741587	-4.526410	4.861323
C	-0.540808	-0.304845	15.928807	H	7.706728	-6.455023	4.776567
H	-2.360117	0.835090	15.746810	C	6.455028	-3.399184	5.640874
H	1.405487	-1.219008	15.999790	H	6.650796	-2.441547	7.554903
H	-1.057559	-1.208476	16.238576	H	6.373538	-4.582601	3.841654
C	-1.539126	-2.127217	13.260449	H	5.877968	-2.585465	5.212618
C	-1.109119	-3.328423	13.871412	C	1.807233	-4.502637	10.306423
C	-2.907841	-1.838266	13.232618	C	1.395791	-5.457367	9.132375
C	-2.009606	-4.226329	14.433327	C	2.528581	-5.812152	8.190583
C	-3.808508	-2.730480	13.794861	C	3.828675	-5.863345	8.978840
H	-3.256135	-0.918127	12.771447	H	1.570471	-4.980897	11.266273
C	-3.364056	-3.917164	14.390211	H	1.008675	-6.397712	9.554484
H	-1.665584	-5.145771	14.897869	H	2.621372	-5.038253	7.411097
H	-4.871077	-2.507996	13.773087	H	3.739386	-6.541052	9.841484
H	-4.082575	-4.605388	14.824540	O	1.005928	-3.349800	10.112124
C	10.913502	-1.314152	10.068101	O	0.367313	-4.741457	8.457525
C	11.582503	-2.421365	9.558815	O	2.265688	-7.099110	7.637270
C	9.899510	-0.653056	9.337137	C	4.938915	-6.335891	8.063694
C	11.238834	-2.868733	8.288876	H	5.881167	-6.470342	8.602268
H	12.350058	-2.927121	10.137224	H	5.094632	-5.601502	7.259077

O	4.124226	-4.522080	9.431884
C	3.275176	-4.040027	10.348456
H	3.377420	-2.954502	10.422681
C	-0.151418	-3.783503	9.382516
C	-1.176589	-4.423385	10.314102
H	-1.549839	-3.684136	11.028090
H	-2.014578	-4.806541	9.724936
H	-0.746223	-5.256622	10.877838
C	-0.711983	-2.602140	8.628742
H	-1.564379	-2.920851	8.022902
H	-1.043821	-1.842051	9.341871
H	0.055042	-2.172629	7.983441
O	4.564513	-7.618412	7.567927
C	3.334472	-7.633816	6.836369
C	2.996077	-9.099387	6.646363
H	2.032565	-9.193196	6.139808
H	3.769229	-9.581670	6.043132
H	2.941506	-9.591079	7.620595
C	3.461961	-6.898971	5.505405
H	4.299261	-7.324810	4.944167
H	2.542685	-7.040285	4.929647
H	3.622796	-5.823630	5.609608
C	1.853831	-2.964122	5.577897
H	1.561279	-2.852719	6.633000
C	1.788068	-1.601480	4.896967
H	0.786435	-1.164831	4.994261
H	2.507734	-0.913159	5.352630
H	2.026767	-1.695545	3.831536
C	0.942511	-3.984255	4.920694
H	1.263575	-4.166219	3.888508
H	0.963681	-4.931030	5.468377
H	-0.090267	-3.621266	4.906476
O	3.189662	-3.492229	5.525212
H	3.716511	-2.963671	6.143029

b’:

O	5.381591	1.988231	10.224236
O	3.660673	1.358663	11.966329
O	5.772854	-1.624023	13.870337
O	7.480258	-1.866684	11.973043
S	4.020675	-3.775196	12.482119
S	3.659902	0.180012	8.204741
O	3.078445	-3.028416	13.334365
O	3.450304	-4.743510	11.529498
O	4.831753	0.002736	7.353763
O	2.512236	-0.816802	7.761571
N	5.055229	-2.851177	11.674653
P	5.894012	-1.630603	12.248907
N	3.770995	0.022064	9.735228
N	5.701344	-0.177283	11.654131
P	4.671756	0.695368	10.887478
C	5.025111	-4.790545	13.624102
C	5.002462	-4.628119	15.010257
C	5.775304	-5.847821	13.104492
C	6.427070	-6.749452	13.932804
C	2.959577	1.796882	7.785744
C	1.588355	2.052628	7.859253
C	3.804248	2.839124	7.385649
C	3.287436	4.076748	7.025843

F	5.124826	2.694205	7.331261	H	4.077926	6.024508	10.215223
F	4.103672	5.058347	6.645348	C	3.355112	3.602630	11.228488
F	0.731727	1.133242	8.286352	C	0.603254	3.092459	10.973088
F	5.891638	-6.028097	11.788649	H	-0.817449	5.175765	10.061385
F	7.134091	-7.755759	13.411848	H	-0.469504	2.934247	10.922960
F	4.351711	-3.628656	15.604463	C	5.955675	6.829641	14.375030
C	1.066249	3.280550	7.476275	C	6.895283	6.015119	13.795843
C	1.915304	4.292265	7.050607	C	6.517484	5.017961	12.863875
C	5.659062	-5.523874	15.847884	C	5.142790	4.890869	12.490514
C	6.353285	-6.597846	15.311092	C	4.197546	5.731875	13.125419
F	6.949737	-7.475397	16.114384	C	4.594618	6.673429	14.045517
F	5.614453	-5.360410	17.172308	H	8.505915	4.206684	12.669156
F	1.421782	5.463479	6.669046	H	7.945724	6.106802	14.059640
F	-0.249617	3.487632	7.524280	C	7.469241	4.111826	12.355802
C	2.811088	2.359605	11.492202	C	4.775794	3.861877	11.564291
C	5.759462	3.013427	11.102730	H	3.145362	5.623286	12.892819
C	6.752160	-0.938727	14.585635	H	3.848453	7.299479	14.525880
C	8.081775	-2.931550	12.635224	C	11.394198	-0.423937	15.841754
C	1.431524	2.043551	11.350146	C	10.410898	-1.185310	15.255499
C	8.530765	-4.055212	11.880369	C	9.059356	-0.765722	15.280209
C	7.119768	3.087364	11.498013	C	8.743476	0.483589	15.898291
C	6.407635	0.295140	15.200033	C	9.777457	1.230986	16.513302
C	2.153863	6.793946	9.689641	C	11.075876	0.787917	16.488009
C	3.007905	5.842054	10.192682	H	12.426740	-0.757745	15.801869
C	2.510318	4.610474	10.680322	H	10.670955	-2.112707	14.757120
C	1.106507	4.352232	10.601259	C	8.007435	-1.500349	14.651768
C	0.250694	5.370894	10.107777	C	7.420332	0.965393	15.863315
C	0.761301	6.564177	9.664292	H	9.523514	2.173699	16.991305
H	2.552609	7.727757	9.305055	H	7.195110	1.899543	16.371755

C	9.824306	-6.134417	16.099470	C	8.189140	-3.650211	8.154811
C	9.747972	-6.162572	14.729918	H	7.408482	-2.350728	9.750171
C	9.222243	-5.059228	14.011749	C	8.085309	2.059885	11.117701
C	8.799235	-3.902154	14.732719	C	8.367782	1.493085	9.903698
C	8.872917	-3.911168	16.144952	C	9.787482	0.271310	11.292161
C	9.368762	-5.005766	16.812633	C	9.361502	0.479645	9.987549
H	9.448001	-5.983422	12.101408	H	7.877232	1.799770	8.986589
H	10.072313	-7.039638	14.175953	S	9.029923	1.326137	12.422117
H	8.519249	-3.047105	16.698665	S	10.944348	-1.016141	11.479409
H	9.403463	-5.004924	17.897840	S	4.909606	2.613622	15.001752
C	9.098280	-5.089812	12.611367	S	1.917239	3.660248	14.924236
C	8.282879	-2.799423	13.994026	S	9.321186	-5.462312	9.621909
H	0.096875	7.327974	9.271530	S	9.308562	-5.327387	6.457939
H	6.254738	7.582958	15.097594	S	1.752247	-0.620088	12.321314
H	11.862350	1.374484	16.953392	S	0.250266	-3.403045	12.248978
H	10.214508	-6.991372	16.640189	C	0.721378	2.356572	15.036960
C	5.062024	0.852146	15.123627	C	-0.658818	2.520103	14.981007
C	3.834452	0.240255	15.170336	C	1.318756	1.083425	15.173644
C	3.192984	2.479380	15.032532	C	-1.457331	1.387412	15.068871
C	2.756034	1.166428	15.143921	H	-1.100760	3.505522	14.867087
H	3.715720	-0.836112	15.223810	C	0.488928	-0.039362	15.279153
C	0.885931	0.702744	11.518372	C	-0.886156	0.117713	15.221817
C	-0.327801	0.269537	11.025466	H	-2.537113	1.490655	15.021353
C	0.452805	-1.696523	11.988456	H	0.928244	-1.027670	15.381543
C	-0.584869	-1.094496	11.283638	H	-1.528215	-0.755411	15.286828
H	-0.987801	0.897250	10.439586	C	-1.603661	-2.039069	10.910731
C	8.479692	-4.109179	10.426002	C	-1.280340	-3.341345	11.358698
C	7.944218	-3.251726	9.492701	C	-2.771087	-1.851246	10.163173
C	8.919822	-4.822924	8.076803	C	-2.086334	-4.436916	11.067243

C	-3.582649	-2.938932	9.880955
H	-3.027254	-0.859971	9.799921
C	-3.241440	-4.222672	10.326181
H	-1.799512	-5.433071	11.387536
H	-4.488846	-2.797624	9.299152
H	-3.882142	-5.065047	10.083383
C	10.904761	-1.286711	9.727647
C	11.639448	-2.244922	9.040669
C	10.029874	-0.395939	9.061007
C	11.518832	-2.298102	7.656663
H	12.291525	-2.934636	9.568027
C	9.941250	-0.461462	7.667397
C	10.685719	-1.404563	6.975933
H	12.078244	-3.044248	7.100782
H	9.282011	0.216536	7.133150
H	10.600095	-1.466362	5.895048
C	8.471074	-3.897705	5.827557
C	7.910235	-3.106618	6.855336
C	8.369874	-3.536817	4.488412
C	7.230767	-1.935485	6.511656
C	7.689654	-2.365780	4.172942
H	8.808797	-4.151028	3.707671
C	7.120900	-1.573609	5.178254
H	6.795890	-1.315060	7.287641
H	7.597405	-2.068302	3.132749
H	6.585250	-0.666419	4.914862
C	0.728980	-2.487800	8.225364
C	1.036822	-3.420151	7.033003
C	2.511752	-3.743483	6.962561

C	3.060002	-3.919383	8.374248
H	0.289296	-3.061208	9.059027
H	0.470149	-4.359078	7.125211
H	3.024107	-2.912348	6.453687
H	2.509804	-4.721103	8.884157
O	-0.179693	-1.530635	7.731979
O	0.611553	-2.657102	5.910020
O	2.702380	-4.971702	6.272314
C	4.534979	-4.274197	8.282702
H	4.944038	-4.516882	9.266845
H	5.094767	-3.417408	7.870761
O	2.935713	-2.713132	9.174015
C	1.963564	-1.838997	8.836938
H	1.726774	-1.201251	9.687866
C	-0.468696	-1.841478	6.350027
C	-1.796239	-2.584441	6.274051
H	-2.600134	-1.949218	6.656656
H	-2.014726	-2.857461	5.237373
H	-1.768464	-3.492883	6.883258
C	-0.453553	-0.556794	5.550203
H	-0.651224	-0.771521	4.496402
H	-1.223928	0.123431	5.923814
H	0.527214	-0.084066	5.644175
O	4.673818	-5.444896	7.478181
C	4.083265	-5.366641	6.180557
C	4.063035	-6.787223	5.652562
H	3.620401	-6.806409	4.653480
H	5.083837	-7.173979	5.604453
H	3.472217	-7.417388	6.321431

C	4.860888	-4.433519	5.257905
H	5.866160	-4.833531	5.105388
H	4.351151	-4.367409	4.292327
H	4.971492	-3.424560	5.658918
C	1.364073	-7.342963	10.425582
H	0.495998	-7.885805	10.026005
C	2.547348	-7.529404	9.483550
H	2.310388	-7.139580	8.488672
H	3.427091	-6.992153	9.857476
H	2.810197	-8.589426	9.389757
C	1.666478	-7.845351	11.833211
H	2.522894	-7.300016	12.248334
H	0.805451	-7.690544	12.491209
H	1.912776	-8.912951	11.823859
O	0.944285	-5.969239	10.457339
H	1.645630	-5.466213	10.902787

TS0:

O	5.258025	1.637224	10.266311
O	3.382689	1.225652	11.957446
O	5.814734	-1.490128	14.359934
O	7.503281	-1.643099	12.458606
S	4.479113	-3.993477	13.019526
S	4.082919	-0.533539	8.435439
O	3.696750	-3.629516	14.202373
O	3.687125	-4.818740	12.005505
O	5.523944	-0.596041	8.141025
O	3.224501	-1.573197	7.832425
N	5.157183	-2.837542	12.200688
P	5.910760	-1.521180	12.744801
N	3.733490	-0.480478	10.001215
N	5.482612	-0.163861	12.115480
P	4.492880	0.446554	11.058567
C	5.777139	-5.126561	13.585998
C	6.248523	-5.068599	14.902821
C	6.361068	-6.052516	12.718363
C	7.340664	-6.932087	13.156501
C	3.486272	1.059946	7.766617
C	2.115964	1.327562	7.771030
C	4.338722	2.049232	7.272518
C	3.836412	3.250888	6.784144
F	5.662562	1.905790	7.241225
F	4.668097	4.183660	6.316066
F	1.247772	0.445772	8.265991
F	6.000696	-6.128853	11.437928
F	7.859617	-7.832812	12.324097
F	5.781101	-4.191803	15.784499
C	1.604146	2.515974	7.271838
C	2.468544	3.480873	6.771471
C	7.240497	-5.935316	15.344006
C	7.786582	-6.864942	14.470071
F	8.750070	-7.681542	14.886178
F	7.674009	-5.870944	16.600389
F	1.988052	4.617454	6.275745
F	0.287560	2.738095	7.271096
C	2.687070	2.271852	11.358363
C	5.725102	2.663079	11.091770
C	6.570699	-0.454154	14.931159
C	8.311008	-2.396945	13.305982

C	1.291799	2.115614	11.102315	C	10.162507	0.372158	15.472860
C	8.958238	-3.554584	12.779152	C	8.749786	0.437279	15.455218
C	7.095250	2.670328	11.467451	C	8.116263	1.671410	15.798136
C	5.906988	0.739906	15.319316	C	8.918773	2.776621	16.175591
C	2.773192	6.587341	9.167725	C	10.287475	2.677794	16.193965
C	3.432345	5.620446	9.888675	H	11.996077	1.399763	15.830426
C	2.741139	4.482502	10.367686	H	10.655457	-0.548996	15.180618
C	1.353420	4.342582	10.065799	C	7.927714	-0.647665	15.022954
C	0.699356	5.368353	9.337850	C	6.712170	1.779103	15.750017
C	1.392847	6.468443	8.901608	H	8.425525	3.708245	16.442102
H	3.321265	7.446278	8.792379	H	6.255648	2.716776	16.058033
H	4.495709	5.715037	10.083913	C	10.778745	-4.359614	17.234293
C	3.384239	3.437186	11.090231	C	10.685396	-4.691029	15.906600
C	0.670352	3.180669	10.464518	C	9.917330	-3.898532	15.016706
H	-0.357960	5.255604	9.112932	C	9.263917	-2.731321	15.511295
H	-0.385618	3.120708	10.216931	C	9.364798	-2.426851	16.889101
C	6.087968	6.459309	14.357698	C	10.102638	-3.225165	17.729882
C	6.984921	5.569853	13.821137	H	10.290546	-5.145523	13.331466
C	6.570689	4.604531	12.869933	H	11.185678	-5.575544	15.520229
C	5.202077	4.581384	12.455696	H	8.842517	-1.559483	17.279113
C	4.300936	5.503379	13.036958	H	10.160403	-2.984254	18.787075
C	4.733812	6.419438	13.965799	C	9.763515	-4.256202	13.666783
H	8.520874	3.723236	12.666460	C	8.508698	-1.940738	14.596109
H	8.029685	5.585606	14.121929	H	0.886086	7.240497	8.330498
C	7.482710	3.665216	12.347955	H	6.416324	7.192096	15.088927
C	4.794927	3.575674	11.528387	H	10.893115	3.532796	16.479692
H	3.254437	5.473060	12.751921	H	11.360922	-4.978249	17.910628
H	4.025549	7.114542	14.406705	C	4.460276	0.868939	15.246053
C	10.912225	1.466320	15.832738	C	3.480443	-0.078439	15.418939

C	2.182908	1.820157	15.048153	C	-1.903052	-0.566736	15.673156
C	2.171790	0.461216	15.329929	H	-2.220587	1.499755	15.138481
H	3.697133	-1.121411	15.616457	C	0.392504	-1.322439	15.827505
C	0.518912	0.942715	11.500610	C	-0.966903	-1.574500	15.926484
C	0.879370	-0.284259	12.007682	H	-2.964131	-0.786159	15.740530
C	-1.433713	-0.547667	11.934444	H	1.114664	-2.114940	16.002383
C	-0.225931	-1.142247	12.254074	H	-1.308687	-2.573886	16.178856
H	1.902464	-0.568877	12.201006	C	-0.411982	-2.469149	12.775347
C	8.814659	-4.008978	11.401060	C	-1.778330	-2.819373	12.839849
C	8.026940	-3.586719	10.352882	C	0.537957	-3.392796	13.214697
C	9.111913	-5.382986	9.342573	C	-2.200047	-4.051517	13.328075
C	8.184497	-4.368477	9.177802	C	0.127280	-4.622534	13.702258
H	7.356114	-2.742569	10.409439	H	1.590830	-3.136550	13.194720
C	8.022384	1.649477	11.001693	C	-1.233608	-4.952421	13.759197
C	8.044249	0.926589	9.834107	H	-3.255239	-4.304730	13.372381
C	9.900471	0.032889	10.922599	H	0.872715	-5.330327	14.053848
C	9.124547	0.009042	9.773274	H	-1.541645	-5.918612	14.147516
H	7.308071	1.040987	9.049316	C	10.755512	-1.629448	9.300772
S	9.354054	1.186829	12.076976	C	11.404579	-2.601546	8.550112
S	11.221504	-1.099133	10.927486	C	9.619685	-0.941139	8.812200
S	3.772389	2.471090	14.941495	C	10.919157	-2.878969	7.277126
S	0.608242	2.536728	14.877926	H	12.267183	-3.130616	8.944060
S	9.817458	-5.400124	10.907620	C	9.152598	-1.239378	7.528553
S	9.367053	-6.377552	7.943456	C	9.806238	-2.199512	6.771319
S	-1.254805	1.053274	11.337233	H	11.407048	-3.639136	6.674651
S	-2.835274	-1.531173	12.236833	H	8.271014	-0.733411	7.144825
C	-0.129892	0.968870	15.248253	H	9.430941	-2.446777	5.782571
C	-1.493408	0.716854	15.331861	C	8.182371	-5.402010	7.058528
C	0.832616	-0.042363	15.478707	C	7.633562	-4.367050	7.848681

C	7.805056	-5.597531	5.734385
C	6.690487	-3.510413	7.274860
C	6.869320	-4.728352	5.187035
H	8.230735	-6.403490	5.143993
C	6.321532	-3.690172	5.950953
H	6.269401	-2.698105	7.856816
H	6.555845	-4.860599	4.155808
H	5.585716	-3.031587	5.502631
C	1.858065	-4.695015	10.187850
C	1.499920	-5.505699	8.896126
C	2.706994	-5.962747	8.099736
C	3.916319	-6.084032	9.018851
H	1.641404	-5.293404	11.082886
H	0.920166	-6.395486	9.185141
H	2.920902	-5.208722	7.324626
H	3.711338	-6.730203	9.883528
O	1.051125	-3.538218	10.124503
O	0.715583	-4.592193	8.148452
O	2.435693	-7.241424	7.536217
C	5.094727	-6.611052	8.224443
H	5.970752	-6.791043	8.853824
H	5.366382	-5.893831	7.438180
O	4.217005	-4.737856	9.509002
C	3.294639	-4.200603	10.246900
H	3.448710	-3.137527	10.431863
C	0.061768	-3.742156	9.092345
C	-1.173448	-4.415932	9.672890
H	-1.676393	-3.735317	10.362842
H	-1.861026	-4.679507	8.863825
H	-0.914621	-5.323835	10.226973
C	-0.224942	-2.407044	8.453234
H	-0.955436	-2.527005	7.648136
H	-0.635827	-1.726741	9.205380
H	0.701692	-1.984682	8.062091
O	4.696991	-7.872754	7.695019
C	3.553913	-7.815923	6.835153
C	3.169657	-9.258484	6.577150
H	2.256498	-9.296742	5.978475
H	3.975034	-9.764774	6.039558
H	2.998210	-9.766794	7.528975
C	3.854314	-7.062095	5.541804
H	4.745654	-7.494692	5.077778
H	3.008461	-7.174382	4.858065
H	4.022429	-5.991848	5.683029
C	1.845541	-3.178179	5.168635
H	1.130758	-3.023869	5.990637
C	1.971371	-1.891839	4.360796
H	1.000054	-1.588637	3.953388
H	2.344770	-1.078387	4.993160
H	2.671322	-2.032869	3.529425
C	1.375807	-4.352715	4.330430
H	2.115349	-4.580895	3.554010
H	1.244153	-5.237960	4.960281
H	0.420425	-4.125432	3.847004
O	3.118925	-3.547135	5.722687
H	3.331132	-2.890414	6.405648

TS0′:

O	5.207801	2.042983	10.256831

O	3.409391	1.248412	11.883763	C	2.337191	4.457426	7.009814
O	5.916459	-1.596801	13.803877	C	5.667783	-5.286879	15.840081
O	7.532216	-1.705298	11.817965	C	6.427338	-6.311460	15.294806
S	4.022191	-3.541516	12.463521	F	7.103134	-7.136355	16.090397
S	3.906425	0.235623	8.088192	F	5.636537	-5.119298	17.164257
O	3.231037	-2.597324	13.274290	F	1.887807	5.658346	6.663439
O	3.288234	-4.475759	11.592278	F	0.133926	3.629891	7.173431
O	5.258860	0.170444	7.529188	C	2.594690	2.279547	11.408186
O	2.916040	-0.720449	7.428236	C	5.563645	3.018312	11.188320
N	5.178841	-2.825435	11.611024	C	6.860355	-0.837381	14.487900
P	5.953414	-1.543907	12.177674	C	8.200616	-2.741425	12.473695
N	3.746261	0.011378	9.633927	C	1.230409	1.993658	11.105028
N	5.598288	-0.093872	11.676179	C	8.629796	-3.877296	11.726381
P	4.535909	0.696483	10.852708	C	6.925433	3.123743	11.570562
C	4.947912	-4.607343	13.626326	C	6.445802	0.354267	15.139887
C	4.930444	-4.447812	15.010819	C	2.140800	6.822239	9.787559
C	5.741685	-5.627711	13.098464	C	2.930619	5.842646	10.339305
C	6.471256	-6.477552	13.916173	C	2.378662	4.590439	10.703218
C	3.301382	1.903847	7.677071	C	0.997725	4.349070	10.444151
C	1.933282	2.167915	7.605150	C	0.202895	5.392013	9.903865
C	4.180918	2.964988	7.434788	C	0.761341	6.603283	9.584750
C	3.702783	4.230262	7.114353	H	2.583246	7.770636	9.497930
F	5.502652	2.827891	7.491027	H	3.990781	6.018093	10.489497
F	4.556943	5.229721	6.892100	C	3.154220	3.541479	11.276912
F	1.037958	1.217718	7.855776	C	0.461406	3.073537	10.688196
F	5.827945	-5.815456	11.781174	H	-0.851045	5.202045	9.719028
F	7.213780	-7.453057	13.387489	H	-0.600722	2.934587	10.508162
F	4.210883	-3.498706	15.613275	C	5.612941	6.619170	14.689364
C	1.448510	3.421894	7.261432	C	6.584503	5.876739	14.066387

C	6.248192	4.928658	13.069528	H	10.203255	-6.844336	14.024584
C	4.878426	4.777729	12.686059	H	8.825279	-2.770024	16.517234
C	3.900271	5.547198	13.358647	H	9.717008	-4.717835	17.723255
C	4.259223	6.442312	14.338972	C	9.206273	-4.907050	12.457519
H	8.269997	4.226576	12.809481	C	8.463934	-2.583701	13.819823
H	7.629616	5.990383	14.342929	H	0.149168	7.389066	9.152526
C	7.236068	4.101294	12.496940	H	5.882328	7.334601	15.460545
C	4.551711	3.794855	11.701611	H	11.880123	1.813454	16.673822
H	2.854805	5.416384	13.103611	H	10.439113	-6.747769	16.481159
H	3.490956	7.015207	14.849797	C	5.053522	0.780024	15.128638
C	11.496735	-0.008393	15.568507	C	3.901007	0.036352	15.149087
C	10.546656	-0.832425	15.012709	C	3.020100	2.193394	15.056155
C	9.172874	-0.504113	15.089548	C	2.731205	0.838080	15.126165
C	8.791194	0.714984	15.732015	H	3.895454	-1.045426	15.158218
C	9.794797	1.528657	16.312111	C	0.616306	0.665420	11.162863
C	11.119011	1.176125	16.233639	C	0.891657	-0.478279	11.881097
H	12.547719	-0.270378	15.491032	C	-1.020901	-1.153718	10.731731
H	10.847185	-1.739555	14.498541	C	-0.060710	-1.521856	11.665900
C	8.153725	-1.300925	14.490068	H	1.730704	-0.560158	12.559568
C	7.434577	1.097100	15.761123	C	8.567947	-3.954930	10.272327
H	9.495841	2.448172	16.809031	C	7.899568	-3.214989	9.325364
H	7.162988	2.007788	16.289736	C	9.068696	-4.683345	7.942924
C	10.045978	-5.894693	15.936384	C	8.177333	-3.625533	7.994476
C	9.917642	-5.949742	14.571681	H	7.227058	-2.404871	9.568562
C	9.387021	-4.850768	13.850571	C	7.920016	2.177767	11.080241
C	9.013715	-3.670417	14.559427	C	8.096669	1.630965	9.835736
C	9.141402	-3.651151	15.968332	C	9.743055	0.504673	11.039886
C	9.640725	-4.741484	16.640364	C	9.152802	0.683377	9.796885
H	9.514909	-5.817981	11.951992	H	7.481513	1.894636	8.984014

S	9.054406	1.504896	12.259444
S	10.998651	-0.699147	11.077678
S	4.711219	2.514897	15.064581
S	1.622009	3.220924	14.904042
S	9.594295	-5.189724	9.496467
S	9.482500	-5.211241	6.338813
S	-0.824556	0.440405	10.140668
S	-2.261658	-2.329015	10.430645
C	0.580275	1.786319	14.935163
C	-0.804660	1.784285	14.814283
C	1.315677	0.589465	15.092431
C	-1.466873	0.563248	14.864324
H	-1.355927	2.710179	14.679593
C	0.621972	-0.622064	15.168471
C	-0.758713	-0.629682	15.050628
H	-2.547377	0.538434	14.761320
H	1.174642	-1.547430	15.300531
H	-1.293275	-1.574311	15.084032
C	-0.361855	-2.824033	12.224883
C	-1.538978	-3.373121	11.661869
C	0.276408	-3.550320	13.237664
C	-2.067500	-4.590117	12.073351
C	-0.252282	-4.760823	13.659227
H	1.190919	-3.170224	13.672016
C	-1.417504	-5.279644	13.086212
H	-2.970109	-4.986903	11.618721
H	0.250171	-5.313944	14.447559
H	-1.817361	-6.228527	13.430671
C	10.773840	-0.968118	9.339221

C	11.500471	-1.857794	8.557872
C	9.757653	-0.149489	8.791782
C	11.220436	-1.916751	7.197423
H	12.267997	-2.489099	8.995371
C	9.496139	-0.230135	7.420685
C	10.230507	-1.104590	6.634563
H	11.772818	-2.608206	6.568595
H	8.712711	0.381382	6.981506
H	10.014712	-1.179206	5.572648
C	8.424353	-3.958077	5.671270
C	7.790546	-3.197077	6.678676
C	8.212825	-3.706230	4.320206
C	6.932836	-2.162313	6.301506
C	7.352476	-2.671360	3.970774
H	8.706005	-4.299970	3.556215
C	6.718119	-1.904631	4.956421
H	6.452301	-1.554563	7.057939
H	7.174657	-2.459277	2.920838
H	6.050079	-1.097404	4.671003
C	0.895841	-2.495455	7.809564
C	1.317952	-3.422950	6.652362
C	2.737052	-3.927281	6.818724
C	3.071996	-4.059834	8.298362
H	0.309577	-3.067014	8.558751
H	0.641688	-4.289835	6.601117
H	3.426770	-3.217724	6.332152
H	2.357160	-4.729525	8.786806
O	0.120311	-1.483697	7.223992
O	1.183393	-2.583067	5.517755

O	2.820716	-5.234242	6.261555
C	4.482542	-4.593452	8.437042
H	4.752688	-4.746088	9.483613
H	5.206815	-3.904183	7.974762
O	2.960162	-2.781099	9.010611
C	2.028911	-1.963667	8.638420
H	1.822655	-1.170566	9.354083
C	0.105901	-1.690391	5.784910
C	-1.233079	-2.300152	5.394764
H	-2.043762	-1.616861	5.662503
H	-1.260139	-2.482208	4.316507
H	-1.397915	-3.247070	5.917211
C	0.389934	-0.376465	5.091672
H	0.367202	-0.518864	4.007621
H	-0.369802	0.359178	5.369141
H	1.376017	-0.015683	5.390125
O	4.486819	-5.878072	7.812389
C	4.091703	-5.892560	6.439708
C	3.835152	-7.349299	6.108517
H	3.491443	-7.442075	5.075456
H	4.757401	-7.921353	6.235414
H	3.070901	-7.746020	6.781409
C	5.151468	-5.279069	5.535413
H	6.096697	-5.810873	5.673813
H	4.837531	-5.371851	4.492115
H	5.328638	-4.222394	5.741972
C	1.278322	-6.631475	10.074275
H	2.369287	-6.614727	9.903808
C	0.996669	-7.426032	11.341085
H	1.387972	-8.445567	11.251140
H	1.473414	-6.951077	12.205266
H	-0.081984	-7.473068	11.525059
C	0.594304	-7.220043	8.853731
H	-0.493322	-7.195124	8.986167
H	0.852047	-6.652655	7.952830
H	0.904661	-8.258908	8.703654
O	0.794846	-5.280479	10.198796
H	1.277112	-4.870183	10.933453

c:

O	5.454170	2.329229	10.196917
O	3.640210	1.457555	11.738845
O	5.844354	-1.430796	13.739063
O	7.576012	-1.614539	11.889280
S	4.129302	-3.480280	12.129224
S	4.479322	0.503810	7.928755
O	3.478617	-3.082286	13.382854
O	3.209084	-3.975152	11.068116
O	5.911625	0.712730	7.658980
O	3.782063	-0.521934	7.126699
N	5.068023	-2.401249	11.434723
P	5.997095	-1.298527	12.131912
N	4.133410	0.212396	9.470991
N	5.851589	0.181051	11.625445
P	4.810977	0.941793	10.723320
C	5.217202	-4.891506	12.511583
C	5.680809	-5.133801	13.807201
C	5.632175	-5.755342	11.496005
C	6.432157	-6.856337	11.764141

C	3.645396	2.083768	7.553901	C	1.058370	4.350121	10.213324
C	2.250492	2.112327	7.530686	C	0.206242	5.319371	9.624501
C	4.326536	3.264859	7.256703	C	0.676937	6.566922	9.305240
C	3.633774	4.418262	6.900606	H	2.402407	7.874842	9.257463
F	5.654287	3.352952	7.289783	H	3.914211	6.247470	10.309189
F	4.300267	5.538421	6.611562	C	3.258510	3.723093	11.096654
F	1.541511	1.035338	7.866057	C	0.602260	3.048347	10.483308
F	5.271410	-5.560033	10.224828	H	-0.824366	5.046848	9.412862
F	6.783795	-7.692237	10.786036	H	-0.426972	2.813347	10.233402
F	5.373663	-4.340179	14.828658	C	5.364591	6.946343	14.605884
C	1.552419	3.251103	7.159941	C	6.405085	6.243883	14.051649
C	2.248256	4.406859	6.830506	C	6.175463	5.273182	13.045941
C	6.492567	-6.227802	14.084405	C	4.841054	5.061337	12.574205
C	6.869270	-7.086941	13.062050	C	3.789298	5.786148	13.183035
F	7.662404	-8.124548	13.322746	C	4.044817	6.702343	14.176360
F	6.916241	-6.450089	15.327333	H	8.234280	4.640626	12.946557
F	1.590091	5.499592	6.451689	H	7.424553	6.406100	14.392459
F	0.216408	3.241181	7.119921	C	7.231867	4.476266	12.558130
C	2.776016	2.434799	11.252675	C	4.621306	4.061880	11.574570
C	5.696476	3.318545	11.145986	H	2.767975	5.605088	12.868086
C	6.744637	-0.679978	14.493556	H	3.220001	7.239246	14.635580
C	8.192456	-2.630136	12.609420	C	11.284643	0.195077	15.897674
C	1.426049	2.060822	11.004254	C	10.385380	-0.637790	15.274427
C	8.660560	-3.780572	11.904803	C	9.004289	-0.331460	15.260468
C	7.022072	3.472128	11.632215	C	8.565741	0.878808	15.882624
C	6.277516	0.512013	15.107289	C	9.515485	1.699859	16.538317
C	2.027694	6.897504	9.546195	C	10.846797	1.367187	16.547068
C	2.875093	5.988560	10.132321	H	12.342234	-0.051137	15.885722
C	2.413262	4.701976	10.499631	H	10.733862	-1.534189	14.773481

C	8.036973	-1.142252	14.590709	H	-1.249634	1.177456	11.256716
C	7.209126	1.253390	15.811670	C	8.502330	-3.961664	10.466735
H	9.170021	2.611604	17.019022	C	7.917866	-3.181457	9.496218
H	6.893209	2.160907	16.320279	C	8.644036	-4.967722	8.186736
C	10.156562	-5.596630	16.176665	C	7.993343	-3.746798	8.198176
C	10.060429	-5.683882	14.811365	H	7.468755	-2.219548	9.691015
C	9.460211	-4.641698	14.061189	C	8.060861	2.519365	11.261820
C	8.983009	-3.479638	14.738036	C	8.281088	1.856163	10.082275
C	9.075884	-3.431607	16.148850	C	9.816104	0.781802	11.470233
C	9.646822	-4.466881	16.849613	C	9.287128	0.862191	10.189741
H	9.703411	-5.635542	12.191095	H	7.712506	2.046837	9.179940
H	10.427012	-6.561556	14.284728	S	9.109328	1.914127	12.557702
H	8.681053	-2.570148	16.676806	S	10.998694	-0.478571	11.683728
H	9.699711	-4.417517	17.933047	S	4.563329	2.681197	14.913792
C	9.307255	-4.742376	12.667700	S	1.480451	3.417237	14.743889
C	8.406139	-2.431205	13.960521	S	9.182432	-5.449756	9.747816
H	0.018515	7.295696	8.842131	S	8.799418	-5.693718	6.612059
H	5.553294	7.678830	15.385053	S	1.856915	-0.689313	11.658699
H	11.567360	2.011355	17.042121	S	0.192813	-3.318722	12.256246
H	10.608860	-6.404376	16.744194	C	0.423040	1.993396	14.784040
C	4.890549	0.941902	14.985596	C	-0.967181	2.014725	14.765464
C	3.732944	0.205547	14.933411	C	1.149656	0.782479	14.857965
C	2.870459	2.370430	14.834980	C	-1.643934	0.802696	14.834320
C	2.569303	1.016320	14.872103	H	-1.511156	2.953068	14.708210
H	3.720358	-0.877996	14.942655	C	0.443212	-0.423479	14.927278
C	0.873915	0.742310	11.302422	C	-0.942484	-0.405522	14.920045
C	-0.467921	0.436449	11.368702	H	-2.729884	0.794609	14.823746
C	0.441894	-1.647305	11.830397	H	0.984854	-1.364376	14.974987
C	-0.726952	-0.920562	11.660834	H	-1.491477	-1.341803	14.963055

C	-1.899065	-1.720632	11.881649	H	6.003736	-1.156390	4.840518
C	-1.558161	-3.055470	12.205174	C	1.579369	-3.087513	8.663023
C	-3.251729	-1.367583	11.819921	C	1.180371	-3.950600	7.422511
C	-2.529626	-4.019493	12.450632	C	2.394633	-4.274101	6.585128
C	-4.222971	-2.326655	12.066577	C	3.541148	-4.659914	7.517946
H	-3.532931	-0.345757	11.579898	H	1.373186	-3.666407	9.585668
C	-3.865940	-3.644157	12.377805	H	0.721403	-4.890377	7.758656
H	-2.252828	-5.039960	12.699340	H	2.682808	-3.374753	6.011516
H	-5.272880	-2.053763	12.018635	H	3.224289	-5.412222	8.251840
H	-4.638812	-4.382213	12.569004	O	0.799391	-1.915829	8.579639
C	10.789667	-0.943869	9.984943	O	0.270891	-3.118472	6.730470
C	11.446813	-1.987590	9.343692	O	2.105241	-5.376754	5.748488
C	9.849770	-0.128516	9.312321	C	4.740014	-5.112395	6.708867
C	11.170519	-2.210977	8.000072	H	5.538729	-5.500259	7.347496
H	12.154223	-2.615869	9.876953	H	5.135052	-4.274988	6.118252
C	9.587926	-0.375931	7.959839	O	3.933546	-3.449917	8.282537
C	10.253569	-1.406233	7.314074	C	3.014106	-2.729329	8.766858
H	11.667814	-3.024922	7.480994	H	3.342504	-1.822852	9.283081
H	8.856992	0.230322	7.431400	C	-0.301496	-2.234762	7.702541
H	10.043173	-1.605433	6.267494	C	-1.410389	-2.923500	8.485944
C	7.947627	-4.306138	5.907736	H	-1.842366	-2.228392	9.210333
C	7.580918	-3.348684	6.881092	H	-2.192367	-3.262974	7.800764
C	7.623414	-4.144961	4.564964	H	-1.031201	-3.789513	9.039252
C	6.880589	-2.208104	6.480436	C	-0.744340	-0.970322	7.010924
C	6.925340	-3.001214	4.191281	H	-1.598726	-1.184603	6.363527
H	7.905392	-4.890239	3.827265	H	-1.038937	-0.231094	7.760479
C	6.554949	-2.041721	5.142859	H	0.077620	-0.569488	6.415179
H	6.597140	-1.456928	7.208799	O	4.305023	-6.193474	5.888730
H	6.663619	-2.856457	3.147162	C	3.233202	-5.869109	5.001936

C	2.777019	-7.186237	4.409933
H	3.591872	-7.636444	3.838128
H	2.485977	-7.863970	5.215787
H	1.920809	-7.020399	3.751823
C	3.661790	-4.874741	3.924722
H	4.484068	-5.305976	3.347382
H	2.817493	-4.679634	3.257919
H	4.006375	-3.920697	4.329665
C	1.470783	-7.355920	10.498572
H	1.097983	-7.973642	9.670854
C	2.711406	-8.008603	11.097013
H	2.483689	-9.007967	11.482967
H	3.502065	-8.095525	10.344912
H	3.089066	-7.404396	11.932700
C	0.363744	-7.173216	11.530568
H	-0.498507	-6.673726	11.077474
H	0.038869	-8.138671	11.934021
H	0.722601	-6.557316	12.364577
O	1.805575	-6.098710	9.892823
H	2.254844	-5.551433	10.560092

TS1:

O	5.339522	1.720873	10.271066
O	3.429639	1.235295	11.878966
O	5.733212	-1.542493	14.226257
O	7.384828	-1.720615	12.319393
S	4.351424	-4.086942	13.109009
S	4.083196	0.023095	8.150045
O	3.645063	-3.555010	14.283834
O	3.576529	-5.000349	12.242453
O	5.513301	-0.037086	7.811047
O	3.181201	-0.848524	7.361838
N	5.006329	-2.973147	12.154121
P	5.783518	-1.650218	12.610496
N	3.748641	-0.242221	9.698693
N	5.395872	-0.313837	11.905336
P	4.504847	0.488301	10.914146
C	5.739535	-5.085675	13.752028
C	6.225626	-4.934949	15.053494
C	6.331646	-6.059437	12.945900
C	7.337260	-6.886371	13.423738
C	3.515121	1.714521	7.776688
C	2.143603	1.978773	7.773768
C	4.383336	2.757406	7.449627
C	3.893925	4.009932	7.094534
F	5.706870	2.613466	7.450770
F	4.736694	4.994295	6.775989
F	1.262925	1.040906	8.120150
F	5.957339	-6.230214	11.675120
F	7.878164	-7.821924	12.641017
F	5.750331	-4.016143	15.889261
C	1.647484	3.222628	7.412140
C	2.526034	4.237765	7.056327
C	7.240849	-5.751825	15.539392
C	7.793762	-6.728848	14.725423
F	8.779054	-7.500179	15.181347
F	7.690241	-5.593749	16.783625
F	2.059438	5.424037	6.677525
F	0.331745	3.449090	7.404217

C	2.778088	2.364261	11.406193	C	4.937056	3.570282	11.684374
C	5.832189	2.656160	11.178255	H	3.484960	5.428233	13.075217
C	6.501239	-0.524213	14.788490	H	4.346362	6.949357	14.793322
C	8.224222	-2.453547	13.138537	C	10.829336	1.385964	15.783537
C	1.373623	2.282687	11.170179	C	10.087763	0.304831	15.371272
C	8.877904	-3.589114	12.574631	C	8.674168	0.358321	15.349882
C	7.206622	2.591918	11.534144	C	8.033169	1.575187	15.740161
C	5.832393	0.653340	15.220983	C	8.826733	2.665882	16.174552
C	2.966338	6.889724	9.709021	C	10.195829	2.575411	16.198135
C	3.611497	5.820032	10.282285	H	11.913595	1.325413	15.782588
C	2.891561	4.663930	10.666222	H	10.589703	-0.597785	15.039957
C	1.486997	4.612999	10.412378	C	7.860355	-0.720020	14.879589
C	0.848769	5.744241	9.843633	C	6.628881	1.680831	15.691162
C	1.570964	6.859628	9.503648	H	8.324829	3.580151	16.481397
H	3.536666	7.762085	9.404533	H	6.167457	2.602352	16.038375
H	4.685825	5.848607	10.433339	C	10.868679	-4.407204	16.947801
C	3.517016	3.524647	11.251400	C	10.743694	-4.716962	15.617124
C	0.767907	3.439598	10.701110	C	9.924434	-3.934515	14.765175
H	-0.221516	5.700947	9.659426	C	9.254716	-2.792695	15.301234
H	-0.301245	3.453152	10.508127	C	9.385637	-2.515359	16.682063
C	6.387075	6.241235	14.651702	C	10.171933	-3.305659	17.485397
C	7.240478	5.353544	14.046206	H	10.285375	-5.142934	13.041400
C	6.771866	4.452160	13.057858	H	11.256291	-5.580086	15.200090
C	5.395126	4.496671	12.672136	H	8.845338	-1.675012	17.105003
C	4.539143	5.410784	13.330070	H	10.249499	-3.085471	18.545857
C	5.022759	6.260729	14.295784	C	9.738286	-4.280297	13.413991
H	8.687189	3.519109	12.770971	C	8.451523	-2.001711	14.425503
H	8.292230	5.319866	14.319069	H	1.074574	7.713099	9.051748
C	7.642794	3.514413	12.468062	H	6.757402	6.925140	15.409809

H	10.794147	3.419171	16.529489
H	11.488903	-5.021008	17.594006
C	4.381994	0.764289	15.164810
C	3.418282	-0.205918	15.298608
C	2.085405	1.685694	15.022444
C	2.100501	0.315172	15.238154
H	3.649393	-1.255037	15.437585
C	0.595246	1.074222	11.418553
C	0.946602	-0.167329	11.897653
C	-1.339250	-0.491441	11.599676
C	-0.145925	-1.065197	11.997311
H	1.949113	-0.428215	12.200021
C	8.666112	-4.009709	11.194168
C	7.912168	-3.476927	10.172279
C	8.809643	-5.328156	9.080781
C	7.981835	-4.224360	8.970560
H	7.337731	-2.568277	10.260340
C	8.091246	1.576072	10.979325
C	8.074617	0.947322	9.758025
C	9.907301	-0.095822	10.749190
C	9.117776	-0.003246	9.612143
H	7.337482	1.156171	8.993791
S	9.418418	0.986075	11.994611
S	11.175305	-1.282941	10.656225
S	3.663809	2.368621	14.942142
S	0.496473	2.376312	14.871107
S	9.513252	-5.479519	10.642122
S	8.942207	-6.281249	7.633346
S	-1.156314	1.139587	11.086595
S	-2.728495	-1.527381	11.757525
C	-0.210866	0.773677	15.139700
C	-1.569038	0.482196	15.166051
C	0.771266	-0.225036	15.339705
C	-1.953043	-0.828821	15.422879
H	-2.311415	1.255273	14.991649
C	0.356957	-1.532871	15.609264
C	-0.997630	-1.824000	15.653753
H	-3.009349	-1.079290	15.442643
H	1.096875	-2.313805	15.760861
H	-1.319603	-2.843781	15.842828
C	-0.325435	-2.418786	12.440821
C	-1.681316	-2.808635	12.394347
C	0.622312	-3.331575	12.906033
C	-2.097124	-4.065273	12.818263
C	0.219187	-4.594517	13.313452
H	1.664399	-3.041151	12.985797
C	-1.133223	-4.957530	13.276224
H	-3.145502	-4.348310	12.788384
H	0.965452	-5.293700	13.678848
H	-1.438411	-5.944153	13.612312
C	10.663124	-1.681106	9.007279
C	11.248100	-2.638925	8.188561
C	9.557618	-0.906429	8.581243
C	10.730091	-2.813687	6.910324
H	12.085544	-3.236595	8.536031
C	9.060171	-1.098669	7.288612
C	9.650834	-2.044186	6.464054
H	11.167370	-3.562011	6.256267

H	8.202507	-0.524171	6.949833
H	9.251146	-2.208912	5.467643
C	7.834468	-5.165470	6.816059
C	7.397529	-4.121196	7.662294
C	7.404699	-5.269043	5.497333
C	6.504804	-3.171740	7.159567
C	6.524046	-4.305377	5.018008
H	7.750727	-6.074678	4.856508
C	6.079136	-3.266127	5.845064
H	6.173238	-2.347975	7.783595
H	6.186215	-4.360601	3.987199
H	5.394318	-2.518553	5.457891
C	1.642015	-4.250485	9.727844
C	1.359980	-5.583064	8.976931
C	2.622634	-6.148771	8.358613
C	3.838876	-5.743519	9.193377
H	1.796659	-4.448923	10.812476
H	0.937986	-6.317977	9.678488
H	2.735238	-5.726350	7.351375
H	3.731280	-6.008781	10.250985
O	0.526612	-3.437243	9.498384
O	0.419112	-5.169529	8.004006
O	2.556809	-7.565032	8.352342
C	5.089357	-6.351364	8.592355
H	5.973233	-6.154562	9.202750
H	5.262112	-5.950095	7.583356
O	3.961728	-4.263049	9.143846
C	2.897005	-3.599737	9.303863
H	2.990789	-2.512275	9.218685
C	-0.382374	-4.150750	8.615127
C	-1.512477	-4.765221	9.422720
H	-2.099875	-3.975649	9.896482
H	-2.159018	-5.345039	8.757172
H	-1.131783	-5.418337	10.213041
C	-0.865772	-3.174959	7.569193
H	-1.456582	-3.695963	6.810582
H	-1.493969	-2.419820	8.049580
H	-0.012311	-2.676708	7.106410
O	4.891007	-7.762654	8.597206
C	3.751525	-8.208809	7.863341
C	3.583667	-9.673389	8.211474
H	2.706339	-10.078542	7.701512
H	4.471208	-10.230247	7.902344
H	3.453537	-9.776415	9.291147
C	3.930287	-7.993488	6.362095
H	4.789350	-8.577507	6.020588
H	3.034919	-8.335631	5.835762
H	4.108555	-6.949878	6.093597
C	2.003420	-3.574783	5.340149
H	0.928960	-3.693260	5.540892
C	2.216672	-2.315922	4.512847
H	1.663175	-2.385206	3.570806
H	1.870853	-1.426585	5.049766
H	3.278930	-2.182808	4.277211
C	2.489457	-4.824876	4.624526
H	3.576400	-4.788468	4.491320
H	2.234274	-5.722907	5.194596
H	2.013454	-4.909795	3.641882

O	2.690820	-3.481449	6.606465
H	2.950309	-2.554778	6.751875

TS1′:

O	5.312218	1.891657	10.160872
O	3.403738	1.135130	11.676647
O	5.851361	-1.754245	13.729641
O	7.544293	-1.790775	11.806398
S	4.106683	-3.757691	12.274692
S	4.366483	0.071433	7.941838
O	3.159230	-2.893682	12.998845
O	3.516568	-4.761942	11.355717
O	5.830457	0.166835	7.811277
O	3.669056	-0.892881	7.071545
N	5.245640	-2.966264	11.488662
P	5.951844	-1.661216	12.103888
N	3.858786	-0.153618	9.450502
N	5.570224	-0.215771	11.639432
P	4.573759	0.549282	10.701922
C	5.005832	-4.759873	13.507761
C	4.901083	-4.570833	14.884506
C	5.863800	-5.761986	13.049342
C	6.567388	-6.572191	13.928380
C	3.696953	1.708885	7.470917
C	2.316675	1.851952	7.326161
C	4.493591	2.826524	7.220427
C	3.930025	4.029287	6.805384
F	5.819182	2.806889	7.351914
F	4.708303	5.087163	6.563029
F	1.491315	0.838184	7.591710
F	6.034392	-5.975136	11.745039
F	7.361347	-7.540037	13.465888
F	4.109745	-3.637951	15.418467
C	1.746077	3.041136	6.898462
C	2.558588	4.133146	6.624995
C	5.617207	-5.364462	15.774022
C	6.439626	-6.375383	15.297579
F	7.094417	-7.158120	16.150618
F	5.504364	-5.168542	17.089987
F	2.027968	5.273019	6.187953
F	0.421505	3.137171	6.745585
C	2.613389	2.143981	11.130201
C	5.582921	2.897947	11.076664
C	6.765329	-1.003653	14.462472
C	8.200458	-2.839246	12.452213
C	1.261611	1.843870	10.797680
C	8.646774	-3.956854	11.687641
C	6.912095	3.046838	11.551856
C	6.321999	0.175253	15.116480
C	2.221986	6.590090	9.250780
C	2.984113	5.648842	9.899498
C	2.421328	4.410346	10.292072
C	1.060915	4.141143	9.961085
C	0.293376	5.144806	9.318026
C	0.860209	6.345135	8.973390
H	2.674027	7.527228	8.940049
H	4.031902	5.842475	10.104391
C	3.170733	3.402146	10.965320
C	0.512970	2.881408	10.259687

H	-0.745121	4.933433	9.077381	C	8.988343	-3.799232	14.534277
H	-0.540103	2.728941	10.042024	C	9.074152	-3.821643	15.946906
C	5.350705	6.702431	14.358282	C	9.615162	-4.904364	16.597795
C	6.369958	5.935852	13.851449	H	9.681223	-5.828819	11.881752
C	6.112919	4.923189	12.895504	H	10.367178	-6.882918	13.933048
C	4.775307	4.728234	12.427799	H	8.695520	-2.977791	16.514502
C	3.745715	5.523576	12.984509	H	9.658627	-4.913957	17.682744
C	4.026636	6.483601	13.928346	C	9.288219	-4.960779	12.402942
H	8.155176	4.239498	12.812663	C	8.424550	-2.710448	13.809982
H	7.392439	6.081336	14.190624	H	0.270460	7.100205	8.462488
C	7.146149	4.079055	12.440671	H	5.559332	7.468942	15.098612
C	4.531907	3.691842	11.473464	H	11.654307	1.598747	16.994429
H	2.722287	5.366325	12.664317	H	10.534163	-6.855035	16.396774
H	3.218680	7.075994	14.347448	C	4.940582	0.639198	15.032388
C	11.340653	-0.180366	15.800542	C	3.755643	-0.040564	15.144686
C	10.426881	-0.986923	15.164375	C	2.981318	2.136822	14.821814
C	9.047619	-0.670762	15.181755	C	2.623705	0.816180	15.057663
C	8.627661	0.520484	15.850309	H	3.707135	-1.114043	15.282629
C	9.592630	1.316832	16.513652	C	0.634592	0.537879	11.008247
C	10.921464	0.975081	16.491268	C	0.860579	-0.475163	11.914883
H	12.395999	-0.434067	15.766646	C	-1.085111	-1.251887	10.890735
H	10.760889	-1.870876	14.632274	C	-0.134107	-1.500852	11.871914
C	8.064658	-1.455125	14.506728	H	1.687065	-0.466313	12.613906
C	7.271382	0.900286	15.813398	C	8.491336	-4.071257	10.241377
H	9.260631	2.215493	17.027305	C	8.115482	-3.170155	9.269045
H	6.965802	1.798057	16.344995	C	8.669424	-5.001008	7.939016
C	10.106502	-6.008137	15.868616	C	8.214910	-3.692594	7.948549
C	10.017857	-6.024121	14.500139	H	7.818357	-2.153035	9.487248
C	9.439984	-4.934845	13.799622	C	7.960183	2.099399	11.189294

C	8.270243	1.513893	9.989572
C	9.830158	0.490282	11.390221
C	9.359814	0.606481	10.089518
H	7.719644	1.713690	9.077639
S	8.997650	1.510260	12.496718
S	11.087885	-0.693447	11.603975
S	4.684354	2.368368	14.772382
S	1.638502	3.221729	14.593224
S	8.949775	-5.630314	9.509820
S	8.946808	-5.674332	6.362855
S	-0.800173	0.194928	10.014384
S	-2.405490	-2.380013	10.834003
C	0.522420	1.870447	14.843271
C	-0.864269	1.945270	14.787136
C	1.194736	0.656690	15.118720
C	-1.596761	0.788833	15.028062
H	-1.363842	2.882163	14.559271
C	0.431242	-0.479956	15.398930
C	-0.952079	-0.411841	15.344818
H	-2.680778	0.823897	14.979828
H	0.926386	-1.411032	15.654080
H	-1.538635	-1.304979	15.538656
C	-0.476222	-2.678856	12.639139
C	-1.703784	-3.237639	12.214293
C	0.193407	-3.298069	13.699410
C	-2.276847	-4.340351	12.837213
C	-0.371214	-4.398312	14.325705
H	1.164944	-2.932581	14.001411
C	-1.604252	-4.912036	13.909391
H	-3.229317	-4.739698	12.500908
H	0.156529	-4.870661	15.149242
H	-2.037044	-5.768334	14.418016
C	11.025442	-1.051407	9.869720
C	11.803859	-1.994740	9.210451
C	10.073513	-0.257850	9.186882
C	11.646378	-2.132481	7.836344
H	12.515378	-2.610437	9.752647
C	9.954158	-0.400204	7.801284
C	10.738713	-1.330497	7.136931
H	12.238516	-2.869536	7.302475
H	9.240292	0.210049	7.256042
H	10.628369	-1.453603	6.063652
C	8.443432	-4.139606	5.639995
C	8.041880	-3.190187	6.608665
C	8.446759	-3.850095	4.279492
C	7.582219	-1.945367	6.170177
C	8.017992	-2.591125	3.874145
H	8.775719	-4.586768	3.552366
C	7.574259	-1.654030	4.814473
H	7.225432	-1.217241	6.888596
H	8.015418	-2.343573	2.816854
H	7.214757	-0.684501	4.482359
C	0.621630	-3.015277	8.127632
C	1.049310	-4.042741	7.072781
C	2.554049	-3.998666	6.885627
C	3.267338	-4.119355	8.222341
H	-0.261224	-3.329678	8.701915
H	0.739707	-5.063655	7.326309

H	2.822213	-3.035630	6.426916
H	2.972576	-5.024148	8.764905
O	0.373329	-1.838662	7.369395
O	0.346974	-3.624789	5.912911
O	2.969401	-5.100706	6.103221
C	4.772062	-4.072265	8.018665
H	5.308239	-4.268597	8.950742
H	5.068633	-3.086877	7.631652
O	2.873111	-2.998372	9.105796
C	1.680723	-2.607404	9.106479
H	1.477036	-1.802395	9.818900
C	-0.088054	-2.258631	6.070496
C	-1.606359	-2.234074	6.031797
H	-1.968009	-1.208192	6.144708
H	-1.959390	-2.636353	5.077920
H	-2.014610	-2.849116	6.839901
C	0.562764	-1.364203	5.036359
H	0.283813	-1.700755	4.034137
H	0.224363	-0.332960	5.173301
H	1.649584	-1.392374	5.145196
O	5.093459	-5.131417	7.118240
C	4.395127	-5.106473	5.869370
C	4.694914	-6.436771	5.212329
H	4.192576	-6.494540	4.243613
H	5.774072	-6.534402	5.069840
H	4.343141	-7.249280	5.852606
C	4.805083	-3.928685	4.994528
H	5.844605	-4.054965	4.687901
H	4.171635	-3.909664	4.102429
H	4.725433	-2.961179	5.495287
C	0.538362	-5.987717	10.170779
H	1.198601	-6.394300	9.383305
C	0.721722	-6.808428	11.439053
H	0.455800	-7.854527	11.252824
H	1.761827	-6.774288	11.778581
H	0.078978	-6.414172	12.233196
C	-0.898887	-6.017317	9.681572
H	-1.560235	-5.606226	10.450368
H	-1.034197	-5.436558	8.763937
H	-1.199812	-7.048127	9.470925
O	0.897429	-4.608467	10.421419
H	1.726281	-4.616097	10.941089

β-isomer:

O	5.507113	1.512279	9.986758
O	3.698820	0.999380	11.689818
O	5.851897	-1.712929	13.918782
O	7.552955	-2.142734	12.047924
S	4.110713	-3.988416	12.910481
S	4.178347	-0.347297	7.958752
O	3.181601	-3.089384	13.622507
O	3.497685	-5.106170	12.172715
O	5.586845	-0.539618	7.596346
O	3.204495	-1.223466	7.231490
N	5.141804	-3.234140	11.933721
P	5.959767	-1.916201	12.307105
N	3.834994	-0.418744	9.504868
N	5.718820	-0.566479	11.535703
P	4.727842	0.258575	10.663653

C	5.156057	-4.781741	14.183056	C	6.357504	0.379198	15.018812
C	5.131737	-4.430608	15.532204	C	2.362689	6.257387	8.948119
C	5.987668	-5.834981	13.796870	C	3.173871	5.355528	9.593084
C	6.728818	-6.551055	14.725177	C	2.642355	4.153963	10.119581
C	3.725819	1.337395	7.405239	C	1.251981	3.875811	9.939596
C	2.381935	1.692478	7.290573	C	0.442151	4.836492	9.280809
C	4.676423	2.303698	7.068750	C	0.982390	6.001385	8.799798
C	4.290670	3.559287	6.609251	H	2.787712	7.168952	8.538959
F	5.985091	2.081302	7.159301	H	4.235799	5.553907	9.699164
F	5.214098	4.472311	6.303659	C	3.445831	3.187044	10.787814
F	1.409990	0.844049	7.641892	C	0.715064	2.658389	10.396690
F	6.093247	-6.190411	12.516568	H	-0.615016	4.622164	9.147120
F	7.502543	-7.567559	14.335988	H	-0.344517	2.483530	10.240754
F	4.396741	-3.419381	15.995464	C	5.892272	6.581212	13.864050
C	1.985198	2.925214	6.795733	C	6.861780	5.751216	13.360681
C	2.946514	3.865536	6.451903	C	6.530489	4.703945	12.466062
C	5.879108	-5.136598	16.469327	C	5.172677	4.544643	12.048702
C	6.660461	-6.210597	16.070052	C	4.193928	5.401780	12.607187
F	7.343051	-6.910026	16.973382	C	4.545389	6.391689	13.494265
F	5.839017	-4.791387	17.758965	H	8.534000	3.915405	12.373959
F	2.583093	5.050491	5.970909	H	7.900472	5.869061	13.658975
F	0.689100	3.201300	6.638912	C	7.508237	3.783882	12.038271
C	2.877467	1.975420	11.132388	C	4.850425	3.463229	11.169830
C	5.849685	2.587713	10.803972	H	3.151386	5.262722	12.344575
C	6.783405	-0.889032	14.539850	H	3.775060	7.029127	13.918282
C	8.238982	-3.044142	12.851690	C	11.381346	0.068608	15.687297
C	1.492873	1.680955	11.001469	C	10.448330	-0.821314	15.209622
C	8.805015	-4.209333	12.252267	C	9.071211	-0.494701	15.205852
C	7.197549	2.704783	11.232228	C	8.674854	0.793993	15.678966

C	9.658609	1.674835	16.191123
C	10.984605	1.321761	16.196592
H	12.434421	-0.195826	15.667366
H	10.768442	-1.779140	14.814515
C	8.069396	-1.360555	14.667972
C	7.323678	1.183044	15.598031
H	9.343214	2.647422	16.560468
H	7.038516	2.150807	16.003145
C	10.227155	-5.595250	16.709140
C	10.159045	-5.814761	15.356889
C	9.548006	-4.865105	14.499567
C	9.027245	-3.660465	15.058208
C	9.095385	-3.472139	16.458671
C	9.677642	-4.420807	17.265189
H	9.909981	-5.988622	12.724850
H	10.555906	-6.729118	14.923683
H	8.669197	-2.573661	16.893407
H	9.707092	-4.269322	18.339961
C	9.453828	-5.082966	13.114365
C	8.428739	-2.711609	14.179369
H	0.353259	6.722876	8.287232
H	6.156359	7.372335	14.559406
H	11.731949	2.010574	16.579015
H	10.684100	-6.335777	17.358706
C	4.973977	0.823704	14.910050
C	3.798541	0.121782	15.011039
C	2.984581	2.288197	14.716995
C	2.652494	0.957680	14.928751
H	3.761189	-0.953492	15.139538
C	0.887217	0.446604	11.491763
C	-0.463664	0.190036	11.594371
C	0.394785	-1.829821	12.357247
C	-0.756529	-1.107280	12.071509
H	-1.227776	0.919439	11.354391
C	8.782133	-4.465248	10.818066
C	8.338382	-3.711769	9.754064
C	9.233791	-5.530817	8.610498
C	8.578461	-4.318333	8.492393
H	7.880858	-2.738888	9.869371
C	8.189199	1.679818	10.924802
C	8.459384	1.001609	9.764995
C	9.975569	-0.007559	11.222283
C	9.504107	0.049843	9.917689
H	7.918652	1.177612	8.842284
S	9.205429	1.125881	12.262531
S	11.204099	-1.210371	11.497439
S	4.684611	2.554041	14.668134
S	1.620473	3.361890	14.570466
S	9.542559	-5.972467	10.241977
S	9.564251	-6.331232	7.103743
S	1.835569	-0.941446	12.060730
S	0.106956	-3.414757	13.019772
C	0.530033	1.988334	14.839085
C	-0.858108	2.051721	14.884691
C	1.228222	0.772168	15.023318
C	-1.562002	0.878483	15.129791
H	-1.379830	2.993214	14.739820
C	0.494860	-0.392655	15.275417

C	-0.888754	-0.332292	15.329267	H	8.983277	-5.687583	4.204336
H	-2.646957	0.903831	15.169633	C	7.390254	-2.842449	5.211776
H	1.014350	-1.337083	15.413005	H	7.171521	-2.161538	7.236308
H	-1.458949	-1.238767	15.511214	H	7.758057	-3.739261	3.282987
C	-1.948439	-1.854389	12.365182	H	6.841540	-1.999516	4.801923
C	-1.637146	-3.137919	12.873521	C	1.307514	-4.873584	9.743410
C	-3.292592	-1.490155	12.229328	C	1.870024	-6.113190	8.989023
C	-2.632034	-4.041998	13.230430	C	3.072542	-5.734383	8.160489
C	-4.286241	-2.389373	12.586735	C	3.957692	-4.860804	9.045529
H	-3.550073	-0.505097	11.849570	H	1.493233	-4.972645	10.823477
C	-3.959262	-3.657575	13.082092	H	2.182732	-6.882780	9.709881
H	-2.379050	-5.022880	13.622373	H	2.745344	-5.178051	7.269009
H	-5.329831	-2.107454	12.483915	H	4.182326	-5.386606	9.981756
H	-4.749329	-4.349051	13.358667	O	-0.081558	-4.863504	9.450712
C	11.137431	-1.649678	9.780959	O	0.764167	-6.556784	8.210195
C	11.905974	-2.630108	9.166297	O	3.800395	-6.898793	7.802387
C	10.196106	-0.876363	9.060028	C	5.241756	-4.539634	8.318299
C	11.751851	-2.824815	7.798382	H	5.933222	-4.017280	8.983248
H	12.608794	-3.229817	9.736831	H	5.045659	-3.901285	7.442941
C	10.073211	-1.083363	7.682877	O	3.320374	-3.601037	9.393845
C	10.851305	-2.048420	7.062314	C	1.976824	-3.556618	9.389440
H	12.336529	-3.591852	7.299658	H	1.637098	-2.713677	9.995299
H	9.359583	-0.498833	7.109224	C	-0.399661	-6.176841	8.936067
H	10.737297	-2.222918	5.996441	C	-0.685578	-7.122888	10.098075
C	8.760241	-5.006863	6.243477	H	-1.584808	-6.792340	10.625986
C	8.272936	-4.012043	7.120680	H	-0.840325	-8.138903	9.724000
C	8.593711	-4.919068	4.865677	H	0.141936	-7.135504	10.812785
C	7.574848	-2.927779	6.583375	C	-1.563186	-6.066429	7.977493
C	7.903985	-3.824381	4.355746	H	-1.872477	-7.064151	7.655769

H	-2.406393	-5.579595	8.474598
H	-1.274757	-5.492920	7.093390
O	5.861866	-5.772514	7.966672
C	5.058621	-6.623840	7.155020
C	5.785077	-7.952003	7.107659
H	5.201045	-8.676633	6.535001
H	6.763595	-7.817733	6.639877
H	5.924803	-8.322401	8.126117
C	4.856175	-6.038289	5.757622
H	4.329930	-5.080758	5.762577
H	5.835022	-5.872263	5.298348
H	4.281705	-6.741016	5.147562
C	0.216792	-2.637715	7.542904
H	-0.351825	-3.565525	7.513033
C	-0.331699	-1.710205	8.604104
H	-1.252383	-1.252122	8.228198
H	-0.581438	-2.249558	9.520889
H	0.370953	-0.906395	8.839313
C	0.324411	-2.033617	6.158130
H	0.879663	-1.093211	6.165925
H	0.806988	-2.729192	5.465063
H	-0.688260	-1.836046	5.792939
O	1.603027	-3.105586	7.902520
H	2.275866	-2.343090	7.710227

α-isomer:

O	5.472176	1.812462	10.072409
O	3.564736	1.163529	11.606266
O	5.680448	-1.630223	13.894281
O	7.407963	-1.978812	12.053445
S	4.000155	-3.778886	12.788803
S	4.385930	0.051002	7.840982
O	3.003079	-3.036711	13.560702
O	3.384691	-4.881512	11.940830
O	5.840709	0.130363	7.618336
O	3.615825	-0.873568	6.996991
N	4.954480	-2.968289	11.822425
P	5.828259	-1.681224	12.278594
N	3.986403	-0.215880	9.380731
N	5.627085	-0.305109	11.585521
P	4.685133	0.506054	10.618028
C	5.010429	-4.701670	13.985977
C	4.977985	-4.444382	15.358067
C	5.802753	-5.759123	13.537175
C	6.483550	-6.581873	14.419749
C	3.718830	1.709703	7.436936
C	2.339158	1.857285	7.288573
C	4.521715	2.816645	7.159477
C	3.966033	4.007190	6.701899
F	5.846462	2.795199	7.300864
F	4.749754	5.055630	6.433948
F	1.507257	0.859974	7.590574
F	5.922011	-6.018143	12.230843
F	7.217803	-7.598904	13.965879
F	4.289337	-3.433406	15.878494
C	1.776581	3.033646	6.816249
C	2.596724	4.109660	6.505815
C	5.668667	-5.257451	16.251032
C	6.402205	-6.339426	15.785550

F	7.027923	-7.137805	16.642765
F	5.618376	-5.006857	17.558746
F	2.074651	5.236785	6.026263
F	0.453989	3.131994	6.654226
C	2.756122	2.150535	11.050420
C	5.724457	2.858279	10.950813
C	6.607399	-0.860860	14.594912
C	8.027935	-2.953725	12.823813
C	1.386599	1.835791	10.837370
C	8.511102	-4.127236	12.171657
C	7.042918	2.995376	11.459690
C	6.181121	0.385243	15.123273
C	2.248350	6.514401	9.032954
C	3.046224	5.603912	9.682388
C	2.518109	4.369551	10.130503
C	1.147623	4.067475	9.859593
C	0.349553	5.036727	9.199035
C	0.884285	6.235085	8.801541
H	2.673054	7.450535	8.683225
H	4.096835	5.820228	9.848408
C	3.309853	3.393357	10.801671
C	0.617903	2.818286	10.230601
H	-0.692210	4.802487	8.996670
H	-0.424153	2.619431	10.001480
C	5.497547	6.781522	14.089759
C	6.514392	5.989537	13.619080
C	6.258108	4.949250	12.692591
C	4.926649	4.758536	12.205917
C	3.897928	5.576240	12.732078
C	4.176393	6.558887	13.653055
H	8.285392	4.216458	12.694883
H	7.533839	6.132839	13.968359
C	7.283207	4.064470	12.301280
C	4.680114	3.692939	11.282328
H	2.874990	5.415890	12.412165
H	3.367682	7.165860	14.049398
C	11.163636	-0.069834	15.997227
C	10.240680	-0.905389	15.413548
C	8.871768	-0.551330	15.364036
C	8.472919	0.710314	15.906316
C	9.445424	1.533846	16.524332
C	10.763178	1.153968	16.570477
H	12.211527	-0.354268	16.011546
H	10.563245	-1.840357	14.969635
C	7.880871	-1.364085	14.730154
C	7.134044	1.133888	15.790335
H	9.128987	2.485408	16.943730
H	6.848054	2.084183	16.234235
C	9.905749	-5.769919	16.546924
C	9.844581	-5.910965	15.183836
C	9.265374	-4.899429	14.376462
C	8.778630	-3.709910	14.996844
C	8.835502	-3.604948	16.406777
C	9.380997	-4.614657	17.163641
H	9.561893	-5.961489	12.551271
H	10.220966	-6.810182	14.703052
H	8.433539	-2.719757	16.889055
H	9.403331	-4.524597	18.245439

C	9.147264	-5.055569	12.984571
C	8.222297	-2.692335	14.167230
H	0.265818	6.963207	8.285385
H	5.704742	7.567669	14.809647
H	11.501905	1.800096	17.035176
H	10.337908	-6.557323	17.157108
C	4.813637	0.860987	14.957670
C	3.623642	0.177355	14.992500
C	2.859606	2.356572	14.672283
C	2.496845	1.029800	14.858130
H	3.561250	-0.896697	15.118794
C	0.768981	0.593112	11.289599
C	-0.583839	0.389712	11.461586
C	0.231845	-1.677158	12.139029
C	-0.903099	-0.902007	11.936282
H	-1.322936	1.165463	11.303074
C	8.399135	-4.349242	10.735619
C	7.959865	-3.545487	9.707765
C	8.670992	-5.398481	8.487667
C	8.110780	-4.134891	8.425822
H	7.581567	-2.543787	9.852523
C	8.060997	1.985759	11.190097
C	8.354872	1.284044	10.050047
C	9.815833	0.277241	11.565861
C	9.375021	0.315334	10.250387
H	7.836247	1.442491	9.111189
S	9.034174	1.439566	12.566061
S	10.996430	-0.960119	11.898039
S	4.565147	2.595203	14.708607
S	1.518710	3.453450	14.490613
S	9.008002	-5.903228	10.098295
S	8.974531	-6.136973	6.941595
S	1.685172	-0.850545	11.753406
S	-0.082708	-3.239341	12.843188
C	0.397760	2.097507	14.717509
C	-0.989341	2.189579	14.744893
C	1.067821	0.866710	14.905320
C	-1.719604	1.031928	14.985002
H	-1.489438	3.142102	14.595906
C	0.307870	-0.283700	15.144508
C	-1.074114	-0.192692	15.190945
H	-2.804078	1.079388	15.015473
H	0.804814	-1.239802	15.283938
H	-1.666032	-1.085195	15.371001
C	-2.101792	-1.582141	12.342250
C	-1.815097	-2.871636	12.850000
C	-3.431614	-1.148517	12.315056
C	-2.821049	-3.714422	13.310816
C	-4.435668	-1.984742	12.779433
H	-3.668614	-0.159016	11.933808
C	-4.133773	-3.259965	13.272729
H	-2.587650	-4.702356	13.696956
H	-5.468118	-1.648996	12.759436
H	-4.931825	-3.903243	13.630572
C	10.927446	-1.462079	10.199087
C	11.647913	-2.507210	9.634018
C	10.036340	-0.670182	9.436677
C	11.495128	-2.750798	8.273795

H	12.313468	-3.118180	10.236580	H	4.629600	-2.782465	5.433337
C	9.915958	-0.925572	8.067064	O	4.147586	-3.888256	7.677566
C	10.645972	-1.956754	7.496027	C	3.397616	-4.058079	8.810661
H	12.044563	-3.566004	7.812627	H	3.544832	-3.184975	9.457475
H	9.243234	-0.324601	7.462060	C	-0.079330	-3.145155	8.387931
H	10.537510	-2.164416	6.435475	C	-1.162282	-3.651951	9.321782
C	8.353578	-4.676934	6.142595	H	-1.342921	-2.928933	10.119567
C	7.908065	-3.705735	7.067779	H	-2.087680	-3.794234	8.756420
C	8.336034	-4.446193	4.770568	H	-0.873252	-4.609241	9.765133
C	7.426025	-2.485664	6.587108	C	-0.337502	-1.742026	7.881644
C	7.879535	-3.212035	4.320228	H	-1.309117	-1.702401	7.381251
H	8.681998	-5.199963	4.069827	H	-0.339246	-1.042163	8.723647
C	7.428569	-2.241776	5.223184	H	0.446175	-1.446415	7.181107
H	7.053985	-1.734541	7.274930	O	3.974744	-4.252039	4.067383
H	7.864130	-3.008912	3.253796	C	2.628474	-3.816138	3.867665
H	7.056462	-1.289749	4.855962	C	2.153766	-4.510315	2.607211
C	1.882102	-4.335017	8.661513	H	1.111410	-4.248502	2.409941
C	1.340844	-4.601930	7.244512	H	2.771173	-4.202789	1.759777
C	2.220475	-3.929266	6.214371	H	2.234515	-5.592278	2.736305
C	3.647215	-4.405961	6.432755	C	2.535246	-2.294750	3.752268
H	1.605497	-5.161240	9.340096	H	3.129262	-1.964016	2.895453
H	1.275732	-5.674517	7.009811	H	1.491380	-2.009869	3.591693
H	2.189546	-2.838281	6.357569	H	2.897967	-1.770694	4.639267
H	3.676760	-5.509072	6.416098	C	4.009024	-6.535985	9.081155
O	1.210598	-3.143770	9.026728	H	3.247660	-6.533446	8.291918
O	0.027375	-4.078920	7.291883	C	5.383280	-6.821867	8.514157
O	1.787720	-4.314224	4.919052	H	5.391293	-7.810833	8.044382
C	4.534564	-3.870907	5.323280	H	5.668972	-6.073462	7.770958
H	5.533822	-4.311702	5.362260	H	6.129683	-6.808373	9.314739

C	3.592271	-7.490333	10.182502
H	4.316887	-7.484849	11.001035
H	2.604747	-7.243456	10.583123
H	3.553623	-8.502253	9.768230
O	4.030523	-5.154577	9.626029
H	3.765547	-5.039557	10.704099

TS1″:

O	5.337672	1.354870	10.036560
O	3.417822	1.196721	11.652146
O	5.471600	-1.822072	14.268338
O	7.130564	-2.033237	12.386423
S	3.622228	-3.856497	13.089866
S	4.131377	-0.693575	8.202930
O	2.760605	-3.007827	13.923000
O	2.931019	-4.852878	12.242612
O	5.564884	-0.804172	7.903854
O	3.240820	-1.702740	7.586193
N	4.661981	-3.063286	12.153331
P	5.534096	-1.815317	12.647147
N	3.758612	-0.665606	9.765400
N	5.302034	-0.386602	12.038529
P	4.481527	0.250201	10.862296
C	4.668599	-4.860560	14.205035
C	4.719877	-4.679910	15.586728
C	5.439982	-5.885634	13.655864
C	6.201600	-6.731127	14.447265
C	3.537622	0.886259	7.502322
C	2.160863	1.093996	7.387398
C	4.388559	1.877119	7.012186
C	3.882186	3.016266	6.394521
F	5.713072	1.782740	7.099047
F	4.712934	3.951346	5.926941
F	1.285655	0.213622	7.870822
F	5.471605	-6.091219	12.334926
F	6.922019	-7.709733	13.893283
F	4.046906	-3.711629	16.202859
C	1.648196	2.222176	6.764325
C	2.512268	3.181042	6.253001
C	5.484239	-5.519931	16.391182
C	6.210431	-6.557230	15.825279
F	6.917071	-7.377628	16.598266
F	5.516895	-5.335259	17.713013
F	2.029097	4.256379	5.636216
F	0.327106	2.390663	6.656621
C	2.713042	2.127956	10.907279
C	5.725941	2.513848	10.692453
C	6.429565	-1.115827	14.979291
C	7.782022	-3.061005	13.045350
C	1.338124	1.856329	10.674938
C	8.235490	-4.148185	12.248276
C	7.086429	2.599086	11.084064
C	6.018893	0.110167	15.562039
C	2.527656	6.201579	8.322226
C	3.267787	5.312509	9.063980
C	2.650937	4.208971	9.700359
C	1.248724	3.999900	9.505000
C	0.511469	4.953999	8.758269
C	1.133313	6.033848	8.185576

H	3.021120	7.033900	7.829674	H	6.683965	1.781583	16.701549
H	4.340658	5.446238	9.161621	C	9.886020	-6.206051	16.340524
C	3.371690	3.274970	10.501256	C	9.764596	-6.195268	14.974729
C	0.625519	2.848981	10.021940	C	9.120359	-5.120863	14.309400
H	-0.555514	4.795870	8.624667	C	8.636292	-4.019330	15.082576
H	-0.444316	2.739706	9.875738	C	8.745377	-4.078941	16.492454
C	6.103063	6.902605	13.115299	C	9.352745	-5.147580	17.106489
C	6.980577	5.892388	12.813248	H	9.350101	-5.977910	12.364037
C	6.561479	4.754458	12.081426	H	10.140367	-7.023928	14.380089
C	5.201679	4.665587	11.636953	H	8.332719	-3.270689	17.088225
C	4.323732	5.723886	11.977432	H	9.413405	-5.183047	18.190003
C	4.761674	6.810800	12.695623	C	8.939227	-5.138562	12.914825
H	8.513580	3.820508	12.088007	C	8.031138	-2.923356	14.400329
H	8.017481	5.946535	13.134811	H	0.559368	6.746536	7.600985
C	7.476328	3.721839	11.778731	H	6.436954	7.768297	13.679298
C	4.782722	3.497053	10.917136	H	11.293344	1.333572	17.696676
H	3.288673	5.675480	11.661494	H	10.366776	-7.042618	16.838538
H	4.062873	7.604072	12.944437	C	4.632001	0.568486	15.433359
C	10.944505	-0.505251	16.606695	C	4.163789	1.713544	14.848437
C	10.030510	-1.286327	15.940600	C	2.167668	0.720344	15.525052
C	8.680916	-0.880126	15.799185	C	2.744411	1.806047	14.883491
C	8.300347	0.383424	16.358273	H	4.819583	2.425020	14.356628
C	9.266183	1.159637	17.044301	C	0.687202	0.614456	11.085039
C	10.562210	0.727752	17.169994	C	-0.544068	0.169955	10.654178
H	11.973130	-0.842705	16.694234	C	0.028717	-1.552568	12.102795
H	10.345673	-2.231161	15.515133	C	-0.935936	-1.059582	11.233669
C	7.698258	-1.647622	15.088062	H	-1.138390	0.699915	9.919666
C	6.969136	0.840718	16.238168	C	8.005690	-4.234107	10.806625
H	8.956912	2.111208	17.468960	C	8.353791	-5.290617	9.990005

C	7.368429	-3.877908	8.432163
C	7.995590	-5.100579	8.634283
H	8.832921	-6.191202	10.353927
C	8.026455	1.519650	10.767767
C	8.788260	0.777350	11.629383
C	9.415813	-0.148313	9.585631
C	9.581091	-0.197090	10.961725
H	8.725910	0.892954	12.706858
S	8.279837	1.041903	9.085262
S	10.289529	-1.370315	8.704907
S	3.322320	-0.432561	16.067525
S	0.426118	0.719599	15.529093
S	7.213123	-2.950830	9.866745
S	6.793194	-3.616650	6.810549
S	1.405119	-0.532882	12.235262
S	-0.396247	-3.051874	12.880691
C	0.438854	2.228127	14.604975
C	-0.682416	2.912553	14.147764
C	1.749944	2.697454	14.354160
C	-0.491256	4.088155	13.432301
H	-1.682447	2.534810	14.338550
C	1.912179	3.895179	13.652291
C	0.798234	4.579832	13.193911
H	-1.354324	4.633031	13.061682
H	2.912695	4.275921	13.474408
H	0.922700	5.506158	12.639716
C	-2.095711	-1.909112	11.180361
C	-1.956965	-3.023334	12.042632
C	-3.282411	-1.768907	10.451729

C	-2.972949	-3.959636	12.198198
C	-4.295984	-2.705035	10.601187
H	-3.408306	-0.923418	9.780995
C	-4.146179	-3.790425	11.472805
H	-2.855528	-4.802462	12.873338
H	-5.218926	-2.591861	10.040320
H	-4.952040	-4.509463	11.584319
C	10.894977	-2.018521	10.235509
C	11.712117	-3.134619	10.380287
C	10.447708	-1.274103	11.352461
C	12.090590	-3.512734	11.662447
H	12.038928	-3.702153	9.514045
C	10.862506	-1.663383	12.629305
C	11.673578	-2.776544	12.778804
H	12.722630	-4.385576	11.796303
H	10.539452	-1.089912	13.492043
H	11.987767	-3.087958	13.771279
C	7.390270	-5.222579	6.360300
C	8.011315	-5.900229	7.437956
C	7.262775	-5.807667	5.104463
C	8.492394	-7.197596	7.228018
C	7.753963	-7.094543	4.921843
H	6.789202	-5.272231	4.286516
C	8.361862	-7.784140	5.977994
H	8.960482	-7.740025	8.044644
H	7.662612	-7.568018	3.948994
H	8.736108	-8.790471	5.815056
C	1.262922	-4.626240	9.694087
C	1.229931	-6.057530	9.063390

C	2.600752	-6.477030	8.568553
C	3.671268	-5.950651	9.515852
H	1.130950	-4.685440	10.794641
H	0.879031	-6.785428	9.810908
H	2.751646	-6.029762	7.574159
H	3.473283	-6.208217	10.560979
O	0.237405	-3.896187	9.064255
O	0.327619	-5.912084	7.989890
O	2.692518	-7.892534	8.554142
C	5.037343	-6.422023	9.074672
H	5.810884	-6.135471	9.789770
H	5.289610	-5.996213	8.091636
O	3.658034	-4.467040	9.452487
C	2.537973	-3.896435	9.519614
H	2.564108	-2.805130	9.455066
C	-0.558830	-4.830616	8.292898
C	-1.731255	-5.305597	9.132429
H	-2.384308	-4.463742	9.375207
H	-2.301858	-6.057479	8.579256
H	-1.387646	-5.748072	10.073334
C	-0.956031	-4.156927	7.002450
H	-1.546030	-4.848225	6.394070
H	-1.563404	-3.275222	7.224576
H	-0.055156	-3.862577	6.458751
O	4.992791	-7.844897	9.059515
C	3.995532	-8.399136	8.198880
C	3.950999	-9.877998	8.522929
H	3.170348	-10.363592	7.932644
H	4.916289	-10.334244	8.290954
H	3.735823	-10.011052	9.585634
C	4.318719	-8.146889	6.729059
H	5.307931	-8.555946	6.504387
H	3.571080	-8.640318	6.102084
H	4.333340	-7.087448	6.465236
C	3.312215	-4.548968	5.216331
H	4.231030	-5.019067	5.619037
C	2.470200	-5.612140	4.531705
H	3.050088	-6.128693	3.759728
H	2.116338	-6.351660	5.257012
H	1.592156	-5.152311	4.065089
C	3.726127	-3.432303	4.266836
H	2.837874	-2.941715	3.853461
H	4.325487	-2.678564	4.790222
H	4.322663	-3.828851	3.437238
O	2.531120	-4.035053	6.300043
H	2.941306	-3.211451	6.612613

Trichloroacetimidate:

C	-0.454001	0.349242	-0.192813
O	0.060041	1.362352	-0.607249
C	0.384462	-0.978994	-0.094020
N	-1.717073	0.248969	0.271790
H	-2.112911	-0.620840	0.585445
H	-2.270111	1.089546	0.304759
Cl	0.942395	-1.103744	1.617755
Cl	1.773299	-0.908897	-1.183548
Cl	-0.611487	-2.438880	-0.494495

Isopropanol:

C	0.599687	1.082824	-0.005473

H	0.935627	0.040620	-0.035622
H	0.968958	1.595652	-0.898383
H	-0.495180	1.094754	-0.033655
C	1.089364	1.777428	1.257201
H	2.190748	1.788617	1.257207
C	0.599630	1.083203	2.520127
H	0.935832	0.041089	2.550840
H	0.968614	1.596473	3.412916
H	-0.495241	1.094897	2.548111
O	0.593776	3.133822	1.256586
H	1.340423	3.737754	1.260622

6. References

1. Tsuiji, N.; Sidorov. P.; Zhu, C.; Nagata, Y.; Gimadiev, T.; Varnek, A.; List, B. Predicting Highly Enantioselective Catalysts Using Tunable Fragment Descriptors. *Angew. Chem., Int. Ed.* **2023**, *62*, e202218659

2. Leinung, W.; Tsuji, N.; Merher, M.; Leutzsch, M.; Raut, R. K.; List, B. Catalytic Asymmetric Ionic Hydrogenation of α-Alkyl Styrenes. *J. Am. Chem. Soc.* **2025**, *147*, 31463−31469.

3. Heuckendorff, M.; Jensen, H. H. Removal of Some Common Glycosylation By-Products during Reaction Work-Up. *Carbohydr. Res.* **2017**, *439,* 50–56.

4. Nagai, H.; Sasaki, K.; Matsumura, S.; Toshima, K. Environmentally benign β-stereoselective glycosidations of glycosyl phosphites using a reusable heterogeneous solid acid, montmorillonite K-10. *Carbohydr. Res.* **2005**, *340*, 337−353.

5. Rotta, M. K. V.; Moktan, S.; Prandhan, Pradhan, P.; Kancharla, P. K. Direct Stereoselective Synthesis of 2‑Deoxyglycosides via Visible-Light-Induced Photoacid-Catalyzed Activation of Glycosyl *o*‑[1‑(*p*‑MeO-Phenyl)vinyl]benzoates (PMPVBs) as Donors. *J. Org. Chem.* **2025**, *90*, 1196−1208.

6. Ghosh, T.; Mukherji, A.; Srivastava, H. K.; Kancharla, P. K. G Secondary amine salt catalyzed controlled activation of 2-deoxy sugar lactols towards alpha-selective dehydrative glycosylation. *Org. Biomol. Chem.* **2018**, *16*, 2870−2875.

7. Kimura, T.; Takahashi, D.; Toshima, K. Glycosylations of Glycals using *N*-Iodosuccinimide (NIS) and Phosphorus Compounds for Syntheses of 2-Iodo- and 2-Deoxyglycosides. *J. Org. Chem.* **2015**, *80*, 9552−9562.

8. Halder, S.; Addanki, R. B.; Moktan, S.; Kancharla, P. Glycosyl *o*‑[1‑(*p*‑MeO-Phenyl)vinyl]benzoates (PMPVB) as Easily Accessible, Stable, and Reactive Glycosyl Donors for *O*‑, *S*‑, and *C*‑Glycosylations under Brønsted Acid Catalysis. *J. Org. Chem.* **2022**, *87*, 7033−7055.

9. Kowalska, K.; Pedersen, C. M. α-Selective glycosylations using glycosyl *N*-(ortho-methoxyphenyl)trifluoroacetimidates. *Org. Biomol. Chem.* **2020**, *18*, 1918−1925.

10. Chretien, F.; Chapleur, Y.; Castro, B.; Gross, B. Alkyloxytris-(dimethylamino)-phosphonium Salts. Part 21. Anomeric Hydroxy-group Activation of 2,3:4,6-Di-*O*-isopropyl idene-α-*D*-mannopyranose, Thioglycosylation and Glycosylation. *J. Chem. Soc., Perkin Trans. 1*, **1980,** 381−384.

11. Maeda, S.; Taketsugu, T.; Morokuma, K. Exploring Transition State Structures for Intramolecular Pathways by the Artificial Force Induced Reaction Method. *J. Comput. Chem.* **2014**, *35*, 166−173.

12. Maeda, S.; Harabuchi, Y.; Takagi, M.; Taketsugu, T.; Morokuma, K. Artificial Force Induced Reaction (AFIR) Method for Exploring Quantum Chemical Potential Energy Surfaces. *Chem. Rec.* **2016**, *16*, 2232−2248.

13. Maeda, S.; Ohno, K.; Morokuma, K. Systematic Exploration of the Mechanism of Chemical Reactions: The Global Reaction Route Mapping (GRRM) Strategy Using the ADDF and AFIR Methods. *Phys. Chem. Chem. Phys.* **2013**, *15*, 3683−3701.

14. Grimme, S.; Bannwarth, C.; Shushkov, P. A Robust and Accurate Tight-Binding Quantum Chemical Method for Structures, Vibrational Frequencies, and Noncovalent Interactions of Large Molecular Systems Parametrized for All Spd-Block Elements (Z = 1-86). *J. Chem. Theory Comput.* **2017**, *13*, 1989−2009.

15. Neese, F. The ORCA Program System. *WIREs Comput. Mol. Sci.* **2012**, *2*, 73−78.

16. Grimme, S.; Hansen, A.; Ehlert, S.; Mewes, J.-M. r^2SCAN-3c: A "Swiss Army Knife" Composite Electronic-Structure Method. *J. Chem. Phys.* **2021**, *154*, 064103.

17. Neese, F. Software Update: The ORCA Program System—Version 5.0. *WIREs Comput. Mol. Sci.* **2022**, *12*, e1606.

18. Cossi, M.; Rega, N.; Scalmani, G.; Barone, V. Energies, Structures, and Electronic Properties of Molecules in Solution with the C-PCM Solvation Model. *J. Comput. Chem.* **2003**, *24,* 669−681.

19. Frisch, M. J. *et al*., "Gaussian 16 Rev. C.01" (Wallingford, CT, 2016); https://gaussian.com/g09_c01/.

20. Lu, T.; Chen, Q. Independent Gradient Model Based on Hirshfeld Partition: A New Method for Visual Study of Interactions in Chemical Systems. *J. Comput. Chem.* **2022**, *43*, 539−555.

21. Lu, T.; Chen, F. Multiwfn: A Multifunctional Wavefunction Analyzer. *J. Comput. Chem.* **2012**, *33*, 580−592.

22. Lu, T. A Comprehensive Electron Wavefunction Analysis Toolbox for Chemists, Multiwfn. *J. Chem. Phys.* **2024**, *161*, 082503.

23. Pettersen, E. F.; Goddard, T. D.; Huang, C. C.; Couch, G. S.; Greenblatt, D. M.; Meng, E. C.; Ferrin, T. E. UCSF Chimera—A Visualization System for Exploratory Research and Analysis. *J. Comput. Chem.* **2004**, *25*, 1605−1612.

24. Goddard, T. D.; Huang, C. C.; Meng, E. C.; Pettersen, E. F.; Couch, G. S.; Morris, J. H.; Ferrin, T. E. UCSF ChimeraX: Meeting Modern Challenges in Visualization and Analysis. *Protein Sci.* **2018**, *27*, 14−25.

25. Blender Online Community, "Blender - a 3D modelling and rendering package" (Stichting Blender Foundation, 2018); https://www.blender.org

26. Mardirossian, N.; Head-Gordon, M. ωB97M-V: A Combinatorially Optimized, Range-Separated Hybrid, Meta-GGA Density Functional with VV10 Nonlocal Correlation. *J. Chem. Phys.* **2016**, *144*, 214110.

27. Zheng, J.; Xu, X.; Truhlar, D. G. Minimally Augmented Karlsruhe Basis Sets. *Theor. Chem. Acc.* **2011**, *128*, 295−305.

28. Bickelhaupt, F. M.; Houk, K. N. Analyzing Reaction Rates with the Distortion/Interaction-Activation Strain Model. *Angew. Chem., Int. Ed.* **2017**, *56*, 10070−10086.

29. Huang, M.; Garrett, G. E.; Birlirakis, N.; Bohé, L.; Pratt, D. A.; Crich, D. Dissecting the Mechanisms of a Class of Chemical Glycosylation Using Primary ^{13}C Kinetic Isotope Effects. *Nat. Chem.* **2012**, *4*, 663−667.

30. Anderson, T.L.; Kwan, E.E. PyQuiver 2020, www.github.com/ekwan/PyQuiver

31. Adero, P. O.; Amarasekara, H.; Wen, P.; Bohé, L.; Crich, D. The Experimental Evidence in Support of Glycosylation Mechanisms at the S_N1-S_N2 Interface. *Chem. Rev.* **2018**, *118*, 8242−8284.
